# In Search of the Most Stable Molecular Configuration of Heptakis(2,6-*O*-dimethyl)-β-cyclodextrin and Its Complex with Mianserin: A Comparison of the B3LYP-GD2 and M062X-GD3 Results

**DOI:** 10.1021/acs.jpcb.1c06831

**Published:** 2021-11-24

**Authors:** Anna Ignaczak, Łukasz Orszański

**Affiliations:** Theoretical and Structural Chemistry Group, Department of Physical Chemistry, Faculty of Chemistry, University of Lodz, Pomorska 163/165, 90-236 Lodz, Poland

## Abstract

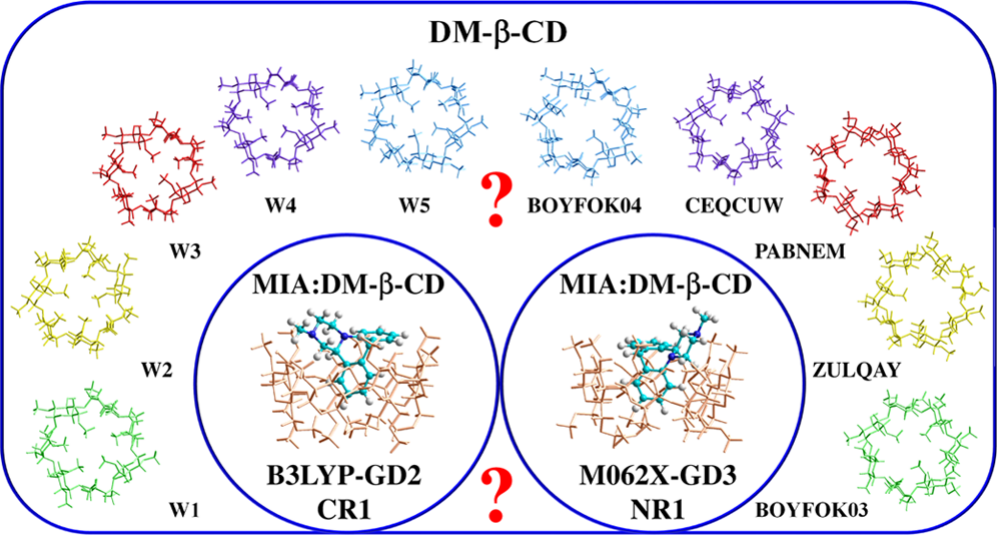

Cyclodextrins are well known for their ability to form stable, highly soluble complexes with various substances, which makes them widely used as excipients in food, cosmetics, and pharmaceuticals. In this work, properties of heptakis(2,6-*O*-dimethyl)-β-cyclodextrin (DM-β-CD) in vacuo and in water, as well as its ability to bind the antidepressant drug mianserin (MIA) in aqueous solution, are investigated computationally. The results are shown to depend strongly on the density functional theory (DFT) applied. The most stable conformers of DM-β-CD found with the B3LYP-GD2 method differ from these indicated by M062X-GD3 and other functionals. According to the latter, two crystal structures, ZULQAY and BOYFOK03, optimized in vacuo and in water, respectively, have the lowest energy. Both the B3LYP-GD2 and M062X-GD3 results show that all tested inclusion and noninclusion complexes of MIA:DM-β-CD in stoichiometry 1:1 are stable in water. However, the structures and their energetic properties obtained with each method differ: in the most stable configurations, different aromatic rings of MIA are embedded inside DM-β-CD, and the corresponding complexation energies (calculated with the 6-31++G(d,p) basis set and corrected for the basis set superposition error) are −29.6 (B3LYP-GD2) and −23.9 (M062X-GD3) kcal/mol. The NMR spectra of DM-β-CD and MIA:DM-β-CD are also compared.

## Introduction

**Chart 1:**
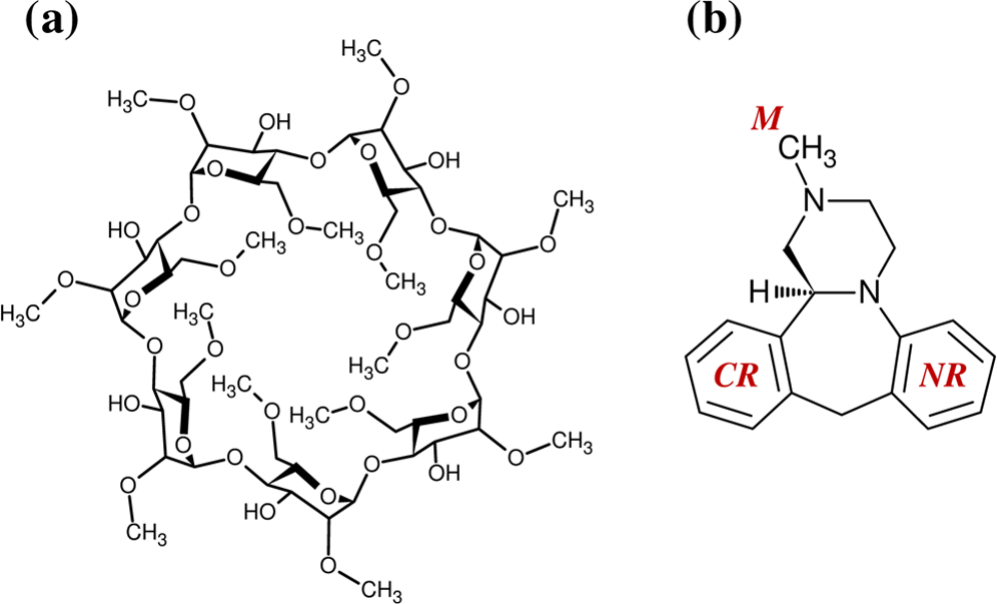
Structures of: (a) DM-β-CD and (b) Mianserin (MIA). The Symbols CR, NR, and M Indicate Fragments of the MIA Molecule (The Two Aromatic Rings and Methyl Group, Respectively) Entering the Cyclodextrin Cavity in the Inclusion Complexes

Cyclodextrins (CDs) are cyclic oligosaccharides, which, due to their toroidal shape and ability to form stable, water-soluble inclusion complexes with various organic molecules, in the last decades found many applications in chemistry, cosmetic and food industry, as well as in pharmacy and medicine.^1−9^ The solubility of natural cyclodextrins (α-, β-, and γ-CDs) in water at the room temperature is not very high (145, 18.5, and 232 mg/mL, respectively) and among them, β-CD seems to be the worst candidate for a drug carrier. However, the size of its central (hydrophobic) cavity with a diameter of 6.0–6.5 Å is very suitable for the inclusion of many drugs, and its solubility can be significantly enhanced by replacing some of the hydroxyl groups with various substituents.^1,10−12^ One such derivative is heptakis(2,6-*O*-dimethyl)-β-cyclodextrin (DM-β-CD; Chart 1a). Its solubility in water is 570 mg/mL, so it is about 30 times greater than that of β-CD, while the diameter of its cavity is practically the same as in the native cyclodextrin.^8^

Despite these undoubted advantages and a wide range of possible practical applications, knowledge about this molecule is quite limited. In several works, the crystal structure of DM-β-CD, mainly in the form of clathrate hydrates with various number of water molecules, was studied experimentally.^13−17^ The authors point out some common features of methylated CDs in the crystal lattice, such as (a) two or three of the O6-CH_3_ groups are rotated “inward”, closing the cavity on one side of the cone; (b) the cavity can be either empty or filled by the O6-CH_3_ groups of another methylated CD; and (c) the space between the methylated CDs can be either empty or occupied by one or two water molecules.^16^

However, the structure and other properties of DM-β-CD in crystals are determined by interactions with similar molecules, while its characteristics in the gas or liquid phase can be very different and thus have a large influence on its chemical behavior, but they were not explored in the past. To our knowledge, only one attempt was made to find the ground state of DM-β-CD in vacuo at the density functional theory (DFT) level, but the results were only briefly described in the conference abstract.^18^ There are also some other theoretical studies concerning the DM-β-CD molecule, but they mostly focus on its inclusion complexes, in which the host geometry is one of the various structures found experimentally or just created in molecular modeling programs.^19−25^ The theory level applied also differs in these works. This makes it difficult to compare the stability of various complexes because the choice of the reference geometry (i.e., isolated reactants) and the quantum method can have a great impact on the obtained complexation energies, as demonstrated in our recent studies of systems containing cyclodextrins.^26,27^

In one of these works, the complex of the native β-CD with mianserin (MIA) was investigated.^26^ Mianserin is a tetracyclic drug used for the treatment of depression and insomnia.^28−31^ It is usually administrated as a racemate of (S)- and (R)-enantiomers, but S-MIA is suggested to be more potent.^32,33^ The results of DFT calculations performed in ref (26) showed that MIA forms very stable, both inclusion and noninclusion complexes with β-CD, but contrary to expectations, one of the noninclusive complexes turned out to be energetically preferred. It is interesting whether the analogous complexes of MIA with DM-β-CD exhibit similar characteristics and whether their stability differs from that of MIA:β-CD. In the literature, there is very little information about the MIA:DM-β-CD systems, mainly concerning the use of native β-CD and its derivatives for the enantiomeric separation of mianserin and its analogues chiral pharmaceuticals.^34−36^

The aim of this study is to broaden the knowledge about properties of the DM-β-CD molecule in vacuo and in water as well as its complex with MIA in aqueous solution using the theoretical approach. For both systems, an attempt is made to find their lowest energy configurations and characterize them at the density functional theory level. Additionally, the dependence of the results on the DFT method applied in the calculations is examined. This includes a comparison of the relative energies of various conformers of DM-β-CD calculated using seven different density functionals and, for both systems, a more detailed comparative analysis of the preferred structures, their energies, as well as the NMR spectra obtained with two DFT methods: B3LYP-GD2 and M062X-GD3. The influence of diffuse functions on the molecular geometries and their stabilization energies is also analyzed.

## Computational Details

The conformational space of the DM-β-CD molecule was explored using the “hierarchical approach”, in which the accuracy of the theoretical methods was gradually increased in three subsequent steps. The procedure is shown in the flow chart [Figure S1 in the Supporting Information (SI)] and described in detail in the section Procedure in SI; therefore, only its most important points are presented below. In the first step, from the initial, regular geometry [Figure S2a (SI)], a large number of different structures were created by varying selected torsion angles using the molecular mechanics methods and the Conformational Search module available in the program Hyperchem.^37^ In the second step, these structures were re-optimized in the program MOPAC^38^ using the semiempirical methods PM6 and PM7 in vacuo and in water (COSMO model^39^). At this stage, to refine the search for the most stable structures, molecular dynamics simulations were performed additionally in the program Gabedit^40^ combined with MOPAC. In the third step, the lowest energy conformers selected after all semiempirical calculations (60 structures in vacuo and 60 in water) were fully optimized in the Gaussian 09 program^41^ using the 6-31G(d,p) basis set and the B3LYP-GD2 method that is the standard B3LYP functional^42^ including the Grimme empirical pairwise long-range (dispersion) corrections.^43^ The DFT calculations were performed, respectively, in vacuo and in water, with the solvent being described by the polarizable continuum model (PCM).^44^

Additionally, the single-point (SP) calculations and optimizations (OPT) in vacuo and in water were carried out for the five DM-β-CD structures extracted (by removing surrounding molecules and adding the missing hydrogens) from the experimental data available in the Cambridge Crystallographic Data files (CCDF; structures crystallized from water at given temperatures): ZULQAY—the anhydrous DM-β-CD at 60 °C,^13^ CEQCUW-DM-β-CD·2H_2_O at 18 °C,^14^ BOYFOK03-DM-β-CD·15H_2_O at 4 °C,^15^ BOYFOK04-DM-β-CD·15H_2_O at 18 °C^16^^16^ and PABNEM—the complex of DM-β-CD with (2,4-dichlorophenoxy)acetic acid at 50 °C.^17^

The quality of the results obtained with the B3LYP-GD2/6-31G(d,p) method was further verified by performing the single-point (SP) calculations using the 6-31G(d,p) basis set and six other DFT methods available in the Gaussian 09 package: M05-GD3,^45,46^ M06-GD3,^47^ M062X-GD3,^47^ ωB97XD,^48^ mPW1PW91,^49^ and M11.^50^ They were chosen based on the results of the work of Boese,^51^ who tested various functionals for the set of 49 complexes containing hydrogen bonds and, for the methods listed above, reported small root-mean-square errors with respect to the CCSD(T)/CBS reference values. The single-point calculations were performed for the set of ten structures containing the five conformers having the lowest B3LYP-GD2 energies after the conformational search and the five optimized crystal structures. Since these tests showed differences between the results obtained with the B3LYP-GD2 and other DFT methods, all ten structures were re-optimized with the M062X-GD3/6-31G(d,p) method. Additionally, to evaluate the effect of the diffuse functions on the relative energies for the optimized structures, the single-point calculations were performed with the B3LYP-GD2 and M062X-GD3 methods and the 6-31++G(d,p) basis set.

For the conformers obtained from the B3LYP-GD2/6-31G(d,p) and M062X-GD3/6-31G(d,p) optimizations, the vibrational analysis was performed. The thermodynamic calculations were done at 298.15 K and 1 atm of pressure. Gibbs energies were additionally recalculated with the program GoodVibes v2.0.3^52^ using the Grimme approach^53^ to include the corrections for low vibrational frequencies (wavenumbers <100 cm^–1^). In water, additional corrections were applied to account for the concentration change to 1 mol/L and for the change of volume available to each molecule in solution when compared to the gas phase.^54^ The corrected Gibbs energy values are denoted below as *G*_corr_. The final IR spectra were obtained using the Gabedit program and the Lorentzian convolution with the half-width 20. The wavenumbers were scaled by 0.961 (B3LYP-GD2)^55^ and 0.951 (M062X-GD3).^56^

For the conformers obtained in water, NMR calculations were performed using the gauge-independent atomic orbital (GIAO) approach^57^ at the B3LYP/6-31++G(d,p)//B3LYP-GD2/6-31G(d,p) and M062X-GD3/6-31++G(d,p)//M062X-GD3/6-31G(d,p) theory levels. The final chemical shifts δ (relative to tetramethylsilane, TMS) were obtained by applying the procedure proposed by Tantillo,^58^ in which they are defined as: δ = (*I* – σ)/(−*S*), where σ are the isotropic values obtained from DFT calculations, and *I* and *S* are the scaling factors obtained with the same method. The detailed description of the procedure used to calculate the scaling factors *I* and *S* as well as their values obtained with the method B3LYP/6-31++G(d,p)//B3LYP-GD2/6-31G(d,p) in water (PCM) are given in ref (59). The parameters obtained in the present work for the method M062X/6-31++G(d,p)//M062X-GD3/6-31G(d,p) in water are: *S*_H_ = −1.1610, *I*_H_ = 32.0341 and *S*_C_ = −1.1036, *I*_C_ = 198.2318.

To assess the complexation energies of MIA with DM-β-CD in water, a strategy similar to that applied in the earlier work of one of us where the complexes of MIA with β-CD were studied,^26^ was used. The procedure is presented in the flow chart in Figure S3 (SI). In the complexes, as the initial structure of DM-β-CD, the most stable structure found in water was used, denoted below as W1. For mianserin, it was the S-MIA enantiomer obtained in ref (26) from the B3LYP/6-31G(d,p) optimization in water. With these molecules, using the Hyperchem program, 11 different structures of the complexes MIA:DM-β-CD (1:1) were built, in which MIA was placed at different positions with respect to the DM-β-CD cone, as shown in Figure S4 (SI). For each configuration, further structures were produced using the Hyperchem program by a systematic rotation of MIA around each axis *X*, *Y*, and *Z*, changing the angle stepwise by 10°. The resulting 1188 different structures (108 different structures for each initial configuration) were fully optimized in vacuo using the semiempirical PM6 method. Next, they were ordered according to the increasing heat of formation, and among them were selected 11 configurations, each being a representative of one orientation of MIA, as shown in Figure S4 (SI) and having the lowest energy within a set of similar structures corresponding to this particular configuration. The eleven selected structures were next fully optimized without constraints in water (PCM) using the DFT method B3LYP-GD2/6-31G(d,p) in the Gaussian 16 program.^60^ The whole procedure was repeated, this time using the lowest conformer indicated by the M062X-GD3/6-31G(d,p) method (BOYFOK03 optimized in water) in the initial model of the complex and optimizing the 11 configurations selected in the final step with this DFT method.

The complexation energies were calculated according to *E*_compl_ = *E*_MIA:DM-β-CD_^OPT^ – (*E*_MIA_^OPT^ + *E*_DM-β-CD_^OPT^), where *E*_MIA:CD_^OPT^, *E*_MIA_^OPT^, and *E*_CD_^OPT^ are the total energies of the optimized complex and its isolated components, mianserin and DM-β-CD, in their most stable geometries. An effect of the basis set superposition error (BSSE) on the complexation energies was calculated using the counterpoise correction.^61^

## Results and Discussion

**Figure 1:**
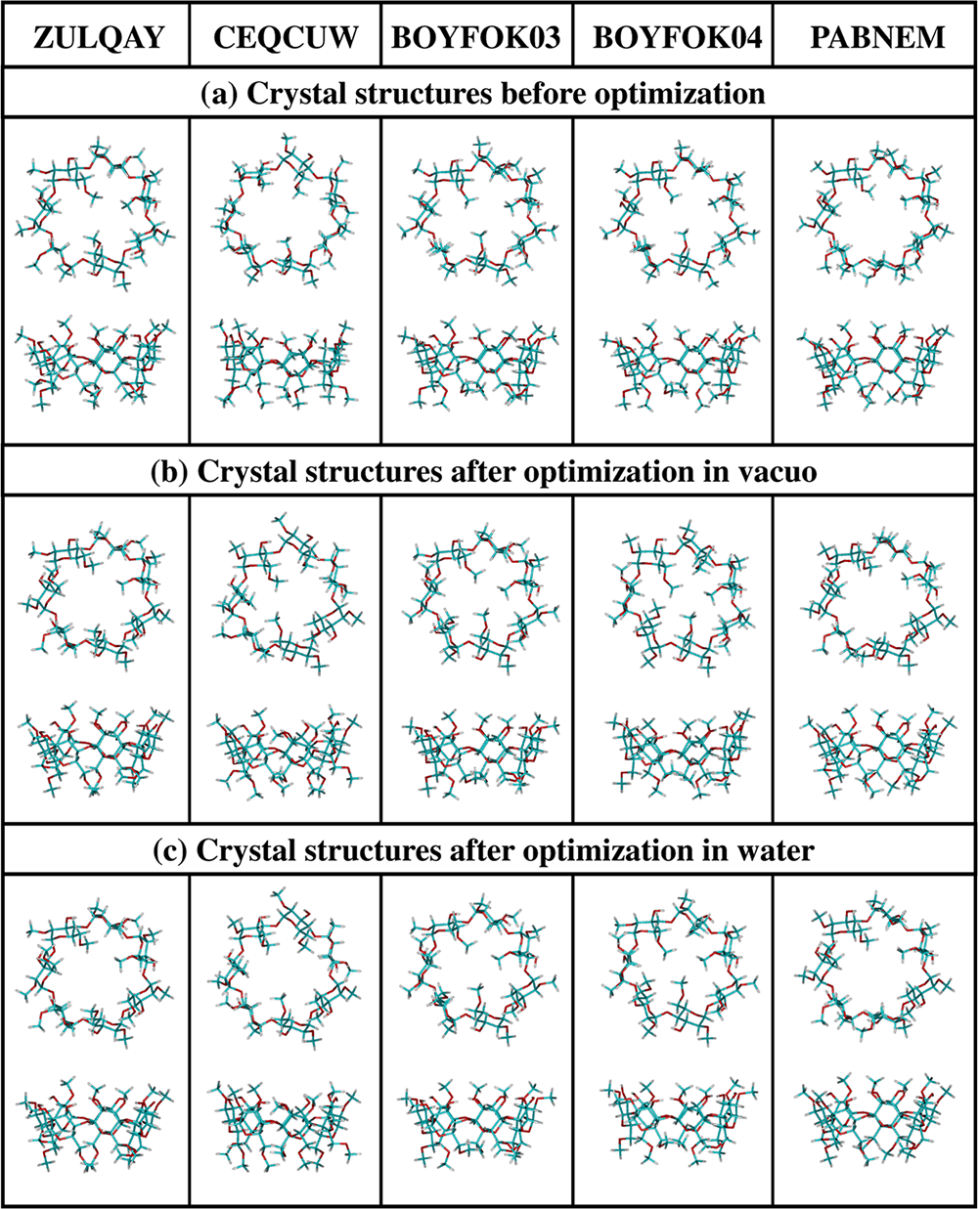
Crystal structures of DM-β-CD: (a) original geometries from CCDF, (b) optimized with the B3LYP-GD2/6-31G(d,p) method in vacuo, and (c) optimized in water (PCM). In all cases, the top (from the narrow side of the cone) and side views are shown.

**Figure 2:**
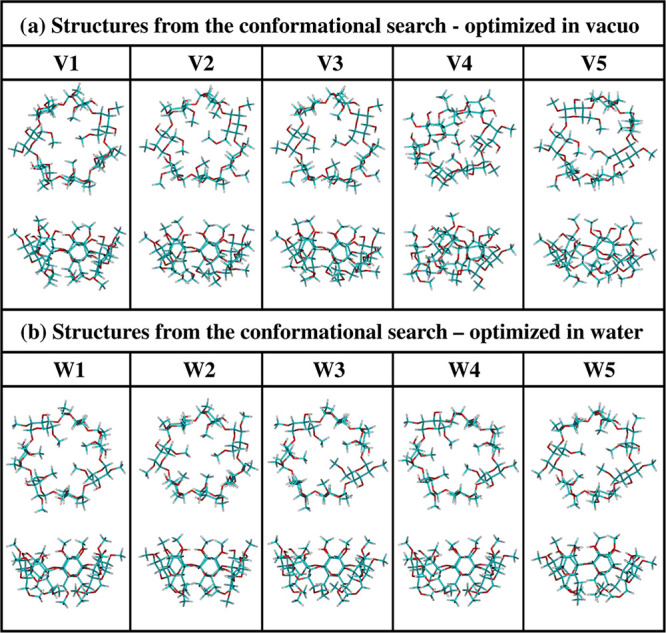
Most stable structures of DM-β-CD obtained from the conformational search and optimized with the B3LYP-GD2/6-31G(d,p) method: (a) in vacuo and (b) in water (PCM). In all cases, the top (from the narrow side of the cone) and side views are shown.

The original crystal structures from CCDF and the geometries obtained from their B3LYP-GD2/6-31G(d,p) optimization in vacuo and in water are presented in Figure 1, while in Figure 2 are shown the structures found from the conformational search. The corresponding relative energies are listed in Table 1. As can be seen in Figure 1a, the crystal structures have two or three O6-CH_3_ groups rotated inward, partially closing one side of the DM-β-CD cone, the structures CEQCUW and PABNEM being the most “open”. In all of these molecules, the O3-H···O2′ hydrogen bonds are formed between the neighboring glucose units. However, as already mentioned, the structure of a single molecule in a liquid or gas phase can be very different than its geometry in crystals, where it is subjected to specific interactions with closely adjacent molecules.

**Table 1:** B3LYP-GD2/6-31G(d,p) Relative Energies Δ*E* (Without Zero-Point Energy (ZPE) Corrections), Enthalpies Δ*H*, and Corrected Gibbs Energies Δ*G*_corr_ Calculated with Respect to the Lowest Energy Conformers of DM-β-CD. SP and OPT Denote the Results Obtained after Single-Point Calculations and the Geometry Optimization, Respectively^a^

structure	SP Δ*E*	OPT Δ*E*	OPT Δ*H*	OPT Δ*G*_corr_	SP 6-31++G(d,p) Δ*E*
in VACUO					
ZULQAY	38.62	2.78	1.67	0.07	0.46
CEQCUW	839.43	9.76	8.68	7.37	7.29
BOYFOK03	0.00	3.78	2.92	2.23	2.38
BOYFOK04	853.71	5.48	5.03	5.29	3.77
PABNEM	638.01	2.15	1.04	0.00	0.31
V1		0.00	0.00	1.44	0.00
V2		0.31	0.57	2.01	1.73
V3		1.28	1.25	2.22	2.66
V4		1.28	1.97	4.29	6.16
V5		1.40	1.93	3.45	5.05
in WATER (PCM)					
ZULQAY	44.86	5.13	3.50	1.51	3.69
CEQCUW	835.15	10.44	8.53	6.02	7.78
BOYFOK03	0.00	2.93	1.67	0.00	1.80
BOYFOK04	848.11	5.69	4.51	3.61	4.50
PABNEM	640.88	5.89	4.03	1.97	4.89
W1		0.00	0.00	1.74	0.00
W2		0.36	0.04	1.15	0.28
W3		0.85	0.63	1.77	1.73
W4		1.44	1.72	3.85	2.28
W5		2.41	2.70	4.64	2.67

a The last column contains the results of SP calculations performed with 6-31++G(d,p) for the optimized structures. All values are in kcal/mol.

Indeed, the B3LYP-GD2 optimization of the crystal structures in vacuo and in water leads to conformers whose energy is lower by at least 200 kcal/mol or even much more. Among the original crystal structures (Figure 1a), BOYFOK03 has the lowest energy both in vacuo and in water. After optimization, the structure corresponding to PABNEM has the lowest energy in vacuo (Figure 1b), while in water (Figure 1c), BOYFOK03 remains favorable [Tables 1 and S1 (SI)].

The conformational search followed by the B3LYP-GD2 optimizations indicated several structures having energy *E* lower by several kilocalories per mol than the optimized crystal structures (Figure 2 and Table 1). All conformers found from this search contain from 3 to 5 O6-CH_3_ groups rotated inward at the more narrow rim of the cone, which practically close this entrance. The most stable conformers in vacuo and in water are V1 and W1, respectively, and the energy of the four other structures, V2–V5 and W2–W5, is higher by no more than 3 kcal/mol (Table 1).

**Figure 3:**
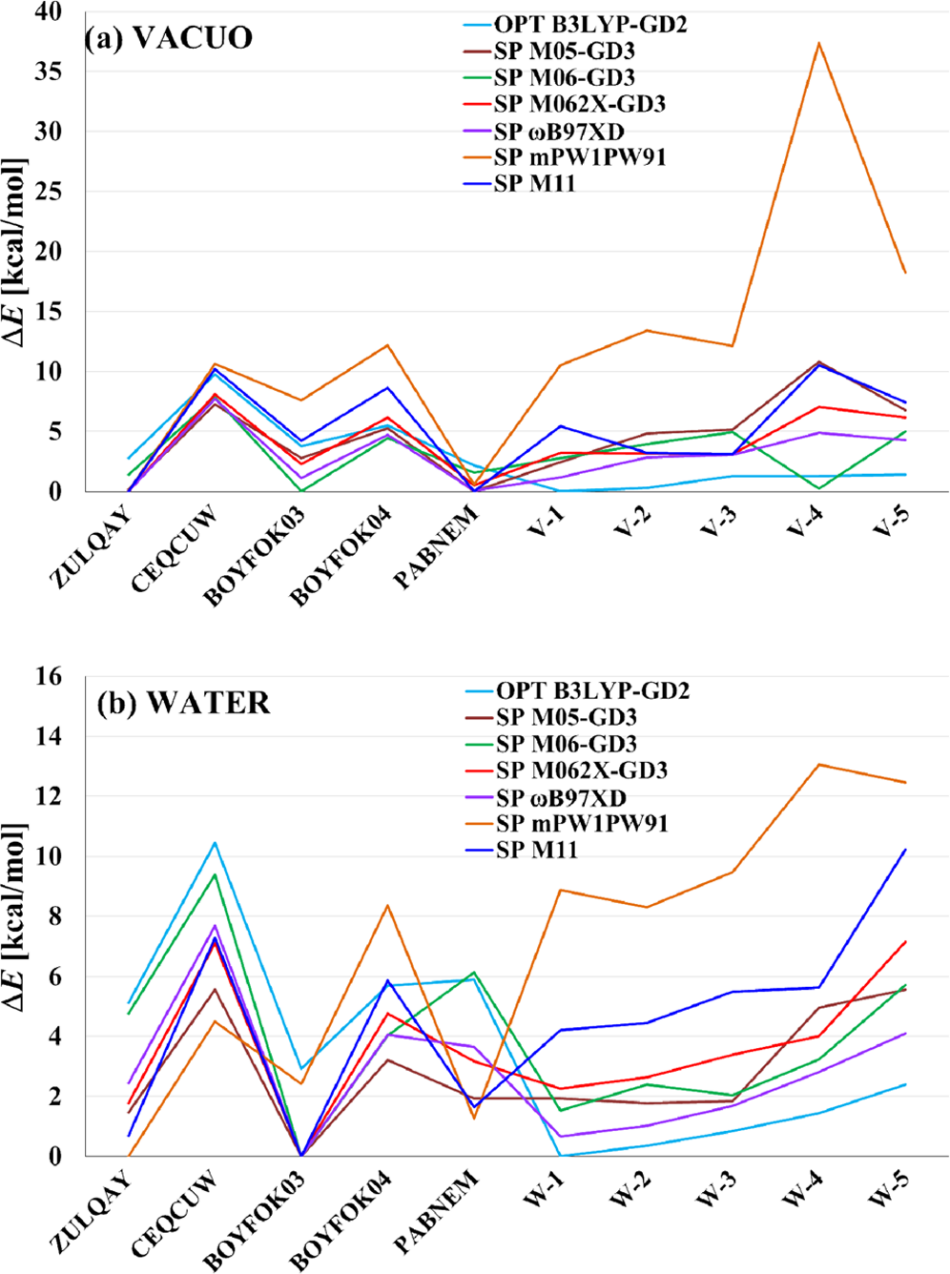
Relative energies obtained for various conformers of DM-β-CD from the B3LYP-GD2/6-31G(d,p) optimization (OPT) in vacuo (a) and in water (b) and from the corresponding single-point (SP) calculations performed with six other DFT methods and the same basis set.

However, the results of SP calculations performed with other DFT methods and the 6-31G(d,p) basis set for the B3LYP-GD2 optimized structures do not confirm V1 and W1 to be the most stable, which is shown in Figure 3, Tables S2, and S3 (SI). The relative energies Δ*E*, as well as the trends seen in the series SP, strongly depend on the functional used; nevertheless, each of them indicates one of the crystal (optimized) structures. In vacuo, four methods (M062X-GD3, ωB97XD, mPW1PW91, and M11) predict ZULQAY to be energetically favorable, while two other methods suggest BOYFOK03 (M06-GD3) and PABNEM (M05-GD3). In water, most methods predict that BOYFOK03 has the lowest energy, except for mPW1PW91 that points toward ZULQAY. The results obtained with the latter functional generally differ from the rest, yielding high Δ*E* values for various conformers. In the case of other methods, the divergences are smaller but still substantial. Concerning the conformers V1 and W1, the differences between their energy and the most stable conformer indicated by a given method range in vacuo from 1.17 (ωB97XD) to 5.44 kcal/mol (M11), while in water they are smaller: from 0.68 (ωB97XD) to 4.22 kcal/mol (M11). Most tested methods confirm the increasing energy trend when going from W1 to W5 and indicate that the crystal structure CEQCUW has the highest energy compared to all other structures.

To verify whether indeed the optimized crystal structures ZULQAY and BOYFOK03 are more stable than V1 and W2, the conformers shown in Figures 1b,c and 2b,c were re-optimized with the method M062X-GD3/6-31G(d,p) [Tables 2 and S4 (SI)]. As can be seen, the re-optimization has little effect on the relative energies and trends reflected already in the SP results. The largest change is noted for the structure CEQCUW in vacuo, for which Δ*E* is lowered from 8.12 to 4.37 kcal/mol.

**Table 2:** M062X-GD3/6-31G(d,p) Relative Energies Δ*E* (Without ZPE Corrections), Enthalpies Δ*H*, and Corrected Gibbs Energies Δ*G*_corr_ Calculated with Respect to the Lowest Energy Conformers of DM-β-CD^a^

structure	SP Δ*E*	OPT Δ*E*	OPT Δ*H*	OPT Δ*G*_corr_	SP 6-31++G(d,p) Δ*E*
in VACUO					
ZULQAY	47.80	0.00	0.00	0.00	0.00
CEQCUW	836.78	4.37	4.42	5.52	4.33
BOYFOK03	0.00	1.96	2.02	3.42	2.48
BOYFOK04	850.78	6.32	6.55	7.97	6.11
PABNEM	640.79	0.18	0.19	0.77	0.25
V1		3.23	3.64	6.12	4.11
V2		2.95	3.62	6.54	5.18
V3		3.23	3.91	6.80	5.55
V4		6.53	6.82	10.62	9.24
V5		5.26	6.07	9.24	8.02
in WATER (PCM)					
ZULQAY	54.23	1.55	1.25	0.40	1.24
CEQCUW	832.56	6.69	6.29	5.09	5.23
BOYFOK03	0.00	0.00	0.00	0.00	0.00
BOYFOK04	845.33	3.71	3.54	4.02	3.49
PABNEM	644.06	2.81	2.30	1.17	2.27
W1		2.47	2.62	4.06	2.25
W2		2.72	2.80	3.95	2.39
W3		3.07	3.15	4.99	3.25
W4		4.11	4.59	7.30	4.51
W5		7.03	7.28	9.21	6.73

a The last column contains the results of SP calculations performed with 6-31++G(d,p) for the optimized structures. All values are in kcal/mol.

**Figure 4:**
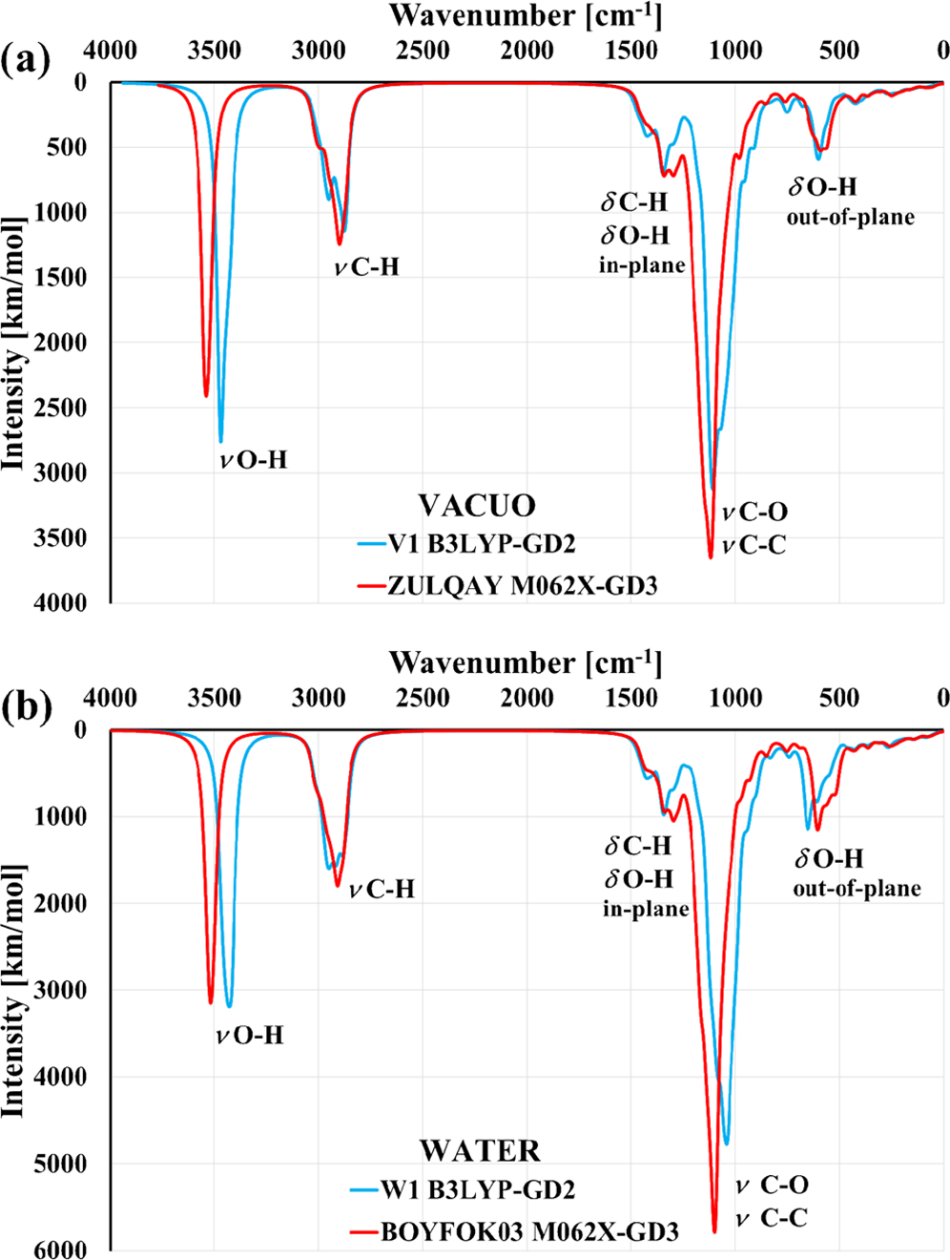
IR spectra obtained from the DFT calculations in vacuo (a) and in water (b) for the DM-β-CD structures predicted as the most stable in vacuo and in water by the methods B3LYP-GD2 (V1 and W1) and M062X-GD3 (ZULQAY and BOYFOK03).

Since the ωB97XD results suggested small energy differences between ZULQAY and V1 in vacuo, and especially between BOYFOK03 and W1 in water, an additional test was performed by re-optimizing these four structures at the ωB97XD/6-31G(d,p) theory level. The resulting relative energies Δ*E* are 3.22 (V1-ZULQAY in vacuo) and 0.27 kcal/mol (W1-BOYFOK03 in water), so they qualitatively confirm the greater stability of the optimized crystal structures. As shown in Figure S5 (SI), in all cases, the re-optimized structures remain similar to these obtained from the B3LYP-GD2 optimization, the largest root-mean-square deviation (RMSD) of atomic positions of 0.191 Å is obtained for ZULQAY optimized in vacuo with the M062X-GD3 method. For comparison, the RMSD values, calculated for different conformers obtained from the M062X-GD3 optimizations, are 7.475 (V1-ZULQAY) and 6.330 Å (W1-BOYFOK03). Very similar values (7.480 and 6.329 Å) are obtained when the B3LYP-GD2 geometries V1 and W1 are compared to the M062X-GD3 structures ZULQAY and BOYFOK03. Despite this, their IR spectra are very similar (Figure 4). Some discrepancies in the intensity and peak shifts are related to the density functional and scaling factors used rather than to structure differences, as they are not observed in the spectra calculated with the same DFT method [Figure S6 (SI)].

Also, adding diffuse functions to the basis set does not significantly affect the geometry of DM-β-CD, which is shown in Figure S7 (SI), where the lowest energy conformers optimized with a given method and two basis sets, 6-31G(d,p) and 6-31++G(d,p), are compared. The RMSD values are very small, reflecting mainly the displacement of some hydrogen atoms due to the slight rotation of methyl groups. Therefore, to assess the influence of diffuse functions on the relative energies for all analyzed structures, only the single-point calculations were performed using the B3LYP-GD2 and M062X-GD3 methods and the 6-31++G(d,p) basis function. The resulting relative energies are included in Tables 1 and 2, while in Figure S8a,b (SI), they are compared to those obtained with the smaller basis set. For most conformers, the impact of diffuse functions on the B3LYP-GD2 relative energies is larger than on the M062X-GD3 values; however, this does not affect the overall trends, which in both cases remain unchanged.

The distribution of the M062X-GD3 relative Gibbs energies Δ*G*_corr_ is similar to that of Δ*E*, while some differences appear in the B3LYP-GD2 series [Tables 1, 2 and Figure S8c,d (SI)]. In the latter case, due to the entropy effects in vacuo, the lowest Gibbs energy has the PABNEM optimized structure, while in water, it is BOYFOK03. As can be seen in Figure S8d (SI), in most cases, the values Δ*G*_corr_ obtained with both methods in water are relatively small, which suggests that at the standard temperature, the conformational interconversion should occur rather easily; therefore, different conformers of DM-β-CD may coexist in the liquid phase.

**Figure 5:**
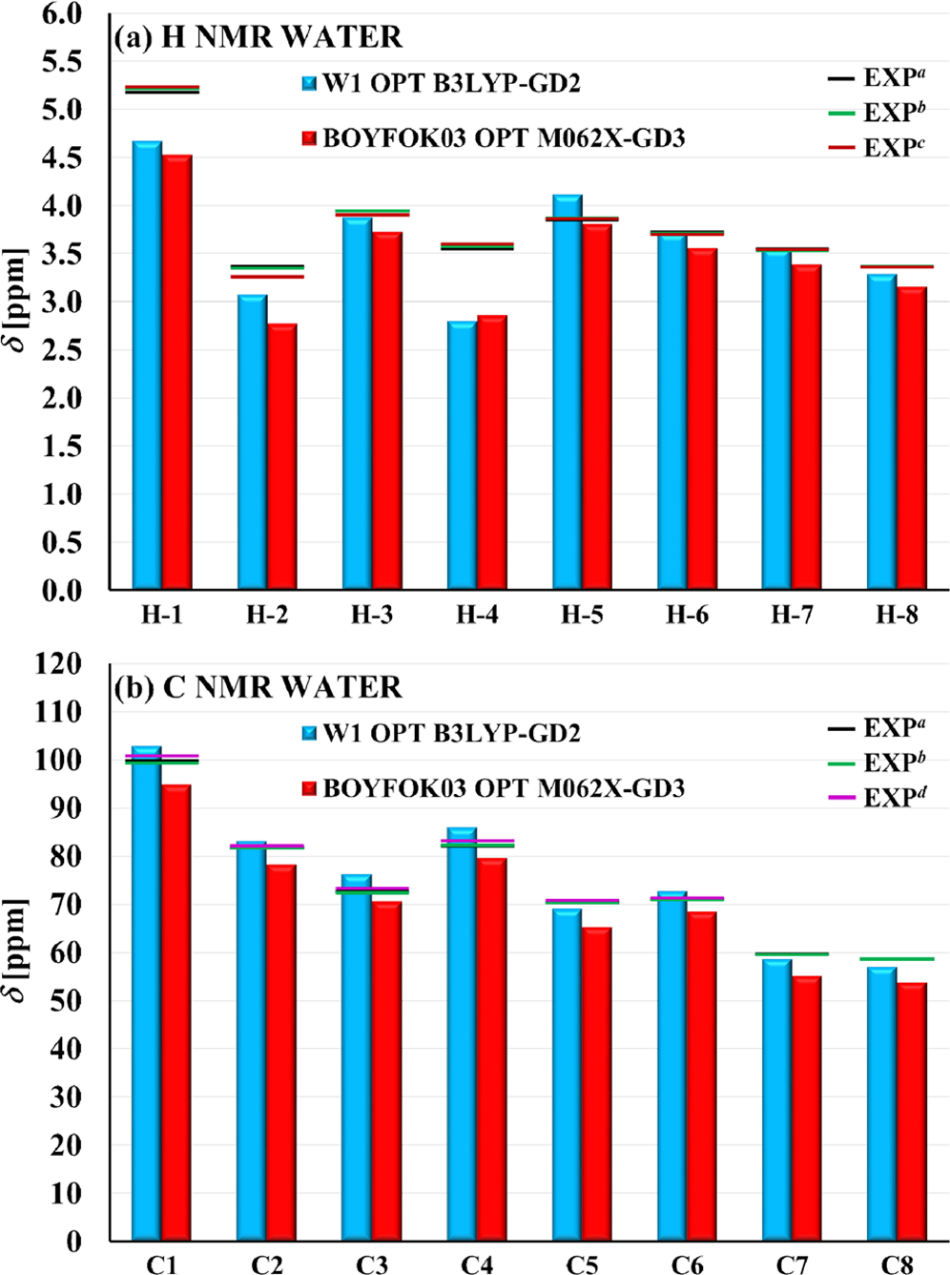
Computed ^1^H (a) and ^13^C (b) NMR chemical shifts δ (calculated as averages over δ values for all equivalent atoms present in DM-β-CD) obtained in water for the structures: W1 optimized with the B3LYP-GD2 method and BOYFOK03 optimized with the M062X-GD3 method, compared to the experimental values (EXP) measured in D_2_O (horizontal lines): ^*a*^ ref (63), ^*b*^ ref (64), ^*c*^ ref (65), ^*d*^ ref^66^. The linear regression analysis between the experimental and calculated values, as well as the corresponding coefficients of determination *R*^2^, are shown in Figure S10 (SI).

This conclusion seems to be supported by a comparison of the NMR chemical shifts calculated in water to the experimental values obtained in D_2_O (Figure 5; the δ values for each atom in these structures are given in Table S5 (SI)). Neither the results B3LYP for W1 nor M062X for BOYFOK03 show perfect agreement with the measured ^1^H and 13C NMR spectra, although both reflect the general trends present in them. For most atoms, the B3LYP chemical shifts are higher than M062X, but the pattern is similar, and in both cases, the largest deviations are observed for the protons H-1, H-2, and H-4 [Figure 5a; for atom numbers see Figure S2b (SI)]. These are the hydrogen atoms from the glucose rings, which belong to the exterior surface of the cyclodextrin cone. The more detailed analysis of chemical shifts for the protons H-1, H-2, and H-4 in all conformers considered in the present work shows that for none of them, the calculated results are completely consistent with the experimental data [Figure S9a–c (SI)]. The B3LYP ZULQAY SP results show the best fit for proton chemical shifts, but at the same time, its δ values for carbons deviate much more from the reference level than in the other structures [Figure S9d–f (SI)].

As well known, the molecule environment influences the ^1^H NMR spectra, and this effect was observed also for DM-β-CD: the chemical shifts measured in DMSO-*d*_6_ were found to be lower than in D_2_O.^62,63^ Thus, the discrepancies between the theoretical and measured values can also be related to a very approximate description of the solvent effect (PCM) in the quantum calculations, as it does not account properly for specific solute–solvent interactions (e.g., formation of hydrogen bonds between DM-β-CD and H_2_O molecules). Additionally, as can be seen in Tables 1 and 2, the relative energy differences corresponding to the successive local minima are rather small. It is therefore very likely that in the liquid phase, many different structures coexist and contribute to the measured NMR spectra.

**Figure 6:**
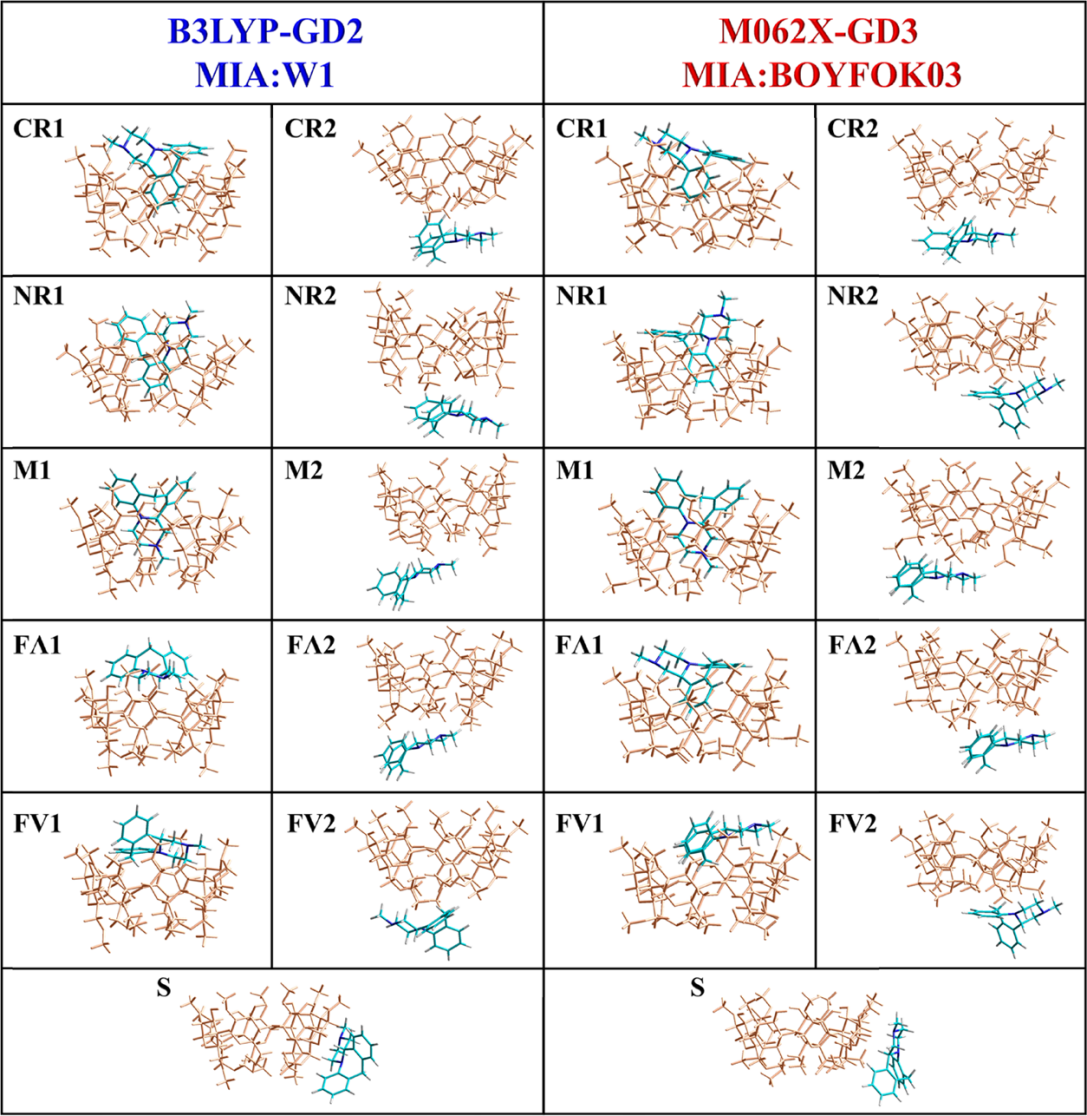
Structures of the MIA:DM-β-CD complex in the eleven configurations obtained after the B3LYP-GD2/6-31G(d,p) and M062X-GD3/6-31G(d,p) optimizations; the symbols MIA:W1 and MIA:BOYFOK03 indicate which of the DM-β-CD conformers were used in the initial model of the complex, CR, NR, and M—the fragments of the MIA molecule (Chart 1b) inserted in the interior cavity of DM-β-CD, FΛ and FV— two different “flat” orientations of MIA, numbers 1, 2, and the label S—different sides of the cyclodextrin cone.

**Figure 7:**
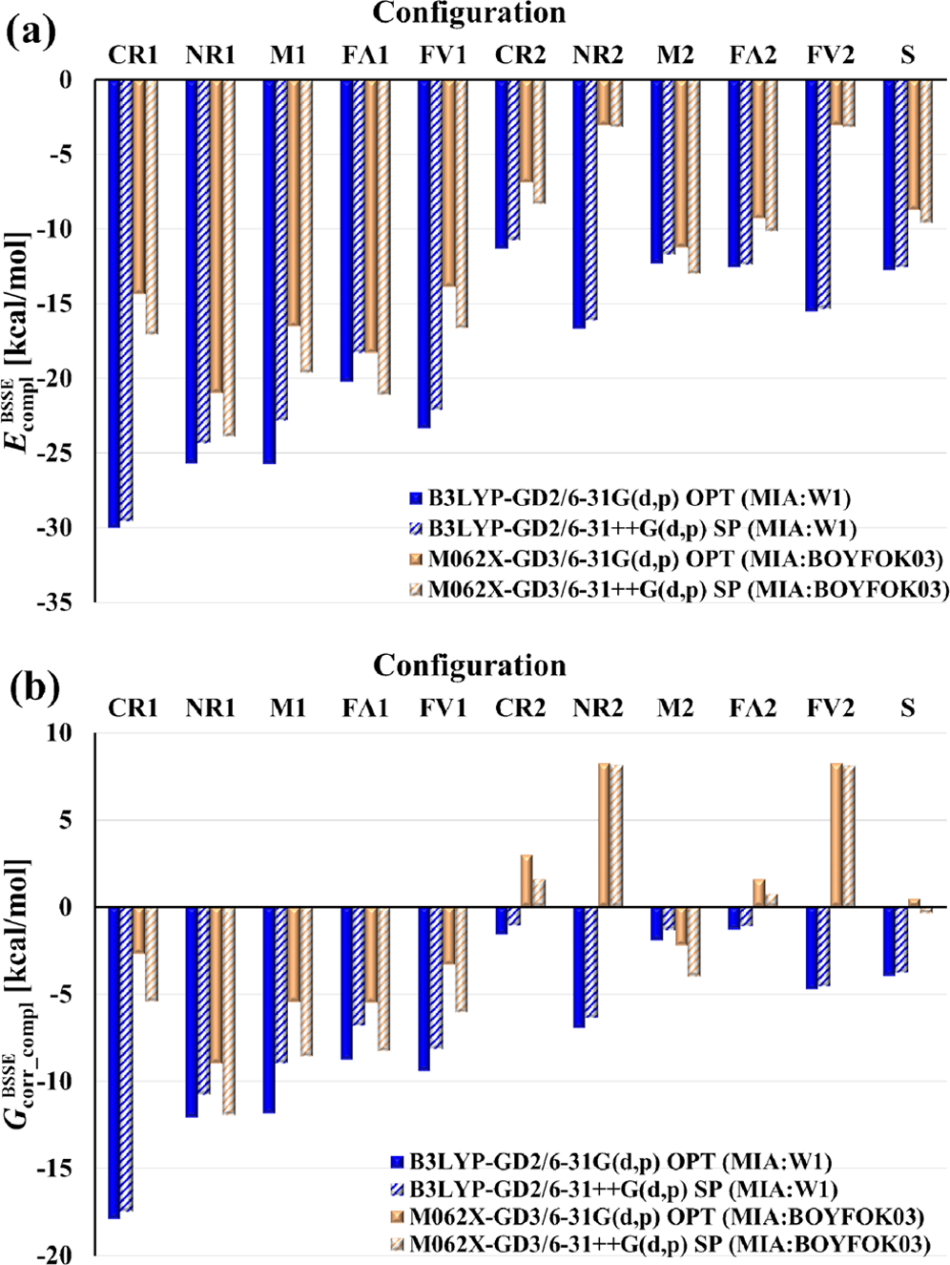
BSSE-corrected complexation energies *E*_compl_^BSSE^ and Gibbs energies *G*_corr_compl_^BSSE^ for MIA:DM-β-CD in the eleven configurations obtained in water (PCM) from the B3LYP-GD2/6-31G(d,p) and M062X-GD3/6-31G(d,p) optimizations (OPT), and the corresponding values (also BSSE-corrected) obtained from the single-point calculations performed with the same methods and the 6-31++G(d,p) basis set.

Another important property of DM-β-CD studied in the present work is its capability to form the complex with mianserin. In Figure 6 are compared the eleven structures obtained from the B3LYP-GD2 and M062X-GD3 calculations. In each set, the initial model of the complex was based on a different cyclodextrin conformer indicated by the given method. In the first set it was W1, and in the second, optimized BOYFOK03. The corresponding BSSE-corrected complexation energies and free enthalpies for both sets are compared in Figure 7. As for DM-β-CD, also for the complex, an effect of diffuse functions was investigated by performing the single-point calculations with the 6-31++G(d,p) basis set for the optimized structures of MIA:DM-β-CD, and these results are also shown in Figure 7. The exact energy values are listed in Tables S6 and S7 (SI). As can be seen, when the equivalent configurations in the B3LYP-GD2 and M062X-GD3 sets are compared, in most cases, the final orientation of the MIA with respect to DM-β-CD is very similar. Note, however, that during the M062X-GD3 optimization, the FΛ1 structure transformed into another CR1 configuration, having the complexation energy lower by about 4 kcal/mol than the one denoted as CR1. Both methods indicate that the formation of MIA:DM-β-CD in an aqueous solution is always energetically profitable, independently of the configuration. Since the narrow entrance to the DM-β-CD is blocked by methoxy groups, the inclusion complexes are formed only via the wider rim of the cone, and there are only three such configurations: CR1, NR1, and M1.

Figure 7 clearly shows that the *E*_compl_^BSSE^ and *G*_corr_compl_^BSSE^ values depend strongly on both the structure and the method used. The absolute B3LYP-GD2/6-31G(d,p) values are generally larger than M062X-GD3/6-31G(d,p), but the differences between them are not uniform. According to the B3LYP-GD2 results, the most stable configuration of the complex MIA:DM-β-CD is CR1 (*E*_compl_^BSSE^ = −30 kcal/mol), while in the M062X-GD3 set, it is NR1 (*E*_compl_^BSSE^ = −20.9 kcal/mol). In these configurations, one of the aromatic rings of MIA (CR or NR) is immersed inside the cyclodextrin cavity. Among the noninclusion complexes, the configuration FV1 deserves attention, as in both series, its *E*_compl_^BSSE^ is comparable to those for the inclusion forms. It may be related to the fact that the wider edge of the cyclodextrin cone is rich in negatively charged oxygen atoms, which allows for more efficient interaction with MIA than in other noninclusion complexes. Nevertheless, a more detailed analysis of the interactions between MIA and DM-β-CD is necessary to verify this supposition. The B3LYP-GD2/6-31G(d,p) Gibbs energies (Figure 7b) predict the spontaneous formation of all configurations, while the M062X-GD3/6-31G(d,p) values of *G*_corr_compl_^BSSE^ are negative for the inclusion complexes and only one noninclusion structure (M2). The latter results mainly from much smaller M062X-GD3 complexation energies, as for most structures, both methods yield similar entropy changes.

The effect of diffuse functions on the complexation energies *E* and *G* is different in the two sets. In the case of B3LYP-GD2, the SP 6-31++G(d,p) *E*_compl_^BSSE^ values are slightly less negative than those obtained with the smaller basis set, while in the M062X-GD3 series, an opposite relation occurs. As a result, the differences between the SP complexation energies obtained by the two DFT methods become smaller, but the trends in each series remain unchanged. Thus, the energetically and thermodynamically most stable MIA:DM-β-CD configuration in the SP B3LYP-GD2 series is still CR1 (*E*_compl_^BSSE^ = −29.6 kcal/mol), while in the SP M062X-GD3 series, it is NR1 (*E*_compl_^BSSE^ = −23.9 kcal/mol).

**Figure 8:**
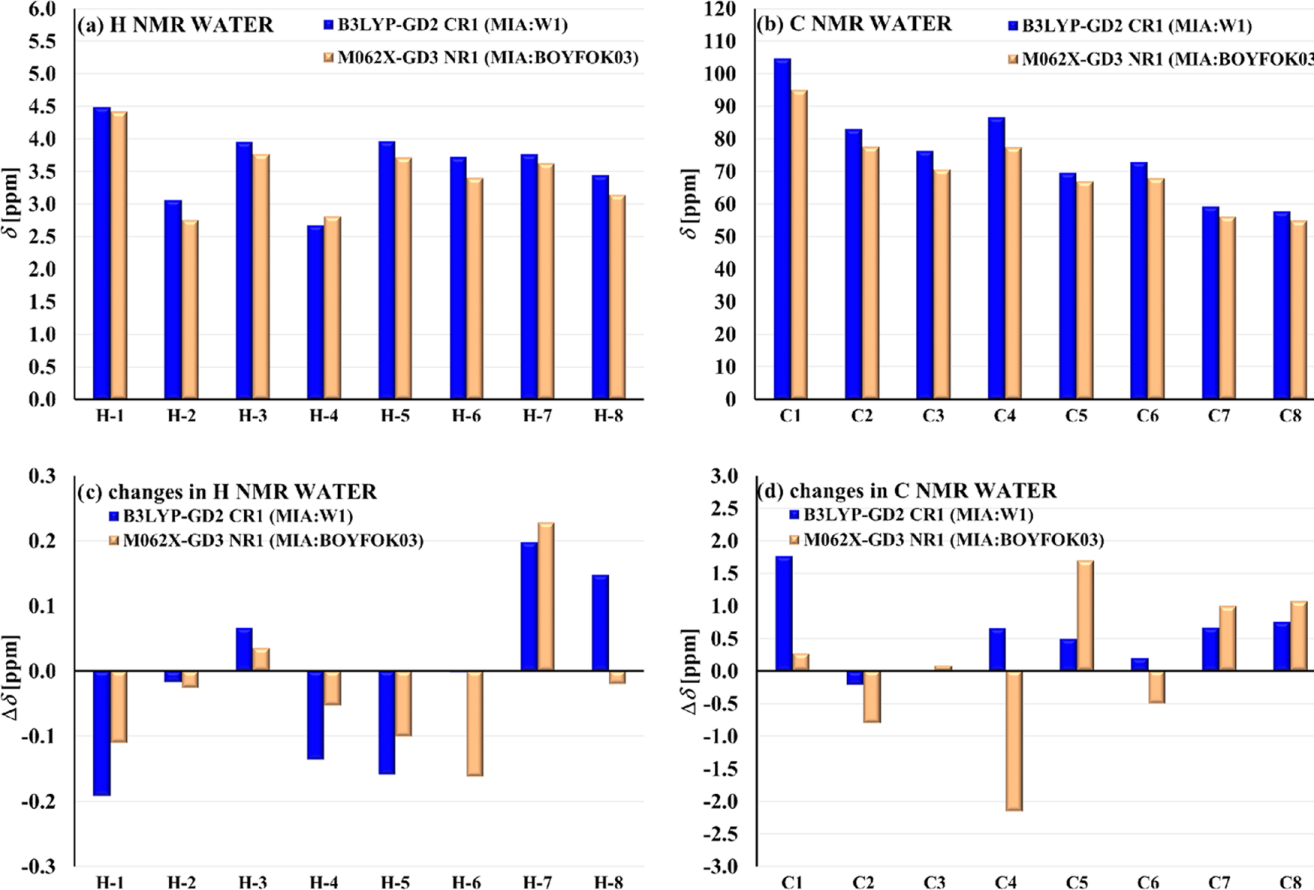
Comparison of the ^1^H and ^13^C NMR averaged chemical shifts δ (a, b) for DM-β-CD in the most stable configurations of MIA:DM-β-CD in water (PCM) indicated by the methods B3LYP-GD2 (CR1) and M062X-GD3 (NR1), and the changes Δδ of chemical shifts upon complexation (c, d), calculated with respect to W1 and optimized BOYFOK03, respectively.

For DM-β-CD in these two configurations, CR1 and NR1, the averaged chemical shifts δ and their changes Δ*δ* upon complexation are additionally analyzed. Despite the structural differences, their ^1^H and ^13^C NMR spectra are similar (Figure 8a,b). However, there are some differences in the changes Δδ (Figure 8c,d), calculated between the averaged δ values for DM-β-CD in the complex and the corresponding ones in the isolated most stable conformer. In the complex NR1 (MIA:BOYFOK03), the signals for the protons H-6 are upfield shifted (lower ppm values) much more than in CR1 (MIA:W1), while for the protons 6-CH_3_, quite a big downfield shift appears in CR1, while it is very small (upfield) in NR1. In both complexes, the largest change Δδ is found for the group of protons 2-CH_3_ (downfield shift). The detailed analysis shows that the main contributions to the averaged values come from the protons 2-CH_3_ belonging to the methyl groups located close to MIA and, at the same time, to the oxygen from the OH group. Nevertheless, in both configurations, the largest negative Δδ is obtained for one of the protons H-3, namely, atoms having the numbers 94 (CR1 B3LYP) and 123 (NR1 M062X) in Table S8 (SI). These hydrogens belong to the interior cavity of DM-β-CD, and in both cases, they appear to be involved in the interaction with the aromatic ring NR of MIA. Since for the six remaining protons H-3, the changes are smaller and, for four of them Δδ are positive, the averaged Δδ are relatively small. For carbons, the largest differences, either in values or/and in a sign, are seen for C1, C4, and C5, which is again an effect of averaging over different values coming from seven equivalent atoms [Table S8 (SI)].

Unfortunately, there is no experimental data for MIA:DM-β-CD, which could be used to verify the results presented above. However, some comparison can be made with the results obtained in the earlier study performed for the complex MIA:β-CD,^26^ where similar configurations were optimized with the B3LYP/6-31G(d,p) method, and for the lowest energy structures, the SP calculations with the B3LYP-GD2/6-31G(d,p) method were performed. In the latter case, the complexation energies were additionally corrected for the BSSE. For the complex formed by β-CD in its ground state geometry (denoted there as CCCW), the SP B3LYP-GD2 results indicated that the noninclusion flat configuration FΛ1 of MIA:β-CD is favorable, having the complexation energy and Gibbs energy of −23.5 (not published result) and −8.84 kcal/mol, respectively. These values are smaller than −30 and −17.9 kcal/mol obtained with the same method for CR1 of MIA:DM-β-CD, which suggests that the formation of the latter is more profitable. However, since for the systems containing cyclodextrins, the B3LYP-GD2 results appear to be less accurate than M062X-GD3, for a more reliable comparison, the structures of MIA:β-CD should be re-optimized with the latter functional.

It should also be mentioned that there are some additional aspects of the complexation phenomenon, which are not included in the present research. The complexation energies presented above were obtained with respect to the most stable structure of DM-β-CD, thus defining some reference level for comparisons. However, this molecule exhibits quite high flexibility, and other conformers might also contribute to the complex formation. In several works, the conformational flexibility of native cyclodextrins as well as of the inclusion complex 1:1 of β-CD with phenylalanine (zPHE) was analyzed using the simulation techniques.^67−69^ According to the results reported by Jana and Bandyopadhyay, upon complexation, the flexibility of both molecules, β-CD and zPHE, is significantly reduced, so their conformational fluctuations in the complex are much more limited than in the free molecules.^69^ Mianserin is much larger and more rigid than zPHE, so it can be expected that it will limit the flexibility of the complexes MIA:β-CD and MIA:DM-β-CD even more, but the verification of this assumption requires more detailed studies. Another factor influencing the complexation process is the solvation of reactants and their partial dehydration during the complex formation. In the present work, this phenomenon is only approximately accounted for by a continuum solvent model, but it would be advisable to analyze such effects in detail at the molecular level.

## Summary and Conclusions

In this work, an attempt was made to find and characterize the lowest energy structures of DM-β-CD and its complex with mianserin using a computational approach, in which the configuration space was explored by successively increasing the accuracy of the applied method up to the DFT level. In the case of the cyclodextrin alone, the theoretically found conformers were compared with five crystal structures. The dependence of the obtained results on various DFT methods as well as on the presence of diffuse functions in the basis set was also examined. The main conclusions can be summarized as follows:

(a) The results of the research carried out for the DM-β-CD molecule turned out to be strongly dependent on the DFT method used. The popularly used method B3LYP-GD2/6-31G(d,p) indicated as the most stable conformers those found from the theoretical search: V1 in vacuo and W1 in water and therefore assigned higher energy to all crystal structures (after their optimization). However, these results are not corroborated by the subsequent single-point calculations performed with several other density functionals, most of which yield the lowest energy for the two preoptimized crystal structures: ZULQAY in vacuo and BOYFOK03 in water. The re-optimization of selected structures with the M062X-GD3/6-31G(d,p) method, followed by the single-point calculations with the same functional and the 6-31++G(d,p) basis set, confirmed that ZULQAY and BOYFOK03 are the most stable conformers. On this basis, it is recommended to use these two DM-β-CD structures to build models of its complexes with other molecules in a given environment.
(b) According to the M062X-GD3 results obtained in water, there are at least several DM-β-CD structures whose relative energies, enthalpies, and Gibbs energies are in the range of several kcal/mol. This suggests that interconversion between different conformers is possible at a low energy cost, so they can coexist in the liquid phase. Some discrepancies observed between the NMR chemical shifts calculated for individual conformers in water and the corresponding experimental values seem to support this conclusion, although they may also be partly due to the simplified model of solvent (PCM) used in the calculations.
(c) The DFT results show that the low energy structures of a single molecule of DM-β-CD in water are less open than in vacuo. In the liquid phase, a narrow end of the cone is closed by a greater number of methoxy groups rotated inward; therefore, the formation of inclusion complexes with drugs can occur only through the wider entrance of the cyclodextrin.
(d) The choice of the DFT method and the initial structure of DM-β-CD used in the model of MIA:DM-β-CD has a large impact on the obtained configurations and complexation energies. The B3LYP-GD2 calculations carried out for the complex containing the conformer W1 of DM-β-CD predict the structure CR1 as the most stable (*E*_compl_^BSSE^ = −29.6 kcal/mol), while the more accurate M062X-GD3 results, obtained with the model based on the BOYFOK03 conformer, indicate the NR1 configuration (*E*_compl_^BSSE^ = −23.9 kcal/mol), followed by less stable FΛ1, which in fact is CR1, and M1 (−21.1 and −19.6 kcal/mol, respectively). All of these structures are typical inclusion complexes, in which some fragment of MIA—one of the aromatic rings (NR or CR) or the methyl group M—is immersed in the interior cavity of DM-β-CD via its wider entrance.
(e) In many works it is assumed that, since cyclodextrins form with other molecules, mainly inclusion complexes, noninclusion configurations are irrelevant and can be neglected. According to the results of the M062X-GD3 calculations, all eleven tested MIA:DM-β-CD configurations are stable in aqueous solution, although indeed the formation of inclusion complexes is energetically and thermodynamically more favorable. Nevertheless, as shown by the negative Gibbs energy values, some of the noninclusion complexes can also be formed spontaneously, which may have some influence on the final properties of the drug in the biological environment.

## Supporting Information Available

Detailed information about the computational procedure, DFT total, and relative energies of DM-β-CD conformers; comparison of selected DM-β-CD structures; NMR chemical shift values and their comparison; DFT total energies of MIA and MIA:DM-β-CD; and Cartesian coordinates of DM-β-CD and MIA:DM-β-CD (PDF)
