# Supplementary material for: In Search of the Most Stable Molecular Configuration of Heptakis(2,6-O-dimethyl)-β-cyclodextrin and Its Complex with Mianserin: A Comparison of the B3LYP-GD2 and M062X-GD3 Results
Source: J Phys Chem B. 2021 Nov 24;125(48):13077–87. doi: 10.1021/acs.jpcb.1c06831 (PMC8667041; doi:10.1021/acs.jpcb.1c06831)
Supplement: Supplementary file 1 — jp1c06831_si_001.pdf [file jp1c06831_si_001.pdf]

In Search of the Most Stable Molecular Configuration of Heptakis(2,6-*O*-dimethyl)-β-cyclodextrin and Its Complex with Mianserin: A Comparison of the B3LYP-GD2 and M062X-GD3 Results

Anna Ignaczak*, Łukasz Orszański

Theoretical and Structural Chemistry Group, Department of Physical Chemistry, Faculty of Chemistry, University of Lodz, Pomorska 163/165, 90-236 Lodz, Poland

* Email: anna.ignaczak@chemia.uni.lodz.pl

Electronic Supporting Information

Table of Contents:

Figure S1 – Flow chart of the procedure applied to find the low energy structures of DM-β-CD S3

Figure S2 – Initial geometry of DM-β-CD, three torsion angles varied in each glucose unit and atom numbering S4

Procedure – Detailed description of the conformational search performed for DM-β-CD S5

Figure S3 – Flow chart of the procedure applied to find the low energy structures of MIA:DM-β-CD S6

Figure S4 – Eleven studied configurations of the complex MIA:DM-β-CD S7

Table S1 – Total energies, enthalpies and Gibbs energies of various structures of DM-β-CD from the B3LYP-GD2 calculations S8

Table S2 – Total energies for selected DM-β-CD structures from the single point calculations performed with other DFT methods S9

Table S3 – Relative energies for selected DM-β-CD structures from the single point calculations with other DFT methods S10

Table S4 – Total energies, enthalpies and Gibbs energies of various DM-β-CD structures obtained from the M062X-GD3 calculations S11

Figure S5 – Comparison of selected conformers of DM-β-CD optimized with the methods B3LYP-GD2, M062X-GD3 and ωB97XD S12

Figure S6 – IR spectra obtained for different structures with the same method S13

Figure S7 – Comparison of DM-β-CD structures optimized with the 6-31G(d,p) and 6-31++G(d,p) basis sets....S14

Figure S8 – Comparison of the B3LYP-GD2 and M062X-GD3 relative energies and Gibbs energies for DM-β-CD structures S15

Table S5 – ^{1}H and ^{13}C NMR chemical shifts for individual atoms in the W1 (B3LYP) and BOYFOK03 (M062X) conformers of DM-β-CD S16

Figure S9 – ^{1}H and ^{13}C NMR chemical shifts for selected atoms in various conformers of DM-β-CD in water ..S21

Figure S10 – The linear regression between the calculated and experimental NMR chemical shifts S22

Table S6 – Total energies of MIA and MIA:DM-β-CD and complexation energies obtained from the B3LYP-GD2/6-31G(d,p) and M062X-GD3/6-31G(d,p) optimizations in water S23

Table S7 – Total energies of MIA and MIA:DM-β-CD and complexation energies obtained from the single point calculations with the 6-31++G(d,p) basis set S24

Table S8 – ^{1}H and ^{13}C NMR chemical shifts for individual atoms in the CR1 (B3LYP) and NR1 (M062X) configurations of MIA:DM-β-CD S25

Table S9 – Atom coordinates for the DM-β-CD conformers V1 and W1 - method B3LYP-GD2/6-31G(d,p).......S29

Table S10 – Atom coordinates for the DM-β-CD conformers ZULQAY and BOYFOK03 – method M062X-GD3/6-31G(d,p))..S33

Table S11 – Atom coordinates for the DM-β-CD conformers ZULQAY and BOYFOK03 - method M062X-GD3/6-31++G(d,p)..S37

Table S12 – Atom coordinates for the MIA:DM-β-CD complexes in water - method B3LYP-GD2/6-31G(d,p)) .S41

Table S13 – Atom coordinates for the MIA:DM-β-CD complexes in water – method M062X-GD3/6-31G(d,p) S58

References S75

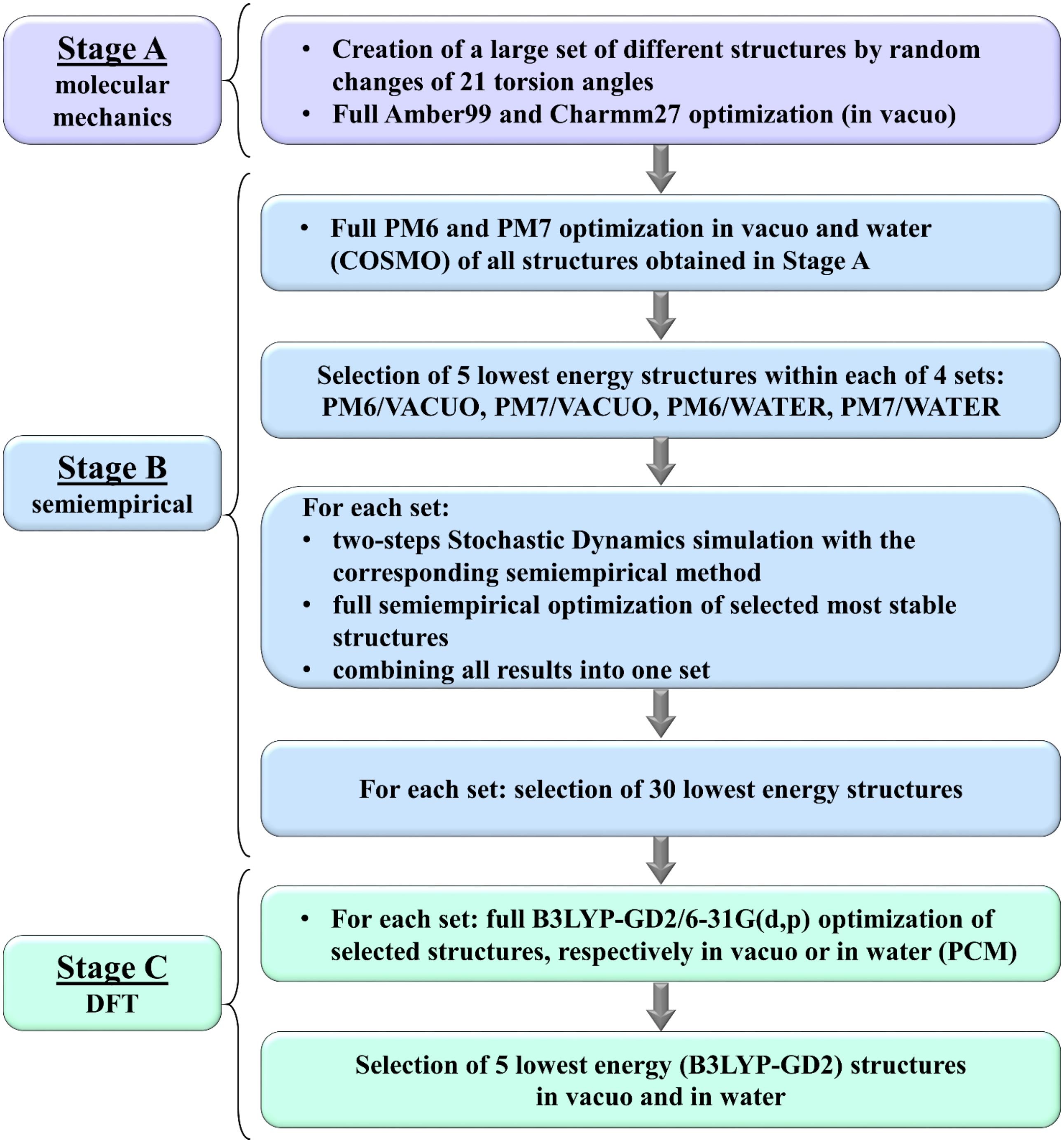

Figure S1. Flow chart of the procedure applied to find the lowest energy structures of the molecule DM-β-CD.

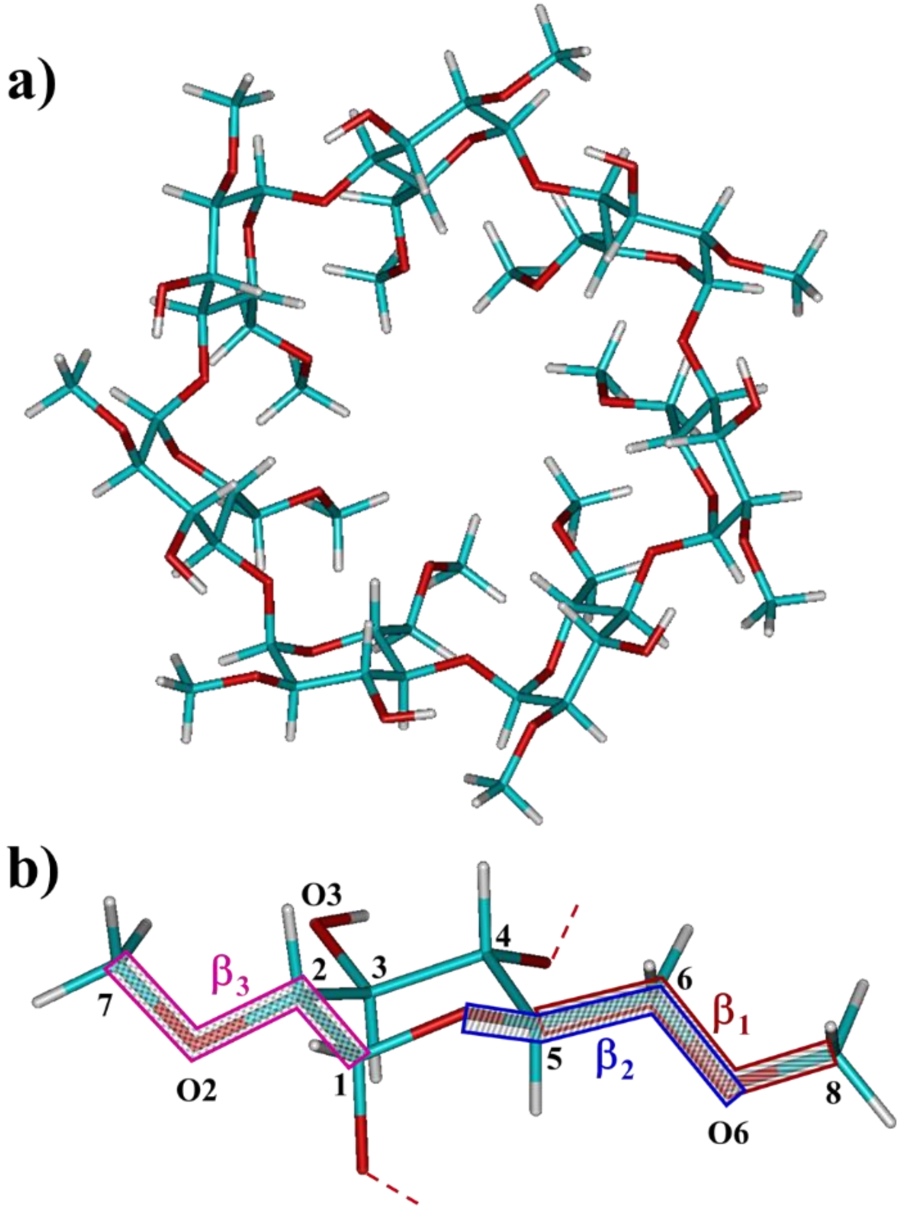

Figure S2. a) Geometry of the DM-β-CD molecule used as an initial structure for construction of various conformers (view from the wider side of the cone), b) three torsion angles modified in each glucose unit in the procedures of construction of various conformers and the atom numbering.

Procedure
Detailed description of the conformational search performed for DM-β-CD

Stage A: The initial structure was built in the Hyperchem program[1] and it had the regular geometry shown in Fig. S2a. In each glucose unit of this structure were selected three torsion angles (Fig. S2b), giving in total 21 torsion angles. The test conformers were produced using the molecular mechanics force fields Amber99 and Charmm27 and the Conformational Search module available in the program Hyperchem program. In the search each new conformer was created by random, simultaneous variation, in the range of 0-180°, of up to 3 torsion angles from the 21 torsion angles selected. The lowest energy conformers found with each force field were stored, giving in total 1722 structures which served as initial geometries in the next stage.

Stage B: All conformers were afterwards optimized in vacuo and in water using two different semiempirical methods: PM6 and PM7 available in the program MOPAC.[2] In the semiempirical calculations the solvent was described with the COSMO model,[3] using the values of 78.39 and 1.3 Å for the dielectric constant and the effective radius, respectively. From all geometries obtained were selected 10 structures corresponding to the lowest heats of formation in vacuo and 10 structures in water; both sets included 5 structures from the PM6 calculations and 5 structures from the PM7 calculations. For each conformer, further examination was performed using the Molecular Dynamics Conformational Search module available in the program Gabedit[4] in conjunction with the MOPAC program (PM6 and PM7 methods). The goal of this stage was to allow the molecule to adopt the geometry that was more stable in terms of energy. The simulations were performed using the Stochastic Dynamics via the Verlet algorithm. The conformational space was explored at T=1000K during the period of 20 ps with the time-step 1 fs. In each simulation, from the calculated trajectory 40 lowest energy conformations were selected and subsequently optimized at the corresponding semiempirical level. From all conformers collected within each set (PM6/VACUO, PM6/WATER, PM7/VACUO, PM7/WATER) were selected 3 most stable structures, for which the simulation was repeated and the lowest energy conformers were stored. The conformers selected in the simulations were merged with these obtained earlier with the same semiempirical method (PM6 or PM7, each in vacuo or in water), which led to creation of four sets of structures. Within each set were selected the 30 lowest energy conformers, which were used as initial geometries in the DFT calculations.

Stage C: All structures obtained in Stage B were fully optimized in the Gaussian09 program[5] using the 6-31G(d,p) basis set and the B3LYP-GD2 method.[6,7] For the 60 structures selected within the two sets PM6/VACUO and PM7/VACUO the DFT calculations were performed in vacuo, while for the 60 structures selected within the two sets PM6/WATER and PM7/WATER - in water. In the DFT calculations the solvent was described with the Polarizable Continuum Model (PCM).[8]

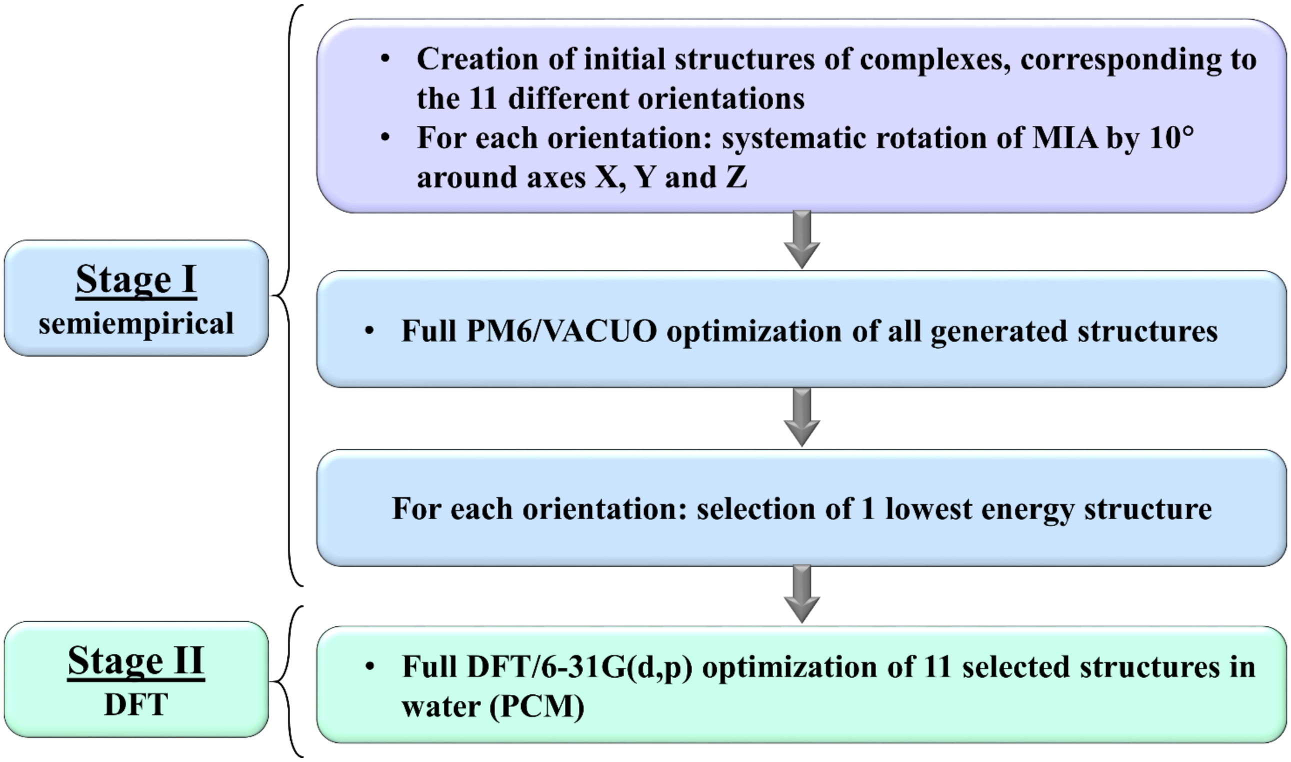

Figure S3. Flow chart of the procedure applied to find the lowest energy structures of the complex MIA:DM-β-CD.

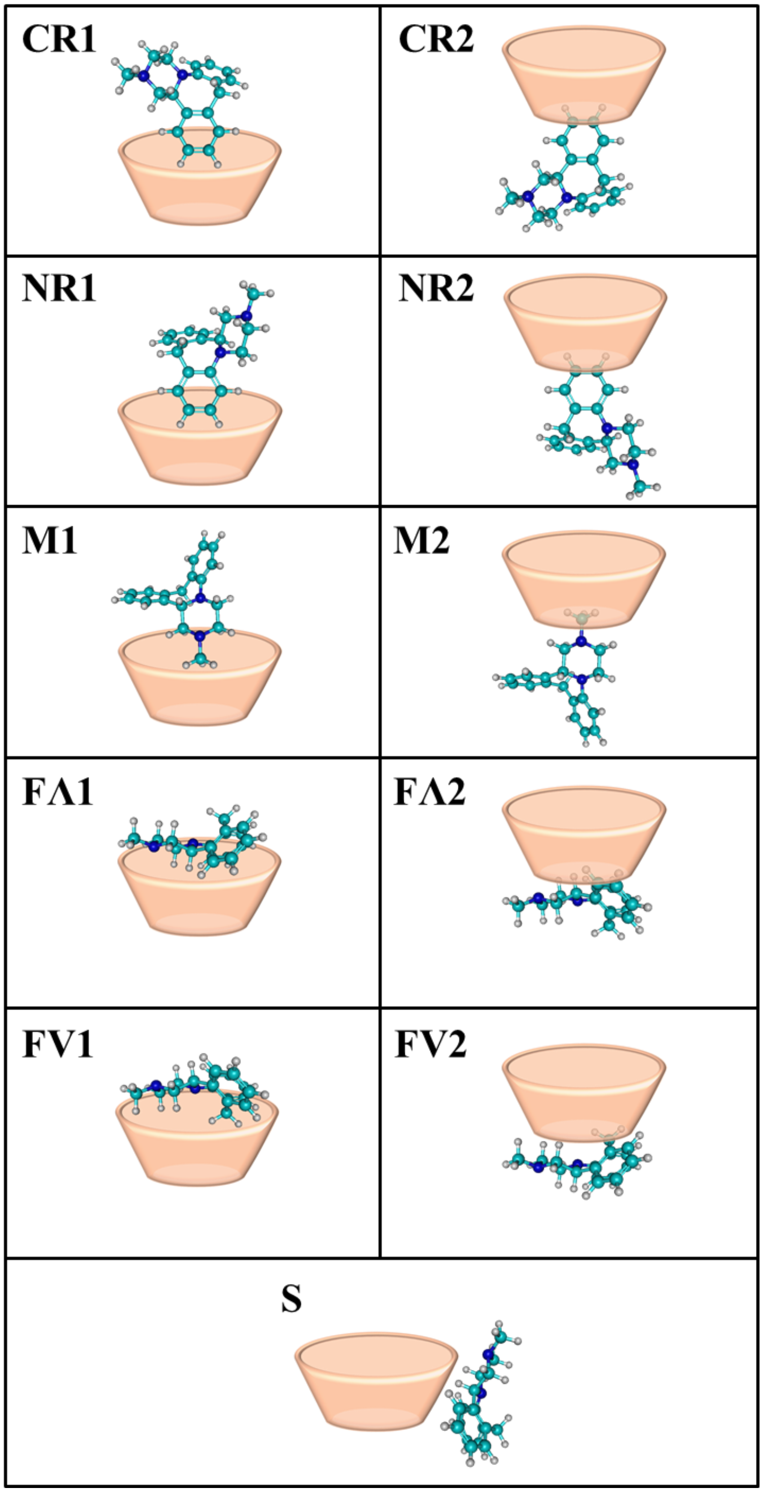

Figure S4. Eleven configurations of the complex MIA:DM-β-CD (1:1) considered in the calculations. CR, NR and M indicate the fragments of the MIA molecule (Chart 1b in the main article) inserted in the interior cavity of DM-β-CD, FΛ and FV - two different "flat" orientations, numbers 1, 2 and the label S – different sides of the cyclodextrin cone.

Table S1. The total electronic energies *E*, enthalpies *H* and corrected Gibbs energies G_{corr} obtained from the B3LYP-GD2/6-31G(d,p) calculations performed for the experimental structures of DM-β-CD (ZULQAY, CEQCUW, BOYFOK03, BOYFOK04, PABNEM; single point calculations for the original structures and after their optimization) and for the lowest energy conformers in vacuo (V1-V5) and in water (W1-W5), found from the conformational search. In the last column are given the electronic energies *E* obtained from the single point (SP) calculations performed with the 6-31++G(d,p) basis set for the optimized structures. All values are in hartree.

Structure	Single point *E*	Optimized *E*	Optimized *H*	Optimized *G*	Optimized G_{corr}	SP 6-31++G(d,p) *E*
**in VACUO**						
**ZULQAY**	-4825.617085	-4826.001307	-4824.292511	-4824.525224	-4824.503101	-4826.170912
**CEQCUW**	-4824.340915	-4825.990171	-4824.281332	-4824.512566	-4824.491477	-4826.160026
**BOYFOK03**	-4825.678624	-4825.999707	-4824.290515	-4824.520273	-4824.499660	-4826.167843
**BOYFOK04**	-4824.318150	-4825.996993	-4824.287142	-4824.513869	-4824.494785	-4826.165639
**PABNEM**	-4824.661886	-4826.002305	-4824.293503	-4824.524125	-4824.503220	-4826.171156
**V1**		-4826.005732	-4824.295165	-4824.518858	-4824.500920	-4826.171643
**V2**		-4826.005230	-4824.294261	-4824.519012	-4824.500011	-4826.168882
**V3**		-4826.003686	-4824.293172	-4824.519233	-4824.499681	-4826.167411
**V4**		-4826.003688	-4824.292020	-4824.512959	-4824.496391	-4826.161819
**V5**		-4826.003502	-4824.292090	-4824.517113	-4824.497719	-4826.163592
**in WATER (PCM)**						
**ZULQAY**	-4825.665558	-4826.042086	-4824.335457	-4824.568213	-4824.534616	-4826.221417
**CEQCUW**	-4824.406142	-4826.033616	-4824.327440	-4824.561236	-4824.527433	-4826.214908
**BOYFOK03**	-4825.737045	-4826.045585	-4824.338372	-4824.570357	-4824.537027	-4826.224433
**BOYFOK04**	-4824.385492	-4826.041185	-4824.333842	-4824.562841	-4824.531282	-4826.220130
**PABNEM**	-4824.715739	-4826.040870	-4824.334607	-4824.567460	-4824.533894	-4826.219507
**W1**		-4826.050254	-4824.341034	-4824.563364	-4824.534257	-4826.227305
**W2**		-4826.049687	-4824.340968	-4824.565247	-4824.535201	-4826.226859
**W3**		-4826.048893	-4824.340023	-4824.564005	-4824.534213	-4826.224549
**W4**		-4826.047957	-4824.338297	-4824.559989	-4824.530895	-4826.223671
**W5**		-4826.046416	-4824.336733	-4824.558734	-4824.529630	-4826.223056

Table S2. The total electronic energies *E* obtained from the single point calculations performed with several other DFT methods and the 6-31G(d,p) basis set for the DM-β-CD structures optimized with the B3LYP-GD2/6-31G(d,p) method. All values are in hartree.

Structure	M05-GD3	M06-GD3	M062X-GD3	ωB97XD	mPW1PW91	M11
			**in VACUO**			
**ZULQAY**	-4823.053737	-4823.023173	-4823.793952	-4824.317152	-4824.481434	-4823.471934
**CEQCUW**	-4823.042455	-4823.012967	-4823.781011	-4824.304906	-4824.464448	-4823.455722
**BOYFOK03**	-4823.049564	-4823.025387	-4823.790332	-4824.315399	-4824.469298	-4823.465195
**BOYFOK04**	-4823.045560	-4823.018277	-4823.784139	-4824.309597	-4824.462025	-4823.458134
**PABNEM**	-4823.054008	-4823.022882	-4823.793160	-4824.317011	-4824.480515	-4823.471866
**V1**	-4823.050061	-4823.020972	-4823.788802	-4824.315282	-4824.464694	-4823.463261
**V2**	-4823.046317	-4823.019080	-4823.788886	-4824.312591	-4824.460037	-4823.466832
**V3**	-4823.045752	-4823.017509	-4823.789023	-4824.312293	-4824.462066	-4823.466990
**V4**	-4823.036804	-4823.024979	-4823.782714	-4824.309393	-4824.421864	-4823.455157
**V5**	-4823.043211	-4823.017440	-4823.784153	-4824.310307	-4824.452413	-4823.460063
			**in WATER (PCM)**			
**ZULQAY**	-4823.093929	-4823.063286	-4823.835604	-4824.359910	-4824.522926	-4823.516462
**CEQCUW**	-4823.087391	-4823.055930	-4823.827084	-4824.351570	-4824.515744	-4823.505944
**BOYFOK03**	-4823.096258	-4823.070885	-4823.838428	-4824.363802	-4824.519056	-4823.517566
**BOYFOK04**	-4823.091122	-4823.064439	-4823.830848	-4824.357325	-4824.509589	-4823.508227
**PABNEM**	-4823.093177	-4823.061116	-4823.833363	-4824.357984	-4824.520907	-4823.514940
**W1**	-4823.093172	-4823.068445	-4823.834813	-4824.362721	-4824.508782	-4823.510844
**W2**	-4823.093424	-4823.067069	-4823.834212	-4824.362162	-4824.509683	-4823.510452
**W3**	-4823.093320	-4823.067626	-4823.833037	-4824.361121	-4824.507820	-4823.508814
**W4**	-4823.088333	-4823.065721	-4823.832024	-4824.359293	-4824.502099	-4823.508596
**W5**	-4823.087409	-4823.061778	-4823.827021	-4824.357252	-4824.503056	-4823.501273

Table S3. The relative energies Δ*E* [kcal/mol] obtained from the single point (SP) calculations performed using several other DFT methods and the 6-31G(d,p) basis set, for the DM-β-CD structures optimized with the B3LYP-GD2 method. The Δ*E* values are always calculated with respect to the lowest energy conformer indicated by a given method.

Structure	M05-GD3	M06-GD3	M062X-GD3	ωB97XD	mPW1PW91	M11
			**in VACUO**			
**ZULQAY**	0.17	1.39	0.00	0.00	0.00	0.00
**CEQCUW**	7.25	7.79	8.12	7.68	10.66	10.17
**BOYFOK03**	2.79	0.00	2.27	1.10	7.62	4.23
**BOYFOK04**	5.30	4.46	6.16	4.74	12.18	8.66
**PABNEM**	0.00	1.57	0.50	0.09	0.58	0.04
**V1**	2.48	2.77	3.23	1.17	10.50	5.44
**V2**	4.83	3.96	3.18	2.86	13.43	3.20
**V3**	5.18	4.94	3.09	3.05	12.15	3.10
**V4**	10.80	0.26	7.05	4.87	37.38	10.53
**V5**	6.78	4.99	6.15	4.30	18.21	7.45
			**in WATER (PCM)**			
**ZULQAY**	1.46	4.77	1.77	2.44	0.00	0.69
**CEQCUW**	5.56	9.38	7.12	7.68	4.51	7.29
**BOYFOK03**	0.00	0.00	0.00	0.00	2.43	0.00
**BOYFOK04**	3.22	4.05	4.76	4.06	8.37	5.86
**PABNEM**	1.93	6.13	3.18	3.65	1.27	1.65
**W1**	1.94	1.53	2.27	0.68	8.88	4.22
**W2**	1.78	2.39	2.65	1.03	8.31	4.46
**W3**	1.84	2.04	3.38	1.68	9.48	5.49
**W4**	4.97	3.24	4.02	2.83	13.07	5.63
**W5**	5.55	5.71	7.16	4.11	12.47	10.22

Table S4. The total electronic energies E, enthalpies H and corrected Gibbs energies G_{corr} of DM-β-CD obtained with the M062X-GD3/6-31G(d,p) method: from the single point calculations performed for the original experimental structures and from the M062X-GD3 re-optimization of the structures optimized with the B3LYP-GD2. In the last column are given the electronic energies E obtained from the single point (SP) calculations performed with the 6-31++G(d,p) basis set for the optimized structures. All values are in hartree.

Structure	Single point *E*	Optimized *E*	Optimized *H*	Optimized *G*	Optimized G_{corr}	SP 6-31++G(d,p) *E*
**in VACUO**						
**ZULQAY**	-4823.389601	-4823.800060	-4822.070092	-4822.302710	-4822.280333	-4823.942002
**CEQCUW**	-4822.132292	-4823.793097	-4822.063048	-4822.291878	-4822.271529	-4823.935096
**BOYFOK03**	-4823.465779	-4823.796939	-4822.066878	-4822.294971	-4822.274881	-4823.938049
**BOYFOK04**	-4822.109973	-4823.789994	-4822.059647	-4822.287794	-4822.267637	-4823.932270
**PABNEM**	-4822.444621	-4823.799771	-4822.069790	-4822.300436	-4822.279111	-4823.941596
**V1**		-4823.794917	-4822.064295	-4822.289657	-4822.270578	-4823.935458
**V2**		-4823.795359	-4822.064328	-4822.289229	-4822.269913	-4823.933750
**V3**		-4823.794921	-4822.063866	-4822.288782	-4822.269502	-4823.933153
**V4**		-4823.789650	-4822.059227	-4822.279908	-4822.263412	-4823.927277
**V5**		-4823.791684	-4822.060418	-4822.285233	-4822.265604	-4823.929223
**in WATER (PCM)**						
**ZULQAY**	-4823.441988	-4823.842104	-4822.114703	-4822.346383	-4822.313262	-4823.991933
**CEQCUW**	-4822.201628	-4823.833921	-4822.106670	-4822.338753	-4822.305780	-4823.985570
**BOYFOK03**	-4823.528403	-4823.844581	-4822.116697	-4822.346140	-4822.313896	-4823.993905
**BOYFOK04**	-4822.181288	-4823.838670	-4822.111056	-4822.338606	-4822.307485	-4823.988344
**PABNEM**	-4822.502021	-4823.840110	-4822.113025	-4822.345536	-4822.312037	-4823.990291
**W1**		-4823.840641	-4822.112514	-4822.338050	-4822.307429	-4823.990322
**W2**		-4823.840239	-4822.112232	-4822.338670	-4822.307607	-4823.990100
**W3**		-4823.839681	-4822.111671	-4822.336153	-4822.305937	-4823.988730
**W4**		-4823.838028	-4822.109390	-4822.331511	-4822.302265	-4823.986724
**W5**		-4823.833373	-4822.105102	-4822.329521	-4822.299215	-4823.983184

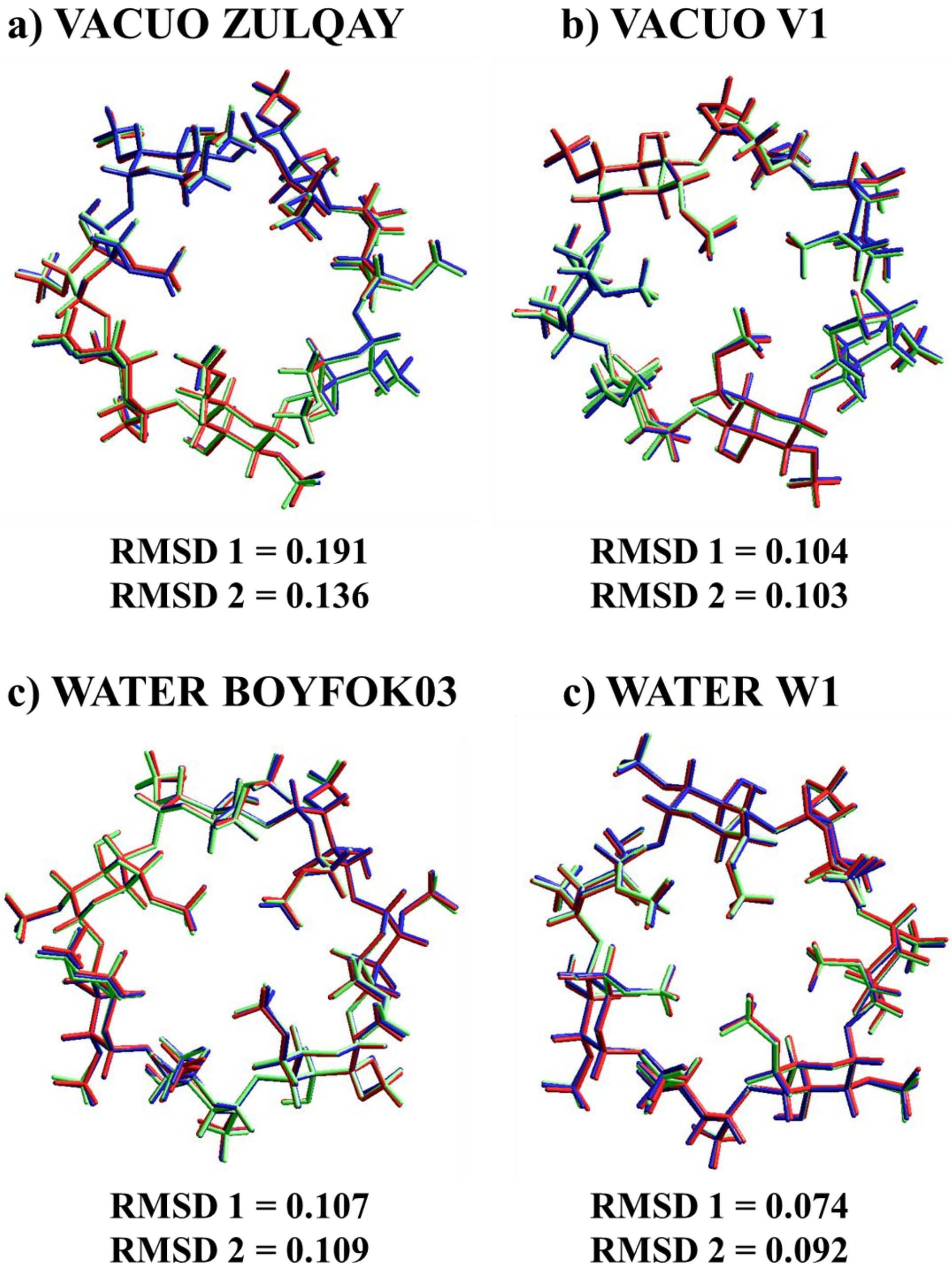

Figure S5. Comparison of the DM-β-CD structures indicated by various methods as the most stable conformers, obtained from the B3LYP-GD2/6-31G(d,p) (dark blue), M062X-GD3/6-31G(d,p) (red) and ωB97XD/6-31G(d,p) (green) optimizations in vacuo (a,b) and in water (c,d). For each case, the root-mean square deviations of atomic positions (in Å) for the structures optimized with the methods M062X-GD3 (RMSD 1) and ωB97XD (RMSD 2), calculated always with respect to the structures optimized with the B3LYP-GD2 method, are also given.

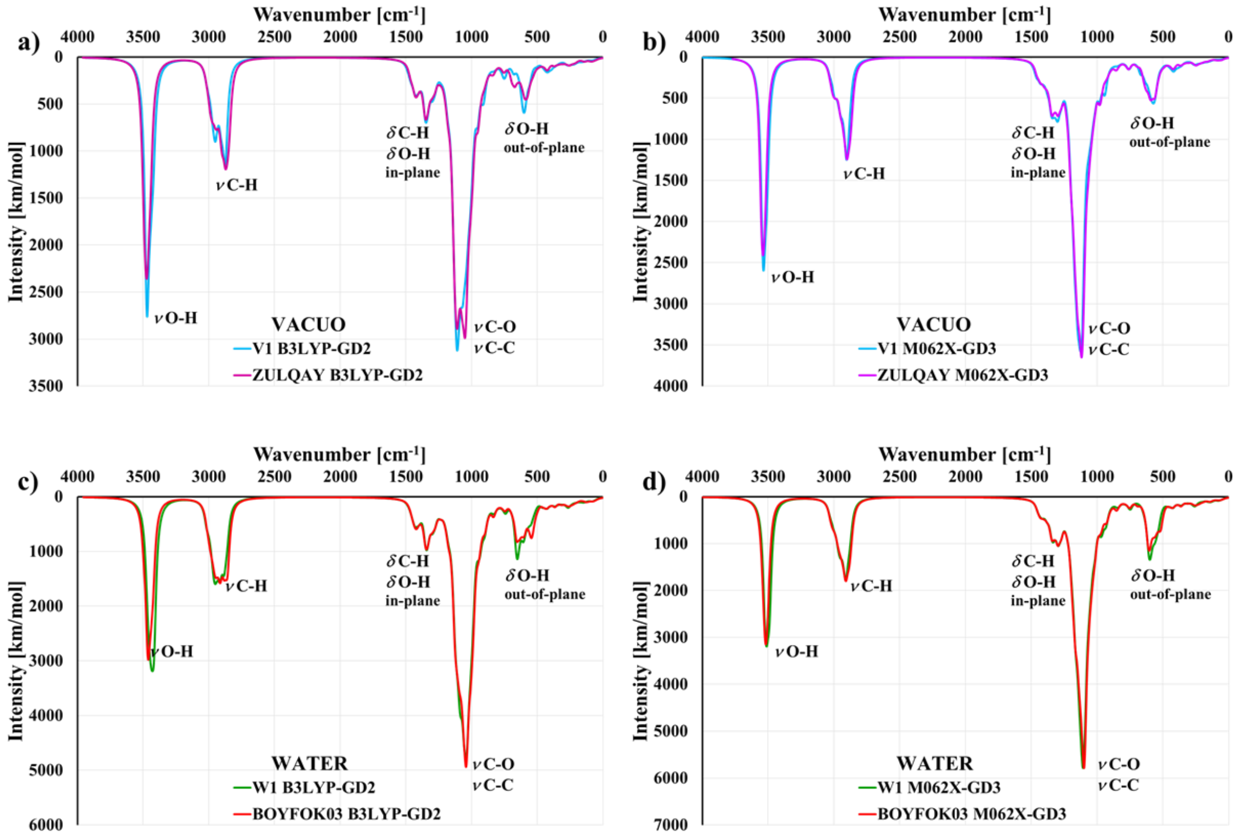

Figure S6. Comparison of IR spectra obtained from the B3LYP-GD2/6-31G(d,p) (a,c) and M062X-GD3/6-31G(d,p) (b,d) calculations for the structures of V1 and ZULQAY in vacuo (a,b) and for the structures of W1 and BOYFOK03 in water (c,d) optimized with the same DFT methods.

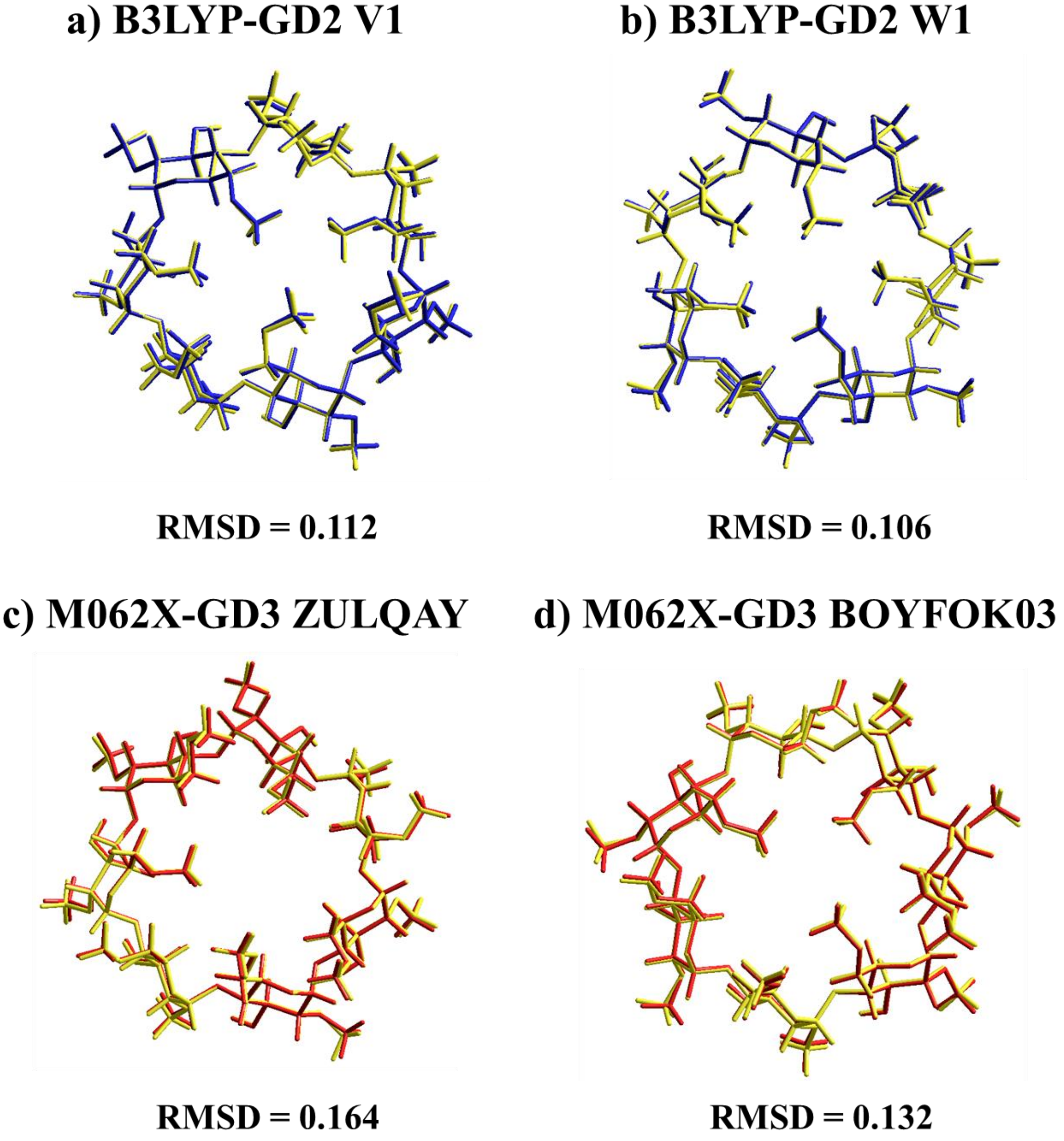

Figure S7. Comparison of the DM-β-CD structures obtained from the B3LYP-GD2/6-31G(d,p) and M062X-GD3/6-31G(d,p) optimizations (blue and red, respectively) performed in vacuo (a,c) and in water (b,d) with the structures obtained after their re-optimization with the same DFT methods and the 6-31++G(d,p) basis set (yellow in all cases). For each case, the root-mean square deviation (RMSD) of atomic positions (in Å) is also given.

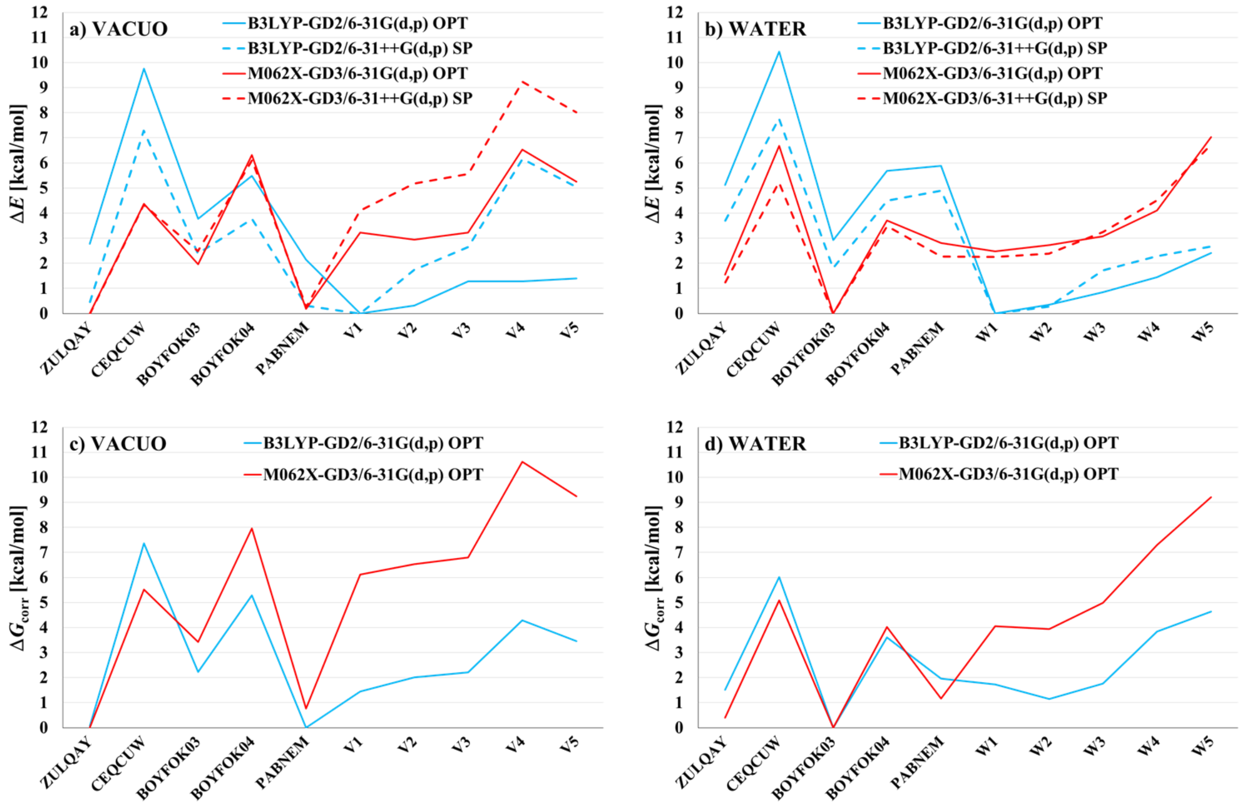

Figure S8. Comparison of the relative energies ΔE (a,b) and corrected Gibbs energies ΔG_{corr} (c,d) calculated with respect to the lowest energy conformer, obtained for various structures of DM-β-CD optimized (OPT) with the B3LYP-GD2/6-31G(d,p) and M062X-GD3/6-31G(d,p) methods in vacuo and water (PCM). The corresponding energy values obtained from the single point calculations (SP) performed with the 6-31++G(d,p) basis set are included in the plots (a) and (b).

Table S5. The computed ^{1}H and ^{13}C NMR chemical shifts (δ_{calc}) [ppm] for individual atoms in the most stable conformers of DM-β-CD in water indicated by the methods B3LYP-GD2 (W1) and M062X-GD3 (BOYFOK03) obtained from the B3LYP/6-31++G(d,p)//B3LYP-GD2/6-31G(d,p) and M062X/6-31++G(d,p)//M062X-GD3/6-31G(d,p) calculations, respectively. The δ_{calc} are scaled according to the procedure of Tantillo,[9] using the formula δ=(I−σ)/(−S), where σ are the isotropic values obtained from DFT calculations, and I and S are the scaling factors obtained with a given method. For the B3LYP method their values in water (PCM) are: S_H = -1.0567, I_H = 31.6557, S_C = -0.9687, I_C = 189.8139;[10] the scaling factors for the M062X method are given in the main article.

a) Protons						
Atom no. in Fig. S2b		W1 B3LYP		BOYFOK03 M062X		
	Atom no. in Table S9	Isotropic	δ_{calc}	Atom no. in Table S10	Isotropic	δ_{calc}
H-1	11	26.6442	4.74	14	27.0612	4.28
H-1	33	26.8390	4.56	40	26.8244	4.49
H-1	54	26.7572	4.64	67	26.7271	4.57
H-1	75	26.6604	4.73	94	26.8800	4.44
H-1	96	26.8174	4.58	121	26.9003	4.42
H-1	117	26.5970	4.79	148	26.7061	4.59
H-1	138	26.7238	4.67	175	26.3486	4.90
H-2	10	28.4516	3.03	15	28.8713	2.72
H-2	32	28.3954	3.09	41	28.8051	2.78
H-2	53	28.4054	3.08	68	28.8884	2.71
H-2	74	28.4285	3.05	95	28.8773	2.72
H-2	95	28.4335	3.05	122	28.7831	2.80
H-2	116	28.3965	3.08	149	28.7311	2.84
H-2	137	28.3219	3.16	176	28.7328	2.84
H-3	9	27.6744	3.77	16	27.5907	3.83
H-3	31	27.4352	3.99	42	27.5547	3.86
H-3	52	27.4861	3.95	69	27.8377	3.61
H-3	73	27.6908	3.75	96	27.7295	3.71
H-3	94	27.4526	3.98	123	27.5883	3.83
H-3	115	27.7304	3.71	150	27.7929	3.65
H-3	136	27.4200	4.01	177	27.8530	3.60
H-4	8	28.8044	2.70	17	28.5038	3.04
H-4	30	28.5785	2.91	43	29.0932	2.53
H-4	51	28.7199	2.78	70	28.2756	3.24
H-4	72	28.7877	2.71	97	29.1889	2.45

H-4	93	28.6106	2.88	124	28.7436	2.83
H-4	114	28.7466	2.75	151	28.2311	3.28
H-4	135	28.6072	2.89	178	28.9522	2.65
H-5	7	27.2487	4.17	18	27.6154	3.81
H-5	29	27.3210	4.10	44	27.3844	4.00
H-5	50	27.3976	4.03	71	27.6777	3.75
H-5	71	27.2157	4.20	98	27.4801	3.92
H-5	92	27.4097	4.02	125	27.7414	3.70
H-5	113	27.1789	4.24	152	27.8308	3.62
H-5	134	27.3485	4.08	179	27.5541	3.86
H-6	17	27.4861	3.95	19	27.6899	3.74
H-6	19	28.0151	3.45	45	27.9517	3.52
H-6	38	27.4016	4.03	46	27.8292	3.62
H-6	40	27.8877	3.57	72	27.4497	3.95
H-6	59	26.9697	4.43	73	28.2577	3.25
H-6	61	28.5071	2.98	99	28.2374	3.27
H-6	80	27.3136	4.11	100	27.5055	3.90
H-6	82	28.0528	3.41	126	28.3169	3.20
H-6	101	27.5298	3.90	127	27.5424	3.87
H-6	103	27.9966	3.46	153	27.4665	3.93
H-6	122	27.5333	3.90	154	28.1069	3.38
H-6	124	28.0943	3.37	180	27.9108	3.55
H-6	142	27.2332	4.19	181	28.0632	3.42
H-6	144	28.1757	3.29	189	28.3100	3.21
H-7	169	27.9701	3.49	20	28.0893	3.40
H-7	170	27.9518	3.51	21	28.3444	3.18
H-7	171	28.3334	3.14	22	27.4798	3.92
H-7	172	27.8792	3.57	47	28.1043	3.38
H-7	173	28.1356	3.33	48	28.3973	3.13
H-7	174	27.2323	4.19	49	27.4288	3.97
H-7	175	27.8932	3.56	74	28.1006	3.39
H-7	176	28.1336	3.33	75	28.2817	3.23
H-7	177	27.2250	4.19	76	28.6132	2.95
H-7	178	27.9306	3.53	101	28.0571	3.43
H-7	179	28.0267	3.43	102	28.4072	3.12
H-7	180	28.3324	3.15	103	27.3654	4.02
H-7	181	27.9124	3.54	128	28.0646	3.42
H-7	182	28.1367	3.33	129	28.3468	3.18
H-7	183	27.3214	4.10	130	27.4935	3.91
H-7	184	27.9533	3.50	155	27.9868	3.49
H-7	185	27.9811	3.48	156	28.2157	3.29
H-7	186	28.3437	3.13	157	28.5527	3.00
H-7	187	27.8609	3.59	182	28.0777	3.41

H-7	188	28.0969	3.37	183	28.0065	3.47
H-7	189	27.2010	4.22	184	28.5780	2.98
H-8	148	28.7861	2.72	23	28.2387	3.27
H-8	149	28.4630	3.02	24	28.6144	2.95
H-8	150	27.8671	3.59	25	27.9950	3.48
H-8	151	28.0947	3.37	50	28.3451	3.18
H-8	152	28.1653	3.30	51	28.3248	3.19
H-8	153	28.0808	3.38	52	28.9057	2.69
H-8	154	27.9711	3.49	77	28.2688	3.24
H-8	155	27.9675	3.49	78	28.5689	2.98
H-8	156	28.3967	3.08	79	27.9647	3.50
H-8	157	28.7363	2.76	104	28.5608	2.99
H-8	158	28.3016	3.17	105	28.4161	3.12
H-8	159	27.6400	3.80	106	28.1699	3.33
H-8	160	28.1468	3.32	131	27.9514	3.52
H-8	161	28.0057	3.45	132	28.3920	3.14
H-8	162	27.8926	3.56	133	28.4879	3.05
H-8	163	28.1957	3.27	158	28.2356	3.27
H-8	164	28.2565	3.22	159	28.6672	2.90
H-8	165	28.5114	2.98	160	28.0379	3.44
H-8	166	27.9270	3.53	185	28.3568	3.17
H-8	167	28.0646	3.40	186	28.1888	3.31
H-8	168	28.2456	3.23	187	28.9416	2.66
HO(-3)	20	26.0685	5.29	26	26.8516	4.46
HO(-3)	41	26.2463	5.12	53	26.6245	4.66
HO(-3)	62	26.2547	5.11	80	26.9678	4.36
HO(-3)	83	26.0041	5.35	107	26.6315	4.65
HO(-3)	104	26.3292	5.04	134	26.5776	4.70
HO(-3)	125	26.1043	5.25	161	27.0497	4.29
HO(-3)	145	26.4584	4.92	188	26.8093	4.50

b) Carbons

Atom no. in Fig. S2b	W1 B3LYP			BOYFOK03 M062X		
	Atom no. in Table S9	Isotropic	δ_{calc}	Atom no. in Table S10	Isotropic	δ_{calc}
C1	6	92.0130	100.96	1	91.7892	96.45
C1	28	88.0065	105.10	27	92.2471	96.04
C1	49	88.8890	104.19	54	95.3537	93.22
C1	70	92.7995	100.15	81	92.2851	96.00
C1	91	88.7145	104.37	108	92.4030	95.89
C1	112	92.4572	100.50	135	95.2146	93.35
C1	133	88.3899	104.70	162	95.7332	92.88
C2	5	109.8332	82.56	2	112.1627	77.99

C2	27	109.3001	83.12	28	112.5141	77.67
C2	48	109.0451	83.38	55	110.0246	79.93
C2	69	108.9672	83.46	82	112.4567	77.72
C2	90	109.3849	83.03	109	113.0464	77.19
C2	111	108.3135	84.13	136	111.0327	79.01
C2	132	109.8661	82.53	163	111.5351	78.56
C3	4	117.6052	74.54	3	118.7960	71.98
C3	26	114.8693	77.37	29	118.8886	71.89
C3	47	114.0432	78.22	56	122.6384	68.50
C3	68	117.6814	74.46	83	117.7428	72.93
C3	89	115.3023	76.92	110	120.7421	70.22
C3	110	117.3606	74.79	137	121.9128	69.15
C3	131	114.5316	77.71	164	122.0207	69.06
C4	3	106.0299	86.49	4	111.5714	78.52
C4	25	107.7663	84.70	30	109.1734	80.70
C4	46	105.1837	87.36	57	111.3047	78.77
C4	67	105.9884	86.53	84	109.3543	80.53
C4	88	108.3398	84.11	111	109.6496	80.27
C4	109	105.5351	87.00	138	112.2882	77.88
C4	130	106.7117	85.79	165	109.1794	80.69
C5	2	123.5467	68.41	5	125.3299	66.06
C5	24	124.0695	67.87	31	129.5882	62.20
C5	45	120.3446	71.71	58	123.2576	67.94
C5	66	124.2874	67.64	85	129.6554	62.14
C5	87	122.9859	68.99	112	124.4275	66.88
C5	108	125.6813	66.20	139	122.2388	68.86
C5	129	119.4999	72.59	166	128.7686	62.94
C6	12	118.6915	73.42	6	125.8594	65.58
C6	34	119.0039	73.10	32	121.2703	69.74
C6	55	118.7226	73.39	59	123.1964	67.99
C6	76	119.2035	72.89	86	122.2980	68.81
C6	97	119.7987	72.28	113	122.0986	68.99
C6	118	119.8514	72.22	140	123.7253	67.51
C6	139	120.0168	72.05	167	120.2990	70.62
C7	21	134.3749	57.23	7	136.0686	56.33
C7	42	132.1479	59.53	33	136.2118	56.20
C7	63	132.0807	59.60	60	138.4825	54.14
C7	84	134.4403	57.16	87	136.7289	55.73
C7	105	131.7756	59.91	114	135.9982	56.39
C7	126	134.1818	57.43	141	139.0413	53.63
C7	146	132.3105	59.36	168	139.1860	53.50
C8	22	135.2010	56.38	8	138.3543	54.26
C8	43	134.4688	57.13	34	138.7343	53.91

C8	64	134.5175	57.08	61	138.3371	54.27
C8	85	135.8689	55.69	88	141.2081	51.67
C8	106	132.7974	58.86	115	138.5428	54.09
C8	127	135.0622	56.52	142	138.0922	54.49
C8	147	133.9329	57.69	169	138.8591	53.80

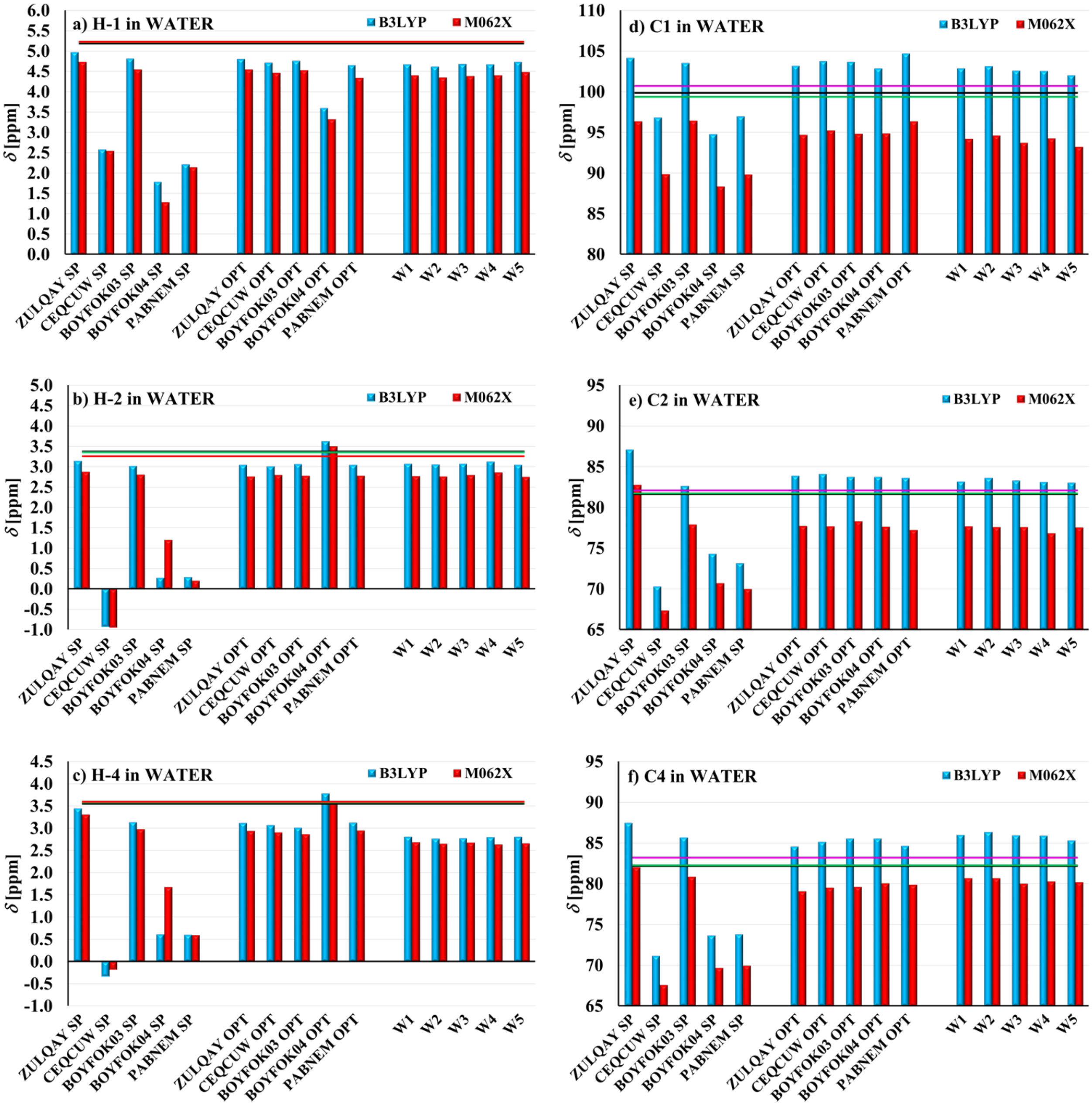

Figure S9. The ^{1}H (a-c) and ^{13}C NMR chemical shifts δ (scaled) for selected protons and carbons in various structures of DM-β-CD computed in water (PCM), compared to the experimental values measured in D_2O (horizontal lines).[11-14] The computed values were obtained from the calculations performed in water (PCM) at the two theory levels: B3LYP/6-31++G(d,p)//B3LYP-GD2/6-31G(d,p) (blue vertical bars) and M062X-GD3/6-31++G(d,p)//M062X-GD3/6-31G(d,p) (red vertical bars).

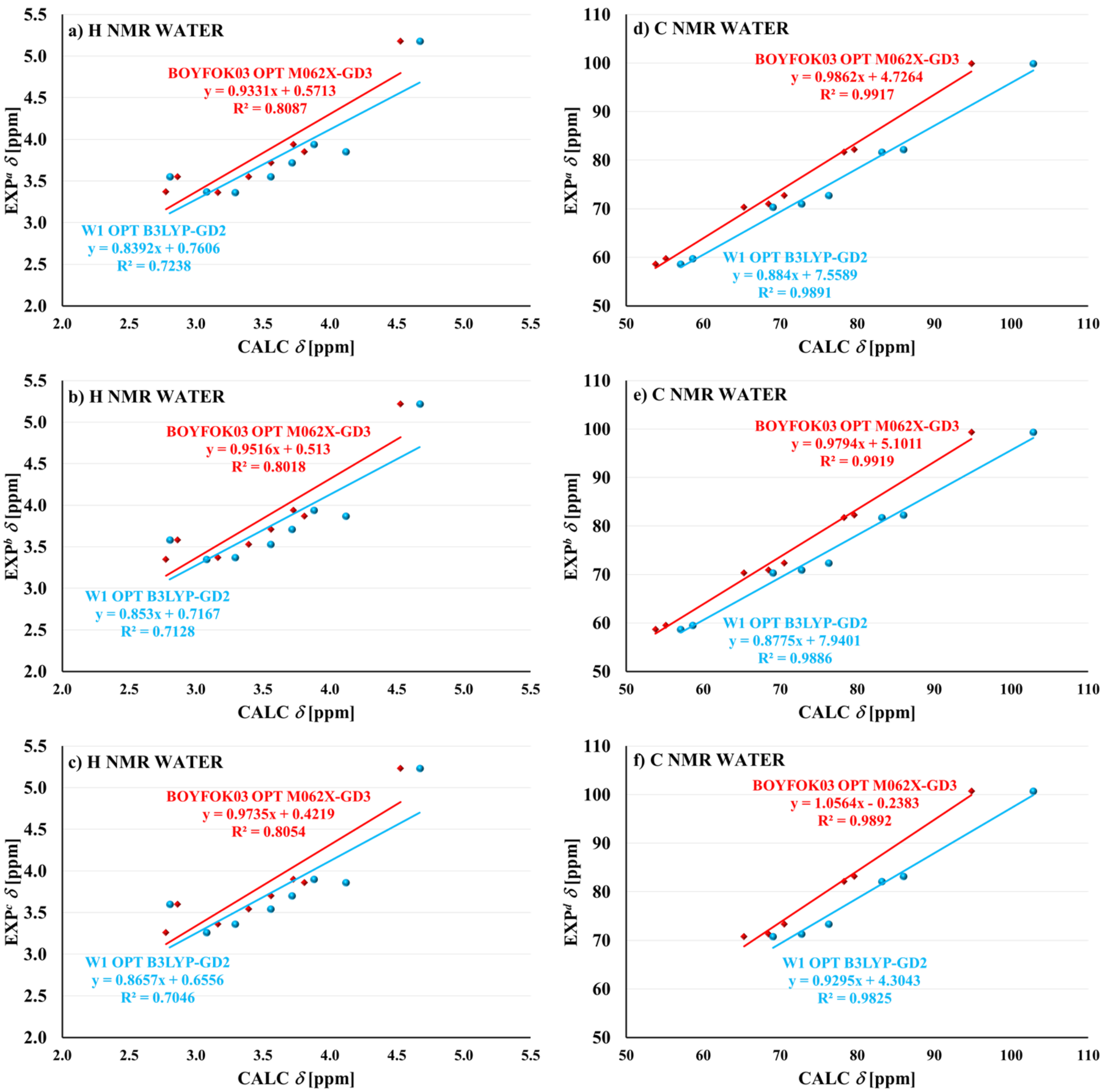

Figure S10. The linear regression between the ^{1}H and ^{13}C NMR chemical shifts δ measured in D_2O and calculated in water (as averages over δ values for all equivalent atoms present in DM-β-CD), corresponding to the results presented in Fig. 5 in the main article. The following notation is used: EXP – experimental data (^{a} ref. 11, ^{b} ref. 12, ^{c} ref. 13, ^{d} ref. 14), CALC – calculated δ values (BOYFOK03 OPT M062X-GD3 – obtained from the M062X/6-31++G(d,p) calculations for the crystal structure BOYFOK03 optimized with the M062X-GD3/6-31G(d,p) method, W1 OPT B3LYP-GD2 – obtained from the B3LYP/6-31++G(d,p) calculations for the conformer W1 optimized with the B3LYP-GD2/6-31G(d,p) method).

Table S6. The total electronic energies (*E*), enthalpies *H* and corrected Gibbs energies (G_{corr}) obtained from the B3LYP-GD2/6-31G(d,p) and M062X-GD3/6-31G(d,p) calculations performed in water (PCM) for the optimized (OPT) structures of the molecule MIA and the 11 configurations of MIA:DM-β-CD shown and in Fig. 6 in the main article (B3LYP-GD2 based on the structure W1 of DM-β-CD and M062X-GD3 based on the optimized BOYFOK03), as well as the corresponding BSSE corrected complexation energies, enthalpies and corrected Gibbs energies.

**B3LYP-GD2/6-31G(d,p) OPT**						
**Structure**	E [hartree]	H [hartree]	G_{corr} [hartree]	E^{BSSE}_{compl} [kcal/mol]	H^{BSSE}_{compl} [kcal/mol]	$G^{BSSE}_{corr\_compl}$ [kcal/mol]
**MIA**	-807.551356	-807.191080	-807.236438			
Complexes MIA:DM-β-CD_W1:						
**CR1**	-5633.673506	-5631.600097	-5631.823352	-30.0	-27.5	-17.9
**NR1**	-5633.670038	-5631.595127	-5631.817524	-25.6	-22.3	-12.1
**M1**	-5633.665950	-5631.590865	-5631.812970	-25.7	-22.2	-11.9
**FΛ1**	-5633.654559	-5631.580928	-5631.805435	-20.2	-17.6	-8.8
**FV1**	-5633.662649	-5631.587641	-5631.809570	-23.3	-19.9	-9.4
**CR2**	-5633.629793	-5631.557523	-5631.783449	-11.3	-9.6	-1.6
**NR2**	-5633.638187	-5631.565903	-5631.791783	-16.7	-14.9	-7.0
**M2**	-5633.628706	-5631.556022	-5631.781306	-12.3	-10.3	-2.0
**FΛ2**	-5633.631248	-5631.557737	-5631.782403	-12.6	-10.0	-1.3
**FV2**	-5633.637132	-5631.564299	-5631.789100	-15.5	-13.4	-4.8
**S**	-5633.630598	-5631.558350	-5631.785696	-12.8	-11.0	-4.0
**M062X-GD3/6-31G(d,p) OPT**						
**MIA**	-807.144935	-806.780708	-806.825847			
Complexes MIA:DM-β-CD_BOYFOK03:						
**CR1**	-5631.031856	-5628.936882	-5629.163499	-14.4	-12.6	-2.7
**NR1**	-5631.041248	-5628.946418	-5629.172434	-20.9	-19.2	-9.0
**M1**	-5631.032364	-5628.938178	-5629.165000	-16.5	-15.2	-5.5
**FΛ1**	-5631.036597	-5628.941696	-5629.166441	-18.3	-16.5	-5.5
**FV1**	-5631.028068	-5628.933587	-5629.161460	-13.9	-12.4	-3.3
**CR2**	-5631.008651	-5628.914577	-5629.143098	-6.9	-5.7	3.0
**NR2**	-5631.003218	-5628.908404	-5629.135399	-3.0	-1.3	8.3
**M2**	-5631.015114	-5628.921279	-5629.150992	-11.2	-10.1	-2.2
**FΛ2**	-5631.013055	-5628.918482	-5629.145901	-9.3	-7.7	1.6
**FV2**	-5631.003217	-5628.908416	-5629.135429	-3.0	-1.4	8.3
**S**	-5631.009524	-5628.915556	-5629.145038	-8.7	-7.6	0.5

Table S7. The total electronic energies (E) obtained from the single point calculations B3LYP-GD2/6-31++G(d,p) and M062X-GD3/6-31++G(d,p) performed in water (PCM) for MIA and the eleven MIA:DM-β-CD structures shown in Fig. 6 in the main article, and the corresponding BSSE corrected complexation energies and Gibbs energies. The latter are calculated by adding to the SP energies the thermal corrections obtained from the values (OPT) given in Table S6.

**Structure**	E [hartree]	$E_{\mathrm{compl}}^{\mathrm{BSSE}}$ [kcal/mol]	$G_{\mathrm{corr\_compl}}^{\mathrm{BSSE}}$ [kcal/mol]
**B3LYP-GD2/6-31++G(d,p) SP**			
**MIA**	-807.577421		
Complexes MIA:DM-β-CD_W1:			
**CR1**	-5633.855971	-29.6	-17.5
**NR1**	-5633.848597	-24.3	-10.8
**M1**	-5633.846785	-22.8	-9.0
**FΛ1**	-5633.837500	-18.3	-6.8
**FV1**	-5633.843966	-22.1	-8.2
**CR2**	-5633.822921	-10.8	-1.1
**NR2**	-5633.830932	-16.1	-6.4
**M2**	-5633.823081	-11.7	-1.4
**FΛ2**	-5633.824386	-12.4	-1.1
**FV2**	-5633.830364	-15.3	-4.6
**S**	-5633.824781	-12.6	-3.8
**M062X-GD3/6-31++G(d,p) SP**			
**MIA**	-807.166090		
Complexes MIA:DM-β-CD_BOYFOK03:			
**CR1**	-5631.189154	-17.1	-5.4
**NR1**	-5631.201390	-23.9	-11.9
**M1**	-5631.196101	-19.6	-8.6
**FΛ1**	-5631.196163	-21.1	-8.3
**FV1**	-5631.190543	-16.6	-6.1
**CR2**	-5631.173514	-8.3	1.6
**NR2**	-5631.166481	-3.2	8.1
**M2**	-5631.179517	-13.0	-4.0
**FΛ2**	-5631.176848	-10.2	0.8
**FV2**	-5631.166492	-3.2	8.1
**S**	-5631.175167	-9.6	-0.3

Table S8. The computed ^{1}H and ^{13}C NMR chemical shifts δ [ppm] for individual atoms of DM-β-CD in the most stable conformers of MIA:DM-β-CD in water indicated by the methods B3LYP-GD2 (CR1) and M062X-GD3 (NR1), obtained from the B3LYP/6-31++G(d,p)//B3LYP-GD2/6-31G(d,p) and M062X/6-31++G(d,p)//M062X-GD3/6-31G(d,p) calculations, respectively. The δ values are scaled as described in Table S5.

	**a) Protons**					
**Atom no. in Fig. S2b**	**CR1 B3LYP**			**NR1 M062X**		
	**Atom no. in Table S9**	**Isotropic**	***δ***	**Atom no. in Table S10**	**Isotropic**	***δ***
H-1	11	27.0276	4.38	14	26.9186	4.41
H-1	33	26.9005	4.50	40	26.6941	4.60
H-1	54	26.7429	4.65	67	26.9901	4.34
H-1	75	27.2540	4.17	94	26.6348	4.65
H-1	96	27.2060	4.21	121	27.0449	4.30
H-1	117	26.6595	4.73	148	27.2445	4.13
H-1	138	26.6655	4.72	175	26.8159	4.49
H-2	10	28.5685	2.92	15	28.6282	2.93
H-2	32	28.4221	3.06	41	29.0444	2.58
H-2	53	28.0742	3.39	68	28.6909	2.88
H-2	74	28.5064	2.98	95	28.7349	2.84
H-2	95	28.7322	2.77	122	29.3048	2.35
H-2	116	28.1601	3.31	149	29.0824	2.54
H-2	137	28.4995	2.99	176	28.4153	3.12
H-3	9	27.7715	3.68	16	28.0958	3.39
H-3	31	27.5781	3.86	42	28.4067	3.12
H-3	52	26.6528	4.73	69	26.8241	4.49
H-3	73	27.4220	4.01	96	27.0934	4.26
H-3	94	28.9871	2.53	123	30.3230	1.47
H-3	115	26.9598	4.44	150	26.3543	4.89
H-3	136	27.0282	4.38	177	26.5581	4.72
H-4	8	28.9378	2.57	17	28.5581	2.99
H-4	30	28.7988	2.70	43	29.3814	2.28
H-4	51	28.7213	2.78	70	28.1721	3.33
H-4	72	28.7163	2.78	97	29.3425	2.32
H-4	93	29.4598	2.08	124	29.0276	2.59
H-4	114	28.7037	2.79	151	28.1956	3.31
H-4	135	28.5252	2.96	178	28.7458	2.83
H-5	7	27.6707	3.77	18	28.4039	3.13
H-5	29	27.3239	4.10	44	28.7868	2.80
H-5	50	27.1825	4.23	71	27.6423	3.78

H-5	71	27.6461	3.79	98	27.0135	4.32
H-5	92	28.3206	3.16	125	27.7383	3.70
H-5	113	27.1245	4.29	152	27.0351	4.31
H-5	134	27.0306	4.38	179	27.4846	3.92
H-6	17	27.3629	4.06	19	27.5150	3.89
H-6	19	28.0355	3.43	45	28.3989	3.13
H-6	38	27.4696	3.96	46	28.7909	2.79
H-6	40	27.9044	3.55	72	27.6981	3.73
H-6	59	26.7549	4.64	73	28.2134	3.29
H-6	61	28.4368	3.05	99	28.1934	3.31
H-6	80	27.2044	4.21	100	27.7096	3.72
H-6	82	28.2746	3.20	126	28.7371	2.84
H-6	101	27.5633	3.87	127	27.7513	3.69
H-6	103	28.4122	3.07	153	27.3179	4.06
H-6	122	27.6969	3.75	154	28.1706	3.33
H-6	124	27.9034	3.55	180	28.0134	3.46
H-6	142	26.7645	4.63	181	28.2606	3.25
H-6	144	28.4519	3.03	189	28.5050	3.04
H-7	169	27.9796	3.48	20	27.9107	3.55
H-7	170	28.2898	3.19	21	28.2091	3.29
H-7	171	27.2582	4.16	22	27.1523	4.20
H-7	172	28.0273	3.43	47	28.0459	3.44
H-7	173	28.1961	3.27	48	28.4652	3.07
H-7	174	27.3224	4.10	49	27.2737	4.10
H-7	175	27.7970	3.65	74	27.7307	3.71
H-7	176	28.1107	3.35	75	28.1118	3.38
H-7	177	26.7674	4.63	76	27.0704	4.28
H-7	178	27.7090	3.74	101	27.9526	3.52
H-7	179	28.1361	3.33	102	28.3472	3.18
H-7	180	26.9199	4.48	103	27.2778	4.10
H-7	181	27.8646	3.59	128	28.0813	3.40
H-7	182	28.2346	3.24	129	28.4114	3.12
H-7	183	27.0329	4.37	130	27.3710	4.02
H-7	184	27.6997	3.74	155	28.1006	3.39
H-7	185	27.9650	3.49	156	28.3870	3.14
H-7	186	26.9934	4.41	157	27.5192	3.89
H-7	187	27.8862	3.57	182	27.9330	3.53
H-7	188	28.1466	3.32	183	28.3524	3.17
H-7	189	27.1300	4.28	184	26.7219	4.58
H-8	148	28.3337	3.14	23	28.2609	3.25
H-8	149	27.6812	3.76	24	28.0750	3.41
H-8	150	27.4053	4.02	25	28.7660	2.81
H-8	151	27.9037	3.55	50	28.8425	2.75

H-8	152	27.7997	3.65	51	29.3977	2.27
H-8	153	28.0869	3.38	52	29.2672	2.38
H-8	154	28.0477	3.41	77	28.1326	3.36
H-8	155	27.8871	3.57	78	28.5060	3.04
H-8	156	28.4753	3.01	79	27.7096	3.72
H-8	157	27.9963	3.46	104	28.0308	3.45
H-8	158	27.7388	3.71	105	27.9210	3.54
H-8	159	28.2579	3.22	106	27.9283	3.54
H-8	160	28.6613	2.83	131	28.3048	3.21
H-8	161	28.2674	3.21	132	28.4922	3.05
H-8	162	28.0134	3.45	133	27.8499	3.60
H-8	163	27.8726	3.58	158	28.2188	3.29
H-8	164	27.7665	3.68	159	28.5898	2.97
H-8	165	28.1539	3.31	160	28.0871	3.40
H-8	166	27.8730	3.58	185	28.2859	3.23
H-8	167	27.9066	3.55	186	28.2294	3.28
H-8	168	28.3203	3.16	187	29.2278	2.42
HO(-3)	20	25.7665	5.57	26	26.6266	4.66
HO(-3)	41	26.1781	5.18	53	26.4247	4.83
HO(-3)	62	26.7640	4.63	80	25.9674	5.23
HO(-3)	83	25.4788	5.85	107	26.3334	4.91
HO(-3)	104	27.1098	4.30	134	26.9944	4.34
HO(-3)	125	25.8125	5.53	161	26.8233	4.49
HO(-3)	145	25.7694	5.57	188	26.2787	4.96

b) Carbons

Atom no. in Fig. S2b	CR1 B3LYP			NR1 M062X		
	Atom no. in Table S9	Isotropic	δ	Atom no. in Table S10	Isotropic	δ
C1	6	88.8366	104.24	1	92.4731	95.83
C1	28	88.7002	104.38	27	94.9613	93.58
C1	49	86.6357	106.51	54	93.0368	95.32
C1	70	88.1708	104.93	81	91.1027	97.07
C1	91	89.7599	103.29	108	94.3126	94.16
C1	112	87.9803	105.12	135	95.0608	93.49
C1	133	89.1953	103.87	162	92.0381	96.22
C2	5	109.9182	82.48	2	112.8043	77.41
C2	27	108.9119	83.52	28	113.3558	76.91
C2	48	110.4756	81.90	55	112.2203	77.94
C2	69	107.9648	84.49	82	112.3675	77.80
C2	90	110.3486	82.03	109	113.2771	76.98
C2	111	109.0600	83.36	136	111.1915	78.87
C2	132	109.4669	82.94	163	113.7756	76.53

C3	4	114.8304	77.41	3	120.1989	70.71
C3	26	114.3359	77.92	29	120.6781	70.27
C3	47	116.7627	75.41	56	120.4031	70.52
C3	68	116.7953	75.38	83	117.2926	73.34
C3	89	117.6730	74.47	110	121.4185	69.60
C3	110	114.8464	77.39	137	123.1553	68.03
C3	131	116.2707	75.92	164	118.9935	71.80
C4	3	106.3629	86.15	4	116.3174	74.22
C4	25	107.8127	84.65	30	110.9498	79.09
C4	46	104.3072	88.27	57	112.6575	77.54
C4	67	103.2829	89.33	84	109.3323	80.55
C4	88	106.1817	86.33	111	118.8941	71.89
C4	109	108.5776	83.86	138	111.2427	78.82
C4	130	104.5718	88.00	165	109.7747	80.15
C5	2	122.9117	69.06	5	122.5520	68.58
C5	24	125.0786	66.83	31	128.2935	63.37
C5	45	118.7078	73.40	58	122.3590	68.75
C5	66	121.1121	70.92	85	126.9301	64.61
C5	87	125.2587	66.64	112	118.7864	71.99
C5	108	124.3657	67.56	139	121.5626	69.47
C5	129	119.6186	72.46	166	129.7001	62.10
C6	12	117.0810	75.08	6	125.7135	65.71
C6	34	119.5777	72.51	32	121.7886	69.27
C6	55	118.9787	73.12	59	122.7450	68.40
C6	76	119.7320	72.35	86	119.8168	71.05
C6	97	120.0710	72.00	113	125.4632	65.94
C6	118	119.4568	72.63	140	124.6450	66.68
C6	139	119.0251	73.08	167	122.5024	68.62
C7	21	132.3585	59.31	7	136.2223	56.19
C7	42	133.1191	58.53	33	137.0557	55.43
C7	63	132.5287	59.14	60	136.5127	55.93
C7	84	132.5530	59.11	87	136.1993	56.21
C7	105	132.4335	59.23	114	135.3672	56.96
C7	126	131.9203	59.76	141	136.0776	56.32
C7	146	131.8570	59.83	168	136.5944	55.85
C8	22	133.2857	58.35	8	136.5136	55.92
C8	43	134.5275	57.07	34	136.7405	55.72
C8	64	136.1837	55.36	61	137.7881	54.77
C8	85	132.9076	58.75	88	137.8103	54.75
C8	106	132.3005	59.37	115	135.9630	56.42
C8	127	133.0478	58.60	142	138.8556	53.80
C8	147	134.4538	57.15	169	140.2172	52.57

Table S9. Cartesian coordinates of atoms in the lowest energy structures of DM-β-CD in vacuo (V1) and in water (W1) obtained from the B3LYP-GD2/6-31G(d,p) calculations. The true minima are confirmed by zero imaginary frequencies.

		V1			W1		
**No.**	**Atom**	**x**	**y**	**z**	**x**	**y**	**z**
1	O	-3.0703	-5.4790	0.5019	3.5658	-4.2864	1.4784
2	C	-3.5932	-4.1510	0.6921	2.1580	-4.1601	1.1930
3	C	-4.2955	-3.6969	-0.5919	1.7720	-5.1899	0.1293
4	C	-3.4247	-3.8465	-1.8478	2.6458	-5.0929	-1.1265
5	C	-2.8111	-5.2434	-1.8979	4.1186	-5.1154	-0.7419
6	C	-2.1529	-5.5784	-0.5549	4.3977	-4.0728	0.3625
7	H	1.5722	-1.9028	2.1638	1.9534	-3.1560	0.8042
8	H	-5.2062	-4.2951	-0.7414	1.8748	-6.1987	0.5511
9	H	-2.6006	-3.1219	-1.7708	2.4326	-4.1236	-1.6019
10	H	-3.6128	-5.9753	-2.0756	4.3702	-6.1104	-0.3480
11	H	-1.8054	-6.6160	-0.5643	5.4207	-4.1725	0.7376
12	C	-4.5514	-4.2373	1.8741	1.4131	-4.3840	2.5032
13	O	-4.6216	-2.3088	-0.5231	0.4249	-4.9541	-0.2874
14	O	-4.2000	-3.6380	-3.0120	2.3741	-6.1696	-2.0012
15	O	-1.8004	-5.3775	-2.8944	4.8925	-4.8449	-1.9056
16	O	-1.0659	-4.6952	-0.3789	4.1920	-2.7877	-0.1839
17	H	-5.0943	-3.2862	1.9685	0.3743	-4.6683	2.2934
18	O	-3.8877	-4.5711	3.0784	1.4579	-3.2526	3.3648
19	H	-5.2688	-5.0420	1.6679	1.8998	-5.2093	3.0351
20	H	-4.7504	-2.8431	-2.8691	1.4282	-6.1067	-2.2481
21	C	-2.2872	-5.6438	-4.2100	6.2679	-5.2104	-1.7778
22	C	-3.4177	-3.4239	3.7762	0.5052	-2.2602	2.9935
23	O	1.9446	-4.8315	1.6677	6.1395	0.1790	0.7607
24	C	0.7118	-4.2652	1.1672	5.0913	-0.7985	0.8979
25	C	0.1138	-5.2579	0.1756	5.2787	-1.8617	-0.1861
26	C	1.0839	-5.5424	-0.9783	5.2808	-1.2309	-1.5835
27	C	2.4218	-6.0139	-0.4145	6.2719	-0.0692	-1.6495
28	C	2.9146	-5.0361	0.6688	6.0768	0.8846	-0.4598
29	H	5.9175	-0.8662	3.6227	4.1187	-0.3183	0.7421
30	H	-0.1123	-6.1953	0.7031	6.2332	-2.3832	-0.0314
31	H	1.2545	-4.5946	-1.5089	4.2743	-0.8218	-1.7593
32	H	2.2812	-7.0015	0.0501	7.2925	-0.4720	-1.5921
33	H	3.7892	-5.4571	1.1765	6.8929	1.6130	-0.4317
34	C	-0.1968	-3.9519	2.3645	5.1668	-1.3435	2.3151
35	O	0.5500	-6.5269	-1.8442	5.6241	-2.1910	-2.5671
36	O	3.4613	-6.0791	-1.3816	6.1178	0.7075	-2.8315
37	O	3.2446	-3.8184	0.0394	4.8345	1.5276	-0.6149
38	H	-1.1959	-4.3669	2.2060	4.5673	-2.2614	2.3813
39	O	-0.3792	-2.5570	2.5619	4.7274	-0.3910	3.2771
40	H	0.2402	-4.4174	3.2623	6.2107	-1.5782	2.5505
41	H	-0.2210	-6.1233	-2.2941	5.1013	-2.9982	-2.3895
42	C	3.4702	-7.2586	-2.1855	6.8779	0.2561	-3.9544
43	C	0.8115	-1.8926	2.9568	3.3069	-0.3225	3.3603
44	O	5.8223	-1.4967	1.2419	3.7682	4.7023	0.9024
45	C	4.5354	-2.1443	1.2435	3.9433	3.2727	0.8395
46	C	4.5530	-3.2803	0.2171	4.7609	2.9384	-0.4123
47	C	4.9849	-2.8066	-1.1774	4.1126	3.5010	-1.6820
48	C	6.2691	-1.9818	-1.0919	3.8174	4.9870	-1.5097

49	C	6.1472	-0.9076	-0.0004	3.0631	5.2399	-0.1948
50	H	2.8163	0.4952	3.2650	2.9640	2.7834	0.7723
51	H	5.2507	-4.0648	0.5432	5.7676	3.3660	-0.3122
52	H	4.1912	-2.1480	-1.5617	3.1527	2.9810	-1.8227
53	H	7.0981	-2.6526	-0.8210	4.7681	5.5348	-1.4570
54	H	7.1140	-0.4139	0.1441	3.0011	6.3169	-0.0119
55	C	4.2529	-2.6204	2.6610	4.6337	2.8527	2.1197
56	O	5.2023	-3.9165	-2.0245	4.9679	3.3185	-2.7953
57	O	6.5778	-1.2916	-2.2941	3.0138	5.5013	-2.5672
58	O	5.1692	0.0170	-0.4069	1.7772	4.6767	-0.3037
59	H	3.3774	-3.2742	2.6380	4.9846	1.8172	2.0406
60	O	3.9407	-1.5367	3.5246	3.7137	2.9825	3.1948
61	H	5.1155	-3.1934	3.0334	5.5076	3.5066	2.2791
62	H	4.4545	-4.5352	-1.9070	5.2320	2.3764	-2.8146
63	C	7.1984	-2.0791	-3.3101	3.7490	6.0228	-3.6776
64	C	5.0456	-1.0283	4.2662	4.3429	2.7625	4.4490
65	O	4.9118	3.4982	0.7245	-1.3127	5.4047	1.5290
66	C	4.6265	2.0886	0.7668	-0.1178	4.6640	1.2043
67	C	5.4579	1.4129	-0.3233	0.6683	5.4522	0.1549
68	C	5.1334	1.9913	-1.7074	-0.1746	5.7771	-1.0839
69	C	5.2319	3.5183	-1.6828	-1.4874	6.4297	-0.6748
70	C	4.4831	4.1033	-0.4722	-2.1747	5.6027	0.4332
71	H	3.5662	1.9223	0.5465	-0.3944	3.6896	0.7857
72	H	6.5257	1.5720	-0.1166	1.0137	6.3941	0.6020
73	H	4.0904	1.7255	-1.9393	-0.4095	4.8227	-1.5795
74	H	6.2937	3.7907	-1.5845	-1.2793	7.4340	-0.2791
75	H	4.7178	5.1688	-0.3756	-3.0404	6.1378	0.8349
76	C	4.9441	1.5915	2.1666	0.6675	4.4806	2.4970
77	O	6.0309	1.4871	-2.6753	0.5286	6.6494	-1.9457
78	O	4.6784	4.1382	-2.8344	-2.3174	6.5243	-1.8281
79	O	3.1034	3.9146	-0.6741	-2.5687	4.3675	-0.1237
80	H	4.8895	0.4981	2.1623	1.7079	4.2237	2.2649
81	O	4.0924	2.1413	3.1576	0.0889	3.5099	3.3619
82	H	5.9638	1.8970	2.4281	0.6575	5.4331	3.0395
83	H	6.0458	0.5135	-2.5907	1.3646	6.2015	-2.1907
84	C	5.5217	4.1515	-3.9856	-3.4013	7.4433	-1.6839
85	C	2.7819	1.5854	3.1404	0.4126	2.1757	2.9817
86	O	0.4040	5.8651	0.8821	-5.6107	2.5543	0.8554
87	C	1.3705	4.7960	0.8084	-4.2623	3.0362	0.9980
88	C	2.2170	5.0092	-0.4494	-3.9546	4.0274	-0.1264
89	C	1.3493	5.0904	-1.7129	-4.2240	3.4290	-1.5099
90	C	0.2330	6.1147	-1.5263	-5.6194	2.8129	-1.5605
91	C	-0.5056	5.8836	-0.1984	-5.8426	1.8789	-0.3610
92	H	0.1730	2.3307	2.8370	-3.5606	2.1970	0.9160
93	H	2.7814	5.9477	-0.3535	-4.5750	4.9265	-0.0080
94	H	0.8803	4.1051	-1.8573	-3.4951	2.6199	-1.6676
95	H	0.6852	7.1170	-1.4850	-6.3665	3.6160	-1.5009
96	H	-1.1791	6.7254	-0.0074	-6.8896	1.5624	-0.3340
97	C	2.1675	4.8084	2.1051	-4.1609	3.6612	2.3825
98	O	2.1390	5.4636	-2.8235	-4.1130	4.4238	-2.5133
99	O	-0.7437	6.0644	-2.5581	-5.8220	2.0395	-2.7385
100	O	-1.2270	4.6807	-0.2860	-4.9879	0.7687	-0.5055
101	H	3.0166	4.1258	2.0212	-3.2398	4.2546	2.4482
102	O	1.3790	4.3608	3.2003	-4.1945	2.6748	3.4065
103	H	2.5541	5.8235	2.2840	-5.0236	4.3199	2.5329
104	H	2.9119	4.8660	-2.8538	-3.2991	4.9354	-2.3340

105	C	-0.4179	6.8009	-3.7372	-6.3618	2.7655	-3.8458
106	C	0.7356	5.3919	3.9366	-2.9037	2.1316	3.6578
107	O	-4.1130	3.5016	1.6471	-5.2408	-2.5531	1.0232
108	C	-2.7261	3.5619	1.2645	-4.7277	-1.2167	0.8692
109	C	-2.5629	4.6706	0.2219	-5.5235	-0.5278	-0.2387
110	C	-3.4943	4.4910	-0.9811	-5.4344	-1.3103	-1.5566
111	C	-4.9297	4.2986	-0.4981	-5.8165	-2.7687	-1.3319
112	C	-4.9864	3.2055	0.5839	-5.0523	-3.3523	-0.1232
113	H	-2.8067	-0.4727	2.7137	-3.6775	-1.2622	0.5614
114	H	-2.7984	5.6329	0.6988	-6.5768	-0.4576	0.0630
115	H	-3.1891	3.5764	-1.5102	-4.3838	-1.2786	-1.8846
116	H	-5.2811	5.2422	-0.0541	-6.8922	-2.8187	-1.1104
117	H	-5.9906	3.1697	1.0194	-5.4415	-4.3406	0.1394
118	C	-1.9139	3.8635	2.5171	-4.8438	-0.5208	2.2162
119	O	-3.4258	5.6283	-1.8193	-6.2970	-0.7491	-2.5253
120	O	-5.8229	3.8857	-1.5218	-5.5308	-3.5026	-2.5179
121	O	-4.6589	1.9710	-0.0119	-3.6860	-3.4291	-0.4637
122	H	-0.9294	4.2363	2.2157	-4.5515	0.5290	2.0978
123	O	-1.7778	2.7604	3.3967	-4.0834	-1.1408	3.2492
124	H	-2.4327	4.6499	3.0768	-5.8865	-0.5526	2.5494
125	H	-2.5024	5.7043	-2.1344	-6.0548	0.1950	-2.6179
126	C	-6.3087	4.9255	-2.3701	-6.2090	-4.7589	-2.5871
127	C	-0.7949	1.8316	2.9580	-2.6974	-1.2535	2.9562
128	O	-6.0459	-1.2801	0.9973	-1.3389	-5.9222	0.8863
129	C	-5.1967	-0.1234	1.1106	-2.1226	-4.7149	0.9014
130	C	-5.6052	0.9004	0.0455	-3.0132	-4.6816	-0.3455
131	C	-5.6382	0.3045	-1.3680	-2.1903	-4.8213	-1.6284
132	C	-6.4257	-1.0056	-1.3787	-1.2901	-6.0494	-1.5406
133	C	-5.9490	-1.9285	-0.2471	-0.4821	-6.0293	-0.2320
134	H	-4.2424	-2.7336	3.9994	-1.4572	-3.8428	0.8840
135	H	-6.6075	1.2899	0.2744	-3.7379	-5.5061	-0.3012
136	H	-4.6002	0.0719	-1.6511	-1.5435	-3.9350	-1.7088
137	H	-7.4892	-0.7763	-1.2156	-1.9155	-6.9524	-1.5320
138	H	-6.5994	-2.8074	-0.1843	0.0467	-6.9796	-0.1138
139	C	-5.3660	0.4152	2.5262	-2.9271	-4.7051	2.1841
140	O	-6.2393	1.2069	-2.2751	-3.0441	-4.9365	-2.7533
141	O	-6.2705	-1.7463	-2.5817	-0.3591	-6.1069	-2.6163
142	H	-4.9616	1.4362	2.5720	-3.6846	-3.9121	2.1273
143	O	-4.7513	-0.4025	3.5049	-2.0500	-4.4656	3.2730
144	H	-6.4377	0.4397	2.7564	-3.4420	-5.6725	2.3024
145	H	-5.8856	2.0996	-2.0941	-3.7001	-4.2143	-2.6963
146	C	-7.0585	-1.3015	-3.6849	-0.8141	-6.8342	-3.7613
147	C	-3.3555	-0.1562	3.6142	-2.7655	-4.2901	4.4881
148	H	-4.1536	-0.4121	0.9379	0.5543	-1.4681	3.7460
149	H	-2.9710	-3.7838	4.7086	0.7315	-1.8197	2.0109
150	H	-2.6500	-2.8798	3.2111	-0.4994	-2.6952	2.9715
151	H	-2.7744	-3.4556	0.9136	3.0649	0.3656	4.1723
152	H	0.5458	-0.8540	3.1732	2.8589	0.0676	2.4351
153	H	1.2571	-2.3491	3.8532	2.8874	-1.3156	3.5643
154	H	0.9252	-3.3307	0.6356	3.5656	2.8251	5.2156
155	H	4.7291	-0.0681	4.6828	4.8172	1.7723	4.4847
156	H	5.3187	-1.7172	5.0801	5.1085	3.5299	4.6451
157	H	3.7605	-1.4230	0.9573	-0.0413	1.5133	3.7245
158	H	2.2333	2.0599	3.9547	0.0019	1.9193	1.9931
159	H	2.2520	1.8216	2.2055	1.5008	2.0312	2.9662
160	H	0.0905	6.0040	3.2959	-3.0376	1.2844	4.3348

161	H	0.1265	4.8934	4.6958	-2.4316	1.7717	2.7334
162	H	1.4765	6.0420	4.4271	-2.2309	2.8805	4.0968
163	H	0.8468	3.8356	0.7265	-2.1956	-1.5100	3.8910
164	H	-0.7214	1.0602	3.7302	-2.5007	-2.0473	2.2268
165	H	-1.0799	1.3439	2.0129	-2.2844	-0.3122	2.5714
166	H	-2.4230	2.6045	0.8252	-2.0309	-4.0655	5.2661
167	H	-3.1585	0.9085	3.7928	-3.4797	-3.4576	4.4058
168	H	-2.9917	-0.7509	4.4561	-3.3156	-5.2040	4.7601
169	H	-1.4069	-5.8157	-4.8355	6.7340	-5.0187	-2.7462
170	H	-2.9186	-6.5452	-4.2143	6.7790	-4.6111	-1.0135
171	H	-2.8681	-4.7977	-4.5860	6.3602	-6.2741	-1.5227
172	H	4.3727	-7.2029	-2.8003	6.7244	0.9914	-4.7482
173	H	3.5183	-8.1547	-1.5490	7.9467	0.2096	-3.7022
174	H	2.5764	-7.3141	-2.8125	6.5399	-0.7315	-4.2817
175	H	7.4824	-1.3840	-4.1049	3.0119	6.4378	-4.3693
176	H	8.1003	-2.5707	-2.9164	4.4315	6.8193	-3.3496
177	H	6.5143	-2.8426	-3.6903	4.3228	5.2293	-4.1648
178	H	5.0073	4.7587	-4.7354	-3.9155	7.4749	-2.6465
179	H	6.4912	4.6131	-3.7459	-4.1119	7.1179	-0.9131
180	H	5.6931	3.1385	-4.3600	-3.0264	8.4428	-1.4280
181	H	-1.2964	6.7459	-4.3856	-6.5265	2.0370	-4.6433
182	H	-0.2128	7.8535	-3.4905	-7.3194	3.2308	-3.5727
183	H	0.4559	6.3708	-4.2337	-5.6632	3.5392	-4.1775
184	H	-7.0560	4.4634	-3.0209	-5.9799	-5.1851	-3.5658
185	H	-6.7853	5.7152	-1.7707	-5.8614	-5.4511	-1.8094
186	H	-5.4993	5.3648	-2.9594	-7.2930	-4.6172	-2.4869
187	H	-6.9239	-2.0452	-4.4751	0.0181	-6.8485	-4.4692
188	H	-8.1208	-1.2572	-3.4027	-1.0771	-7.8647	-3.4849
189	H	-6.7406	-0.3124	-4.0260	-1.6834	-6.3429	-4.2075

Table S10. Cartesian coordinates of atoms in the DM-β-CD structures ZULQAY in vacuo and BOYFOK03 in water, obtained after the M062X-GD3/6-31++G(d,p) re-optimization of the structures given in Table S9. The true minima are confirmed by zero imaginary frequencies.

	ZULQAY				BOYFOK03			
**No.**	**Atom**	**x**	**y**	**z**	**Atom**	**x**	**y**	**z**
1	C	4.5160	3.9893	-0.4803	C	-2.6578	5.4732	-0.1770
2	C	5.0281	3.3625	-1.7803	C	-1.9691	5.9896	-1.4452
3	C	4.7939	1.8582	-1.7703	C	-0.6096	5.3256	-1.6207
4	C	5.3592	1.2668	-0.4836	C	0.1791	5.4678	-0.3256
5	C	4.8593	1.9986	0.7585	C	-0.6056	4.9143	0.8602
6	C	5.5202	1.4875	2.0316	C	0.1440	5.0547	2.1668
7	C	5.0075	3.9550	-4.0974	C	-2.7836	6.6841	-3.5773
8	C	7.6661	2.4896	1.8706	C	1.5337	6.4639	3.3903
9	O	4.3606	4.0228	-2.8343	O	-2.8389	5.7259	-2.5280
10	O	5.4271	1.2836	-2.8857	O	0.0750	5.9419	-2.6857
11	O	4.9377	-0.0848	-0.4578	O	1.4009	4.7739	-0.5094
12	O	5.1233	3.3995	0.6411	O	-1.8356	5.6390	0.9516
13	O	6.9113	1.2970	1.9235	O	0.6961	6.3469	2.2576
14	C	-0.2803	5.9717	0.3819	H	-3.5650	6.0543	0.0187
15	C	0.5915	6.5333	-0.7459	H	-1.8201	7.0734	-1.3339
16	C	1.6000	5.4839	-1.1896	H	-0.7719	4.2537	-1.8173
17	C	2.3488	4.9251	0.0191	H	0.3722	6.5326	-0.1458
18	C	1.3932	4.4584	1.1127	H	-0.8189	3.8472	0.6968
19	C	2.1182	4.0778	2.3829	H	0.9446	4.3029	2.2064
20	C	0.1949	7.9191	-2.6508	H	-3.5158	6.3734	-4.3233
21	C	1.8106	3.0074	4.4349	H	-3.0490	7.6804	-3.2043
22	O	-0.2933	6.9147	-1.7763	H	-1.7866	6.7143	-4.0219
23	O	2.5133	6.0589	-2.0923	H	1.9830	7.4579	3.3632
24	O	3.1267	3.8314	-0.4422	H	0.9559	6.3569	4.3189
25	O	0.5130	5.5255	1.4490	H	2.3188	5.6991	3.3656
26	O	1.2201	3.3876	3.2176	H	1.0132	5.6842	-2.6223
27	C	-4.9270	3.5661	0.1904	C	2.5849	5.4139	-0.1090
28	C	-4.6402	4.5370	-0.9612	C	3.5875	5.2736	-1.2603
29	C	-3.1408	4.6394	-1.2161	C	3.9848	3.8139	-1.4186
30	C	-2.4378	4.9223	0.1064	C	4.4502	3.2516	-0.0776
31	C	-2.8003	3.8842	1.1631	C	3.3812	3.4661	0.9926
32	C	-2.1215	4.1446	2.4911	C	3.8322	3.0299	2.3749
33	C	-5.6335	5.0252	-3.0808	C	3.8322	6.3528	-3.3766
34	C	-1.4164	5.8161	3.9418	C	1.9565	1.8925	3.2409
35	O	-5.3698	4.0628	-2.0711	O	2.9536	5.7873	-2.4134
36	O	-2.9006	5.6825	-2.1274	O	5.0070	3.6950	-2.3796
37	O	-1.0459	4.9311	-0.1622	O	4.6959	1.8723	-0.3005
38	O	-4.2176	3.9257	1.3463	O	3.0949	4.8612	1.0745
39	O	-2.1648	5.5201	2.7847	O	2.7792	3.0371	3.3161
40	C	-5.9003	-1.6235	-0.0558	H	2.3952	6.4685	0.1097
41	C	-6.5235	-0.7531	-1.1510	H	4.4805	5.8688	-1.0196
42	C	-5.7211	0.5286	-1.3328	H	3.0883	3.2523	-1.7249
43	C	-5.4695	1.2016	0.0171	H	5.3779	3.7623	0.2227
44	C	-4.8702	0.2167	1.0183	H	2.4754	2.9176	0.6962
45	C	-4.7155	0.7986	2.4119	H	4.5819	3.7437	2.7326
46	C	-7.5128	-1.1694	-3.2857	H	4.2958	2.0354	2.3138
47	C	-2.5893	0.0128	3.0603	H	3.2051	6.7503	-4.1754
48	O	-6.5557	-1.5542	-2.3121	H	4.4122	7.1713	-2.9340

49	O	-6.4246	1.3987	-2.1821	H	4.5135	5.5985	-3.7762
50	O	-4.5834	2.2787	-0.2447	H	1.2339	1.9596	4.0563
51	O	-5.7506	-0.8975	1.1355	H	1.4094	1.8314	2.2893
52	O	-3.9796	-0.0411	3.2757	H	2.5469	0.9736	3.3599
53	C	-2.4721	-5.5643	-0.3315	H	5.2671	2.7559	-2.4043
54	C	-3.2811	-5.3349	-1.6137	C	5.9202	1.3221	0.1067
55	C	-3.7151	-3.8773	-1.7203	C	6.4650	0.4942	-1.0702
56	C	-4.3709	-3.4592	-0.4096	C	5.5544	-0.6934	-1.3368
57	C	-3.4611	-3.7366	0.7816	C	5.3396	-1.4493	-0.0313
58	C	-4.0823	-3.3376	2.1031	C	4.8187	-0.5341	1.0726
59	C	-3.1520	-6.0807	-3.8787	C	4.6402	-1.2564	2.3899
60	C	-6.0775	-3.2351	3.2874	C	7.7075	2.1017	-2.2983
61	O	-2.4615	-5.7582	-2.6811	C	5.5626	-2.8725	3.7860
62	O	-4.6290	-3.7386	-2.7801	O	6.5513	1.2773	-2.2434
63	O	-4.6630	-2.0780	-0.5343	O	6.1553	-1.5316	-2.2929
64	O	-3.1769	-5.1370	0.8050	O	4.4275	-2.4955	-0.3136
65	O	-5.4336	-3.7268	2.1330	O	5.7681	0.5214	1.2517
66	C	2.7457	-5.2684	0.3217	O	5.7653	-2.0652	2.6436
67	C	2.1977	-6.1723	-0.7954	H	6.6214	2.1136	0.3921
68	C	0.8219	-5.6895	-1.2205	H	7.4606	0.1151	-0.7968
69	C	-0.0761	-5.5016	0.0025	H	4.5809	-0.3157	-1.6891
70	C	0.5878	-4.6483	1.0790	H	6.3023	-1.8645	0.2916
71	C	-0.2061	-4.6096	2.3642	H	3.8485	-0.1082	0.7785
72	C	4.2160	-6.9333	-1.7769	H	3.7367	-1.8785	2.3437
73	C	-0.3302	-3.5180	4.4239	H	4.5074	-0.5114	3.1918
74	O	3.0491	-6.1481	-1.9210	H	7.7001	2.5906	-3.2729
75	O	0.2481	-6.6267	-2.0898	H	7.6955	2.8727	-1.5191
76	O	-1.2600	-4.8768	-0.4659	H	8.6164	1.4988	-2.1983
77	O	1.8544	-5.2134	1.4025	H	6.4312	-3.5256	3.8836
78	O	0.3158	-3.5850	3.1780	H	5.4736	-2.2567	4.6918
79	C	5.8398	-1.0648	0.0014	H	4.6539	-3.4752	3.6717
80	C	5.9968	-2.0908	-1.1297	H	5.6980	-2.3913	-2.2537
81	C	4.6854	-2.8367	-1.3424	C	4.7225	-3.7737	0.1879
82	C	4.2011	-3.3710	-0.0012	C	4.5117	-4.7687	-0.9569
83	C	4.0786	-2.2559	1.0300	C	3.0355	-4.8376	-1.3192
84	C	3.6072	-2.7651	2.3752	C	2.1886	-5.0645	-0.0681
85	C	7.0646	-2.1109	-3.2674	C	2.5158	-4.0438	1.0194
86	C	3.7471	-4.5501	3.8567	C	1.8081	-4.3438	2.3283
87	O	6.4102	-1.3536	-2.2611	C	5.7791	-5.3428	-2.8987
88	O	4.8802	-3.9031	-2.2420	C	1.0609	-2.2171	3.0326
89	O	2.9564	-4.0020	-0.2401	O	5.3113	-4.3191	-2.0319
90	O	5.3678	-1.6585	1.1787	O	2.8253	-5.8777	-2.2448
91	O	4.2732	-3.9659	2.6871	O	0.8325	-4.9436	-0.4765
92	H	4.7845	5.0512	-0.4510	O	3.9166	-4.0951	1.2880
93	H	6.1126	3.5472	-1.8461	O	1.9402	-3.3000	3.2724
94	H	3.7067	1.6827	-1.7844	H	5.7544	-3.8143	0.5482
95	H	6.4557	1.3233	-0.5293	H	4.8477	-5.7606	-0.6206
96	H	3.7742	1.8352	0.8388	H	2.7477	-3.8624	-1.7425
97	H	5.1063	0.5023	2.2631	H	2.3832	-6.0769	0.3163
98	H	5.2846	2.1855	2.8509	H	2.2444	-3.0427	0.6552
99	H	5.0098	2.9342	-4.4822	H	2.2793	-5.2284	2.7711
100	H	6.0419	4.3135	-4.0188	H	0.7507	-4.5677	2.1429
101	H	4.4497	4.6138	-4.7646	H	6.4245	-4.8595	-3.6331
102	H	7.3514	3.1893	2.6564	H	6.3634	-6.0832	-2.3392
103	H	7.5673	2.9985	0.9051	H	4.9475	-5.8411	-3.4015
104	H	8.7092	2.2116	2.0319	H	1.1505	-1.5379	3.8831

105	H	5.5230	0.3291	-2.7150	H	1.3245	-1.6631	2.1202
106	H	-0.9283	6.7486	0.7972	H	0.0258	-2.5710	2.9509
107	H	1.1333	7.4131	-0.3649	H	1.8728	-5.8971	-2.4539
108	H	1.0422	4.6551	-1.6512	C	-0.0029	-6.0588	-0.3394
109	H	3.0001	5.7144	0.4251	C	-0.8608	-6.1598	-1.6050
110	H	0.8210	3.5928	0.7510	C	-1.8218	-4.9841	-1.6733
111	H	2.4810	4.9958	2.8738	C	-2.6065	-4.8777	-0.3687
112	H	2.9910	3.4542	2.1378	C	-1.6540	-4.8078	0.8247
113	H	1.0643	7.5678	-3.2097	C	-2.3703	-4.7982	2.1556
114	H	0.4682	8.8198	-2.0856	C	-0.4357	-6.9812	-3.8033
115	H	-0.6233	8.1607	-3.3308	C	-2.0083	-4.4408	4.4418
116	H	2.1541	3.8821	5.0051	O	0.0168	-6.1884	-2.7123
117	H	2.6698	2.3404	4.2702	O	-2.6978	-5.1535	-2.7616
118	H	1.0539	2.4774	5.0152	O	-3.3879	-3.7006	-0.4888
119	H	3.0770	5.3430	-2.4375	O	-0.8106	-5.9604	0.8056
120	H	-5.9889	3.6101	0.4574	O	-1.4226	-4.5262	3.1616
121	H	-5.0061	5.5336	-0.6682	H	0.5881	-6.9722	-0.2158
122	H	-2.7887	3.6706	-1.6055	H	-1.4374	-7.0945	-1.5519
123	H	-2.7590	5.9060	0.4703	H	-1.2259	-4.0645	-1.7837
124	H	-2.5080	2.8822	0.8154	H	-3.2560	-5.7610	-0.2701
125	H	-1.0779	3.8035	2.4394	H	-1.0446	-3.8957	0.7420
126	H	-2.6383	3.5633	3.2734	H	-2.8463	-5.7781	2.3220
127	H	-4.7152	5.3312	-3.5849	H	-3.1572	-4.0302	2.1405
128	H	-6.1174	5.9106	-2.6491	H	0.3369	-6.9267	-4.5708
129	H	-6.3177	4.5512	-3.7863	H	-0.5646	-8.0251	-3.4940
130	H	-0.3821	5.4668	3.8311	H	-1.3794	-6.5957	-4.1951
131	H	-1.8638	5.3477	4.8306	H	-1.2104	-4.2098	5.1489
132	H	-1.4280	6.9000	4.0693	H	-2.7657	-3.6462	4.4800
133	H	-1.9520	5.8994	-2.0900	H	-2.4820	-5.3894	4.7268
134	H	-6.5506	-2.4681	0.1872	H	-3.3627	-4.4428	-2.7107
135	H	-7.5472	-0.4856	-0.8450	C	-4.7441	-3.7365	-0.1287
136	H	-4.7405	0.2539	-1.7537	C	-5.5468	-3.1542	-1.3093
137	H	-6.4274	1.5800	0.4065	C	-5.2317	-1.6760	-1.4696
138	H	-3.8962	-0.1177	0.6338	C	-5.4189	-0.9863	-0.1247
139	H	-5.7124	0.9013	2.8535	C	-4.5626	-1.6423	0.9525
140	H	-4.2575	1.7954	2.3473	C	-4.7135	-0.9819	2.3039
141	H	-7.2750	-0.1928	-3.7120	C	-5.8263	-5.0988	-2.6451
142	H	-8.5169	-1.1329	-2.8434	C	-6.2504	0.0001	3.7497
143	H	-7.4918	-1.9386	-4.0592	O	-5.2296	-3.8159	-2.5156
144	H	-2.2189	1.0480	3.0978	O	-6.0918	-1.1110	-2.4300
145	H	-2.2938	-0.4255	2.0965	O	-5.0780	0.3729	-0.3192
146	H	-2.1170	-0.5624	3.8589	O	-4.9778	-3.0087	1.0493
147	H	-5.9320	2.2385	-2.2210	O	-6.0790	-0.7599	2.5704
148	H	-2.2930	-6.6367	-0.1959	H	-5.0564	-4.7627	0.0938
149	H	-4.1871	-5.9592	-1.5615	H	-6.6191	-3.2636	-1.0895
150	H	-2.8152	-3.2613	-1.8777	H	-4.1772	-1.5756	-1.7729
151	H	-5.2970	-4.0332	-0.2805	H	-6.4720	-1.0743	0.1727
152	H	-2.5226	-3.1753	0.6590	H	-3.5025	-1.6009	0.6671
153	H	-4.0068	-2.2488	2.2279	H	-4.1725	-0.0254	2.2994
154	H	-3.5199	-3.8220	2.9192	H	-4.2629	-1.6294	3.0733
155	H	-3.6074	-5.1933	-4.3219	H	-5.6129	-5.4449	-3.6566
156	H	-3.9342	-6.8256	-3.6834	H	-5.4040	-5.8184	-1.9338
157	H	-2.4113	-6.5081	-4.5561	H	-6.9102	-5.0377	-2.4997
158	H	-5.9790	-2.1443	3.3497	H	-7.3189	0.1882	3.8654
159	H	-5.6553	-3.6872	4.1965	H	-5.8857	-0.5496	4.6283
160	H	-7.1316	-3.5076	3.2133	H	-5.7122	0.9523	3.6724

161	H	-5.0831	-2.8844	-2.6680	H	-5.9813	-0.1450	-2.3721
162	H	3.6779	-5.6567	0.7437	C	-5.9218	1.3467	0.2379
163	H	2.1073	-7.1997	-0.4094	C	-6.2059	2.3790	-0.8654
164	H	0.9419	-4.7053	-1.7001	C	-4.9226	3.1063	-1.2273
165	H	-0.3056	-6.4942	0.4197	C	-4.2379	3.6414	0.0298
166	H	0.7149	-3.6235	0.7029	C	-4.0661	2.5517	1.0847
167	H	-0.1171	-5.5877	2.8649	C	-3.5532	3.0890	2.4087
168	H	-1.2687	-4.4363	2.1428	C	-8.0546	1.3306	-1.9235
169	H	3.9592	-7.9693	-1.5240	C	-1.8392	1.5913	3.0085
170	H	4.8937	-6.5284	-1.0143	O	-6.7028	1.7509	-2.0296
171	H	4.7311	-6.9120	-2.7379	O	-5.2156	4.1612	-2.1085
172	H	-0.2057	-4.4510	4.9918	O	-2.9695	4.1241	-0.3890
173	H	-1.4071	-3.3261	4.3065	O	-5.3350	1.9599	1.3532
174	H	0.1205	-2.6970	4.9842	O	-3.1424	2.0592	3.2834
175	H	-0.6096	-6.2679	-2.3775	H	-6.8406	0.8866	0.6133
176	H	6.8017	-0.6100	0.2555	H	-6.9379	3.1075	-0.4850
177	H	6.7742	-2.8170	-0.8460	H	-4.2391	2.3760	-1.6891
178	H	3.9430	-2.1201	-1.7277	H	-4.8441	4.4611	0.4430
179	H	4.9325	-4.1000	0.3711	H	-3.3781	1.7908	0.6898
180	H	3.3643	-1.4999	0.6753	H	-4.3734	3.6203	2.9028
181	H	2.5226	-2.9362	2.3395	H	-2.7364	3.8013	2.2276
182	H	3.8120	-1.9957	3.1376	H	-8.3607	0.9982	-2.9158
183	H	6.3909	-2.8462	-3.7119	H	-8.1681	0.4929	-1.2250
184	H	7.9374	-2.6285	-2.8491	H	-8.6955	2.1593	-1.6022
185	H	7.4000	-1.3965	-4.0208	H	-1.5970	0.8373	3.7597
186	H	2.6676	-4.7166	3.7521	H	-1.7599	1.1331	2.0134
187	H	3.9321	-3.9120	4.7335	H	-1.1089	2.4107	3.0722
188	H	4.2555	-5.5050	4.0016	H	-4.3774	4.6227	-2.2964
189	H	4.0529	-4.4171	-2.2598	H	-0.5493	4.8665	3.0035

Table S11. Cartesian coordinates of atoms in the DM-β-CD structures ZULQAY in vacuo and BOYFOK03 in water, obtained after the M062X-GD3/6-31++G(d,p) re-optimization of the structures given in Table S10.

	ZULQAY				BOYFOK03			
**No.**	**Atom**	**x**	**y**	**z**	**Atom**	**x**	**y**	**z**
1	C	4.4761	4.0818	-0.3800	C	-1.8188	5.7773	-0.1587
2	C	5.0827	3.5048	-1.6624	C	-1.0545	6.2137	-1.4138
3	C	4.8777	1.9969	-1.7134	C	0.2038	5.3750	-1.5921
4	C	5.3766	1.3724	-0.4145	C	1.0002	5.3893	-0.2939
5	C	4.7740	2.0509	0.8137	C	0.1376	4.9286	0.8791
6	C	5.3515	1.5113	2.1151	C	0.8839	4.9426	2.1944
7	C	5.2368	4.2232	-3.9434	C	-1.7783	7.0820	-3.5223
8	C	7.4920	2.5525	2.1952	C	2.4059	6.1602	3.4812
9	O	4.4772	4.1876	-2.7404	O	-1.9415	6.0923	-2.5098
10	O	5.5853	1.4727	-2.8130	O	0.9701	5.9075	-2.6527
11	O	4.9938	0.0096	-0.4628	O	2.1110	4.5290	-0.4868
12	O	5.0199	3.4599	0.7577	O	-0.9877	5.8071	0.9763
13	O	6.7513	1.3505	2.1053	O	1.5654	6.1687	2.3412
14	C	-0.4040	5.9746	0.3146	H	-2.6346	6.4780	0.0448
15	C	0.4723	6.5269	-0.8146	H	-0.7645	7.2661	-1.2817
16	C	1.5092	5.4915	-1.2261	H	-0.0922	4.3376	-1.8127
17	C	2.2601	4.9763	0.0012	H	1.3460	6.4121	-0.0961
18	C	1.2867	4.4872	1.0715	H	-0.2130	3.9012	0.6953
19	C	1.9789	4.0952	2.3561	H	1.6044	4.1130	2.2081
20	C	0.0510	7.9276	-2.7083	H	-2.5409	6.8841	-4.2762
21	C	1.6115	3.0146	4.3997	H	-1.9315	8.0834	-3.1042
22	O	-0.3999	6.8722	-1.8701	H	-0.7840	7.0154	-3.9697
23	O	2.4084	6.0744	-2.1409	H	2.9194	7.1222	3.5135
24	O	3.0877	3.9093	-0.4376	H	1.8155	6.0347	4.3990
25	O	0.3877	5.5459	1.3930	H	3.1381	5.3473	3.4058
26	O	1.0560	3.4017	3.1655	H	1.8738	5.5444	-2.5916
27	C	-5.0253	3.4999	0.1248	C	3.3775	4.9864	-0.0863
28	C	-4.7400	4.4450	-1.0479	C	4.3519	4.7167	-1.2388
29	C	-3.2393	4.5736	-1.2808	C	4.5499	3.2188	-1.4106
30	C	-2.5589	4.9110	0.0407	C	4.9347	2.5819	-0.0776
31	C	-2.9202	3.8970	1.1227	C	3.8983	2.9301	0.9916
32	C	-2.2701	4.2025	2.4550	C	4.2749	2.4145	2.3691
33	C	-5.7809	4.8803	-3.1604	C	4.7611	5.8626	-3.2983
34	C	-1.6433	5.9263	3.8920	C	2.2500	1.5304	3.2132
35	O	-5.4354	3.9271	-2.1629	O	3.8035	5.3159	-2.3970
36	O	-3.0073	5.5909	-2.2284	O	5.5467	2.9802	-2.3810
37	O	-1.1634	4.9215	-0.2142	O	4.9806	1.1836	-0.3170
38	O	-4.3410	3.9060	1.2817	O	3.8054	4.3515	1.0907
39	O	-2.3622	5.5823	2.7276	O	3.2243	2.5517	3.3062
40	C	-5.8822	-1.7234	0.0253	H	3.3429	6.0549	0.1453
41	C	-6.5550	-0.8791	-1.0624	H	5.3149	5.1862	-0.9908
42	C	-5.7840	0.4150	-1.2884	H	3.5922	2.7785	-1.7290
43	C	-5.5033	1.1164	0.0408	H	5.9253	2.9493	0.2312
44	C	-4.8410	0.1548	1.0254	H	2.9269	2.5172	0.6825
45	C	-4.6195	0.7573	2.4010	H	5.1147	3.0074	2.7467
46	C	-7.6633	-1.3861	-3.1220	H	4.5912	1.3652	2.2946
47	C	-2.4319	0.0294	2.9265	H	4.1976	6.3061	-4.1199
48	O	-6.6112	-1.6912	-2.2168	H	5.3510	6.6411	-2.8010

49	O	-6.5297	1.2554	-2.1378	H	5.4244	5.0822	-3.6792
50	O	-4.6485	2.2069	-0.2663	H	1.5310	1.6974	4.0171
51	O	-5.6979	-0.9720	1.1977	H	1.7185	1.5509	2.2515
52	O	-3.8077	-0.0529	3.2275	H	2.7090	0.5405	3.3393
53	C	-2.3864	-5.6145	-0.2897	H	5.7162	2.0196	-2.4025
54	C	-3.2395	-5.4227	-1.5491	C	6.1181	0.4557	0.0650
55	C	-3.7091	-3.9770	-1.6627	C	6.5077	-0.4469	-1.1173
56	C	-4.3414	-3.5519	-0.3427	C	5.4222	-1.4815	-1.3653
57	C	-3.3932	-3.7941	0.8283	C	5.1133	-2.1998	-0.0567
58	C	-3.9829	-3.3952	2.1643	C	4.7667	-1.2150	1.0584
59	C	-3.1731	-6.2635	-3.7889	C	4.5030	-1.8981	2.3820
60	C	-5.9443	-3.3435	3.4203	C	8.0299	0.8068	-2.4457
61	O	-2.4475	-5.8441	-2.6397	C	5.2041	-3.6264	3.7852
62	O	-4.6495	-3.8728	-2.7082	O	6.6990	0.3255	-2.2871
63	O	-4.6560	-2.1762	-0.4836	O	5.8751	-2.4022	-2.3344
64	O	-3.0703	-5.1863	0.8602	O	4.0329	-3.0779	-0.3244
65	O	-5.3216	-3.8247	2.2478	O	5.8678	-0.3144	1.2155
66	C	2.8516	-5.2237	0.2405	O	5.5098	-2.8523	2.6394
67	C	2.2897	-6.1051	-0.8868	H	6.9381	1.1312	0.3304
68	C	0.8989	-5.6371	-1.2773	H	7.4379	-0.9737	-0.8598
69	C	0.0207	-5.4965	-0.0333	H	4.5117	-0.9689	-1.7122
70	C	0.6942	-4.6383	1.0360	H	5.9983	-2.7742	0.2462
71	C	-0.0781	-4.6056	2.3344	H	3.8659	-0.6462	0.7833
72	C	4.1524	-7.0304	-2.0287	H	3.5238	-2.3947	2.3440
73	C	-0.1410	-3.5471	4.4212	H	4.4797	-1.1391	3.1804
74	O	3.1367	-6.0442	-2.0161	H	8.0499	1.3799	-3.3731
75	O	0.3340	-6.5722	-2.1606	H	8.3217	1.4597	-1.6148
76	O	-1.1924	-4.9018	-0.4655	H	8.7322	-0.0304	-2.5165
77	O	1.9722	-5.1961	1.3350	H	6.0001	-4.3631	3.9025
78	O	0.4588	-3.5853	3.1482	H	5.1617	-2.9940	4.6824
79	C	5.9065	-0.9701	-0.0238	H	4.2409	-4.1341	3.6530
80	C	6.0687	-1.9820	-1.1663	H	5.3050	-3.1930	-2.2945
81	C	4.7683	-2.7458	-1.3819	C	4.1200	-4.3953	0.1564
82	C	4.2999	-3.3105	-0.0478	C	3.7409	-5.3332	-0.9941
83	C	4.1742	-2.2128	1.0037	C	2.2656	-5.1809	-1.3327
84	C	3.7280	-2.7468	2.3478	C	1.4140	-5.3072	-0.0710
85	C	7.2010	-1.9671	-3.2755	C	1.8988	-4.3334	1.0031
86	C	3.9357	-4.5403	3.8213	C	1.1609	-4.4992	2.3189
87	O	6.4681	-1.2376	-2.2998	C	4.9743	-6.1131	-2.8892
88	O	4.9814	-3.7913	-2.3054	C	0.7970	-2.2591	2.9987
89	O	3.0535	-3.9417	-0.2882	O	4.5726	-5.0046	-2.0910
90	O	5.4507	-1.5879	1.1483	O	1.9035	-6.1614	-2.2815
91	O	4.4313	-3.9313	2.6491	O	0.0782	-5.0112	-0.4571
92	H	4.7292	5.1442	-0.2943	O	3.2794	-4.5947	1.2621
93	H	6.1646	3.7139	-1.6495	O	1.4703	-3.4806	3.2527
94	H	3.7994	1.7957	-1.8066	H	5.1341	-4.6082	0.5071
95	H	6.4723	1.4574	-0.3804	H	3.9339	-6.3663	-0.6704
96	H	3.6881	1.8685	0.8131	H	2.1083	-4.1712	-1.7428
97	H	4.9434	0.5113	2.2856	H	1.4794	-6.3361	0.3134
98	H	5.0436	2.1765	2.9376	H	1.7828	-3.3075	0.6250
99	H	5.3471	3.2225	-4.3648	H	1.4732	-5.4456	2.7745
100	H	6.2286	4.6529	-3.7536	H	0.0794	-4.5356	2.1421
101	H	4.6901	4.8694	-4.6317	H	5.6156	-5.7148	-3.6762
102	H	7.1091	3.1863	3.0065	H	5.5430	-6.8307	-2.2865
103	H	7.4549	3.1267	1.2626	H	4.1054	-6.6075	-3.3304
104	H	8.5232	2.2695	2.4129	H	0.9922	-1.6047	3.8507

105	H	5.6936	0.5122	-2.6841	H	1.1690	-1.7671	2.0888
106	H	-1.0570	6.7545	0.7168	H	-0.2830	-2.4278	2.9024
107	H	0.9893	7.4258	-0.4433	H	0.9444	-6.0869	-2.4476
108	H	0.9821	4.6394	-1.6813	C	-0.9039	-5.9946	-0.2713
109	H	2.8758	5.7901	0.4151	C	-1.7893	-6.0218	-1.5211
110	H	0.7273	3.6222	0.6872	C	-2.5885	-4.7323	-1.6227
111	H	2.3334	5.0051	2.8683	C	-3.3307	-4.4717	-0.3143
112	H	2.8501	3.4638	2.1299	C	-2.3549	-4.4876	0.8641
113	H	0.9751	7.6523	-3.2212	C	-3.0453	-4.3340	2.1998
114	H	0.2139	8.8385	-2.1178	C	-1.4996	-7.0206	-3.6741
115	H	-0.7460	8.1080	-3.4310	C	-2.6191	-3.9496	4.4742
116	H	1.9394	3.8895	4.9788	O	-0.9449	-6.2114	-2.6402
117	H	2.4714	2.3446	4.2539	O	-3.4967	-4.8289	-2.6983
118	H	0.8354	2.4863	4.9556	O	-3.9429	-3.2007	-0.4685
119	H	3.0419	5.3910	-2.4275	O	-1.6738	-5.7440	0.8777
120	H	-6.0913	3.5294	0.3757	O	-2.0593	-4.1642	3.1944
121	H	-5.1380	5.4382	-0.7874	H	-0.4437	-6.9758	-0.1175
122	H	-2.8560	3.6062	-1.6411	H	-2.4816	-6.8706	-1.4247
123	H	-2.8898	5.9046	0.3693	H	-1.8864	-3.8999	-1.7853
124	H	-2.5966	2.8935	0.8059	H	-4.0966	-5.2493	-0.1714
125	H	-1.2147	3.8974	2.4232	H	-1.6279	-3.6710	0.7398
126	H	-2.7801	3.6206	3.2409	H	-3.6532	-5.2296	2.4066
127	H	-4.8876	5.2958	-3.6308	H	-3.7119	-3.4608	2.1643
128	H	-6.3748	5.6917	-2.7210	H	-0.7412	-7.0922	-4.4542
129	H	-6.3870	4.3486	-3.8955	H	-1.7259	-8.0231	-3.2933
130	H	-0.5867	5.6471	3.7856	H	-2.4063	-6.5625	-4.0760
131	H	-2.0670	5.4261	4.7749	H	-1.7913	-3.8109	5.1711
132	H	-1.7293	7.0067	4.0196	H	-3.2529	-3.0529	4.4799
133	H	-2.0694	5.8507	-2.1809	H	-3.2205	-4.8118	4.7903
134	H	-6.5155	-2.5693	0.3076	H	-4.0921	-4.0567	-2.6605
135	H	-7.5739	-0.6312	-0.7253	C	-5.2961	-3.0407	-0.1285
136	H	-4.8131	0.1596	-1.7412	C	-5.9942	-2.3455	-1.3126
137	H	-6.4518	1.4823	0.4644	C	-5.4630	-0.9311	-1.4745
138	H	-3.8832	-0.1720	0.5959	C	-5.5596	-0.2126	-0.1347
139	H	-5.5875	0.8430	2.9061	C	-4.8312	-0.9895	0.9589
140	H	-4.1884	1.7621	2.2983	C	-4.9051	-0.3156	2.3100
141	H	-7.5429	-0.3848	-3.5413	C	-6.7271	-4.1013	-2.7384
142	H	-8.6340	-1.4564	-2.6143	C	-6.3142	0.8651	3.7501
143	H	-7.6169	-2.1378	-3.9113	O	-5.7765	-3.0664	-2.5092
144	H	-2.0833	1.0725	2.9436	O	-6.2273	-0.2527	-2.4484
145	H	-2.1924	-0.4034	1.9450	O	-4.9972	1.0729	-0.3301
146	H	-1.9011	-0.5374	3.6935	O	-5.4364	-2.2840	1.0485
147	H	-6.0587	2.1052	-2.2201	O	-6.2336	0.0808	2.5739
148	H	-2.1797	-6.6801	-0.1420	H	-5.7569	-4.0110	0.0842
149	H	-4.1259	-6.0702	-1.4576	H	-7.0715	-2.2916	-1.0979
150	H	-2.8329	-3.3392	-1.8580	H	-4.4054	-0.9782	-1.7770
151	H	-5.2575	-4.1346	-0.1807	H	-6.6177	-0.1229	0.1448
152	H	-2.4727	-3.2106	0.6730	H	-3.7702	-1.1005	0.6933
153	H	-3.9378	-2.3027	2.2736	H	-4.2487	0.5655	2.3087
154	H	-3.3813	-3.8535	2.9668	H	-4.5477	-1.0147	3.0822
155	H	-3.7232	-5.4301	-4.2302	H	-6.4613	-4.5696	-3.6863
156	H	-3.8746	-7.0651	-3.5245	H	-6.6970	-4.8572	-1.9449
157	H	-2.4349	-6.6489	-4.4937	H	-7.7369	-3.6830	-2.8056
158	H	-5.9047	-2.2472	3.4534	H	-7.3567	1.1621	3.8730
159	H	-5.4573	-3.7524	4.3171	H	-5.9976	0.2852	4.6276
160	H	-6.9831	-3.6768	3.3958	H	-5.6814	1.7561	3.6555

161	H	-5.1393	-3.0377	-2.5983	H	-6.0192	0.6975	-2.3801
162	H	3.7909	-5.6244	0.6358	C	-5.6770	2.1807	0.2015
163	H	2.2171	-7.1416	-0.5235	C	-5.7740	3.2378	-0.9112
164	H	0.9833	-4.6450	-1.7470	C	-4.3878	3.7537	-1.2562
165	H	-0.1726	-6.5003	0.3767	C	-3.6500	4.1893	0.0099
166	H	0.8112	-3.6119	0.6597	C	-3.6567	3.0792	1.0594
167	H	0.0174	-5.5846	2.8328	C	-3.0600	3.5165	2.3849
168	H	-1.1424	-4.4235	2.1334	C	-7.7800	2.7089	-2.0744
169	H	3.7120	-8.0340	-2.0604	C	-1.6641	1.7063	2.9847
170	H	4.8106	-6.9457	-1.1535	O	-6.3575	2.6804	-2.0730
171	H	4.7448	-6.8638	-2.9292	O	-4.5053	4.8361	-2.1519
172	H	0.0219	-4.4889	4.9641	O	-2.3197	4.4877	-0.3900
173	H	-1.2233	-3.3675	4.3456	O	-5.0080	2.7013	1.3207
174	H	0.3212	-2.7286	4.9756	O	-2.8458	2.4316	3.2667
175	H	-0.5506	-6.2537	-2.4147	H	-6.6660	1.8851	0.5643
176	H	6.8667	-0.5113	0.2306	H	-6.3855	4.0761	-0.5462
177	H	6.8592	-2.6965	-0.8892	H	-3.8138	2.9325	-1.7128
178	H	4.0092	-2.0425	-1.7580	H	-4.1359	5.0871	0.4209
179	H	5.0349	-4.0461	0.3044	H	-3.1034	2.2178	0.6594
180	H	3.4390	-1.4660	0.6697	H	-3.7643	4.1934	2.8797
181	H	2.6492	-2.9514	2.3186	H	-2.1223	4.0590	2.2047
182	H	3.9191	-1.9798	3.1159	H	-8.1029	2.2794	-3.0231
183	H	6.5918	-2.7624	-3.7105	H	-8.1971	2.1139	-1.2533
184	H	8.1035	-2.4002	-2.8256	H	-8.1422	3.7398	-1.9966
185	H	7.4925	-1.2474	-4.0420	H	-1.5548	0.9489	3.7627
186	H	2.8673	-4.7680	3.7120	H	-1.7079	1.2059	2.0081
187	H	4.0836	-3.8866	4.6935	H	-0.7893	2.3711	3.0007
188	H	4.4980	-5.4638	3.9705	H	-3.6107	5.1870	-2.3226
189	H	4.1864	-4.3555	-2.3075	H	0.1681	4.7975	3.0199

Table S12. Cartesian coordinates of atoms in 11 different structures of MIA:DM-β-CD in water (PCM) obtained from the B3LYP-GD2/6-31G(d,p) calculations performed using in the initial model of the complex the structure W1 of DM-β-CD. The true minima are confirmed by zero imaginary frequencies.

	CR1			NR1			M1		
**atom**	**x**	**y**	**z**	**x**	**y**	**z**	**x**	**y**	**z**
O	-4.1769	-4.0470	-1.7153	4.0540	3.3396	2.4445	-2.6029	4.7575	-2.0744
C	-2.7845	-4.0584	-1.3390	4.1338	1.9209	2.2122	-3.1261	3.4194	-1.9572
C	-2.5942	-4.9756	-0.1271	5.3975	1.6160	1.4016	-4.2085	3.4085	-0.8700
C	-3.5638	-4.6914	1.0246	5.4567	2.4546	0.1155	-3.6704	3.9538	0.4598
C	-4.9924	-4.6365	0.4979	5.1935	3.9324	0.3869	-3.0435	5.3239	0.2300
C	-5.0589	-3.6614	-0.6877	3.9454	4.0965	1.2649	-2.0040	5.2387	-0.9037
H	-2.4640	-3.0423	-1.0801	3.2639	1.5968	1.6297	-2.3230	2.7379	-1.6608
H	-2.7443	-6.0149	-0.4506	6.2941	1.8027	2.0075	-5.0586	4.0262	-1.1907
H	-3.3179	-3.7028	1.4359	4.6385	2.1019	-0.5218	-2.8717	3.2841	0.8046
H	-5.2956	-5.6337	0.1493	6.0537	4.3705	0.9116	-3.8196	6.0350	-0.0840
H	-6.0609	-3.6748	-1.1270	3.8431	5.1395	1.5784	-1.6329	6.2442	-1.1284
C	-2.0044	-4.5918	-2.5379	4.1305	1.2402	3.5736	-3.6642	3.0268	-3.3263
O	-1.2719	-4.7973	0.3834	5.3496	0.2533	0.9746	-4.6383	2.0771	-0.5955
O	-3.4472	-5.7015	2.0119	6.7077	2.3137	-0.5272	-4.7007	4.0533	1.4246
O	-5.9249	-4.1534	1.4569	4.9238	4.6651	-0.8046	-2.3785	5.8266	1.3881
O	-4.7391	-2.3722	-0.2058	2.8129	3.6858	0.5195	-0.9242	4.3764	-0.5718
H	-1.0175	-4.9395	-2.2055	4.4178	0.1863	3.4442	-4.1842	2.0625	-3.2426
O	-1.8741	-3.6528	-3.5983	2.8810	1.3469	4.2419	-2.6552	2.9742	-4.3261
H	-2.5576	-5.4494	-2.9381	4.8743	1.7335	4.2098	-4.3811	3.7899	-3.6504
H	-2.5532	-5.6120	2.4052	6.8611	1.3573	-0.6779	-5.2335	3.2309	1.3924
C	-6.4283	-5.1328	2.3684	6.0728	5.1068	-1.5314	-3.2102	6.5599	2.2966
C	-0.7912	-2.7535	-3.3819	1.9144	0.4322	3.7293	-1.8161	1.8300	-4.2133
O	-6.2104	0.7391	-1.4600	-0.2453	5.6961	0.6850	2.4612	5.6291	0.0613
C	-5.2989	-0.3743	-1.4551	0.6579	4.6649	1.1254	1.4130	5.0086	-0.7120
C	-5.7057	-1.3297	-0.3332	1.8517	4.6823	0.1735	0.1564	4.9465	0.1642
C	-5.7380	-0.6161	1.0268	1.3995	4.3550	-1.2523	0.4463	4.0567	1.3802
C	-6.5525	0.6770	0.9398	0.1939	5.2095	-1.6667	1.6391	4.6539	2.1465
C	-6.1288	1.5089	-0.2793	-0.8566	5.3876	-0.5491	2.8288	4.8584	1.1901
H	-4.2842	-0.0182	-1.2454	0.1676	3.6868	1.0556	1.7148	3.9845	-0.9613
H	-6.7010	-1.7464	-0.5366	2.3117	5.6786	0.1818	-0.0991	5.9585	0.4977
H	-4.6999	-0.3430	1.2736	1.1036	3.2937	-1.2670	0.7663	3.0816	0.9989
H	-7.6135	0.4167	0.8224	0.5754	6.2117	-1.9079	1.3559	5.6295	2.5661
H	-6.8150	2.3504	-0.4148	-1.4881	6.2478	-0.7913	3.6303	5.4128	1.6878
C	-5.3374	-1.0001	-2.8398	1.0140	4.9435	2.5771	1.2541	5.7832	-2.0290
O	-6.3033	-1.4481	2.0225	2.4495	4.5961	-2.1803	-0.6829	3.8358	2.2030
O	-6.3798	1.5178	2.0754	-0.4919	4.6687	-2.7925	2.1043	3.7914	3.1808
O	-4.8170	1.9603	-0.0524	-1.6298	4.2208	-0.4467	3.2608	3.5796	0.8049
H	-4.8693	-1.9929	-2.8010	1.8900	4.3434	2.8553	0.2426	6.2027	-2.1184
O	-4.6925	-0.1851	-3.8122	-0.0668	4.6669	3.4597	1.5427	4.9838	-3.1671
H	-6.3813	-1.1071	-3.1542	1.2538	6.0064	2.6895	1.9730	6.6072	-2.0377
H	-5.9637	-2.3568	1.8900	3.3012	4.4194	-1.7319	-1.3064	4.5802	2.0713
C	-7.2061	1.2102	3.2002	0.1064	4.9428	-4.0625	1.4387	3.9322	4.4385
C	-3.2744	-0.3050	-3.7709	-0.2190	3.2757	3.7231	0.5491	3.9914	-3.4118
O	-3.1157	4.7698	-1.6858	-4.9865	3.7778	0.9664	6.2920	2.2099	-0.6958
C	-3.5858	3.4138	-1.5704	-3.5471	3.8371	0.9979	5.0054	2.8516	-0.6654
C	-4.4916	3.3195	-0.3423	-3.0505	4.3567	-0.3529	4.6592	3.2913	0.7599

C	-3.8457	3.8485	0.9426	-3.6122	3.5826	-1.5512	4.8794	2.1903	1.8026
C	-3.1761	5.2005	0.7145	-5.1213	3.3751	-1.4309	6.2280	1.5048	1.6254
C	-2.3529	5.2027	-0.5860	-5.4870	2.8979	-0.0136	6.3588	1.0816	0.1534
H	-2.7349	2.7319	-1.4543	-3.1377	2.8367	1.1790	4.2369	2.1464	-0.9950
H	-5.4044	3.9007	-0.5311	-3.3439	5.4100	-0.4562	5.2481	4.1749	1.0400
H	-3.0731	3.1434	1.2649	-3.1410	2.5984	-1.5555	4.1254	1.4188	1.6216
H	-3.9628	5.9634	0.6123	-5.6303	4.3330	-1.6126	7.0522	2.1887	1.8678
H	-2.0572	6.2303	-0.8168	-6.5747	2.8962	0.1049	7.3308	0.6136	-0.0274
C	-4.3302	3.0837	-2.8474	-3.1591	4.7551	2.1371	5.0395	4.0270	-1.6186
O	-4.8550	3.9812	1.9331	-3.3094	4.2869	-2.7463	4.7441	2.7273	3.1089
O	-2.2682	5.5830	1.7386	-5.6305	2.3808	-2.3140	6.3210	0.3177	2.4098
O	-1.2080	4.3919	-0.4033	-4.9640	1.6027	0.1570	5.3065	0.1865	-0.1092
H	-4.8643	2.1338	-2.7317	-2.0880	4.9819	2.0834	4.1112	4.6005	-1.4903
O	-3.4025	2.9998	-3.9178	-3.4709	4.1118	3.3641	5.1600	3.5451	-2.9460
H	-5.0687	3.8807	-3.0387	-3.7240	5.6979	2.0425	5.8870	4.6863	-1.3662
H	-5.2739	3.1049	2.0320	-2.3338	4.3665	-2.7815	3.8287	3.0709	3.1754
C	-2.8399	5.8146	3.0282	-5.6773	2.7371	-3.6967	6.8443	0.5119	3.7255
C	-4.0600	2.7878	-5.1585	-3.2098	4.9495	4.4813	4.9369	4.5768	-3.9002
O	1.8747	4.7335	-2.3816	-5.6307	-1.2056	2.3995	4.6021	-2.3498	-2.6335
C	0.6315	4.1081	-2.0072	-5.0616	0.0512	1.9908	4.5030	-1.0670	-1.9784
C	0.0424	4.9199	-0.8497	-5.7870	0.5961	0.7541	5.5628	-0.9821	-0.8808
C	0.9996	4.9251	0.3504	-6.0435	-0.4506	-0.3415	5.4898	-2.1666	0.0877
C	2.3427	5.4860	-0.1218	-6.6036	-1.7276	0.2775	5.5105	-3.4734	-0.6959
C	2.8421	4.7149	-1.3539	-5.6347	-2.1747	1.3870	4.4131	-3.4466	-1.7818
H	0.8200	3.0782	-1.6807	-4.0126	-0.0945	1.7264	3.5171	-0.9653	-1.5106
H	-0.0880	5.9555	-1.1913	-6.7557	1.0204	1.0519	6.5650	-0.9425	-1.3288
H	1.1483	3.8904	0.6898	-5.0843	-0.7115	-0.7973	4.5298	-2.1140	0.6185
H	2.1894	6.5313	-0.4259	-7.5804	-1.5361	0.7423	6.4756	-3.5831	-1.2092
H	3.7260	5.2132	-1.7636	-5.9795	-3.1137	1.8336	4.4947	-4.3465	-2.4002
C	-0.2512	4.0837	-3.2366	-5.1712	0.9977	3.1786	4.6645	0.0009	-3.0553
O	0.4976	5.7382	1.3959	-6.9313	0.0826	-1.3119	6.5779	-2.1180	0.9960
O	3.3676	5.4166	0.8578	-6.7256	-2.8109	-0.6433	5.2778	-4.6165	0.1267
O	3.1440	3.3993	-0.9609	-4.3035	-2.3071	0.9082	3.1096	-3.3335	-1.2338
H	-1.2304	3.6717	-2.9739	-4.8921	2.0107	2.8575	5.0710	0.9199	-2.6174
O	0.3943	3.2890	-4.2204	-4.3952	0.5810	4.2937	3.4389	0.2564	-3.7339
H	-0.4066	5.1098	-3.6098	-6.2159	1.0082	3.5118	5.3795	-0.3694	-3.7991
H	-0.4542	5.5349	1.5134	-6.4741	0.8491	-1.7145	6.4577	-1.3126	1.5403
C	3.4278	6.5271	1.7516	-7.9270	-2.8025	-1.4261	6.4497	-5.1648	0.7437
C	-0.4774	2.9004	-5.2717	-3.0107	0.8963	4.1641	2.6727	1.2879	-3.1145
O	5.3227	0.8734	-2.4922	-2.2873	-5.3361	0.5177	0.4980	-5.9062	-1.1117
C	4.0813	1.5684	-2.2468	-2.7778	-4.1388	1.1593	1.2858	-4.8419	-1.6818
C	4.3710	2.8193	-1.4100	-3.8904	-3.5289	0.2953	2.4569	-4.5084	-0.7465
C	5.1789	2.5232	-0.1416	-3.3738	-3.2245	-1.1178	1.9689	-4.1898	0.6720
C	6.3989	1.6678	-0.4627	-2.7367	-4.4891	-1.7089	1.0386	-5.2992	1.1709
C	5.9796	0.4616	-1.3174	-1.7143	-5.1172	-0.7521	-0.0652	-5.5850	0.1420
H	3.4009	0.9176	-1.6868	-1.9640	-3.4121	1.2490	0.6649	-3.9453	-1.7859
H	4.9341	3.5366	-2.0227	-4.7317	-4.2311	0.2336	3.1507	-5.3557	-0.7158
H	4.5379	1.9502	0.5402	-2.5952	-2.4542	-1.0366	1.3733	-3.2743	0.6175
H	7.1284	2.2632	-1.0295	-3.5311	-5.2288	-1.8832	1.6136	-6.2256	1.3087
H	6.8633	-0.0997	-1.6366	-1.4228	-6.1060	-1.1201	-0.6376	-6.4658	0.4478
C	3.4873	1.9325	-3.6018	-3.2466	-4.5415	2.5721	1.7071	-5.3142	-3.0879
O	5.5948	3.7389	0.4557	-4.3848	-2.7397	-1.9813	3.0374	-3.9534	1.5753
O	7.0187	1.1256	0.7005	-2.0433	-4.2311	-2.9230	0.3884	-4.9422	2.3860
O	5.1239	-0.3474	-0.5397	-0.5863	-4.2707	-0.6652	-0.9010	-4.4525	0.0541
H	2.6920	2.6735	-3.4554	-4.3388	-4.4404	2.6611	2.8016	-5.3934	-3.1628
O	2.9957	0.8165	-4.3368	-2.6070	-3.8227	3.6149	1.2051	-4.4917	-4.1278

H	4.2807	2.3807	-4.2111	-2.9918	-5.5954	2.7173	1.2856	-6.3113	-3.2451
H	4.7838	4.2276	0.7029	-5.2608	-2.8859	-1.5687	3.8642	-4.2967	1.1790
C	7.9164	2.0057	1.3800	-2.8372	-4.2811	-4.1090	1.1060	-5.2613	3.5796
C	1.7652	0.3248	-3.8118	-3.1909	-2.5448	3.8540	1.9723	-3.3013	-4.2940
O	4.9971	-4.0071	-0.6148	2.8627	-5.4609	-0.2021	-4.3498	-4.1623	-1.1720
C	4.7649	-2.6742	-1.1054	1.5335	-5.0660	0.2028	-2.9155	-4.0513	-1.2473
C	5.5168	-1.6800	-0.2196	0.6896	-4.7953	-1.0465	-2.3163	-4.6659	0.0187
C	5.1590	-1.8470	1.2608	1.3473	-3.7303	-1.9353	-2.8823	-4.0094	1.2841
C	5.1754	-3.3086	1.7010	2.8049	-4.1026	-2.2181	-4.4084	-4.0152	1.2572
C	4.4730	-4.2138	0.6775	3.5505	-4.4633	-0.9189	-4.9087	-3.4544	-0.0856
H	3.6963	-2.4389	-1.0478	1.5836	-4.1359	0.7766	-2.6312	-2.9948	-1.2871
H	6.6005	-1.8073	-0.3419	0.5675	-5.7177	-1.6300	-2.5435	-5.7400	0.0416
H	4.1303	-1.4747	1.3807	1.3386	-2.7873	-1.3763	-2.5641	-2.9588	1.2880
H	6.2186	-3.6431	1.7785	2.8230	-4.9847	-2.8735	-4.7711	-5.0484	1.3547
H	4.6638	-5.2635	0.9195	4.5348	-4.8754	-1.1574	-5.9922	-3.5860	-0.1628
C	5.2206	-2.6392	-2.5562	0.9966	-6.1730	1.1121	-2.4772	-4.7474	-2.5272
O	6.0614	-1.1172	2.0763	0.6599	-3.5953	-3.1681	-2.4205	-4.6944	2.4342
O	4.5072	-3.4905	2.9453	3.5354	-3.0350	-2.8242	-4.9791	-3.1998	2.2732
O	3.0920	-3.9382	0.7182	3.6782	-3.2976	-0.1400	-4.5615	-2.0931	-0.1429
H	5.2959	-1.5916	-2.8798	-0.0731	-6.3325	0.9308	-1.4010	-4.9576	-2.4692
O	4.3490	-3.3586	-3.4199	1.2115	-5.8724	2.4892	-2.7819	-3.9961	-3.6961
H	6.2064	-3.1112	-2.6291	1.5335	-7.1007	0.8888	-3.0156	-5.6979	-2.6112
H	6.2156	-0.2482	1.6490	-0.3004	-3.5796	-2.9786	-1.4417	-4.6851	2.4108
C	5.3274	-3.3210	4.1041	3.3811	-2.9094	-4.2430	-5.0918	-3.8043	3.5630
C	3.1800	-2.6142	-3.7426	0.1383	-5.0931	3.0132	-1.8830	-2.9071	-3.8934
O	0.4124	-6.2029	-0.3767	5.8587	-1.6298	2.2478	-5.8737	0.7438	-2.0422
C	1.3734	-5.1412	-0.5389	4.6856	-2.3051	1.7582	-5.0274	-0.3924	-1.7811
C	2.1741	-5.0292	0.7617	4.9567	-2.9646	0.3974	-5.5488	-1.1480	-0.5568
C	1.2527	-4.7528	1.9553	5.6663	-2.0437	-0.6005	-5.8043	-0.2476	0.6575
C	0.1600	-5.8150	2.0123	6.8514	-1.3493	0.0599	-6.5798	1.0059	0.2658
C	-0.5294	-5.9640	0.6459	6.3562	-0.6625	1.3455	-5.9314	1.6548	-0.9692
H	0.8518	-4.1940	-0.7253	3.8836	-1.5719	1.6468	-4.0069	-0.0528	-1.5851
H	2.7093	-5.9718	0.9379	5.5649	-3.8692	0.5376	-6.4872	-1.6559	-0.8168
H	0.7727	-3.7763	1.7891	4.9578	-1.2611	-0.8880	-4.8312	0.0722	1.0452
H	0.6221	-6.7815	2.2561	7.6254	-2.0790	0.3335	-7.6147	0.7345	0.0132
H	-1.1812	-6.8420	0.6655	7.1806	-0.1550	1.8552	-6.5334	2.5011	-1.3132
C	2.2262	-5.4941	-1.7384	4.2623	-3.3458	2.7745	-5.0361	-1.2602	-3.0205
O	1.9852	-4.7701	3.1659	6.0732	-2.7836	-1.7415	-6.5181	-0.9715	1.6441
O	-0.8493	-5.5011	2.9688	7.4087	-0.3366	-0.7677	-6.5764	2.0057	1.2779
H	3.1157	-4.8557	-1.7802	3.6222	-4.0912	2.2790	-4.6051	-2.2450	-2.7928
O	1.4414	-5.3271	-2.9119	3.5473	-2.7142	3.8247	-4.2856	-0.6128	-4.0378
H	2.5551	-6.5420	-1.6401	5.1562	-3.8605	3.1626	-6.0806	-1.4036	-3.3421
H	2.7779	-4.2087	3.0465	5.2575	-2.9049	-2.2706	-5.9406	-1.7149	1.9175
C	-0.5998	-6.0027	4.2865	8.4116	-0.7799	-1.6849	-7.4500	1.7757	2.3857
C	2.1035	-5.8410	-4.0589	3.1202	-3.6603	4.7958	-4.4671	-1.2430	-5.2972
H	-0.7351	-2.0966	-4.2551	1.0267	0.5128	4.3630	-1.1904	1.8030	-5.1103
H	-0.9600	-2.1369	-2.4906	1.6263	0.6676	2.6966	-1.1570	1.8910	-3.3358
H	0.1531	-3.3033	-3.2656	2.2983	-0.5959	3.7549	-2.4122	0.9087	-4.1490
H	-2.8793	0.2858	-4.6001	-1.0018	3.1790	4.4778	0.8299	3.4855	-4.3385
H	-2.8538	0.0902	-2.8346	-0.5303	2.7165	2.8289	0.5061	3.2525	-2.5996
H	-2.9750	-1.3569	-3.8755	0.7226	2.8467	4.0941	-0.4524	4.4310	-3.5114
H	-3.2841	2.7152	-5.9254	-3.4383	4.3678	5.3789	5.0694	4.1326	-4.8908
H	-4.6468	1.8598	-5.1380	-2.1576	5.2625	4.5013	3.9169	4.9760	-3.8086
H	-4.7275	3.6302	-5.3995	-3.8493	5.8461	4.4587	5.6614	5.3967	-3.7743
H	0.1206	2.3239	-5.9830	-2.5464	0.6880	5.1331	1.7334	1.3613	-3.6666
H	-1.3024	2.2859	-4.8905	-2.5168	0.2741	3.4073	2.4283	1.0497	-2.0681

H	-0.9056	3.7769	-5.7827	-2.8728	1.9523	3.9030	3.2069	2.2449	-3.1470
H	1.3877	-0.4193	-4.5203	-2.6653	-2.1086	4.7075	1.4689	-2.6993	-5.0553
H	1.9071	-0.1609	-2.8404	-3.0951	-1.8864	2.9862	2.0501	-2.7350	-3.3625
H	1.0383	1.1379	-3.7002	-4.2611	-2.6328	4.0814	2.9942	-3.5395	-4.6246
H	2.6236	-3.1886	-4.4855	0.4138	-4.7900	4.0261	-2.0642	-2.5197	-4.8983
H	2.5345	-2.4687	-2.8658	-0.0478	-4.1975	2.4099	-2.0592	-2.0963	-3.1755
H	3.4469	-1.6294	-4.1473	-0.7882	-5.6796	3.0450	-0.8427	-3.2483	-3.8149
H	1.4724	-5.6178	-4.9236	2.4852	-3.1303	5.5105	-3.7949	-0.7521	-6.0062
H	3.0882	-5.3722	-4.1904	2.5498	-4.4729	4.3238	-4.2242	-2.3135	-5.2435
H	2.2383	-6.9311	-3.9789	3.9802	-4.0985	5.3258	-5.5066	-1.1356	-5.6442
H	-7.1950	-4.6322	2.9651	5.6993	5.7444	-2.3368	-2.5485	6.9419	3.0775
H	-6.8823	-5.9676	1.8166	6.7344	5.6927	-0.8785	-3.6853	7.4002	1.7738
H	-5.6315	-5.5154	3.0119	6.6285	4.2591	-1.9412	-3.9789	5.9137	2.7275
H	-7.0266	1.9972	3.9368	-0.6204	4.6307	-4.8168	1.9241	3.2299	5.1204
H	-8.2659	1.2143	2.9097	0.3101	6.0170	-4.1701	1.5520	4.9566	4.8208
H	-6.9488	0.2334	3.6185	1.0386	4.3839	-4.1836	0.3772	3.6933	4.3399
H	-2.0439	6.2527	3.6364	-6.2525	1.9526	-4.1949	6.8944	-0.4765	4.1886
H	-3.6794	6.5191	2.9545	-6.1811	3.7045	-3.8286	7.8528	0.9464	3.6803
H	-3.1826	4.8747	3.4646	-4.6705	2.7962	-4.1176	6.1905	1.1687	4.3067
H	4.3276	6.3906	2.3561	-7.9204	-3.7257	-2.0102	6.1260	-6.0630	1.2748
H	3.5062	7.4693	1.1911	-8.8027	-2.7922	-0.7638	7.1844	-5.4373	-0.0253
H	2.5432	6.5605	2.3933	-7.9555	-1.9305	-2.0841	6.8936	-4.4470	1.4378
H	8.3834	1.4165	2.1734	-2.1428	-4.1751	-4.9465	0.4406	-5.0197	4.4125
H	8.6905	2.3675	0.6892	-3.3520	-5.2501	-4.1805	1.3501	-6.3326	3.6070
H	7.3813	2.8602	1.8024	-3.5694	-3.4715	-4.1227	2.0243	-4.6733	3.6464
H	4.7011	-3.5688	4.9645	4.0818	-2.1319	-4.5557	-5.6327	-3.0896	4.1875
H	6.1875	-4.0041	4.0691	3.6289	-3.8567	-4.7408	-5.6575	-4.7446	3.4981
H	5.6861	-2.2905	4.1802	2.3588	-2.6194	-4.4944	-4.1040	-3.9974	3.9867
H	-1.4850	-5.7623	4.8803	8.7908	0.1150	-2.1843	-7.4548	2.7005	2.9683
H	-0.4585	-7.0922	4.2645	9.2320	-1.2717	-1.1440	-8.4672	1.5607	2.0308
H	0.2871	-5.5283	4.7147	7.9880	-1.4745	-2.4152	-7.0977	0.9397	2.9952
C	2.5594	3.4813	3.2233	1.4746	-1.2324	0.5514	-3.2090	0.8057	2.7822
C	-0.4367	0.9309	-0.1300	-1.1347	-0.8512	-4.1097	2.0179	0.5260	2.8832
C	3.8215	0.9992	3.0620	-1.1432	-0.6785	1.2376	-2.7446	-1.3472	4.4802
C	3.9543	3.4027	3.2321	0.9297	-1.8027	1.7009	-4.0662	0.5697	3.8593
C	1.2483	-1.1466	0.6131	-3.0792	0.2173	-2.4671	1.4240	-1.7201	4.3953
C	-0.1138	-0.0578	-1.0591	-2.4717	-0.9194	-4.4985	2.8766	0.1515	3.9190
C	4.5906	2.1646	3.1567	-0.3911	-1.5332	2.0503	-3.8374	-0.5059	4.7184
C	0.7260	-1.1085	-0.6807	-3.4522	-0.3689	-3.6740	2.5830	-0.9883	4.6703
C	-3.6988	1.9386	4.1217	3.1899	2.0558	-4.7011	0.4438	-0.9059	-2.2678
C	-0.2454	3.4257	3.9990	2.7614	0.2234	-1.4566	-1.9047	0.7019	0.1750
C	-1.8067	1.3992	2.7465	1.2664	1.0105	-3.7111	0.6044	-0.6192	0.1028
C	-1.5569	2.9243	4.6002	3.2840	1.2513	-2.4475	-1.4344	-0.0712	-1.0516
C	1.5736	-0.1763	2.9203	-1.3977	0.8994	-0.7244	-0.7358	-2.0792	3.1179
C	1.7852	2.3152	3.1468	0.7285	-0.3677	-0.2598	-2.1293	-0.0573	2.5205
C	0.0542	0.8819	1.1817	-0.7427	-0.2155	-2.9211	0.8838	-0.2296	2.5705
C	2.4298	1.0599	3.0581	-0.5990	-0.0674	0.1084	-1.8970	-1.1507	3.3909
C	0.9165	-0.1660	1.5549	-1.7368	0.3065	-2.0764	0.5680	-1.3542	3.3513
C	-0.4894	1.9206	2.1418	0.7603	-0.0727	-2.7365	0.1136	0.1710	1.3319
N	-2.4494	2.4273	3.5578	2.7263	1.0231	-3.7790	0.0266	-0.0874	-1.1362
N	0.3794	2.3307	3.2604	1.2926	0.2672	-1.3916	-1.3533	0.0639	1.3607
H	2.0636	4.4453	3.2369	2.4713	-1.5052	0.2511	-3.3930	1.6553	2.1344
H	-1.0741	1.7630	-0.4162	-0.3659	-1.2890	-4.7407	2.2130	1.4310	2.3189
H	4.3153	0.0354	3.0050	-2.1775	-0.4734	1.4805	-2.5605	-2.1973	5.1321
H	4.5408	4.3148	3.2731	1.5483	-2.4435	2.3212	-4.9066	1.2379	4.0233
H	1.9225	-1.9476	0.8977	-3.8402	0.6218	-1.8123	1.1724	-2.5900	4.9956

H	-0.5055	0.0026	-2.0691	-2.7412	-1.4133	-5.4269	3.7553	0.7548	4.1222
H	5.6734	2.1012	3.1460	-0.8351	-1.9961	2.9232	-4.4993	-0.6939	5.5584
H	0.9862	-1.8885	-1.3889	-4.5025	-0.4257	-3.9408	3.2395	-1.2972	5.4790
H	-4.2117	2.7577	4.6375	4.2823	2.0068	-4.7831	0.0279	-0.4970	-3.1955
H	-4.3469	1.5741	3.3208	2.7563	1.8865	-5.6932	1.5312	-0.9143	-2.3531
H	-3.5371	1.1155	4.8450	2.9143	3.0696	-4.3585	0.0968	-1.9484	-2.1687
H	0.4262	3.7372	4.8030	3.1698	0.4437	-0.4679	-2.9899	0.6894	0.2125
H	-0.4358	4.2924	3.3486	3.1031	-0.7799	-1.7554	-1.5722	1.7498	0.0989
H	-2.4910	1.1239	1.9352	0.8784	0.8001	-4.7125	1.6964	-0.5624	0.0345
H	-1.5810	0.4855	3.3311	0.8545	1.9841	-3.3808	0.3141	-1.6748	0.2464
H	-1.3242	2.1331	5.3398	3.0371	2.2605	-2.0822	-1.8430	-1.0962	-0.9776
H	-2.0616	3.7434	5.1254	4.3773	1.1719	-2.5028	-1.8333	0.3814	-1.9637
H	2.1800	-1.0808	3.0349	-2.3248	1.1523	-0.2019	-0.8027	-2.9613	3.7563
H	0.8031	-0.1646	3.7027	-0.8249	1.8265	-0.8536	-0.7742	-2.4195	2.0778
H	-0.7438	2.8150	1.5500	1.2064	-1.0313	-3.0494	0.3577	1.2248	1.1361

	**FΛ1**			**FV1**		
**atom**	**x**	**y**	**z**	**x**	**y**	**z**
O	-4.9115	-3.0311	-1.9585	5.1457	2.7783	1.8700
C	-3.4979	-3.2423	-1.7507	4.6738	1.4191	1.7650
C	-3.3216	-4.3352	-0.6920	5.5960	0.6419	0.8215
C	-4.0444	-3.9730	0.6097	5.7307	1.3364	-0.5390
C	-5.5111	-3.6813	0.3062	6.1270	2.7968	-0.3525
C	-5.6032	-2.6195	-0.8025	5.1655	3.4663	0.6415
H	-3.0411	-2.3168	-1.3823	3.6635	1.4158	1.3411
H	-3.7339	-5.2757	-1.0817	6.5894	0.5401	1.2794
H	-3.5868	-3.0532	0.9993	4.7412	1.3170	-1.0127
H	-6.0000	-4.6007	-0.0447	7.1483	2.8573	0.0482
H	-6.6457	-2.4731	-1.1012	5.4985	4.4848	0.8633
C	-2.8960	-3.6280	-3.0949	4.6542	0.8318	3.1696
O	-1.9369	-4.4916	-0.3690	5.0220	-0.6431	0.5719
O	-3.9553	-5.0264	1.5502	6.6905	0.6808	-1.3480
O	-6.2200	-3.1570	1.4225	6.0342	3.5513	-1.5567
O	-5.0494	-1.4256	-0.3000	3.8775	3.4728	0.0628
H	-1.9431	-4.1463	-2.9392	4.6308	-0.2640	3.1083
O	-2.7222	-2.5123	-3.9634	3.5681	1.3132	3.9543
H	-3.5876	-4.3143	-3.5965	5.5740	1.1349	3.6826
H	-3.0041	-5.2228	1.6876	6.3966	-0.2496	-1.4521
C	-6.7544	-4.1275	2.3264	7.1837	3.4975	-2.4049
C	-1.5562	-1.7564	-3.6471	2.3430	0.6524	3.6488
O	-6.0690	2.0067	-1.0435	1.4564	6.1981	0.4130
C	-5.4283	0.7428	-1.2923	2.1742	5.0193	0.8209
C	-5.8522	-0.2495	-0.2103	3.2477	4.7200	-0.2246
C	-5.5892	0.2946	1.1993	2.6327	4.5484	-1.6179
C	-6.0576	1.7385	1.3647	1.6533	5.6728	-1.9511
C	-5.6422	2.5994	0.1630	0.7181	5.9994	-0.7734
H	-4.3417	0.8641	-1.2311	1.4886	4.1656	0.8564
H	-6.9178	-0.4941	-0.3128	3.9860	5.5320	-0.2532
H	-4.4985	0.2772	1.3347	2.0707	3.6028	-1.6064
H	-7.1543	1.7500	1.4271	2.2297	6.5813	-2.1720
H	-6.1351	3.5747	0.2162	0.2031	6.9447	-0.9694
C	-5.8114	0.3006	-2.6953	2.7349	5.2756	2.2113
O	-6.2389	-0.5042	2.1745	3.6436	4.5234	-2.6149
O	-5.4977	2.3606	2.5173	0.8263	5.3363	-3.0621
O	-4.2407	2.7432	0.1938	-0.2069	4.9488	-0.6276
H	-5.5382	-0.7570	-2.8198	3.5048	4.5234	2.4309
O	-5.1943	1.0952	-3.7006	1.7270	5.2519	3.2149

H	-6.8941	0.4103	-2.8211	3.1873	6.2729	2.2356
H	-6.1141	-1.4448	1.9238	4.4037	4.0154	-2.2607
C	-6.1617	2.0842	3.7528	1.3660	5.6726	-4.3427
C	-3.8298	0.7439	-3.9093	1.3376	3.9261	3.5616
O	-2.4126	5.5161	-1.3814	-3.4939	5.1160	1.0103
C	-3.0079	4.2036	-1.3393	-2.0713	4.8882	0.9472
C	-3.6865	4.0448	0.0249	-1.5850	5.2797	-0.4510
C	-2.6635	4.2022	1.1561	-2.3308	4.4941	-1.5343
C	-1.9357	5.5354	1.0074	-3.8392	4.6409	-1.3503
C	-1.3942	5.6996	-0.4246	-4.2339	4.3298	0.1043
H	-2.2274	3.4399	-1.4425	-1.8596	3.8253	1.1152
H	-4.4649	4.8118	0.1346	-1.7479	6.3545	-0.6065
H	-1.9266	3.3925	1.0483	-2.0681	3.4396	-1.3926
H	-2.6478	6.3504	1.1961	-4.1259	5.6796	-1.5658
H	-1.0320	6.7228	-0.5609	-5.2841	4.5914	0.2634
C	-3.9539	4.1021	-2.5158	-1.4399	5.7054	2.0530
O	-3.2946	4.1463	2.4223	-1.9625	4.9429	-2.8246
O	-0.8196	5.6491	1.8854	-4.5876	3.7509	-2.1756
O	-0.3542	4.7709	-0.6153	-4.0203	2.9579	0.3348
H	-4.6005	3.2234	-2.4122	-0.3537	5.7556	1.9171
O	-3.1740	4.0041	-3.7009	-1.7566	5.0932	3.2964
H	-4.5897	5.0028	-2.5444	-1.8452	6.7303	2.0127
H	-3.9354	3.4062	2.4100	-0.9840	4.9569	-2.8658
C	-1.1156	6.1259	3.2024	-4.8135	4.1938	-3.5180
C	-3.9877	4.0429	-4.8645	-1.3495	5.8977	4.3940
O	2.5956	4.5913	-2.7649	-5.3479	0.4810	2.6711
C	1.2725	4.2455	-2.3027	-4.3788	1.4001	2.1242
C	0.8475	5.2457	-1.2264	-5.0408	2.2269	1.0197
C	1.8828	5.3897	-0.1086	-5.7448	1.3618	-0.0304
C	3.2808	5.6074	-0.6705	-6.6503	0.3283	0.6284
C	3.5713	4.5554	-1.7528	-5.8689	-0.4179	1.7231
H	1.2885	3.2413	-1.8658	-3.5464	0.8366	1.6891
H	0.6832	6.2266	-1.6928	-5.7718	2.9129	1.4689
H	1.8967	4.4416	0.4446	-4.9685	0.8122	-0.5712
H	3.3532	6.6070	-1.1208	-7.5152	0.8253	1.0892
H	4.5317	4.7739	-2.2292	-6.5416	-1.0921	2.2618
C	0.3322	4.2813	-3.5023	-3.8790	2.2856	3.2609
O	1.5351	6.4688	0.7453	-6.4965	2.1794	-0.9131
O	4.2937	5.4406	0.3164	-7.0976	-0.6743	-0.2804
O	3.5807	3.2721	-1.1598	-4.8094	-1.1355	1.1226
H	-0.6989	4.4249	-3.1528	-3.4761	3.2192	2.8465
O	0.4232	3.1212	-4.3205	-2.9109	1.6488	4.0856
H	0.6108	5.1356	-4.1296	-4.7325	2.5293	3.9035
H	0.6903	6.2224	1.1816	-5.8538	2.7405	-1.3958
C	4.5587	6.5875	1.1283	-8.2219	-0.3147	-1.0893
C	-0.3091	2.0226	-3.7868	-1.6113	1.6567	3.5037
O	6.1494	0.7230	-1.6916	-3.8452	-4.5866	1.8525
C	5.0108	1.5404	-2.0230	-3.9771	-3.1738	2.0964
C	4.8252	2.6024	-0.9406	-4.8747	-2.5614	1.0241
C	4.7003	1.9748	0.4570	-4.3871	-2.8827	-0.3962
C	5.7547	0.8917	0.7064	-3.9363	-4.3360	-0.5700
C	5.9414	-0.0266	-0.5140	-3.1623	-4.8654	0.6491
H	4.1072	0.9211	-2.0266	-2.9948	-2.6949	2.0165
H	5.6576	3.3162	-0.9421	-5.9077	-2.9130	1.1374
H	3.7151	1.4835	0.4957	-3.5058	-2.2478	-0.5779
H	6.7162	1.3867	0.9047	-4.8320	-4.9614	-0.6886

H	6.8393	-0.6353	-0.3754	-3.0987	-5.9553	0.5842
C	5.2251	2.0833	-3.4396	-4.4950	-2.9976	3.5269
O	4.8266	2.9620	1.4648	-5.4127	-2.6182	-1.3410
O	5.4083	0.0380	1.7947	-3.0720	-4.4928	-1.6950
O	4.8026	-0.8414	-0.6437	-1.8778	-4.2935	0.6620
H	4.9429	3.1422	-3.4990	-5.2459	-2.1978	3.5688
O	4.4986	1.3426	-4.4115	-3.4511	-2.7175	4.4488
H	6.2866	1.9958	-3.6906	-4.9691	-3.9320	3.8430
H	4.3950	3.7799	1.1476	-5.9264	-1.8480	-1.0187
C	5.5944	0.5847	3.1051	-3.7208	-4.5657	-2.9689
C	3.1264	1.7283	-4.4399	-3.0734	-1.3439	4.3992
O	4.1212	-4.0737	-2.1961	1.2268	-5.4034	2.2406
C	4.0171	-2.6482	-2.0180	0.0981	-4.5310	2.0377
C	5.0273	-2.2152	-0.9573	-0.7972	-5.1698	0.9802
C	4.8349	-2.9928	0.3511	-0.0338	-5.4116	-0.3274
C	4.8110	-4.4972	0.0910	1.2646	-6.1682	-0.0656
C	3.8025	-4.7969	-1.0293	2.0391	-5.5202	1.0950
H	3.0158	-2.3991	-1.6520	0.4459	-3.5636	1.6603
H	6.0466	-2.3685	-1.3363	-1.1726	-6.1274	1.3646
H	3.8530	-2.7040	0.7415	0.2274	-4.4286	-0.7366
H	5.8062	-4.8347	-0.2304	1.0297	-7.2028	0.2191
H	3.8343	-5.8564	-1.3001	2.8860	-6.1516	1.3799
C	4.2460	-2.0032	-3.3753	-0.5795	-4.3457	3.3852
O	5.8573	-2.6659	1.2752	-0.8385	-6.1405	-1.2374
O	4.3812	-5.2641	1.2127	2.1370	-6.1600	-1.1927
O	2.5264	-4.4361	-0.5482	2.4790	-4.2524	0.6661
H	4.3530	-0.9179	-3.2494	-1.5435	-3.8418	3.2442
O	3.2137	-2.2941	-4.3121	0.2233	-3.6261	4.3155
H	5.1718	-2.3994	-3.8063	-0.7603	-5.3293	3.8318
H	5.6947	-1.7392	1.5524	-1.5832	-5.5534	-1.4847
C	5.3786	-5.5115	2.2082	1.9251	-7.2253	-2.1241
C	1.9606	-1.7172	-3.9669	0.4238	-2.2658	3.9522
O	-0.6389	-5.9138	-1.6780	5.4069	-2.4518	1.9866
C	0.4798	-5.0100	-1.7490	4.0332	-2.8874	1.9753
C	1.4529	-5.3686	-0.6235	3.8493	-3.8981	0.8358
C	0.7692	-5.2874	0.7433	4.2469	-3.2580	-0.4911
C	-0.5296	-6.0864	0.7497	5.6687	-2.7196	-0.4121
C	-1.3919	-5.7843	-0.4900	5.8060	-1.7871	0.8037
H	0.1321	-3.9790	-1.6077	3.3768	-2.0279	1.7896
H	1.8345	-6.3869	-0.7768	4.4594	-4.7916	1.0239
H	0.5114	-4.2313	0.9172	3.5734	-2.4033	-0.6369
H	-0.2811	-7.1560	0.7217	6.3692	-3.5547	-0.2786
H	-2.1865	-6.5329	-0.5613	6.8562	-1.5183	0.9515
C	1.1006	-5.1404	-3.1237	3.7194	-3.4641	3.3386
O	1.6216	-5.7812	1.7615	4.1446	-4.1674	-1.5716
O	-1.3180	-5.7966	1.9009	6.0118	-1.9775	-1.5794
H	2.0683	-4.6225	-3.1369	2.7467	-3.9717	3.3052
O	0.2227	-4.5619	-4.0764	3.6893	-2.4029	4.2812
H	1.2714	-6.2064	-3.3483	4.4913	-4.2032	3.6104
H	2.5327	-5.4838	1.5676	3.3534	-4.7263	-1.4336
C	-1.0458	-6.6156	3.0426	6.6766	-2.7317	-2.5975
C	0.7793	-4.5874	-5.3833	3.3581	-2.8703	5.5816
H	-1.4689	-0.9711	-4.4036	1.5918	1.0324	4.3474
H	-1.6311	-1.2778	-2.6591	2.0033	0.8677	2.6246
H	-0.6695	-2.3991	-3.6712	2.4538	-0.4312	3.7710
H	-3.4722	1.3346	-4.7548	0.6380	4.0053	4.3956

H	-3.2062	0.9831	-3.0365	0.8248	3.4192	2.7322
H	-3.7351	-0.3279	-4.1260	2.2113	3.3274	3.8517
H	-3.3264	3.9127	-5.7257	-1.5840	5.3419	5.3063
H	-4.7364	3.2392	-4.8494	-0.2715	6.1038	4.3546
H	-4.5073	5.0105	-4.9495	-1.8941	6.8552	4.4030
H	-0.2063	1.1953	-4.4953	-0.9374	1.1770	4.2198
H	0.0893	1.6971	-2.8137	-1.5789	1.0870	2.5624
H	-1.3689	2.2860	-3.6688	-1.2814	2.6863	3.3122
H	2.6390	1.1357	-5.2178	-2.2909	-1.1946	5.1469
H	2.6264	1.5386	-3.4796	-2.6820	-1.0613	3.4116
H	3.0152	2.7975	-4.6521	-3.9253	-0.6869	4.6064
H	1.3150	-1.8058	-4.8433	0.8333	-1.7575	4.8276
H	1.4818	-2.2483	-3.1342	1.1438	-2.1639	3.1301
H	2.0686	-0.6573	-3.6989	-0.5209	-1.7876	3.6600
H	0.0726	-4.0775	-6.0436	3.3056	-1.9951	6.2350
H	1.7475	-4.0674	-5.4073	2.3878	-3.3866	5.5779
H	0.9218	-5.6219	-5.7325	4.1275	-3.5609	5.9609
H	-7.3503	-3.5725	3.0552	6.9944	4.1964	-3.2235
H	-7.4010	-4.8331	1.7871	8.0787	3.8161	-1.8526
H	-5.9535	-4.6774	2.8272	7.3373	2.4870	-2.7927
H	-5.7073	2.7408	4.4989	0.5932	5.4305	-5.0764
H	-7.2335	2.3098	3.6692	1.5954	6.7461	-4.3935
H	-6.0339	1.0370	4.0404	2.2744	5.0980	-4.5446
H	-0.1556	6.2057	3.7166	-5.4858	3.4614	-3.9712
H	-1.5982	7.1115	3.1534	-5.2905	5.1833	-3.5200
H	-1.7666	5.4229	3.7269	-3.8722	4.2402	-4.0701
H	5.4081	6.3226	1.7630	-8.5029	-1.2154	-1.6409
H	4.8270	7.4456	0.4968	-9.0594	0.0014	-0.4528
H	3.6904	6.8457	1.7401	-7.9645	0.4929	-1.7789
H	5.4159	-0.2383	3.8007	-2.9305	-4.7492	-3.7009
H	6.6193	0.9637	3.2228	-4.4492	-5.3887	-2.9834
H	4.8852	1.3951	3.2950	-4.2303	-3.6259	-3.1964
H	4.9361	-6.2145	2.9181	2.7007	-7.1271	-2.8875
H	6.2684	-5.9637	1.7496	2.0258	-8.1964	-1.6207
H	5.6628	-4.5843	2.7127	0.9328	-7.1489	-2.5779
H	-1.7853	-6.3430	3.7993	6.9262	-2.0234	-3.3911
H	-1.1550	-7.6791	2.7894	7.5982	-3.1820	-2.2040
H	-0.0345	-6.4317	3.4153	6.0195	-3.5162	-2.9823
C	0.7730	3.0650	2.9151	1.4436	1.2867	-3.0612
C	1.8475	-2.1601	2.7715	-1.2247	-2.8188	-5.2666
C	2.7414	2.6710	4.8517	3.0006	-1.0060	-2.7492
C	1.7563	4.0372	3.1286	2.8367	1.3639	-3.1726
C	3.2165	-1.4740	5.0757	0.4646	-4.3030	-3.6628
C	2.9617	-2.9052	3.1572	-0.9363	-4.1387	-5.6124
C	2.7541	3.8380	4.0820	3.6175	0.2146	-3.0409
C	3.6554	-2.5584	4.3168	-0.0994	-4.8968	-4.7909
C	-3.4022	-1.8863	3.5282	-4.8190	0.2189	-3.1654
C	-1.5432	1.3411	3.0364	-1.3013	1.1423	-2.3197
C	-1.0632	-1.4033	3.5069	-2.7465	-0.9712	-3.5035
C	-2.6382	0.3970	3.5215	-2.7708	0.8716	-2.0359
C	1.6819	0.4684	5.5281	0.9343	-2.3597	-2.1522
C	0.7638	1.8860	3.6766	0.8199	0.0594	-2.7843
C	1.3856	-1.0789	3.5357	-0.7025	-2.2228	-4.1074
C	1.7469	1.7096	4.6764	1.6183	-1.0832	-2.5732
C	2.0834	-0.7372	4.7083	0.1852	-2.9746	-3.3152
C	0.0732	-0.4480	3.0824	-1.2342	-0.8217	-3.7866

N	-2.3778	-0.9626	3.0458	-3.3665	0.3182	-3.2446
N	-0.2542	0.9071	3.5764	-0.5905	-0.0975	-2.6592
H	0.0100	3.2469	2.1678	0.8570	2.1905	-3.1847
H	1.3165	-2.4260	1.8607	-1.8801	-2.2343	-5.9073
H	3.4931	2.5119	5.6209	3.6003	-1.9018	-2.6357
H	1.7413	4.9502	2.5462	3.2966	2.3288	-3.3590
H	3.7546	-1.1841	5.9746	1.1428	-4.8640	-3.0296
H	3.2841	-3.7435	2.5565	-1.3628	-4.5675	-6.5145
H	3.5253	4.5871	4.2341	4.6996	0.2695	-3.1165
H	4.5302	-3.1245	4.6230	0.1317	-5.9293	-5.0368
H	-4.3850	-1.5448	3.1968	-5.2389	1.1981	-2.9201
H	-3.2229	-2.8904	3.1281	-5.2133	-0.1045	-4.1363
H	-3.4086	-1.9511	4.6343	-5.1548	-0.5013	-2.4058
H	-1.7579	2.3569	3.3753	-0.8104	1.5780	-1.4432
H	-1.5408	1.3476	1.9314	-1.2572	1.8848	-3.1349
H	-0.8637	-2.3969	3.0912	-3.2205	-1.4242	-4.3781
H	-1.0328	-1.4854	4.6120	-2.8764	-1.6694	-2.6546
H	-2.6678	0.4328	4.6285	-2.8648	0.1758	-1.1774
H	-3.6077	0.7355	3.1454	-3.2793	1.7997	-1.7776
H	2.3444	0.5649	6.3934	1.6720	-3.0736	-1.7768
H	0.6517	0.3451	5.8872	0.2344	-2.1278	-1.3387
H	0.0825	-0.4250	1.9774	-1.1428	-0.2065	-4.6998

	**CR2**			**NR2**			**M2**		
**atom**	**x**	**y**	**z**	**x**	**y**	**z**	**x**	**y**	**z**
O	3.5051	-3.9089	0.3804	-4.6083	1.8644	-2.6496	4.3814	3.0220	-2.9908
C	2.0722	-3.8504	0.2294	-4.1947	0.5822	-2.1318	3.3178	3.2834	-2.0519
C	1.5976	-5.1571	-0.4004	-5.4493	-0.2257	-1.7951	3.7372	4.4507	-1.1534
C	2.3066	-5.4335	-1.7334	-6.3886	0.5201	-0.8400	5.0904	4.2108	-0.4734
C	3.8222	-5.3093	-1.5853	-6.6805	1.9217	-1.3661	6.1389	3.8079	-1.5061
C	4.1880	-4.0105	-0.8477	-5.3665	2.6274	-1.7392	5.6045	2.6692	-2.3894
H	1.8110	-3.0268	-0.4446	-3.6110	0.7296	-1.2162	3.1500	2.3952	-1.4320
H	1.7883	-5.9883	0.2914	-5.9903	-0.4446	-2.7256	3.8102	5.3600	-1.7653
H	1.9786	-4.6574	-2.4402	-5.8647	0.6265	0.1209	4.9661	3.3699	0.2250
H	4.1983	-6.1594	-0.9990	-7.3071	1.8493	-2.2661	6.3607	4.6707	-2.1496
H	5.2554	-4.0050	-0.6076	-5.5825	3.5753	-2.2409	6.3027	2.4767	-3.2096
C	1.4727	-3.5789	1.6042	-3.3385	-0.0960	-3.1982	2.0715	3.6129	-2.8635
O	0.2007	-5.0389	-0.6774	-5.0698	-1.4347	-1.1364	2.7828	4.6284	-0.1034
O	1.9802	-6.7289	-2.2048	-7.6000	-0.1992	-0.6883	5.5138	5.3832	0.1982
O	4.4960	-5.2432	-2.8375	-7.3083	2.7589	-0.4011	7.3388	3.3186	-0.9190
O	3.8552	-2.9219	-1.6814	-4.6349	2.8433	-0.5516	5.4492	1.5190	-1.5842
H	0.4620	-4.0052	1.6636	-3.3867	-1.1848	-3.0780	1.3362	4.1116	-2.2211
O	1.4509	-2.1855	1.8919	-1.9814	0.3431	-3.1852	1.4951	2.4747	-3.4948
H	2.0956	-4.0562	2.3675	-3.7454	0.1637	-4.1814	2.3609	4.3030	-3.6643
H	1.0235	-6.7192	-2.4219	-7.3764	-1.0549	-0.2652	4.8227	5.6108	0.8551
C	4.7682	-6.5053	-3.4503	-8.7285	2.6237	-0.3115	8.2844	4.3200	-0.5364
C	0.3202	-1.5416	1.3262	-1.2165	-0.2925	-2.1632	0.7118	1.6927	-2.5986
O	5.8394	0.1632	-1.8055	-2.5617	5.8100	0.0362	6.0527	-1.9037	-2.7646
C	4.9205	-0.7811	-1.2265	-2.9111	4.5449	-0.5529	5.3191	-0.6741	-2.6183
C	4.8892	-2.0339	-2.1038	-4.3425	4.1838	-0.1560	6.2044	0.3493	-1.9034
C	4.5437	-1.7013	-3.5607	-4.5343	4.1989	1.3658	6.7158	-0.1870	-0.5599
C	5.3985	-0.5450	-4.0830	-3.9681	5.4796	1.9829	7.3197	-1.5827	-0.7233
C	5.4280	0.6167	-3.0761	-2.5564	5.7664	1.4466	6.3822	-2.4961	-1.5269
H	3.9129	-0.3524	-1.2127	-2.2527	3.7643	-0.1582	4.4327	-0.8477	-1.9980
H	5.8646	-2.5375	-2.0786	-5.0550	4.8853	-0.6093	7.0675	0.6061	-2.5317
H	3.4943	-1.3685	-3.5760	-3.9544	3.3558	1.7718	5.8432	-0.2797	0.1052
H	6.4294	-0.9012	-4.2172	-4.6138	6.3240	1.7044	8.2631	-1.4966	-1.2797

H	6.1676	1.3593	-3.3903	-2.2227	6.7527	1.7829	6.8918	-3.4348	-1.7651
C	5.3835	-1.0520	0.1977	-2.7098	4.6705	-2.0555	4.8880	-0.2403	-4.0112
O	4.7362	-2.8392	-4.3846	-5.9085	4.0852	1.6941	7.6935	0.6780	-0.0106
O	4.9075	-0.0020	-5.3025	-3.8504	5.4008	3.3985	7.5456	-2.2264	0.5258
O	4.1390	1.1857	-3.0217	-1.6985	4.7560	1.9233	5.2331	-2.7319	-0.7485
H	4.8263	-1.9121	0.5924	-3.2054	3.8297	-2.5578	4.5635	0.8083	-3.9795
O	5.2184	0.0691	1.0561	-1.3337	4.7054	-2.4142	3.8474	-1.0622	-4.5286
H	6.4534	-1.2873	0.1852	-3.1547	5.6108	-2.3982	5.7413	-0.3334	-4.6918
H	4.4244	-3.6223	-3.8885	-6.3101	3.4410	1.0770	7.4061	1.5996	-0.1715
C	5.3058	-0.6991	-6.4844	-5.0380	5.7211	4.1255	8.8090	-1.9466	1.1319
C	3.8566	0.2892	1.4118	-0.7309	3.4182	-2.3556	2.5715	-0.6936	-4.0154
O	3.3941	4.6431	-1.9993	1.9542	4.9988	2.2647	2.8623	-5.5190	-1.0866
C	3.6616	3.2437	-1.7867	0.7204	4.7326	1.5682	3.3886	-4.2048	-1.3586
C	4.0034	2.6015	-3.1364	-0.4407	5.1214	2.4885	4.7343	-4.0643	-0.6417
C	2.8916	2.8234	-4.1670	-0.3691	4.3729	3.8258	4.6049	-4.3329	0.8626
C	2.5441	4.3073	-4.2501	1.0096	4.5419	4.4587	3.9081	-5.6685	1.1034
C	2.2932	4.8878	-2.8491	2.1197	4.2365	3.4411	2.6134	-5.7606	0.2809
H	2.7713	2.7586	-1.3695	0.6522	3.6624	1.3383	2.6976	-3.4441	-0.9758
H	4.9314	3.0453	-3.5216	-0.4056	6.2017	2.6832	5.4506	-4.7796	-1.0679
H	1.9978	2.2858	-3.8151	-0.5127	3.3025	3.6118	3.9694	-3.5403	1.2864
H	3.3977	4.8464	-4.6832	1.1271	5.5882	4.7728	4.5708	-6.4792	0.7711
H	2.2028	5.9758	-2.9171	3.0856	4.5375	3.8575	2.2188	-6.7794	0.3379
C	4.8085	3.1420	-0.8038	0.7528	5.5239	0.2760	3.5022	-4.0728	-2.8628
O	3.3015	2.3664	-5.4429	-1.3564	4.8590	4.7168	5.8827	-4.3525	1.4718
O	1.3718	4.5428	-5.0258	1.2075	3.6717	5.5696	3.5535	-5.8553	2.4712
O	1.1119	4.3207	-2.3323	2.1047	2.8549	3.1677	1.6881	-4.8376	0.8045
H	5.1128	2.0926	-0.7063	-0.2276	5.4832	-0.2135	4.1010	-3.1927	-3.1245
O	4.3949	3.6578	0.4525	1.7541	4.9687	-0.5634	2.1936	-3.9589	-3.4049
H	5.6667	3.7180	-1.1902	0.9840	6.5778	0.5060	4.0078	-4.9698	-3.2582
H	3.6937	1.4774	-5.3293	-2.2082	4.8759	4.2359	6.3520	-3.5371	1.2021
C	1.6125	4.7565	-6.4204	0.8041	4.2126	6.8308	4.5563	-6.4966	3.2648
C	5.4268	3.5274	1.4204	1.9107	5.7018	-1.7724	2.2093	-4.0070	-4.8240
O	-1.3057	5.3430	0.2248	4.6923	0.6689	1.7604	-1.9629	-4.6414	0.5619
C	-0.2021	4.6057	-0.3394	3.4796	1.4492	1.7627	-0.5984	-4.2745	0.2665
C	0.1784	5.1987	-1.6990	3.3649	2.1849	3.0971	0.3357	-5.2753	0.9492
C	-1.0159	5.3535	-2.6433	3.4309	1.2251	4.2862	0.0678	-5.3944	2.4523
C	-2.1382	6.0981	-1.9284	4.6806	0.3562	4.1701	-1.4129	-5.6679	2.6969
C	-2.4545	5.4069	-0.5905	4.7523	-0.3040	2.7793	-2.2729	-4.6443	1.9340
H	-0.4961	3.5587	-0.4789	2.6163	0.7830	1.6494	-0.3977	-3.2752	0.6677
H	0.6268	6.1896	-1.5427	4.1899	2.9054	3.1789	0.1975	-6.2623	0.4875
H	-1.3825	4.3476	-2.8928	2.5525	0.5647	4.2356	0.3109	-4.4253	2.9120
H	-1.8128	7.1254	-1.7126	5.5693	0.9928	4.2835	-1.6621	-6.6731	2.3289
H	-3.1852	5.9995	-0.0319	5.7186	-0.8037	2.6610	-3.3286	-4.9214	2.0123
C	0.9644	4.7160	0.6410	3.5587	2.4144	0.5900	-0.4204	-4.2880	-1.2503
O	-0.6274	6.0629	-3.8063	3.4603	1.9514	5.5022	0.8514	-6.4375	3.0052
O	-3.3501	6.1217	-2.6697	4.7140	-0.6936	5.1268	-1.7845	-5.5474	4.0648
O	-2.9625	4.1175	-0.8642	3.6944	-1.2300	2.6625	-2.0523	-3.3598	2.4859
H	1.9123	4.6076	0.0980	2.9270	3.2888	0.7737	0.6336	-4.4598	-1.4958
O	0.8978	3.7731	1.7036	3.2093	1.7981	-0.6400	-0.8898	-3.1070	-1.8886
H	0.9320	5.7129	1.0935	4.5971	2.7501	0.4905	-1.0102	-5.1185	-1.6536
H	0.0348	5.5091	-4.2712	2.6419	2.4891	5.5378	1.7915	-6.1949	2.8706
C	-3.4692	7.1854	-3.6170	5.2828	-0.3507	6.3923	-1.5571	-6.7094	4.8670
C	1.3811	2.4955	1.3032	1.8062	1.6845	-0.8364	-0.0052	-2.0026	-1.7334
O	-5.3835	2.1960	1.1244	3.5781	-4.2882	0.6132	-4.6232	-0.8236	3.1325
C	-4.0881	2.7014	0.7470	3.4862	-2.8715	0.8712	-3.7357	-1.6458	2.3519
C	-4.2497	3.7513	-0.3564	3.9907	-2.5807	2.2929	-3.0991	-2.7262	3.2300

C	-5.0429	3.2001	-1.5487	3.3244	-3.4629	3.3569	-2.4203	-2.1174	4.4653
C	-6.3421	2.5502	-1.0725	3.3778	-4.9304	2.9412	-3.3493	-1.1288	5.1722
C	-6.0724	1.5628	0.0729	2.8524	-5.0933	1.5090	-3.9650	-0.1388	4.1754
H	-3.4774	1.8812	0.3523	2.4415	-2.5499	0.7862	-2.9159	-1.0286	1.9665
H	-4.7736	4.6322	0.0381	5.0743	-2.7581	2.3326	-3.8448	-3.4626	3.5547
H	-4.4273	2.4152	-2.0151	2.2654	-3.1692	3.4148	-1.5512	-1.5472	4.1017
H	-7.0135	3.3349	-0.6971	4.4202	-5.2764	2.9722	-4.1719	-1.6866	5.6396
H	-7.0208	1.2163	0.4945	2.9944	-6.1257	1.1747	-4.7317	0.4601	4.6761
C	-3.4469	3.2589	2.0093	4.3510	-2.1762	-0.1749	-4.5458	-2.1683	1.1619
O	-5.3576	4.2276	-2.4679	3.9738	-3.3140	4.6050	-2.0291	-3.1175	5.3878
O	-6.9925	1.8043	-2.0936	2.5655	-5.7679	3.7557	-2.6590	-0.3560	6.1515
O	-5.3261	0.4772	-0.4371	1.4806	-4.7491	1.4947	-2.9345	0.6803	3.6786
H	-2.6324	3.9425	1.7351	4.4707	-1.1217	0.1033	-4.3190	-3.2247	0.9671
O	-2.9627	2.2219	2.8528	3.8362	-2.2968	-1.4912	-4.2936	-1.3999	-0.0072
H	-4.2039	3.8136	2.5741	5.3401	-2.6487	-0.1715	-5.6112	-2.0792	1.3941
H	-4.5476	4.7571	-2.6196	4.0667	-2.3557	4.7846	-1.7400	-3.9027	4.8793
C	-7.8394	2.5570	-2.9646	3.1658	-6.2142	4.9739	-2.6579	-0.9191	7.4657
C	-1.6700	1.7847	2.4505	2.8972	-1.2726	-1.8077	-3.0980	-1.8416	-0.6514
O	-5.6205	-2.9816	0.7653	-1.0928	-6.4245	-0.5165	-3.1289	3.9359	2.0164
C	-5.1358	-1.6257	0.7816	0.0149	-5.5439	-0.2539	-2.9371	2.5100	2.0858
C	-5.8605	-0.8418	-0.3162	0.5186	-5.7575	1.1754	-3.2065	2.0730	3.5238
C	-5.6621	-1.4990	-1.6888	-0.6127	-5.5978	2.1990	-2.2632	2.7971	4.4946
C	-5.9985	-2.9891	-1.6284	-1.8331	-6.4235	1.7915	-2.3217	4.3093	4.2802
C	-5.3140	-3.6641	-0.4308	-2.2002	-6.1775	0.3216	-2.1876	4.6528	2.7852
H	-4.0615	-1.6169	0.5637	-0.3198	-4.5036	-0.3331	-1.8941	2.2710	1.8529
H	-6.9355	-0.8037	-0.0953	0.9561	-6.7587	1.2838	-4.2475	2.2993	3.7891
H	-4.5944	-1.4071	-1.9424	-0.9116	-4.5381	2.1864	-1.2402	2.4666	4.2593
H	-7.0836	-3.0979	-1.4961	-1.5925	-7.4895	1.9012	-3.2986	4.6787	4.6228
H	-5.7024	-4.6797	-0.3076	-2.9783	-6.8821	0.0123	-2.4092	5.7126	2.6279
C	-5.3743	-1.0868	2.1834	1.0674	-5.8200	-1.3175	-3.8417	1.8691	1.0460
O	-6.4804	-0.8874	-2.6683	-0.1981	-6.0009	3.4909	-2.6068	2.4881	5.8337
O	-5.5761	-3.6836	-2.7972	-2.9812	-6.0973	2.5696	-1.2756	5.0034	4.9485
O	-3.9267	-3.6838	-0.6693	-2.6491	-4.8490	0.1998	-0.8719	4.3509	2.3816
H	-5.2188	0.0005	2.1850	2.0410	-5.4467	-0.9725	-3.9346	0.7973	1.2513
O	-4.5315	-1.7193	3.1419	0.7359	-5.2254	-2.5663	-3.3703	2.0671	-0.2857
H	-6.4085	-1.3020	2.4747	1.1329	-6.9015	-1.4780	-4.8347	2.3272	1.0939
H	-6.4506	0.0808	-2.5276	0.7272	-5.7068	3.6181	-2.5455	1.5156	5.9318
C	-6.5395	-3.7328	-3.8517	-3.1464	-6.8802	3.7541	-1.5489	5.3406	6.3100
C	-3.2323	-1.1396	3.1851	1.0326	-3.8310	-2.5766	-2.1134	1.4413	-0.5354
O	-1.3682	-5.8581	0.8357	-4.6905	-3.3211	-2.4397	0.8524	5.8702	-0.4926
C	-2.2290	-4.7060	0.7453	-3.4675	-3.6667	-1.7592	-0.0101	4.9199	0.1621
C	-3.2077	-4.8916	-0.4222	-3.8080	-4.5973	-0.5933	-0.1409	5.3222	1.6348
C	-2.5066	-5.2389	-1.7379	-4.8385	-3.9705	0.3533	1.2296	5.4049	2.3146
C	-1.5605	-6.4132	-1.5222	-6.0546	-3.4967	-0.4346	2.1684	6.2939	1.5045
C	-0.6272	-6.1259	-0.3354	-5.6178	-2.6342	-1.6293	2.1670	5.8885	0.0209
H	-1.6265	-3.8056	0.5752	-2.9928	-2.7588	-1.3678	0.4318	3.9175	0.1001
H	-3.9010	-5.7086	-0.1793	-4.2177	-5.5362	-0.9893	-0.6281	6.3044	1.7009
H	-1.9035	-4.3698	-2.0399	-4.3713	-3.0879	0.8154	1.6542	4.3892	2.3282
H	-2.1463	-7.3097	-1.2781	-6.5848	-4.3724	-0.8334	1.8093	7.3304	1.5624
H	-0.0288	-7.0148	-0.1152	-6.4833	-2.4320	-2.2672	2.7048	6.6425	-0.5616
C	-2.9799	-4.6032	2.0567	-2.5623	-4.3106	-2.7879	-1.3318	4.9294	-0.5748
O	-3.4621	-5.5750	-2.7269	-5.2445	-4.9116	1.3299	1.1025	5.9297	3.6235
O	-0.7434	-6.6593	-2.6642	-6.9390	-2.7079	0.3575	3.5126	6.2151	1.9684
H	-3.7764	-3.8543	1.9730	-1.7225	-4.8217	-2.3036	-2.0747	4.3514	-0.0120
O	-2.0903	-4.2559	3.1086	-2.0915	-3.2948	-3.6665	-1.1311	4.3590	-1.8589
H	-3.4445	-5.5828	2.2603	-3.1484	-5.0597	-3.3450	-1.6904	5.9685	-0.6637

H	-4.1096	-4.8420	-2.7736	-4.4382	-5.2297	1.7859	0.3705	5.4538	4.0645
C	-1.2931	-7.5913	-3.6014	-7.9562	-3.4521	1.0349	3.8588	7.1792	2.9668
C	-2.7515	-4.3340	4.3665	-1.5473	-3.8377	-4.8590	-2.3385	4.3095	-2.6048
H	0.4081	-0.4745	1.5445	-0.1952	0.0836	-2.2481	0.2397	0.9038	-3.1911
H	0.2704	-1.6753	0.2353	-1.6024	-0.0584	-1.1604	1.3269	1.2179	-1.8193
H	-0.6089	-1.9321	1.7684	-1.2164	-1.3811	-2.3089	-0.0574	2.3112	-2.1248
H	3.8509	1.0560	2.1885	0.2958	3.5362	-2.7020	1.8328	-1.3465	-4.4839
H	3.2707	0.6469	0.5547	-0.7029	3.0260	-1.3304	2.5133	-0.8344	-2.9275
H	3.3853	-0.6299	1.7886	-1.2666	2.6913	-2.9826	2.3508	0.3578	-4.2401
H	5.0509	3.9484	2.3561	2.7199	5.2242	-2.3337	1.1802	-3.8577	-5.1632
H	5.6938	2.4721	1.5683	0.9809	5.6945	-2.3589	2.8521	-3.2188	-5.2390
H	6.3272	4.0827	1.1138	2.1825	6.7487	-1.5610	2.5707	-4.9844	-5.1810
H	1.3671	1.8529	2.1863	1.6632	1.3247	-1.8594	-0.4921	-1.1459	-2.2031
H	0.7461	2.0429	0.5266	1.3527	0.9608	-0.1435	0.1700	-1.7629	-0.6745
H	2.4068	2.5828	0.9336	1.3297	2.6615	-0.7175	0.9540	-2.2129	-2.2213
H	-1.3728	0.9973	3.1415	2.5894	-1.4226	-2.8443	-2.9085	-1.1832	-1.4992
H	-1.6786	1.3734	1.4294	2.0077	-1.3254	-1.1604	-2.2407	-1.8133	0.0333
H	-0.9526	2.6068	2.5105	3.3612	-0.2888	-1.6982	-3.2052	-2.8764	-0.9913
H	-2.6601	-1.6761	3.9436	0.8951	-3.4727	-3.5954	-2.0531	1.2329	-1.6058
H	-2.7082	-1.2379	2.2232	0.3598	-3.2618	-1.9191	-1.2807	2.0952	-0.2484
H	-3.2983	-0.0741	3.4367	2.0671	-3.6567	-2.2588	-2.0302	0.4923	0.0076
H	-2.0410	-4.0047	5.1284	-1.1978	-2.9981	-5.4651	-2.1187	3.7862	-3.5393
H	-3.6370	-3.6841	4.3847	-0.7080	-4.5138	-4.6443	-3.1139	3.7636	-2.0531
H	-3.0640	-5.3682	4.5798	-2.3119	-4.3961	-5.4216	-2.7049	5.3226	-2.8336
H	5.3703	-6.2934	-4.3376	-9.0666	3.3873	0.3935	9.1764	3.7872	-0.1973
H	5.3407	-7.1467	-2.7656	-9.1910	2.8019	-1.2923	8.5415	4.9503	-1.3991
H	3.8417	-7.0146	-3.7299	-9.0063	1.6269	0.0424	7.8873	4.9493	0.2649
H	4.9597	-0.0980	-7.3291	-4.7616	5.7268	5.1829	8.8732	-2.5796	2.0204
H	6.4003	-0.7899	-6.5254	-5.4063	6.7164	3.8394	9.6294	-2.1982	0.4450
H	4.8609	-1.6978	-6.5214	-5.8224	4.9811	3.9418	8.8828	-0.8916	1.4114
H	0.6446	4.9957	-6.8676	1.0841	3.4754	7.5874	4.1213	-6.6394	4.2569
H	2.3024	5.5989	-6.5688	1.3269	5.1583	7.0313	4.8210	-7.4741	2.8376
H	2.0317	3.8568	-6.8790	-0.2760	4.3836	6.8485	5.4522	-5.8727	3.3287
H	-4.4814	7.1230	-4.0244	5.3290	-1.2766	6.9709	-1.9746	-6.4880	5.8524
H	-3.3358	8.1557	-3.1187	6.2984	0.0476	6.2595	-2.0771	-7.5756	4.4353
H	-2.7289	7.0818	-4.4148	4.6665	0.3909	6.9081	-0.4889	-6.9292	4.9441
H	-8.3555	1.8290	-3.5957	2.4680	-6.9306	5.4149	-2.1624	-0.1921	8.1140
H	-8.5788	3.1234	-2.3816	4.1210	-6.7155	4.7655	-3.6863	-1.0808	7.8184
H	-7.2568	3.2494	-3.5785	3.3379	-5.3781	5.6569	-2.1162	-1.8693	7.4784
H	-6.1091	-4.3610	-4.6355	-4.0950	-6.5713	4.2002	-0.7022	5.9380	6.6573
H	-7.4770	-4.1836	-3.4968	-3.1909	-7.9503	3.5070	-2.4692	5.9377	6.3803
H	-6.7431	-2.7300	-4.2382	-2.3234	-6.7029	4.4526	-1.6557	4.4401	6.9218
H	-0.5344	-7.7351	-4.3745	-8.5935	-2.7195	1.5362	4.9240	7.0450	3.1701
H	-1.5037	-8.5524	-3.1121	-8.5564	-4.0284	0.3170	3.6828	8.1988	2.5966
H	-2.2130	-7.1947	-4.0394	-7.5100	-4.1321	1.7658	3.2748	7.0161	3.8771
C	4.1134	2.2829	4.5718	1.3540	0.5166	-4.5795	-6.4750	2.6539	-2.5014
C	0.2087	-1.1070	4.5960	5.9117	0.7102	-2.0273	-6.2910	-2.5505	-2.3914
C	4.9817	0.2495	6.2676	2.8195	-1.5898	-5.6602	-8.8905	1.8675	-3.6562
C	5.4428	1.8525	4.5243	0.7153	-0.6883	-4.8928	-7.6524	3.3479	-2.2047
C	1.7029	-2.1975	6.6451	6.5456	-1.2061	-3.9225	-8.4833	-2.2736	-4.0555
C	0.2172	-2.4899	4.7688	6.8005	-0.3119	-1.6988	-7.2802	-3.5335	-2.3922
C	5.8817	0.8320	5.3681	1.4463	-1.7436	-5.4374	-8.8631	2.9580	-2.7792
C	0.9603	-3.0396	5.8142	7.1256	-1.2754	-2.6553	-8.3823	-3.4003	-3.2389
C	-2.1129	3.5629	5.7245	5.3168	5.6306	-4.0917	-1.6937	-1.1345	-4.5536
C	1.6014	3.5156	5.1732	2.5784	3.1091	-4.5802	-4.0877	1.6146	-3.6544
C	-0.5442	1.7416	5.7010	5.3112	3.2334	-3.8490	-4.0001	-1.1920	-3.9199

C	0.2675	3.9874	5.7417	3.4371	4.3384	-4.8573	-2.9602	0.9092	-4.3956
C	2.6533	0.0535	7.2754	4.9447	-0.2015	-5.5979	-7.7107	-0.0255	-4.8874
C	3.2086	1.7002	5.4711	2.7296	0.6810	-4.8125	-6.5007	1.5659	-3.3861
C	0.9419	-0.2498	5.4238	5.3344	0.7961	-3.3022	-6.3699	-1.4269	-3.2287
C	3.6573	0.6791	6.3374	3.4654	-0.3913	-5.3672	-7.7226	1.1741	-3.9718
C	1.7108	-0.8094	6.4613	5.6535	-0.1809	-4.2598	-7.4931	-1.2822	-4.0653
C	0.7960	1.2380	5.1348	4.4418	2.0008	-3.5464	-5.1638	-0.4924	-3.1905
N	-0.8028	3.1117	5.2797	4.4982	4.4483	-3.8601	-2.7552	-0.4279	-3.8426
N	1.8687	2.1398	5.6094	3.4059	1.9045	-4.6083	-5.3352	0.8556	-3.7812
H	3.7747	3.0667	3.9036	0.7721	1.3301	-4.1595	-5.5417	2.9484	-2.0330
H	-0.3591	-0.6843	3.7751	5.6333	1.4464	-1.2804	-5.4444	-2.6356	-1.7184
H	5.3123	-0.5410	6.9367	3.3971	-2.4050	-6.0884	-9.8267	1.5561	-4.1129
H	6.1287	2.3122	3.8201	-0.3479	-0.7860	-4.6956	-7.6191	4.1890	-1.5177
H	2.3102	-2.6187	7.4424	6.7740	-1.9639	-4.6679	-9.3511	-2.1458	-4.6978
H	-0.3274	-3.1185	4.0739	7.2217	-0.3620	-0.6991	-7.1919	-4.3916	-1.7320
H	6.9130	0.4942	5.3318	0.9589	-2.6785	-5.6926	-9.7783	3.4961	-2.5502
H	0.9834	-4.1148	5.9679	7.8120	-2.0820	-2.4140	-9.1635	-4.1550	-3.2523
H	-2.2722	4.5946	5.3907	4.6859	6.5253	-4.0459	-0.7669	-0.5525	-4.5028
H	-2.8795	2.9296	5.2690	6.0862	5.7023	-3.3153	-1.5224	-2.1059	-4.0799
H	-2.2201	3.5368	6.8267	5.8194	5.6096	-5.0776	-1.9417	-1.2901	-5.6207
H	2.4079	4.1544	5.5443	1.8006	3.0254	-5.3435	-4.2443	2.6080	-4.0843
H	1.5653	3.5993	4.0751	2.0923	3.2340	-3.5989	-3.8101	1.7387	-2.6004
H	-1.3500	1.1023	5.3276	6.0706	3.3308	-3.0664	-3.8325	-2.1691	-3.4587
H	-0.5156	1.6436	6.8048	5.8299	3.0780	-4.8155	-4.3054	-1.3552	-4.9719
H	0.3289	4.0060	6.8481	3.8519	4.2640	-5.8810	-3.2073	0.8619	-5.4738
H	0.0644	5.0069	5.3946	2.8122	5.2375	-4.8053	-2.0323	1.4824	-4.2838
H	3.1601	-0.5561	8.0293	5.3406	-1.0106	-6.2188	-8.6573	-0.1009	-5.4312
H	2.0951	0.8515	7.7804	5.1000	0.7518	-6.1196	-6.8963	0.0925	-5.6128
H	0.7192	1.3429	4.0408	3.9350	2.2082	-2.5952	-4.8749	-0.3777	-2.1340

	**FΛ2**			**FV2**			**S**		
**atom**	**x**	**y**	**z**	**x**	**y**	**z**	**x**	**y**	**z**
O	-3.6486	3.4509	1.3104	-3.4075	4.0914	-0.7510	-4.3591	4.5251	-1.8824
C	-3.2434	2.0909	1.5671	-2.0094	4.0137	-0.4025	-4.6399	3.1314	-1.6418
C	-3.6719	1.7243	2.9879	-1.2787	5.1809	-1.0725	-5.9391	3.0189	-0.8413
C	-3.0779	2.6845	4.0223	-1.5126	5.2253	-2.5874	-5.9111	3.8565	0.4426
C	-3.4039	4.1273	3.6408	-3.0055	5.1598	-2.8986	-5.4821	5.2883	0.1333
C	-3.0368	4.3898	2.1674	-3.6606	3.9996	-2.1325	-4.1959	5.2871	-0.7095
H	-2.1524	2.0169	1.5004	-1.5917	3.0686	-0.7683	-3.8245	2.6895	-1.0578
H	-4.7677	1.7601	3.0518	-1.6391	6.1220	-0.6352	-6.7755	3.3611	-1.4655
H	-1.9844	2.5680	3.9941	-1.0396	4.3319	-3.0218	-5.1543	3.4165	1.1085
H	-4.4838	4.2936	3.7604	-3.4801	6.0984	-2.5798	-6.2736	5.7846	-0.4455
H	-3.4127	5.3709	1.8619	-4.7478	4.0467	-2.2470	-3.9667	6.3055	-1.0375
C	-3.8899	1.1945	0.5199	-1.9192	4.0823	1.1159	-4.7459	2.4541	-3.0022
O	-3.1913	0.4184	3.3122	0.1330	5.0497	-0.8851	-6.1330	1.6596	-0.4417
O	-3.5925	2.3954	5.3108	-0.9697	6.4149	-3.1309	-7.1913	3.8629	1.0483
O	-2.6778	5.0844	4.4020	-3.2777	4.9209	-4.2746	-5.1863	6.0460	1.2999
O	-1.6331	4.3311	2.0481	-3.1559	2.7854	-2.6480	-3.1486	4.7682	0.0830
H	-4.0171	0.1922	0.9361	-0.8947	4.3363	1.4122	-5.3043	1.5143	-2.9043
O	-3.1513	1.1205	-0.6912	-2.3443	2.8865	1.7569	-3.4776	2.2211	-3.6040
H	-4.8741	1.5971	0.2592	-2.5913	4.8768	1.4602	-5.2991	3.1209	-3.6731
H	-3.3316	1.4760	5.5263	-0.0145	6.4289	-2.9114	-7.4119	2.9336	1.2698
C	-3.2544	5.4201	5.6660	-3.2664	6.0802	-5.1107	-6.3129	6.6479	1.9413
C	-1.9841	0.3095	-0.5930	-1.3505	1.8650	1.7535	-2.8417	1.0609	-3.0754
O	0.3506	6.2741	-0.3573	-5.1489	-0.2791	-2.9971	0.3500	5.8570	-0.1526
C	-0.5189	5.2111	0.0737	-4.4046	0.7362	-2.3008	-0.8212	5.1149	-0.5388
C	-0.9301	5.4605	1.5264	-4.0630	1.8722	-3.2680	-1.9978	5.5794	0.3228

C	0.2946	5.6210	2.4367	-3.3324	1.3561	-4.5129	-1.6898	5.4230	1.8182
C	1.2900	6.6215	1.8492	-4.0702	0.1640	-5.1212	-0.3381	6.0492	2.1645
C	1.5600	6.3185	0.3669	-4.4163	-0.8759	-4.0448	0.7482	5.5997	1.1759
H	0.0215	4.2591	0.0399	-3.4615	0.3145	-1.9355	-0.6591	4.0479	-0.3491
H	-1.5472	6.3661	1.5971	-4.9772	2.3909	-3.5851	-2.2193	6.6349	0.1160
H	0.7968	4.6420	2.4767	-2.3440	0.9994	-4.1841	-1.6185	4.3433	2.0229
H	0.8592	7.6303	1.9115	-5.0137	0.5160	-5.5599	-0.4277	7.1416	2.0893
H	2.1512	7.1265	-0.0749	-5.0672	-1.6456	-4.4706	1.6603	6.1822	1.3378
C	-1.6876	5.1683	-0.8979	-5.2526	1.1860	-1.1212	-1.0220	5.3415	-2.0287
O	-0.0963	6.0535	3.7275	-3.2139	2.3760	-5.4885	-2.6950	6.0330	2.6071
O	2.5488	6.5858	2.5118	-3.2891	-0.5012	-6.1082	0.1142	5.6837	3.4634
O	2.2473	5.0921	0.2868	-3.2150	-1.4394	-3.5729	0.9863	4.2276	1.3826
H	-2.4837	4.5444	-0.4700	-4.8474	2.1274	-0.7275	-2.0185	4.9806	-2.3169
O	-1.3127	4.6762	-2.1795	-5.3059	0.2137	-0.0849	-0.0152	4.7033	-2.8072
H	-2.0654	6.1857	-1.0464	-6.2810	1.3474	-1.4612	-0.9531	6.4149	-2.2373
H	-0.8836	5.5348	3.9893	-3.0184	3.2150	-5.0240	-3.5629	5.8231	2.2064
C	2.6520	7.4270	3.6624	-3.4448	-0.0016	-7.4384	-0.3447	6.5247	4.5235
C	-1.0748	3.2702	-2.1669	-4.0756	0.1154	0.6328	-0.2529	3.3074	-2.9629
O	4.3493	3.8727	-2.4740	-2.6952	-4.6479	-1.8528	4.0065	2.3712	0.4334
C	3.1205	4.0515	-1.7418	-2.9375	-3.2299	-1.9445	2.6979	2.9225	0.2246
C	3.3880	4.9959	-0.5651	-3.1407	-2.8562	-3.4175	2.3355	3.7706	1.4490
C	4.5217	4.4779	0.3262	-1.9746	-3.3160	-4.2971	2.4365	2.9689	2.7528
C	5.7535	4.1666	-0.5152	-1.7022	-4.7969	-4.0654	3.8025	2.2995	2.8457
C	5.3847	3.2836	-1.7186	-1.5591	-5.0901	-2.5632	4.1201	1.5344	1.5575
H	2.7762	3.0838	-1.3564	-2.0760	-2.6812	-1.5446	1.9648	2.1145	0.1080
H	3.6657	5.9859	-0.9512	-4.0609	-3.3324	-3.7823	3.0275	4.6215	1.5106
H	4.1793	3.5374	0.7844	-1.0800	-2.7544	-3.9885	1.6719	2.1777	2.7186
H	6.1600	5.1073	-0.9107	-2.5565	-5.3793	-4.4363	4.5746	3.0726	2.9580
H	6.2438	3.2138	-2.3926	-1.5122	-6.1720	-2.4069	5.1660	1.2271	1.5838
C	2.1029	4.5998	-2.7193	-4.1739	-2.9397	-1.1194	2.7732	3.7425	-1.0463
O	4.8588	5.4394	1.3085	-2.2739	-3.1041	-5.6640	2.2513	3.8195	3.8695
O	6.7509	3.4805	0.2361	-0.5037	-5.2244	-4.7068	3.8801	1.3679	3.9229
O	5.0131	2.0082	-1.2507	-0.3869	-4.4665	-2.0934	3.2490	0.4289	1.4553
H	1.2169	4.9641	-2.1853	-4.5333	-1.9228	-1.3200	1.8580	4.3312	-1.1754
O	1.7468	3.5645	-3.6254	-3.8741	-3.1002	0.2598	2.9552	2.8663	-2.1525
H	2.5554	5.4477	-3.2599	-4.9633	-3.6472	-1.4240	3.6284	4.4339	-0.9645
H	4.0326	5.7034	1.7622	-2.5248	-2.1636	-5.7714	1.4345	4.3363	3.7172
C	7.7165	4.3297	0.8631	-0.6850	-5.7351	-6.0312	4.3697	1.9236	5.1467
C	0.9485	4.0560	-4.6922	-5.0631	-3.0630	1.0416	3.1694	3.5951	-3.3543
O	5.0215	-1.2419	-2.9468	1.7729	-5.1716	0.7744	3.5757	-2.7015	-0.4275
C	4.4167	-0.0693	-2.3571	0.8008	-4.4292	0.0078	3.0795	-1.3721	-0.1623
C	5.5465	0.8738	-1.9382	0.4782	-5.2323	-1.2519	3.8242	-0.8138	1.0509
C	6.5258	0.1967	-0.9703	1.7383	-5.5417	-2.0715	3.8041	-1.7344	2.2727
C	7.0095	-1.1351	-1.5359	2.8207	-6.1629	-1.1920	4.1982	-3.1545	1.8788
C	5.8273	-1.9699	-2.0518	2.9974	-5.3547	0.1030	3.4039	-3.5994	0.6424
H	3.8432	-0.3595	-1.4692	1.2254	-3.4623	-0.2863	2.0047	-1.4146	0.0467
H	6.0944	1.1889	-2.8362	0.0002	-6.1772	-0.9612	4.8688	-0.6778	0.7585
H	5.9749	-0.0130	-0.0411	2.1245	-4.5834	-2.4498	2.7770	-1.7641	2.6629
H	7.6886	-0.9380	-2.3774	2.5207	-7.1861	-0.9254	5.2657	-3.1789	1.6242
H	6.2061	-2.8316	-2.6092	3.6472	-5.9001	0.7938	3.7759	-4.5670	0.2918
C	3.4797	0.5345	-3.3973	-0.4104	-4.1997	0.9044	3.3664	-0.5342	-1.4018
O	7.6419	1.0351	-0.7328	1.4294	-6.4289	-3.1309	4.7102	-1.2398	3.2495
O	7.6597	-1.9487	-0.5648	4.1011	-6.1754	-1.8150	3.9125	-4.1114	2.8935
O	5.0580	-2.3883	-0.9456	3.5578	-4.1048	-0.2309	2.0418	-3.6868	1.0023
H	3.3294	1.6015	-3.1918	-1.3157	-4.0873	0.2943	3.3314	0.5308	-1.1434
O	2.2300	-0.1488	-3.4578	-0.2340	-3.0672	1.7533	2.4907	-0.8167	-2.4855

H	3.9480	0.4336	-4.3829	-0.5342	-5.0722	1.5545	4.3792	-0.7654	-1.7447
H	7.3060	1.8661	-0.3349	0.7851	-5.9730	-3.7133	4.3930	-0.3504	3.5157
C	9.0425	-1.6555	-0.3537	4.3383	-7.2780	-2.6928	4.9191	-4.2377	3.8999
C	1.3122	0.3049	-2.4648	-0.5911	-1.8481	1.1079	1.2434	-0.1422	-2.3624
O	3.4448	-5.6847	-0.7808	5.9291	-2.3420	1.9469	-0.2884	-6.1506	-0.4077
C	3.7034	-4.3701	-1.3089	4.6639	-2.9076	1.5612	0.5507	-4.9816	-0.4048
C	4.9671	-3.7853	-0.6668	4.8355	-3.7613	0.3038	1.3774	-4.9441	0.8824
C	4.9805	-3.8757	0.8627	5.5365	-3.0050	-0.8292	0.5048	-5.0552	2.1364
C	4.5724	-5.2707	1.3282	6.7953	-2.3060	-0.3213	-0.4682	-6.2254	2.0137
C	3.2833	-5.7064	0.6202	6.4955	-1.5114	0.9586	-1.2031	-6.1883	0.6650
H	2.8575	-3.7083	-1.0886	3.9546	-2.1051	1.3251	-0.0739	-4.0803	-0.4336
H	5.8552	-4.3095	-1.0452	5.4149	-4.6660	0.5311	2.1046	-5.7671	0.8885
H	4.2259	-3.1702	1.2406	4.8441	-2.2230	-1.1763	-0.0922	-4.1327	2.2024
H	5.3623	-5.9874	1.0657	7.5512	-3.0635	-0.0735	0.0985	-7.1653	2.0576
H	3.0486	-6.7421	0.8836	7.4319	-1.1344	1.3809	-1.7773	-7.1110	0.5375
C	3.8588	-4.5382	-2.8149	4.1582	-3.6904	2.7650	1.4091	-5.0534	-1.6606
O	6.2717	-3.5644	1.3609	5.8760	-3.8955	-1.8795	1.3091	-5.2328	3.2898
O	4.3104	-5.3168	2.7288	7.3179	-1.3819	-1.2717	-1.4636	-6.2046	3.0323
O	2.2547	-4.8384	1.0314	5.6330	-0.4488	0.6265	-2.0538	-5.0652	0.6574
H	4.2620	-3.6131	-3.2446	3.3263	-4.3386	2.4653	2.2397	-4.3412	-1.5790
O	2.6353	-4.8790	-3.4466	3.7543	-2.8080	3.8048	0.6461	-4.7927	-2.8324
H	4.5697	-5.3533	-2.9971	4.9751	-4.3105	3.1510	1.8133	-6.0679	-1.7499
H	6.6313	-2.8360	0.8165	5.1355	-4.5255	-1.9832	2.0928	-4.6541	3.2016
C	5.4520	-5.6036	3.5408	8.2568	-1.9406	-2.1943	-1.1149	-6.9079	4.2271
C	1.9339	-3.7270	-3.9060	2.3874	-2.4311	3.6831	0.5481	-3.3977	-3.0959
O	-1.3870	-5.1074	0.9511	4.9420	2.6685	2.4200	-4.9993	-4.4499	-1.4377
C	-0.0871	-4.6152	0.5579	4.6427	1.3515	1.9211	-3.6284	-4.2880	-1.0277
C	0.9787	-5.4104	1.3074	5.8935	0.8231	1.2211	-3.3765	-5.2331	0.1461
C	0.7792	-5.3410	2.8244	6.3082	1.7573	0.0758	-4.3313	-4.9265	1.3077
C	-0.6441	-5.7604	3.1807	6.4323	3.1978	0.5719	-5.7796	-4.9124	0.8182
C	-1.6606	-4.9788	2.3272	5.1940	3.6020	1.3930	-5.9249	-4.0531	-0.4505
H	0.0071	-3.5584	0.8361	3.8404	1.4147	1.1787	-3.4689	-3.2603	-0.6839
H	0.9443	-6.4598	0.9853	6.7135	0.7523	1.9474	-3.5362	-6.2679	-0.1841
H	0.9063	-4.2912	3.1270	5.5035	1.7298	-0.6744	-4.0891	-3.9175	1.6751
H	-0.7679	-6.8314	2.9670	7.3102	3.2708	1.2292	-6.0729	-5.9405	0.5630
H	-2.6619	-5.3939	2.4760	5.3760	4.5596	1.8900	-6.9171	-4.2039	-0.8863
C	0.0287	-4.7393	-0.9660	4.1881	0.5047	3.0974	-2.7492	-4.5734	-2.2343
O	1.7132	-6.1813	3.4800	7.5422	1.3363	-0.4775	-4.1900	-5.8966	2.3293
O	-0.9842	-5.4940	4.5353	6.5366	4.1422	-0.4861	-6.6841	-4.3723	1.7730
O	-1.6215	-3.6202	2.7140	4.0938	3.6931	0.5170	-5.7340	-2.7003	-0.0988
H	1.0367	-5.0533	-1.2464	4.1624	-0.5466	2.7927	-1.7000	-4.5753	-1.9171
O	-0.1958	-3.5120	-1.6427	2.9237	0.9003	3.6273	-2.9444	-3.6625	-3.3121
H	-0.6883	-5.5062	-1.3017	4.9070	0.6086	3.9165	-2.9915	-5.5656	-2.6295
H	2.6066	-5.8351	3.2757	7.4171	0.4230	-0.8088	-3.2473	-5.9092	2.5954
C	-0.5934	-6.5005	5.4722	7.8583	4.3460	-0.9899	-7.1623	-5.2980	2.7511
C	-1.5052	-2.9995	-1.4414	1.8573	0.8246	2.6873	-2.7179	-2.3034	-2.9636
O	-4.5486	-1.3965	2.7662	1.1237	5.7888	1.0891	-7.2187	0.1691	-1.8643
C	-3.4677	-2.1393	2.1716	1.9443	4.6140	1.2320	-6.2669	-0.8884	-1.6449
C	-2.8098	-3.0203	3.2376	3.2882	4.8710	0.5424	-6.7323	-1.7455	-0.4618
C	-2.3545	-2.1914	4.4461	3.0976	5.2602	-0.9270	-6.9639	-0.8985	0.7935
C	-3.4879	-1.2955	4.9460	2.0986	6.4073	-1.0464	-7.8678	0.2881	0.4710
C	-4.1283	-0.5165	3.7895	0.8171	6.1052	-0.2523	-7.3575	1.0450	-0.7652
H	-2.7090	-1.4432	1.7957	1.4520	3.7605	0.7492	-5.2862	-0.4581	-1.4070
H	-3.5116	-3.7913	3.5818	3.8146	5.6855	1.0578	-7.6716	-2.2519	-0.7211
H	-1.5392	-1.5361	4.1022	2.6722	4.3880	-1.4471	-5.9870	-0.5007	1.1091
H	-4.2672	-1.9285	5.3916	2.5471	7.3128	-0.6160	-8.8741	-0.0851	0.2379

H	-5.0332	-0.0138	4.1430	0.1933	7.0031	-0.2121	-8.0985	1.7906	-1.0686
C	-4.0361	-2.9188	0.9948	2.0804	4.3344	2.7140	-6.1670	-1.6820	-2.9305
O	-1.9313	-3.0310	5.5043	4.3302	5.6542	-1.5011	-7.5615	-1.6735	1.8160
O	-3.0340	-0.3335	5.8949	1.7195	6.6442	-2.3986	-7.9275	1.2177	1.5493
H	-3.2953	-3.6544	0.6725	2.8331	3.5517	2.8714	-5.6071	-2.6076	-2.7429
O	-4.2903	-2.0694	-0.1194	0.8174	3.9141	3.2034	-5.4995	-0.8917	-3.9020
H	-4.9474	-3.4480	1.3081	2.4140	5.2494	3.2312	-7.1795	-1.9494	-3.2748
H	-1.4178	-3.7687	5.1172	5.0032	4.9919	-1.2452	-7.0863	-2.5277	1.8605
C	-3.0868	-0.7676	7.2570	2.5232	7.6072	-3.0860	-8.9921	0.9889	2.4775
C	-5.6270	-1.5748	-0.1849	0.8755	3.5258	4.5687	-5.2627	-1.6249	-5.0959
H	-1.6172	0.1577	-1.6113	-1.7175	1.0634	2.4020	-1.9232	0.9062	-3.6486
H	-1.2015	0.7896	0.0117	-1.1873	1.4564	0.7445	-2.5728	1.1858	-2.0157
H	-2.2418	-0.6622	-0.1635	-0.4051	2.2538	2.1450	-3.5003	0.1926	-3.1805
H	-1.0438	2.9392	-3.2061	-4.3112	-0.3415	1.5950	0.5169	2.9233	-3.6350
H	-0.1110	3.0244	-1.7030	-3.3488	-0.5127	0.1028	-0.1745	2.7681	-2.0081
H	-1.8770	2.7478	-1.6350	-3.6491	1.1106	0.8034	-1.2501	3.1334	-3.3860
H	0.6552	3.1954	-5.3003	-4.7744	-3.1780	2.0871	3.2472	2.8657	-4.1651
H	0.0494	4.5614	-4.3141	-5.5933	-2.1101	0.9090	2.3353	4.2819	-3.5509
H	1.5170	4.7646	-5.3149	-5.7398	-3.8842	0.7566	4.1050	4.1731	-3.2981
H	0.3778	-0.2417	-2.6171	-0.4663	-1.0508	1.8444	0.6494	-0.4002	-3.2442
H	1.6811	0.1055	-1.4470	0.0556	-1.6437	0.2417	0.6908	-0.4630	-1.4656
H	1.1345	1.3834	-2.5736	-1.6379	-1.8827	0.7768	1.3953	0.9447	-2.3174
H	2.5072	-3.2078	-4.6895	2.2498	-1.5206	4.2692	-0.1709	-3.2742	-3.9093
H	0.9855	-4.0784	-4.3171	2.1046	-2.2290	2.6421	0.1848	-2.8463	-2.2176
H	1.7230	-3.0197	-3.0968	1.7239	-3.2274	4.0511	1.5216	-2.9682	-3.3673
H	-1.5974	-2.1025	-2.0549	0.9309	0.8651	3.2601	-2.6438	-1.7448	-3.8982
H	-2.2673	-3.7377	-1.7356	1.8755	1.6656	1.9837	-3.5470	-1.8914	-2.3772
H	-1.6771	-2.7398	-0.3909	1.8903	-0.1155	2.1199	-1.7885	-2.1792	-2.3938
H	-5.6704	-0.9060	-1.0472	-0.1173	3.1613	4.8416	-4.7092	-0.9689	-5.7733
H	-6.3351	-2.4010	-0.3369	1.6086	2.7207	4.7168	-4.6686	-2.5273	-4.8908
H	-5.8905	-1.0258	0.7252	1.1448	4.3788	5.2114	-6.2095	-1.9234	-5.5720
H	-2.6443	6.2278	6.0782	-3.5902	5.7484	-6.1005	-5.9123	7.2764	2.7408
H	-4.2864	5.7740	5.5331	-3.9710	6.8320	-4.7289	-6.8678	7.2737	1.2284
H	-3.2523	4.5585	6.3395	-2.2654	6.5173	-5.1624	-6.9831	5.8879	2.3520
H	3.6892	7.3632	4.0007	-2.8513	-0.6525	-8.0852	0.1577	6.1810	5.4312
H	2.4174	8.4682	3.3998	-4.4991	-0.0451	-7.7455	-0.0716	7.5717	4.3301
H	1.9736	7.0918	4.4523	-3.0888	1.0303	-7.5089	-1.4299	6.4502	4.6390
H	8.4446	3.6698	1.3411	0.2966	-6.0786	-6.3669	4.4304	1.0986	5.8608
H	8.2248	4.9547	0.1157	-1.3867	-6.5807	-6.0265	5.3697	2.3563	5.0017
H	7.2365	4.9698	1.6087	-1.0610	-4.9512	-6.6945	3.6885	2.6958	5.5156
H	9.4171	-2.4171	0.3350	5.3813	-7.2003	-3.0102	4.6041	-5.0558	4.5525
H	9.5950	-1.7172	-1.3018	4.1885	-8.2286	-2.1617	5.8865	-4.4895	3.4425
H	9.1718	-0.6571	0.0734	3.6718	-7.2417	-3.5592	5.0219	-3.3111	4.4719
H	5.0893	-5.6839	4.5685	8.6241	-1.1112	-2.8035	-1.9937	-6.8715	4.8756
H	5.9114	-6.5557	3.2399	9.0986	-2.4009	-1.6580	-0.8707	-7.9555	4.0015
H	6.1928	-4.8026	3.4614	7.7756	-2.6920	-2.8272	-0.2606	-6.4340	4.7192
H	-1.0084	-6.1998	6.4375	7.7909	5.1627	-1.7131	-7.9181	-4.7669	3.3352
H	-1.0122	-7.4742	5.1820	8.5383	4.6349	-0.1760	-7.6238	-6.1671	2.2618
H	0.4956	-6.5787	5.5322	8.2375	3.4404	-1.4719	-6.3494	-5.6382	3.3988
H	-2.7888	0.0890	7.8665	2.0778	7.7316	-4.0761	-8.9511	1.8057	3.2022
H	-4.1081	-1.0706	7.5271	2.5107	8.5697	-2.5560	-9.9631	1.0045	1.9634
H	-2.4039	-1.6061	7.4205	3.5544	7.2537	-3.1752	-8.8613	0.0271	2.9812
C	-5.5224	2.0391	-2.6530	-6.0241	-2.6239	5.4075	5.5908	0.8970	-3.4116
C	-5.1809	-3.1938	-2.9177	-2.1162	0.9173	5.8804	7.4219	-1.5907	0.8207
C	-7.9500	1.2331	-3.7678	-3.7019	-4.0465	4.8246	7.3010	-1.0459	-4.4475
C	-6.7116	2.6702	-2.2758	-5.8864	-3.9469	5.8392	5.2334	0.1919	-4.5641

C	-7.4179	-2.8626	-4.5048	-0.6854	-1.0763	4.6078	9.1192	-2.6742	-1.0746
C	-6.1299	-4.2121	-2.9832	-0.7416	0.8201	6.0904	7.9267	-2.8774	1.0065
C	-7.9295	2.2689	-2.8277	-4.7238	-4.6630	5.5543	6.0865	-0.7796	-5.0891
C	-7.2562	-4.0506	-3.7925	-0.0153	-0.1774	5.4372	8.7854	-3.4254	0.0521
C	-0.6266	-1.5082	-4.8094	-5.7056	3.3112	2.9235	7.7741	3.4585	2.0325
C	-3.1444	1.1483	-3.9653	-6.4847	-0.1923	4.0092	6.6179	2.6652	-1.4510
C	-2.9613	-1.6459	-4.2959	-4.3353	1.6302	3.9335	7.8515	1.4173	0.7665
C	-1.9911	0.4859	-4.7077	-6.5245	1.0619	3.1461	7.2819	3.4508	-0.3278
C	-6.7512	-0.5274	-5.1625	-2.7493	-2.0607	3.5711	8.9500	-0.6416	-2.5534
C	-5.5420	0.9984	-3.5944	-4.9999	-2.0023	4.6782	6.8095	0.6339	-2.7694
C	-5.3215	-2.0029	-3.6467	-2.7971	0.0377	5.0246	7.7568	-0.8233	-0.3080
C	-6.7699	0.6034	-4.1643	-3.8297	-2.7316	4.3774	7.6732	-0.3464	-3.2995
C	-6.4672	-1.8339	-4.4465	-2.0688	-0.9882	4.3950	8.6216	-1.3783	-1.2691
C	-4.1502	-1.0266	-3.5391	-4.2722	0.3565	4.7972	7.1768	0.5883	-0.3419
N	-1.7489	-0.8440	-4.1541	-5.7039	2.1103	3.7532	7.1966	2.7078	0.9267
N	-4.3694	0.3425	-4.0626	-5.1090	-0.6840	4.1547	7.2492	1.3427	-1.6142
H	-4.5852	2.3382	-2.2008	-6.9320	-2.0795	5.6404	4.9010	1.6183	-2.9924
H	-4.3260	-3.2976	-2.2587	-2.6811	1.6983	6.3833	6.7598	-1.1765	1.5772
H	-8.8900	0.9166	-4.2132	-2.7960	-4.5951	4.5791	7.9754	-1.8012	-4.8436
H	-6.6804	3.4723	-1.5433	-6.6913	-4.4112	6.4021	4.2771	0.3955	-5.0372
H	-8.3036	-2.7148	-5.1177	-0.1445	-1.8729	4.1064	9.7765	-3.0951	-1.8311
H	-5.9924	-5.1190	-2.4014	-0.2435	1.5187	6.7555	7.6505	-3.4427	1.8919
H	-8.8542	2.7575	-2.5343	-4.6145	-5.6911	5.8867	5.8114	-1.3284	-5.9851
H	-8.0085	-4.8317	-3.8562	1.0596	-0.2481	5.5708	9.1856	-4.4269	0.1788
H	0.2761	-0.8969	-4.7194	-6.7343	3.6693	2.8010	7.2418	4.4095	2.1445
H	-0.4466	-2.4656	-4.3122	-5.1152	4.0955	3.4110	7.6672	2.8852	2.9602
H	-0.8229	-1.6949	-5.8823	-5.2720	3.1335	1.9225	8.8488	3.6782	1.8813
H	-3.3495	2.1305	-4.4027	-7.0824	-0.9804	3.5430	6.7299	3.2071	-2.3952
H	-2.8526	1.2973	-2.9234	-6.9293	0.0426	4.9923	5.5499	2.5753	-1.2210
H	-2.7689	-2.6436	-3.8946	-3.7544	2.4179	4.4222	7.7814	0.8710	1.7119
H	-3.2459	-1.7558	-5.3606	-3.8486	1.4183	2.9725	8.9256	1.5240	0.5143
H	-2.2237	0.4293	-5.7888	-6.1933	0.8198	2.1220	8.3375	3.6616	-0.5909
H	-1.0851	1.0906	-4.5850	-7.5600	1.4189	3.0877	6.7626	4.4083	-0.2087
H	-7.7123	-0.5946	-5.6813	-2.0084	-2.7917	3.2433	9.6202	-1.2515	-3.1664
H	-5.9654	-0.3302	-5.9020	-3.1973	-1.6224	2.6748	9.4512	0.3056	-2.3190
H	-3.8666	-0.9636	-2.4751	-4.7147	0.6008	5.7804	6.1145	0.5064	-0.0717

Table S13. Cartesian coordinates of atoms in 11 different structures of MIA:DM-β-CD in water (PCM) obtained from the M062X-GD3/6-31G(d,p) calculations performed using in the initial model of the complex the optimized structure BOYFOK03 of DM-β-CD. The true minima are confirmed by zero imaginary frequencies.

	CR1			NR1			M1		
**atom**	**x**	**y**	**z**	**x**	**y**	**z**	**x**	**y**	**z**
C	-3.1096	5.1866	-0.2202	5.8196	1.3628	-0.4678	2.5087	5.1552	-0.0242
C	-2.2596	5.2913	-1.4974	5.6463	2.3537	0.6876	1.8437	5.1178	1.3586
C	-0.8394	4.7858	-1.2809	4.3160	3.0782	0.5764	0.4884	4.4313	1.2862
C	-0.2597	5.4187	-0.0244	4.1042	3.6241	-0.8272	-0.3332	5.0773	0.1810
C	-1.1854	5.1762	1.1650	4.3333	2.5458	-1.8800	0.4175	5.0366	-1.1461
C	-0.6364	5.7524	2.4611	4.1781	3.0730	-3.2997	-0.3797	5.6790	-2.2718
C	-2.6536	4.9632	-3.8460	6.2797	2.3241	2.9956	2.6211	4.8240	3.6070
C	-1.1044	8.0688	2.1892	6.2044	4.2830	-3.5600	-0.0948	7.9939	-1.8066
O	-2.9369	4.5655	-2.5069	5.7295	1.6140	1.8921	2.7309	4.4479	2.2362
O	-0.0665	5.1064	-2.4158	4.2794	4.1190	1.5249	-0.1515	4.5576	2.5312
O	1.0049	4.8132	0.1758	2.7588	4.0645	-0.8507	-1.5551	4.3656	0.0976
O	-2.4540	5.7721	0.8764	5.6458	2.0027	-1.7099	1.6595	5.7282	-0.9874
O	-0.1214	7.0604	2.3445	4.7885	4.3231	-3.5295	-0.9893	6.9016	-1.9216
H	-4.0388	5.7495	-0.3548	6.8393	0.9644	-0.4710	3.3987	5.7910	0.0111
H	-2.2041	6.3549	-1.7744	6.4629	3.0878	0.6332	1.6932	6.1548	1.6943
H	-0.8703	3.6966	-1.1195	3.5155	2.3437	0.7639	0.6519	3.3706	1.0225
H	-0.1455	6.5002	-0.1822	4.7891	4.4657	-0.9986	-0.5242	6.1250	0.4508
H	-1.3149	4.0914	1.3070	3.5879	1.7491	-1.7334	0.6152	3.9876	-1.4209
H	0.2040	5.1263	2.7780	3.1093	3.2201	-3.4863	-1.2031	5.0061	-2.5314
H	-3.3816	4.4524	-4.4777	6.3233	1.6193	3.8269	3.4207	4.3014	4.1335
H	-2.7722	6.0468	-3.9579	7.2924	2.6731	2.7631	2.7578	5.9058	3.7206
H	-1.6401	4.6778	-4.1310	5.6499	3.1760	3.2608	1.6504	4.5326	4.0103
H	-0.6066	9.0260	2.3523	6.5419	5.2434	-3.9524	-0.7020	8.9001	-1.7734
H	-1.5501	8.0543	1.1894	6.6297	4.1259	-2.5631	0.5152	7.9303	-0.8997
H	-1.9070	7.9480	2.9280	6.5574	3.4780	-4.2174	0.5775	8.0411	-2.6731
H	0.8664	5.1700	-2.1384	3.5877	4.7470	1.2438	-1.1072	4.4111	2.3992
C	2.0645	5.6321	0.6175	2.4427	5.2351	-1.5629	-2.7423	5.1187	-0.0080
C	3.1329	5.6535	-0.4790	1.8762	6.2390	-0.5540	-3.6382	4.7528	1.1768
C	3.7484	4.2726	-0.6320	0.5637	5.7126	0.0045	-4.0823	3.3005	1.0837
C	4.2036	3.7304	0.7212	-0.3766	5.2906	-1.1226	-4.6364	3.0142	-0.3127
C	3.0600	3.7946	1.7325	0.3191	4.3310	-2.0822	-3.7069	3.4931	-1.4308
C	3.4813	3.3915	3.1329	-0.5334	3.9788	-3.2885	-4.3974	3.4046	-2.7853
C	3.2884	6.7627	-2.5977	2.8596	7.6169	1.1397	-3.5981	5.1325	3.5505
C	1.7865	1.9329	3.8571	-0.5724	1.6329	-3.2817	-2.6131	3.0476	-4.2959
O	2.4756	6.0707	-1.6594	2.8633	6.3740	0.4510	-2.8749	5.0286	2.3312
O	4.8219	4.3403	-1.5388	-0.0369	6.6966	0.8138	-5.0772	3.0766	2.0530
O	4.5812	2.3809	0.4923	-1.4738	4.6214	-0.5163	-4.8243	1.6101	-0.3818
O	2.5872	5.1387	1.8182	1.5117	4.9466	-2.5705	-3.3860	4.8647	-1.2239
O	2.3714	3.2105	3.9867	-0.0967	2.7959	-3.9247	-3.6316	3.9159	-3.8506
H	1.7020	6.6402	0.8322	3.3292	5.6327	-2.0644	-2.5140	6.1875	-0.0047
H	3.9129	6.3769	-0.2009	1.7094	7.2021	-1.0580	-4.5312	5.3956	1.1533
H	2.9620	3.5956	-1.0043	0.7929	4.8094	0.5907	-3.2047	2.6506	1.2512
H	5.0607	4.3161	1.0852	-0.7211	6.1793	-1.6719	-5.6038	3.5319	-0.3991
H	2.2516	3.1397	1.3800	0.5756	3.4177	-1.5296	-2.7893	2.8859	-1.4135
H	4.0868	4.1993	3.5573	-0.4526	4.7900	-4.0188	-5.3070	4.0145	-2.7446
H	4.0969	2.4812	3.0858	-1.5857	3.8921	-2.9795	-4.6810	2.3570	-2.9707

H	2.6315	7.0600	-3.4160	3.6991	7.5885	1.8355	-2.9024	5.5421	4.2847
H	3.7213	7.6595	-2.1393	3.0041	8.4449	0.4354	-4.4470	5.8163	3.4339
H	4.0889	6.1218	-2.9729	1.9245	7.7616	1.6845	-3.9627	4.1572	3.8790
H	0.9685	1.8718	4.5771	-0.2040	0.7701	-3.8402	-2.2329	3.4492	-5.2367
H	1.3822	1.7563	2.8508	-0.2251	1.5531	-2.2432	-1.7818	2.9896	-3.5831
H	2.5203	1.1450	4.0787	-1.6734	1.6100	-3.2769	-3.0006	2.0334	-4.4686
H	5.2483	3.4629	-1.5648	-0.8269	6.2921	1.2209	-5.4881	2.2129	1.8693
C	5.8670	1.9468	0.8435	-2.7194	5.2593	-0.5164	-6.0165	1.1019	-0.9280
C	6.4208	1.1623	-0.3484	-3.3276	5.0891	0.8825	-6.7125	0.2765	0.1593
C	5.5259	-0.0405	-0.5986	-3.6515	3.6238	1.1304	-5.8004	-0.8759	0.5392
C	5.4336	-0.8606	0.6797	-4.5548	3.1511	0.0016	-5.4258	-1.6646	-0.7068
C	5.0042	-0.0170	1.8799	-3.8318	3.3312	-1.3305	-4.8393	-0.7681	-1.7947
C	5.0969	-0.8030	3.1812	-4.6446	2.8178	-2.4973	-4.5897	-1.5175	-3.0961
C	7.5048	1.7952	-2.3817	-2.9913	6.2013	2.9684	-8.0494	0.8150	2.0662
C	7.4467	-0.8094	3.5495	-6.7946	2.6901	-3.3753	-6.8044	-1.7575	-3.9189
O	6.4600	2.0466	-1.4497	-2.4006	5.5930	1.8247	-6.9423	1.1536	1.2412
O	6.0507	-0.8141	-1.6531	-4.2567	3.4831	2.3916	-6.4242	-1.7068	1.4892
O	4.4763	-1.8796	0.4347	-4.8470	1.7895	0.2425	-4.4675	-2.6200	-0.2841
O	5.8393	1.1403	1.9936	-3.5636	4.7238	-1.5086	-5.7389	0.3146	-2.0546
O	6.2756	-1.5672	3.3092	-5.9662	3.2883	-2.3964	-5.6286	-2.3988	-3.4612
H	6.5161	2.7964	1.0789	-2.6119	6.3231	-0.7515	-6.6671	1.9146	-1.2672
H	7.4381	0.8246	-0.1019	-4.2587	5.6724	0.9251	-7.6662	-0.1042	-0.2351
H	4.5195	0.3252	-0.8472	-2.7104	3.0499	1.0817	-4.8820	-0.4316	0.9468
H	6.4156	-1.3068	0.8866	-5.4791	3.7422	-0.0105	-6.3204	-2.1705	-1.0942
H	3.9602	0.3019	1.7336	-2.8825	2.7724	-1.3023	-3.8792	-0.3658	-1.4385
H	4.2775	-1.5248	3.2187	-4.6387	1.7176	-2.4685	-3.6993	-2.1416	-2.9829
H	4.9944	-0.0986	4.0222	-4.1775	3.1402	-3.4419	-4.4067	-0.7761	-3.8900
H	7.4248	2.5616	-3.1535	-2.1707	6.5763	3.5821	-8.1225	1.5972	2.8227
H	8.4817	1.8755	-1.8912	-3.6310	7.0402	2.6710	-8.9731	0.7969	1.4761
H	7.3967	0.8032	-2.8263	-3.5768	5.4723	3.5318	-7.9012	-0.1559	2.5434
H	8.2194	-1.5095	3.8721	-7.8143	3.0323	-3.1925	-7.4294	-2.5254	-4.3779
H	7.7869	-0.2824	2.6513	-6.4897	2.9955	-4.3858	-7.3573	-1.2807	-3.1020
H	7.2783	-0.0667	4.3402	-6.7477	1.5969	-3.2992	-6.5668	-0.9896	-4.6667
H	5.5827	-1.6710	-1.6452	-4.7760	2.6569	2.3935	-5.8834	-2.5146	1.5717
C	4.8273	-3.2053	0.7250	-6.1784	1.3471	0.1200	-4.6379	-3.9524	-0.6861
C	4.4100	-4.0651	-0.4779	-6.5014	0.5955	1.4132	-4.3902	-4.8276	0.5468
C	2.8931	-4.1889	-0.5559	-5.5568	-0.5883	1.5332	-2.9273	-4.7453	0.9559
C	2.3381	-4.6348	0.7961	-5.5527	-1.4141	0.2476	-2.0331	-5.0144	-0.2513
C	2.7866	-3.6233	1.8541	-5.4321	-0.5800	-1.0276	-2.3878	-4.1056	-1.4235
C	2.2697	-3.9121	3.2504	-5.7857	-1.4274	-2.2381	-1.6134	-4.4569	-2.6802
C	5.3100	-4.3293	-2.6834	-7.0649	1.2497	3.6450	-5.6972	-5.3574	2.4730
C	1.7386	-1.7388	4.0081	-4.2558	-0.7810	-3.9144	-0.9161	-2.4031	-3.5862
O	4.9340	-3.4428	-1.6367	-6.3304	1.5288	2.4605	-5.2585	-4.3612	1.5595
O	2.5588	-5.0726	-1.5898	-5.9270	-1.4023	2.6233	-2.6704	-5.6841	1.9730
O	0.9284	-4.6935	0.6697	-4.4288	-2.2740	0.3116	-0.6957	-4.7936	0.1728
O	4.2133	-3.6504	1.9051	-6.3383	0.5175	-0.9961	-3.7691	-4.2886	-1.7316
O	2.5642	-2.8778	4.1682	-5.5971	-0.7680	-3.4696	-1.7994	-3.5055	-3.7062
H	5.9049	-3.2858	0.8974	-6.8534	2.1950	-0.0246	-5.6500	-4.1102	-1.0707
H	4.8473	-5.0667	-0.3562	-7.5441	0.2484	1.3753	-4.6325	-5.8684	0.2871
H	2.5045	-3.1765	-0.7524	-4.5402	-0.1910	1.6695	-2.7264	-3.7183	1.3012
H	2.7383	-5.6285	1.0448	-6.4855	-1.9976	0.2103	-2.1652	-6.0611	-0.5633
H	2.4473	-2.6252	1.5395	-4.4000	-0.2098	-1.1002	-2.2014	-3.0588	-1.1383
H	2.7530	-4.8192	3.6301	-6.8502	-1.6807	-2.1770	-1.9885	-5.4165	-3.0549
H	1.1865	-4.0791	3.2019	-5.1971	-2.3563	-2.1978	-0.5493	-4.5688	-2.4413
H	5.7203	-3.7084	-3.4816	-6.8880	2.0844	4.3242	-6.3798	-4.8661	3.1672
H	6.0799	-5.0288	-2.3373	-8.1368	1.1873	3.4230	-6.2337	-6.1543	1.9444

H	4.4435	-4.8848	-3.0485	-6.7335	0.3152	4.1016	-4.8533	-5.7857	3.0180
H	1.9019	-1.1027	4.8810	-4.2544	-0.4407	-4.9514	-1.1668	-1.7044	-4.3876
H	1.9965	-1.1639	3.1065	-3.6236	-0.0972	-3.3313	-1.0238	-1.8867	-2.6219
H	0.6802	-2.0230	3.9528	-3.8371	-1.7947	-3.8572	0.1246	-2.7343	-3.6929
H	1.6226	-4.9284	-1.8465	-5.1857	-2.0191	2.7743	-1.7264	-5.6128	2.2081
C	0.2565	-5.7654	1.2813	-4.6405	-3.6242	0.6003	0.1725	-5.8915	0.1592
C	-0.6579	-6.4218	0.2488	-3.6019	-4.0370	1.6469	1.0261	-5.8351	1.4277
C	-1.6781	-5.3936	-0.2053	-2.1961	-3.9531	1.0521	1.9724	-4.6457	1.3725
C	-2.3883	-4.7424	0.9794	-2.1631	-4.8106	-0.2182	2.7523	-4.6642	0.0605
C	-1.4196	-4.2775	2.0629	-3.2328	-4.2837	-1.1818	1.8006	-4.7332	-1.1332
C	-2.1443	-3.8931	3.3391	-3.2736	-5.0193	-2.5069	2.5225	-4.8425	-2.4641
C	-0.3214	-8.0536	-1.4350	-3.4783	-3.7688	4.0178	0.6096	-6.3749	3.7174
C	-0.7304	-4.1273	5.2312	-5.4059	-4.7260	-3.5157	1.1312	-5.8790	-4.0901
O	0.1461	-6.8669	-0.8176	-3.7437	-3.1658	2.7560	0.1396	-5.7564	2.5255
O	-2.6180	-6.0009	-1.0646	-1.2199	-4.3050	2.0041	2.8443	-4.6949	2.4741
O	-3.0945	-3.6083	0.4948	-0.8903	-4.7065	-0.8308	3.5301	-3.4737	0.0436
O	-0.5116	-5.3302	2.3789	-4.5146	-4.4051	-0.5623	0.9799	-5.8944	-0.9912
O	-1.2807	-3.2570	4.2567	-4.0490	-4.3223	-3.4615	1.6429	-4.6710	-3.5556
H	0.9714	-6.4932	1.6777	-5.6547	-3.7923	0.9774	-0.3915	-6.8295	0.1338
H	-1.1659	-7.2673	0.7366	-3.8123	-5.0725	1.9530	1.6179	-6.7602	1.4820
H	-1.1124	-4.6086	-0.7268	-2.0102	-2.9091	0.7651	1.3589	-3.7287	1.3906
H	-3.0877	-5.4720	1.4152	-2.3839	-5.8579	0.0347	3.4111	-5.5451	0.0543
H	-0.8612	-3.4031	1.7028	-3.0176	-3.2244	-1.3885	1.1724	-3.8290	-1.1440
H	-2.6038	-4.7872	3.7837	-3.6514	-6.0417	-2.3605	3.0374	-5.8130	-2.5193
H	-2.9432	-3.1904	3.0770	-2.2515	-5.0727	-2.8959	3.2742	-4.0497	-2.5174
H	0.3990	-8.3088	-2.2140	-3.6441	-2.9983	4.7720	-0.1935	-6.2889	4.4499
H	-0.3703	-8.8776	-0.7112	-4.1641	-4.6050	4.1986	0.8262	-7.4352	3.5418
H	-1.3094	-7.9072	-1.8813	-2.4467	-4.1217	4.0637	1.5072	-5.8742	4.0855
H	0.0241	-3.5569	5.7779	-5.8975	-4.0795	-4.2459	0.4230	-5.6005	-4.8734
H	-1.5041	-4.4661	5.9317	-5.4890	-5.7695	-3.8460	1.9361	-6.4823	-4.5291
H	-0.2564	-4.9993	4.7701	-5.8994	-4.6169	-2.5450	0.6136	-6.4701	-3.3282
H	-3.1095	-5.2801	-1.5042	-0.9562	-5.2338	1.8610	3.6186	-4.1343	2.2774
C	-4.4665	-3.7717	0.2666	-0.1851	-5.9008	-1.1021	4.8864	-3.5678	-0.3208
C	-4.8128	-3.1630	-1.1022	0.6312	-6.3495	0.1186	5.7201	-2.8778	0.7643
C	-4.7446	-1.6406	-1.0787	1.5522	-5.2015	0.5041	5.4986	-1.3722	0.7534
C	-5.5074	-1.1190	0.1351	2.4174	-4.8655	-0.7025	5.6267	-0.8329	-0.6663
C	-5.0032	-1.7759	1.4136	1.5692	-4.5615	-1.9366	4.7545	-1.6097	-1.6449
C	-5.7005	-1.2501	2.6590	2.4087	-4.3404	-3.1743	4.9116	-1.1042	-3.0730
C	-4.2399	-3.6083	-3.4004	0.2118	-7.7027	2.0424	6.3067	-3.4070	3.0311
C	-7.8058	-2.3418	2.5125	4.3368	-5.0343	-4.2784	7.0486	-1.9660	-3.6366
O	-3.9089	-3.7457	-2.0233	-0.2766	-6.7076	1.1462	5.3409	-3.4726	1.9888
O	-5.3094	-1.1355	-2.2682	2.3494	-5.5431	1.6148	6.4404	-0.7696	1.6074
O	-5.2849	0.2771	0.1640	3.1895	-3.7292	-0.3411	5.2153	0.5215	-0.6259
O	-5.2116	-3.1865	1.3034	0.6722	-5.6519	-2.1766	5.1161	-2.9922	-1.5800
O	-7.0988	-1.1147	2.5284	3.4068	-5.3331	-3.2579	6.2426	-0.8181	-3.4385
H	-4.7317	-4.8338	0.2665	-0.8732	-6.6961	-1.4138	5.1841	-4.6175	-0.4074
H	-5.8443	-3.4541	-1.3541	1.2204	-7.2315	-0.1706	6.7821	-3.0790	0.5593
H	-3.6891	-1.3372	-0.9745	0.9177	-4.3351	0.7290	4.4716	-1.1801	1.0999
H	-6.5778	-1.3340	0.0078	3.0767	-5.7144	-0.9200	6.6768	-0.9044	-0.9797
H	-3.9267	-1.5706	1.5136	0.9869	-3.6520	-1.7485	3.6971	-1.5029	-1.3593
H	-5.3200	-0.2437	2.8614	2.8739	-3.3489	-3.1189	4.3701	-0.1572	-3.1625
H	-5.4494	-1.9053	3.5067	1.7542	-4.3650	-4.0598	4.4633	-1.8382	-3.7596
H	-3.5776	-4.2858	-3.9428	-0.5791	-7.8808	2.7712	5.9427	-4.0549	3.8302
H	-5.2829	-3.8936	-3.5786	0.4176	-8.6297	1.4962	7.2753	-3.7790	2.6784
H	-4.0850	-2.5821	-3.7381	1.1184	-7.3605	2.5446	6.4214	-2.3849	3.3969
H	-8.8636	-2.1016	2.6303	5.1051	-5.8090	-4.2588	7.9704	-1.6302	-4.1141

H	-7.6573	-2.8871	1.5748	3.8542	-5.0336	-5.2656	7.2892	-2.4655	-2.6919
H	-7.4867	-2.9866	3.3417	4.7945	-4.0529	-4.1046	6.5443	-2.6890	-4.2909
H	-5.6246	-0.2300	-2.0936	3.0656	-4.8819	1.6702	6.4414	0.1891	1.4284
C	-6.3459	1.1393	0.5038	4.5685	-3.7649	-0.5938	6.0166	1.4989	-1.2464
C	-6.5727	2.0542	-0.6986	5.2840	-3.2325	0.6572	6.3325	2.5489	-0.1807
C	-5.2968	2.8365	-0.9701	5.0735	-1.7316	0.8076	5.0246	3.1388	0.3199
C	-4.7392	3.4731	0.3048	5.3057	-1.0038	-0.5136	4.1760	3.6261	-0.8530
C	-4.7449	2.5354	1.5133	4.5132	-1.6441	-1.6461	4.0817	2.6388	-2.0169
C	-4.4655	3.2787	2.8070	4.8016	-1.0133	-2.9971	3.5990	3.3701	-3.2612
C	-7.6705	1.8000	-2.8119	5.4484	-3.8342	2.9780	7.9397	2.6936	1.5860
C	-2.6776	2.1124	3.7879	2.6494	-0.7032	-3.9099	1.8489	2.1243	-4.1844
O	-6.9056	1.1995	-1.7756	4.7713	-3.9856	1.7365	7.0400	1.8810	0.8441
O	-5.5452	3.8316	-1.9381	5.9546	-1.2269	1.7825	5.2873	4.2048	1.2056
O	-3.3880	3.8391	0.0379	4.8862	0.3351	-0.2926	2.8510	3.8465	-0.3883
O	-6.0137	1.8978	1.6369	4.8988	-3.0154	-1.7316	5.3550	2.0833	-2.3332
O	-4.0575	2.4064	3.8394	3.8761	-1.3985	-3.9909	3.2037	2.5010	-4.2968
H	-7.2443	0.5712	0.7563	4.8893	-4.7893	-0.8057	6.9293	1.0499	-1.6474
H	-7.4029	2.7412	-0.4812	6.3632	-3.4214	0.5450	6.9589	3.3345	-0.6270
H	-4.5440	2.1169	-1.3258	4.0225	-1.5617	1.0903	4.4672	2.3378	0.8255
H	-5.3378	4.3663	0.5387	6.3774	-1.0359	-0.7587	4.6128	4.5683	-1.2189
H	-3.9848	1.7626	1.3707	3.4409	-1.5719	-1.4118	3.3866	1.8339	-1.7385
H	-5.3866	3.7698	3.1368	5.7873	-1.3535	-3.3311	4.4336	3.9714	-3.6374
H	-3.7039	4.0516	2.6200	4.8299	0.0794	-2.8912	2.7799	4.0490	-2.9787
H	-7.9036	1.0085	-3.5250	5.1184	-4.6612	3.6104	8.4411	2.0348	2.2960
H	-8.6051	2.2076	-2.4085	6.5335	-3.8963	2.8363	8.6873	3.1405	0.9201
H	-7.1104	2.5973	-3.3043	5.1976	-2.8785	3.4442	7.4060	3.4834	2.1180
H	-2.4632	1.4023	4.5878	2.0243	-1.0548	-4.7324	1.6434	1.3820	-4.9576
H	-2.3839	1.6601	2.8300	2.1211	-0.8910	-2.9644	1.6364	1.6847	-3.2035
H	-2.0752	3.0194	3.9385	2.8021	0.3808	-4.0117	1.1828	2.9887	-4.3231
H	-4.6731	4.1417	-2.2459	5.7815	-0.2704	1.8637	4.4605	4.3593	1.6997
H	-1.4250	5.7111	3.2277	4.5643	2.3177	-4.0019	0.2758	5.8020	-3.1471
C	3.2072	-0.1247	-3.2686	0.3944	-1.5618	0.0985	2.4037	-1.4066	2.7861
C	-0.7681	-1.1559	-0.3752	1.9827	2.4796	2.9729	-2.7339	-0.2147	2.4480
C	1.5714	2.1099	-3.5516	-1.8828	0.0358	0.2800	1.8843	0.1571	5.0218
C	3.7610	1.1565	-3.2493	-0.4436	-1.4083	-1.0072	3.0237	-1.6991	4.0010
C	-1.6964	1.1593	-1.5731	-0.7255	2.9944	3.0558	-2.0771	1.7180	4.2965
C	-1.4758	-0.2319	0.3865	1.5401	3.7926	3.0965	-3.7193	0.2712	3.2986
C	2.9484	2.2775	-3.3855	-1.5827	-0.6160	-0.9175	2.7605	-0.9245	5.1246
C	-1.9336	0.9351	-0.2200	0.1740	4.0516	3.1475	-3.3905	1.2661	4.2166
C	-0.5004	-5.4135	-3.7071	3.6019	-1.7356	5.6233	-0.8588	0.8377	-2.2626
C	2.0938	-2.6899	-3.8657	1.5310	-2.3925	2.5819	1.6026	-0.4040	0.2609
C	-0.5618	-3.1186	-2.9708	2.3131	-0.2601	4.2531	-1.0381	0.5547	0.0869
C	1.2645	-3.8951	-4.2923	2.0979	-2.6058	3.9714	1.0758	0.3173	-0.9653
C	-0.4805	0.6248	-3.7163	-1.3270	0.5845	2.7006	0.3603	1.6627	3.6588
C	1.8316	-0.2969	-3.4419	0.0869	-0.9256	1.3053	1.5211	-0.3272	2.6835
C	-0.4909	-0.9227	-1.7254	1.0901	1.4040	2.8892	-1.4224	0.2735	2.4824
C	1.0099	0.8385	-3.6044	-1.0619	-0.1170	1.3937	1.2704	0.4728	3.8137
C	-0.9618	0.2485	-2.3337	-0.2906	1.6711	2.9186	-1.0757	1.2284	3.4490
C	0.3193	-1.9907	-2.4291	1.7339	0.0187	2.8577	-0.5351	-0.1833	1.3359
N	0.2878	-4.2695	-3.2675	3.0184	-1.5304	4.3080	-0.3559	0.1081	-1.1155
N	1.2178	-1.5713	-3.5223	0.8532	-1.0985	2.4904	0.9113	0.0590	1.4588
H	3.8543	-0.9842	-3.1184	1.2865	-2.1785	0.0210	2.6274	-2.0196	1.9189
H	-0.3807	-2.0641	0.0839	3.0530	2.2869	2.9291	-2.9840	-0.9832	1.7172
H	0.9220	2.9797	-3.6298	-2.7760	0.6539	0.3553	1.6883	0.7819	5.8891
H	4.8294	1.2655	-3.0880	-0.2112	-1.9124	-1.9410	3.7024	-2.5446	4.0569
H	-2.0530	2.0807	-2.0312	-1.7942	3.1982	3.0795	-1.8093	2.4653	5.0393

H	-1.6423	-0.4134	1.4452	2.2624	4.6010	3.1455	-4.7312	-0.1218	3.2352
H	3.3803	3.2735	-3.3394	-2.2374	-0.5097	-1.7772	3.2389	-1.1494	6.0721
H	-2.4370	1.7068	0.3470	-0.1921	5.0709	3.2227	-4.1467	1.6733	4.8800
H	0.1637	-6.2663	-3.8764	4.1344	-2.6910	5.6417	-0.3826	0.4821	-3.1817
H	-1.2188	-5.6862	-2.9299	4.3157	-0.9361	5.8406	-1.9410	0.6916	-2.3565
H	-1.0411	-5.2021	-4.6459	2.8402	-1.7490	6.4230	-0.6626	1.9230	-2.1638
H	2.7359	-2.3884	-4.6981	0.7864	-3.1638	2.3698	2.6692	-0.1788	0.3673
H	2.7414	-2.9803	-3.0228	2.3554	-2.4959	1.8566	1.4944	-1.4942	0.1101
H	-1.3182	-3.3988	-2.2313	3.0121	0.5408	4.5131	-2.1135	0.3780	-0.0308
H	-1.0930	-2.7594	-3.8728	1.4766	-0.2284	4.9801	-0.8996	1.6442	0.2532
H	0.7500	-3.6628	-5.2420	1.2714	-2.6659	4.7058	1.3084	1.3986	-0.8697
H	1.9319	-4.7461	-4.4639	2.6369	-3.5603	3.9879	1.5873	-0.0671	-1.8569
H	-0.9772	1.5390	-4.0517	-2.3242	1.0379	2.6917	0.4148	2.2958	4.5483
H	-0.6839	-0.1750	-4.4363	-1.2867	-0.1502	3.5127	0.7057	2.2626	2.8115
H	0.9320	-2.4584	-1.6409	2.5880	0.0657	2.1527	-0.7081	-1.2654	1.1747

	FΛ1			FV1		
**atom**	**x**	**y**	**z**	**x**	**y**	**z**
C	5.5125	2.0073	-1.2894	4.0198	4.6299	-0.2456
C	5.4932	2.9590	-0.0880	3.3913	5.2504	1.0077
C	4.0611	3.3680	0.2257	1.8924	4.9858	1.0386
C	3.4241	3.9206	-1.0416	1.2886	5.4285	-0.2880
C	3.5122	2.9204	-2.1921	1.9795	4.7332	-1.4568
C	2.9401	3.4683	-3.4920	1.4123	5.1467	-2.8077
C	6.8022	3.1357	1.9002	4.1543	5.5628	3.2476
C	4.6241	5.0174	-4.1310	2.3098	7.3336	-3.0412
O	6.1121	2.2870	0.9909	4.0568	4.6946	2.1243
O	4.0605	4.3468	1.2408	1.3068	5.6838	2.1114
O	2.0801	4.2283	-0.7130	-0.0888	5.1053	-0.2306
O	4.8896	2.5912	-2.4065	3.3722	5.0614	-1.4165
O	3.2775	4.8148	-3.7434	1.1511	6.5278	-2.9215
H	6.5457	1.8021	-1.5889	5.0600	4.9586	-0.3370
H	6.0703	3.8570	-0.3540	3.5580	6.3369	0.9686
H	3.5038	2.4720	0.5435	1.7427	3.8985	1.1461
H	3.9573	4.8375	-1.3282	1.4155	6.5153	-0.3892
H	2.9562	2.0091	-1.9220	1.8567	3.6436	-1.3491
H	1.8482	3.4348	-3.4422	0.4495	4.6467	-2.9520
H	7.2425	2.4885	2.6605	4.7209	5.0250	4.0084
H	7.6012	3.6826	1.3861	4.6898	6.4810	2.9794
H	6.1141	3.8444	2.3666	3.1627	5.8156	3.6281
H	4.6979	6.0401	-4.5048	1.9760	8.3292	-3.3380
H	5.3178	4.8858	-3.2935	2.8620	7.4008	-2.0977
H	4.9114	4.3194	-4.9282	2.9856	6.9346	-3.8086
H	3.2030	4.8129	1.1961	0.3643	5.8205	1.9017
C	1.5602	5.4463	-1.1830	-1.0094	6.0364	-0.7524
C	0.8759	6.1563	-0.0048	-2.0544	6.2992	0.3357
C	-0.4186	5.4512	0.3866	-2.8506	5.0309	0.5816
C	-1.2510	5.0950	-0.8442	-3.3715	4.4212	-0.7138
C	-0.4225	4.3838	-1.9051	-2.2596	4.2891	-1.7494
C	-1.1890	4.1241	-3.1897	-2.7875	3.8841	-3.1130
C	1.5061	6.9454	2.1730	-2.0371	7.5633	2.3680
C	-0.8549	1.8362	-3.6228	-1.4192	2.0947	-3.7895
O	1.8460	6.1845	1.0204	-1.3344	6.6948	1.4890
O	-1.1824	6.2888	1.2253	-3.9056	5.2832	1.4797
O	-2.3051	4.2649	-0.3713	-3.8358	3.1203	-0.3755
O	0.6703	5.2349	-2.2427	-1.6121	5.5480	-1.9179
O	-0.5580	3.1604	-4.0075	-1.7610	3.4503	-3.9789

H	2.3624	6.0704	-1.5858	-0.4962	6.9576	-1.0383
H	0.6276	7.1827	-0.3180	-2.7175	7.1101	0.0019
H	-0.1473	4.5175	0.9028	-2.1549	4.2948	0.9977
H	-1.6599	6.0210	-1.2739	-4.1904	5.0327	-1.1202
H	-0.0420	3.4367	-1.4956	-1.5366	3.5449	-1.3859
H	-1.2254	5.0579	-3.7597	-3.2517	4.7612	-3.5752
H	-2.2175	3.8218	-2.9560	-3.5572	3.1083	-2.9912
H	2.4235	7.0380	2.7579	-1.3473	7.8139	3.1748
H	1.1550	7.9435	1.8858	-2.3294	8.4818	1.8455
H	0.7342	6.4355	2.7541	-2.9254	7.0737	2.7724
H	-0.3996	1.1744	-4.3613	-0.6523	1.8482	-4.5262
H	-0.4561	1.5860	-2.6281	-1.0194	1.8998	-2.7838
H	-1.9411	1.6625	-3.6148	-2.2910	1.4429	-3.9488
H	-2.0014	5.8088	1.4445	-4.2752	4.4172	1.7347
C	-3.6254	4.6793	-0.5963	-5.2134	2.8918	-0.4064
C	-4.4318	4.3691	0.6651	-5.5858	2.0449	0.8205
C	-4.5405	2.8654	0.8749	-5.0511	0.6218	0.6961
C	-5.0713	2.2335	-0.4063	-5.3770	0.0598	-0.6841
C	-4.2129	2.6168	-1.6080	-4.9236	1.0023	-1.7924
C	-4.7342	2.0220	-2.9098	-5.2231	0.4743	-3.1875
C	-4.7089	5.5418	2.7141	-5.5622	2.3248	3.2058
C	-6.6337	3.3869	-3.3160	-7.5570	0.8943	-3.3601
O	-3.7992	5.0229	1.7491	-5.0677	2.7365	1.9383
O	-5.4076	2.6140	1.9540	-5.6334	-0.1867	1.6951
O	-5.0649	0.8322	-0.2069	-4.6993	-1.1803	-0.7556
O	-4.1842	4.0426	-1.7194	-5.5809	2.2586	-1.6074
O	-6.1354	2.0878	-3.0503	-6.5160	-0.0668	-3.3441
H	-3.6600	5.7528	-0.8081	-5.7654	3.8369	-0.3784
H	-5.4436	4.7747	0.5213	-6.6840	1.9942	0.8808
H	-3.5326	2.4593	1.0663	-3.9531	0.6572	0.8038
H	-6.0986	2.5871	-0.5706	-6.4631	-0.0926	-0.7565
H	-3.1864	2.2505	-1.4463	-3.8342	1.1424	-1.7152
H	-4.4803	0.9564	-2.9335	-4.5258	-0.3438	-3.3968
H	-4.2361	2.5286	-3.7517	-5.0518	1.2850	-3.9129
H	-4.1128	6.0462	3.4755	-5.2476	3.0858	3.9214
H	-5.3866	6.2678	2.2507	-6.6565	2.2613	3.1928
H	-5.2886	4.7353	3.1684	-5.1505	1.3540	3.4911
H	-7.6785	3.2735	-3.6094	-8.4546	0.3848	-3.7140
H	-6.5707	4.0377	-2.4372	-7.7420	1.3123	-2.3654
H	-6.0765	3.8588	-4.1356	-7.3155	1.7190	-4.0429
H	-5.7167	1.6923	1.8800	-5.5862	-1.1127	1.3946
C	-6.1798	0.0986	-0.6563	-5.3190	-2.2427	-1.4436
C	-6.7059	-0.7063	0.5339	-5.5152	-3.3783	-0.4378
C	-5.6853	-1.7576	0.9419	-4.1511	-3.8592	0.0286
C	-5.2405	-2.5677	-0.2747	-3.2490	-4.1792	-1.1598
C	-4.7790	-1.6541	-1.4085	-3.1909	-3.0110	-2.1419
C	-4.4553	-2.4135	-2.6815	-2.4512	-3.3654	-3.4172
C	-7.9781	-0.1426	2.4793	-7.0910	-3.7648	1.3174
C	-2.4749	-1.4551	-3.5237	-1.0984	-1.4863	-3.8445
O	-6.9581	0.2292	1.5626	-6.2774	-2.8305	0.6205
O	-6.2497	-2.6017	1.9156	-4.2903	-4.9805	0.8671
O	-4.1710	-3.3911	0.1695	-1.9584	-4.4261	-0.6169
O	-5.8304	-0.7436	-1.7198	-4.5211	-2.6547	-2.5183
O	-3.8775	-1.5821	-3.6655	-2.2258	-2.2427	-4.2430
H	-6.9490	0.7715	-1.0431	-6.2722	-1.9208	-1.8697
H	-7.6394	-1.2054	0.2354	-6.0628	-4.1995	-0.9219

H	-4.7957	-1.2339	1.3293	-3.6830	-3.0198	0.5680
H	-6.0810	-3.1865	-0.6221	-3.6265	-5.0744	-1.6760
H	-3.8971	-1.0926	-1.0712	-2.7059	-2.1606	-1.6441
H	-5.3897	-2.8074	-3.0975	-3.0686	-4.0662	-3.9907
H	-3.7935	-3.2578	-2.4456	-1.5039	-3.8591	-3.1643
H	-8.1029	0.6976	3.1634	-7.6098	-3.2048	2.0970
H	-8.9224	-0.3189	1.9505	-7.8289	-4.2088	0.6390
H	-7.6993	-1.0407	3.0344	-6.4834	-4.5539	1.7672
H	-2.1224	-0.8476	-4.3596	-0.9379	-0.7250	-4.6109
H	-2.1979	-0.9524	-2.5854	-1.2563	-0.9836	-2.8795
H	-1.9900	-2.4387	-3.5481	-0.2094	-2.1257	-3.7744
H	-5.5914	-3.2872	2.1342	-3.3990	-5.2379	1.1697
C	-4.2887	-4.7829	0.0582	-1.4135	-5.7055	-0.7728
C	-3.7953	-5.3924	1.3730	-0.7157	-6.0783	0.5372
C	-2.3200	-5.0748	1.5445	0.4806	-5.1657	0.7615
C	-1.5409	-5.5089	0.3066	1.3879	-5.2392	-0.4653
C	-2.1505	-4.9483	-0.9784	0.6016	-4.8673	-1.7245
C	-1.5107	-5.5243	-2.2284	1.4154	-4.9793	-3.0003
C	-4.8004	-5.7138	3.5136	-1.4801	-6.8831	2.6521
C	-2.9811	-5.2999	-4.0841	-0.0097	-5.2944	-4.8779
O	-4.5751	-4.8409	2.4137	-1.6782	-5.9863	1.5659
O	-1.8213	-5.7236	2.6875	1.1636	-5.5694	1.9222
O	-0.2146	-5.0295	0.4884	2.4556	-4.3373	-0.2265
O	-3.5414	-5.2740	-1.0247	-0.5126	-5.7547	-1.8496
O	-1.8592	-4.7913	-3.3837	0.7492	-4.3879	-4.0974
H	-5.3277	-5.0715	-0.1298	-2.1971	-6.4360	-0.9990
H	-3.9381	-6.4812	1.3243	-0.3591	-7.1150	0.4492
H	-2.2311	-3.9808	1.6350	0.1161	-4.1285	0.8568
H	-1.5457	-6.6080	0.2598	1.7725	-6.2656	-0.5613
H	-2.0186	-3.8566	-0.9912	0.2442	-3.8313	-1.6249
H	-1.7976	-6.5811	-2.3282	1.6401	-6.0377	-3.1978
H	-0.4243	-5.4751	-2.1145	2.3604	-4.4475	-2.8595
H	-5.4207	-5.1670	4.2242	-2.3740	-6.8239	3.2748
H	-5.3335	-6.6143	3.1870	-1.3674	-7.9107	2.2871
H	-3.8553	-5.9961	3.9822	-0.5978	-6.6023	3.2318
H	-3.1968	-4.5930	-4.8883	-0.5169	-4.7025	-5.6427
H	-2.7565	-6.2825	-4.5182	0.6432	-6.0300	-5.3651
H	-3.8578	-5.3866	-3.4349	-0.7557	-5.8208	-4.2742
H	-0.8637	-5.5415	2.7345	2.0453	-5.1509	1.9130
C	0.8236	-5.9699	0.4723	3.7693	-4.7710	-0.4737
C	1.8026	-5.6413	1.6089	4.6109	-4.4475	0.7654
C	2.6361	-4.4035	1.3054	4.7436	-2.9407	0.9253
C	3.1956	-4.4831	-0.1085	5.2040	-2.3151	-0.3850
C	2.0810	-4.7389	-1.1160	4.3081	-2.7250	-1.5487
C	2.5774	-4.7922	-2.5530	4.7810	-2.1725	-2.8862
C	1.7042	-5.8515	3.9883	4.7662	-5.3256	2.9884
C	3.6034	-6.9352	-2.6247	6.7003	-3.5097	-3.2903
O	1.0338	-5.4911	2.7861	3.9530	-5.0629	1.8530
O	3.6888	-4.3185	2.2404	5.6718	-2.6499	1.9459
O	3.8420	-3.2452	-0.3333	5.1724	-0.9142	-0.1689
O	1.4712	-5.9895	-0.7748	4.2978	-4.1564	-1.6191
O	3.7655	-5.5319	-2.7282	6.1807	-2.2128	-3.0623
H	0.4256	-6.9790	0.6195	3.7878	-5.8482	-0.6682
H	2.4879	-6.4960	1.7099	5.6106	-4.8871	0.6295
H	1.9812	-3.5169	1.3658	3.7500	-2.5351	1.1689
H	3.9215	-5.3068	-0.1591	6.2329	-2.6405	-0.5912

H	1.3315	-3.9361	-1.0464	3.2855	-2.3632	-1.3672
H	2.8089	-3.7713	-2.8716	4.5002	-1.1178	-2.9505
H	1.7728	-5.1905	-3.1900	4.2708	-2.7263	-3.6895
H	0.9743	-5.7521	4.7924	4.1528	-5.9032	3.6811
H	2.0498	-6.8907	3.9397	5.6430	-5.9186	2.7021
H	2.5558	-5.1931	4.1725	5.0964	-4.3982	3.4607
H	4.5211	-7.3905	-3.0006	7.7363	-3.3873	-3.6107
H	3.4355	-7.2528	-1.5905	6.6698	-4.1294	-2.3874
H	2.7554	-7.2759	-3.2330	6.1361	-4.0241	-4.0793
H	4.3785	-3.7476	1.8525	5.9260	-1.7140	1.8474
C	5.0446	-3.2446	-1.0655	6.2230	-0.1409	-0.6853
C	6.1138	-2.5867	-0.1894	6.6953	0.7886	0.4385
C	5.7627	-1.1247	0.0284	5.6191	1.8156	0.7545
C	5.4217	-0.4203	-1.2818	5.0896	2.4805	-0.5121
C	4.4034	-1.2147	-2.0934	4.6881	1.4402	-1.5510
C	4.1789	-0.6441	-3.4807	4.2889	2.0547	-2.8802
C	7.3452	-3.2700	1.7475	7.9853	0.4516	2.4228
C	1.8415	-0.5474	-3.7068	2.2259	1.0533	-3.4019
O	6.1250	-3.3203	1.0212	6.9738	-0.0383	1.5526
O	6.8306	-0.4653	0.6706	6.1274	2.7809	1.6440
O	4.8608	0.8316	-0.9065	3.9525	3.2373	-0.1172
O	4.8839	-2.5466	-2.2694	5.8089	0.5961	-1.8048
O	3.0337	-1.1919	-4.0991	3.6020	1.1375	-3.7055
H	5.3289	-4.2639	-1.3371	7.0383	-0.7819	-1.0313
H	7.0875	-2.6686	-0.6942	7.6082	1.3037	0.1064
H	4.8555	-1.0965	0.6486	4.7751	1.2704	1.2000
H	6.3378	-0.2724	-1.8724	5.8637	3.1386	-0.9327
H	3.4567	-1.2329	-1.5372	3.8573	0.8432	-1.1485
H	5.0396	-0.9046	-4.1052	5.1999	2.3492	-3.4110
H	4.1111	0.4520	-3.4222	3.6842	2.9551	-2.7038
H	7.2289	-3.9451	2.5966	8.1210	-0.3026	3.1988
H	8.1773	-3.6166	1.1232	8.9286	0.5825	1.8793
H	7.5535	-2.2570	2.0992	7.6888	1.4017	2.8718
H	1.0216	-1.0152	-4.2554	1.7830	0.3336	-4.0927
H	1.6442	-0.6457	-2.6305	2.0440	0.7121	-2.3734
H	1.8781	0.5235	-3.9556	1.7369	2.0292	-3.5352
H	6.5495	0.4570	0.8197	5.3943	3.3841	1.8687
H	3.2791	2.8222	-4.3175	2.1086	4.8157	-3.5943
C	3.8001	1.2515	3.0651	1.7756	-1.9802	3.7419
C	-0.9510	1.1424	0.6784	-1.9916	1.5342	4.4967
C	3.0049	-1.4028	3.3480	2.4063	0.1393	2.0561
C	4.7440	0.2266	3.0165	3.0988	-1.5632	3.6072
C	-0.8790	-1.3985	1.7529	-0.8001	2.6622	2.2811
C	-1.6632	0.1175	0.0653	-1.9966	2.9176	4.3504
C	4.3533	-1.1011	3.1559	3.4185	-0.4946	2.7763
C	-1.6212	-1.1684	0.5993	-1.3958	3.4874	3.2325
C	-1.2919	5.0283	4.2017	-4.5932	-2.9753	3.6569
C	1.9807	3.2505	3.9269	-0.8490	-3.0799	3.6018
C	-0.6965	3.0026	3.0880	-2.9683	-1.2264	3.4658
C	0.8633	4.0548	4.5636	-2.2773	-3.4908	3.2882
C	0.5976	-0.6773	3.6509	-0.0370	0.4145	1.4308
C	2.4504	0.9544	3.2844	0.7533	-1.3301	3.0413
C	-0.2119	0.9222	1.8469	-1.3990	0.6960	3.5463
C	2.0562	-0.3891	3.4275	1.0826	-0.2766	2.1662
C	-0.1938	-0.3667	2.4008	-0.7965	1.2722	2.4184
C	0.4278	2.1593	2.4627	-1.5364	-0.7965	3.8082

N	-0.2076	4.2692	3.6047	-3.2191	-2.5811	3.9230
N	1.4658	1.9562	3.4861	-0.6090	-1.7191	3.1201
H	4.1170	2.2811	2.9206	1.5416	-2.8082	4.4022
H	-0.9974	2.1474	0.2589	-2.4554	1.0875	5.3736
H	2.6948	-2.4396	3.4519	2.6391	0.9685	1.3894
H	5.7871	0.4681	2.8449	3.8852	-2.0825	4.1454
H	-0.8227	-2.4004	2.1720	-0.3326	3.1060	1.4041
H	-2.2545	0.3296	-0.8212	-2.4594	3.5425	5.1072
H	5.0898	-1.8979	3.0980	4.4534	-0.1827	2.6666
H	-2.1763	-1.9813	0.1383	-1.3891	4.5654	3.0936
H	-0.9355	6.0229	4.4869	-4.7708	-3.9790	4.0557
H	-2.0953	5.1394	3.4722	-5.2777	-2.2771	4.1488
H	-1.7033	4.5330	5.0994	-4.8263	-2.9916	2.5805
H	2.7699	3.0763	4.6622	-0.1562	-3.7666	3.1049
H	2.4122	3.8230	3.0882	-0.6880	-3.1544	4.6916
H	-1.4576	3.2172	2.3265	-3.6780	-0.5515	3.9546
H	-1.1730	2.3893	3.8792	-3.1125	-1.1198	2.3704
H	0.4983	3.5182	5.4601	-2.4003	-3.5100	2.1907
H	1.2474	5.0297	4.8846	-2.4573	-4.5045	3.6602
H	0.4568	-1.7272	3.9207	0.3616	1.0395	0.6259
H	0.2344	-0.0564	4.4787	-0.7009	-0.3394	0.9914
H	0.8680	2.7451	1.6311	-1.4161	-0.9382	4.8980

	**CR2**			**NR2**			**M2**		
**atom**	**x**	**y**	**z**	**x**	**y**	**z**	**x**	**y**	**z**
C	-3.0346	-3.8483	-2.5247	-4.6905	1.4812	-2.5190	-0.7501	5.9341	1.1733
C	-3.3485	-3.3712	-3.9454	-4.3799	2.4021	-3.6999	-1.8850	6.0556	2.1941
C	-3.3705	-1.8517	-3.9953	-3.1058	3.1890	-3.4242	-2.9324	4.9837	1.9345
C	-4.2922	-1.3131	-2.9053	-3.1859	3.8551	-2.0535	-3.3544	5.0292	0.4711
C	-3.9234	-1.8820	-1.5414	-3.5662	2.8563	-0.9687	-2.1559	4.9577	-0.4696
C	-4.8528	-1.4359	-0.4266	-3.7008	3.4714	0.4169	-2.5428	5.0929	-1.9365
C	-2.8165	-4.2103	-6.1135	-4.6394	2.1973	-6.0593	-1.9665	6.6814	4.4932
C	-6.7002	-2.8875	-0.8119	-5.7461	4.6368	0.1885	-3.0898	7.4005	-2.0472
O	-2.3619	-3.9249	-4.7954	-4.2760	1.5675	-4.8371	-1.3013	5.9390	3.4778
O	-3.8064	-1.4216	-5.2626	-2.9097	4.1654	-4.4212	-4.0506	5.1930	2.7658
O	-4.1075	0.0929	-2.9091	-1.8798	4.3570	-1.8188	-4.2066	3.9119	0.2922
O	-3.9363	-3.3106	-1.5933	-4.7864	2.1957	-1.3140	-1.2341	6.0032	-0.1431
O	-6.2250	-1.5568	-0.7222	-4.3542	4.7202	0.4353	-3.5375	6.0646	-2.1812
H	-3.1510	-4.9353	-2.4699	-5.6634	1.0026	-2.6699	-0.0569	6.7735	1.2894
H	-4.3435	-3.7542	-4.2171	-5.2197	3.1038	-3.8142	-2.3464	7.0469	2.0738
H	-2.3513	-1.4919	-3.7809	-2.2685	2.4732	-3.3988	-2.4703	4.0029	2.1284
H	-5.3337	-1.5650	-3.1480	-3.9117	4.6788	-2.0954	-3.9088	5.9610	0.2939
H	-2.9089	-1.5365	-1.2995	-2.7637	2.1157	-0.9310	-1.6670	3.9820	-0.3282
H	-4.6914	-0.3733	-0.2261	-2.6982	3.6431	0.8193	-2.9572	4.1431	-2.2825
H	-1.9735	-4.6517	-6.6458	-4.5476	1.4419	-6.8402	-1.4079	6.5198	5.4155
H	-3.6446	-4.9286	-6.0890	-5.6769	2.5502	-6.0186	-1.9654	7.7511	4.2524
H	-3.1397	-3.2965	-6.6158	-3.9776	3.0392	-6.2742	-2.9952	6.3352	4.6132
H	-7.7865	-2.8470	-0.7102	-6.1698	5.6184	0.4057	-3.8753	8.0432	-2.4484
H	-6.4403	-3.3519	-1.7691	-5.9621	4.3687	-0.8517	-2.9007	7.6680	-1.0016
H	-6.2835	-3.5123	-0.0107	-6.2132	3.8868	0.8412	-2.1644	7.5618	-2.6155
H	-3.9385	-0.4565	-5.2130	-2.2297	4.7808	-4.0905	-4.7819	4.6563	2.4084
C	-5.2281	0.9332	-2.9432	-1.7408	5.5909	-1.1669	-5.3476	4.0623	-0.5099
C	-4.9277	2.0267	-3.9721	-0.7911	6.4429	-2.0165	-6.5281	3.4879	0.2774
C	-3.7587	2.8721	-3.4856	0.6119	5.8521	-1.9785	-6.3868	1.9768	0.4157
C	-3.9976	3.3456	-2.0543	1.0476	5.5843	-0.5384	-6.0861	1.3347	-0.9395
C	-4.3988	2.2046	-1.1196	0.0106	4.7403	0.1943	-4.8918	2.0149	-1.6047
C	-4.8715	2.7028	0.2374	0.3177	4.5106	1.6685	-4.5869	1.4943	-2.9892

C	-4.9324	2.1233	-6.3593	-1.0368	7.6173	-4.0804	-7.7993	4.2512	2.1516
C	-4.0572	1.2258	1.8881	0.6915	2.2102	1.9870	-3.0070	1.6533	-4.7057
O	-4.6455	1.3678	-5.1902	-1.3364	6.4545	-3.3209	-6.5272	4.1481	1.5275
O	-3.5847	3.9836	-4.3340	1.5170	6.7305	-2.6063	-7.5745	1.4487	0.9549
O	-2.7682	3.9339	-1.6589	2.2766	4.8756	-0.6123	-5.8296	-0.0369	-0.6726
O	-5.4971	1.4928	-1.6864	-1.2451	5.4176	0.1345	-5.1937	3.4030	-1.7378
O	-5.1613	1.6305	1.1064	-0.2223	3.2808	2.1204	-3.3613	2.0550	-3.4018
H	-6.1247	0.3729	-3.2273	-2.7126	6.0796	-1.0560	-5.5124	5.1161	-0.7505
H	-5.8192	2.6615	-4.0794	-0.7673	7.4617	-1.6027	-7.4556	3.7100	-0.2705
H	-2.8633	2.2315	-3.4800	0.5780	4.8806	-2.4962	-5.5252	1.7782	1.0734
H	-4.7940	4.1054	-2.0724	1.1832	6.5394	-0.0106	-6.9696	1.4388	-1.5863
H	-3.5371	1.5303	-0.9983	-0.0652	3.7656	-0.3102	-3.9997	1.8834	-0.9770
H	-5.8001	3.2666	0.0905	-0.1516	5.3123	2.2460	-5.3982	1.7844	-3.6763
H	-4.1159	3.3692	0.6732	1.4013	4.5501	1.8416	-4.5326	0.3966	-2.9691
H	-4.7325	1.4705	-7.2097	-1.5604	7.5128	-5.0314	-7.6558	4.8435	3.0561
H	-5.9876	2.4212	-6.3747	-1.4002	8.5159	-3.5674	-8.5095	4.7666	1.4937
H	-4.3029	3.0139	-6.4149	0.0379	7.7081	-4.2528	-8.1938	3.2656	2.4078
H	-4.3149	0.2617	2.3316	0.1433	1.2798	2.1534	-2.0747	2.1583	-4.9617
H	-3.1455	1.1090	1.2833	1.1289	2.1731	0.9794	-2.8564	0.5661	-4.7600
H	-3.8421	1.9451	2.6935	1.5031	2.2897	2.7230	-3.7800	1.9350	-5.4330
H	-2.9936	4.6098	-3.8771	2.3859	6.2883	-2.6311	-7.5142	0.4763	0.9127
C	-2.7983	5.0939	-0.8731	3.4365	5.5294	-0.1771	-6.5185	-1.0074	-1.4145
C	-1.9255	6.1471	-1.5639	4.5679	5.1687	-1.1437	-7.0516	-2.0554	-0.4315
C	-0.4523	5.7611	-1.4936	4.8748	3.6801	-1.0598	-5.9009	-2.8284	0.1956
C	-0.0873	5.3915	-0.0588	5.0840	3.2695	0.3918	-4.9971	-3.3618	-0.9078
C	-1.0144	4.3042	0.4711	3.9509	3.7501	1.2930	-4.5332	-2.2457	-1.8392
C	-0.6954	3.8726	1.8909	4.1902	3.4460	2.7653	-3.6761	-2.7637	-2.9865
C	-2.1989	7.4999	-3.5150	5.1974	5.9552	-3.3057	-8.9218	-2.0971	1.0521
C	-1.6750	5.5656	3.2328	5.7922	5.1155	3.3113	-5.3115	-3.8088	-4.3556
O	-2.4062	6.2409	-2.8891	4.1444	5.5537	-2.4373	-7.8214	-1.3642	0.5301
O	0.3286	6.8435	-1.9406	6.0279	3.3859	-1.8159	-6.4160	-3.8885	0.9658
O	1.2655	4.9658	-0.0786	5.1318	1.8516	0.3719	-3.9010	-3.9814	-0.2589
O	-2.3478	4.8249	0.4284	3.7628	5.1586	1.1389	-5.6900	-1.5984	-2.3830
O	-0.4855	4.9366	2.7909	5.4940	3.7334	3.2196	-4.1497	-3.9617	-3.5610
H	-3.8235	5.4662	-0.7730	3.2895	6.6142	-0.1635	-7.3502	-0.5535	-1.9632
H	-2.0599	7.1060	-1.0415	5.4610	5.7387	-0.8486	-7.6863	-2.7571	-0.9925
H	-0.3007	4.8704	-2.1252	3.9984	3.1341	-1.4427	-5.3161	-2.1310	0.8166
H	-0.1943	6.2810	0.5772	6.0428	3.6793	0.7386	-5.5582	-4.1043	-1.4917
H	-0.9502	3.4142	-0.1713	3.0371	3.2272	0.9834	-3.9425	-1.5144	-1.2677
H	0.2367	3.2980	1.8761	4.0486	2.3713	2.9161	-2.6760	-2.9911	-2.6067
H	-1.5057	3.2162	2.2445	3.4421	3.9927	3.3602	-3.5968	-1.9715	-3.7473
H	-2.6884	7.4476	-4.4882	4.7318	6.2478	-4.2472	-9.4366	-1.4339	1.7481
H	-2.6574	8.3019	-2.9242	5.7362	6.8132	-2.8868	-9.6108	-2.3805	0.2478
H	-1.1341	7.7072	-3.6411	5.8956	5.1323	-3.4733	-8.5803	-2.9941	1.5731
H	-1.4092	6.1853	4.0913	6.7184	5.2035	3.8812	-5.4412	-4.7376	-4.9136
H	-2.1211	6.1919	2.4540	5.9264	5.5708	2.3245	-6.2035	-3.6223	-3.7476
H	-2.4169	4.8176	3.5460	4.9913	5.6533	3.8344	-5.1961	-2.9758	-5.0614
H	1.2521	6.6723	-1.6785	6.3821	2.5395	-1.4876	-5.6852	-4.5102	1.1388
C	2.1311	5.4999	0.8901	5.9730	1.2139	1.3015	-3.4648	-5.2271	-0.7405
C	3.3977	5.9762	0.1665	7.0147	0.4101	0.5143	-3.3500	-6.1656	0.4661
C	4.1596	4.7787	-0.3777	6.3592	-0.7768	-0.1757	-2.2280	-5.6966	1.3804
C	4.3976	3.7709	0.7417	5.4940	-1.5566	0.8125	-0.9485	-5.4771	0.5769
C	3.0893	3.3812	1.4226	4.4832	-0.6337	1.4864	-1.1925	-4.5561	-0.6163
C	3.3101	2.5072	2.6476	3.6179	-1.3231	2.5367	0.0273	-4.4415	-1.5114
C	3.8988	7.8971	-1.1588	8.9325	1.0352	-0.7674	-4.9438	-7.3438	1.8002
C	2.1694	0.4830	2.3062	1.4477	-1.3703	1.6676	0.1919	-2.1186	-1.8896

O	2.9797	6.8567	-0.8555	7.5884	1.3191	-0.4049	-4.6103	-6.1505	1.1049
O	5.3870	5.1981	-0.9292	7.3531	-1.6105	-0.7265	-2.0072	-6.6503	2.3937
O	4.9933	2.6254	0.1510	4.8272	-2.5690	0.0709	-0.0037	-4.9027	1.4703
O	2.4362	4.5631	1.8845	5.2191	0.3962	2.1546	-2.2400	-5.1122	-1.4099
O	2.1701	1.7339	2.9679	2.3186	-0.7637	2.6053	-0.0644	-3.3920	-2.4533
H	1.6524	6.3369	1.4069	6.4607	1.9531	1.9433	-4.1734	-5.6236	-1.4729
H	4.0289	6.5095	0.8924	7.7787	0.0412	1.2143	-3.1209	-7.1773	0.1000
H	3.5291	4.2939	-1.1390	5.6920	-0.3842	-0.9594	-2.5254	-4.7256	1.8063
H	5.0733	4.2107	1.4907	6.1313	-2.0079	1.5874	-0.5839	-6.4478	0.2090
H	2.4496	2.8581	0.6959	3.8339	-0.1867	0.7206	-1.4918	-3.5660	-0.2451
H	3.5111	3.1669	3.4984	4.0923	-1.1834	3.5133	0.1195	-5.3728	-2.0812
H	4.1823	1.8601	2.5010	3.5577	-2.3996	2.3330	0.9236	-4.3210	-0.8925
H	3.4341	8.5088	-1.9329	9.2531	1.8403	-1.4296	-5.9539	-7.2070	2.1881
H	4.0835	8.5168	-0.2732	9.5753	1.0233	0.1210	-4.9345	-8.2014	1.1168
H	4.8442	7.4885	-1.5221	9.0072	0.0747	-1.2818	-4.2483	-7.5267	2.6220
H	1.2596	-0.0458	2.5997	0.4891	-0.8505	1.6981	0.3144	-1.4137	-2.7159
H	2.1743	0.5986	1.2122	1.8565	-1.3166	0.6460	-0.6324	-1.7767	-1.2485
H	3.0435	-0.1087	2.6056	1.2845	-2.4252	1.9276	1.1206	-2.1361	-1.3031
H	5.7992	4.4216	-1.3511	6.9038	-2.2993	-1.2509	-1.3128	-6.2980	2.9811
C	6.3538	2.4160	0.4114	5.2898	-3.8808	0.2481	1.1194	-5.6701	1.8012
C	7.0014	1.8738	-0.8644	5.2662	-4.5930	-1.1076	1.4148	-5.4687	3.2903
C	6.4331	0.4996	-1.1802	3.8314	-4.7631	-1.5776	1.8787	-4.0432	3.5501
C	6.5068	-0.4151	0.0419	2.9669	-5.3872	-0.4838	3.0106	-3.6656	2.5956
C	5.9128	0.2533	1.2812	3.1204	-4.6375	0.8400	2.6095	-3.9420	1.1493
C	6.1076	-0.5462	2.5575	2.3972	-5.2890	2.0059	3.7136	-3.6885	0.1429
C	7.7507	2.8831	-2.8892	6.6948	-4.5587	-3.0172	0.4361	-6.3374	5.2862
C	5.9667	0.9395	4.4150	3.4150	-4.5663	4.0346	3.3483	-4.9052	-1.8756
O	6.7392	2.8112	-1.8912	6.0317	-3.8110	-2.0042	0.2282	-5.7794	3.9943
O	7.1335	-0.0821	-2.2547	3.7894	-5.5671	-2.7338	2.3036	-3.9224	4.8857
O	5.7488	-1.5499	-0.3453	1.6532	-5.2758	-1.0079	3.2247	-2.2785	2.8041
O	6.5334	1.5243	1.4808	4.5060	-4.5726	1.1847	2.2305	-5.3133	1.0192
O	5.3279	-0.0493	3.6265	2.3272	-4.4420	3.1357	3.1924	-3.6845	-1.1733
H	6.8405	3.3513	0.7060	6.3104	-3.8787	0.6440	0.9349	-6.7307	1.6015
H	8.0836	1.7916	-0.6883	5.7266	-5.5827	-0.9754	2.2182	-6.1646	3.5722
H	5.3673	0.6304	-1.4250	3.4269	-3.7581	-1.7778	1.0321	-3.3706	3.3376
H	7.5564	-0.6944	0.2188	3.2449	-6.4451	-0.3638	3.9117	-4.2393	2.8580
H	4.8368	0.3853	1.1029	2.7251	-3.6240	0.6972	1.7570	-3.2933	0.9000
H	7.1760	-0.5535	2.8203	2.8947	-6.2380	2.2563	4.4999	-4.4488	0.2546
H	5.7815	-1.5744	2.3789	1.3675	-5.4990	1.7053	4.1553	-2.7045	0.3312
H	7.4334	3.6445	-3.6023	7.2645	-3.8434	-3.6109	-0.5509	-6.5641	5.6906
H	8.7091	3.1793	-2.4467	7.3820	-5.2874	-2.5713	1.0194	-7.2632	5.2174
H	7.8636	1.9217	-3.3948	5.9716	-5.0778	-3.6496	0.9524	-5.6274	5.9354
H	5.2954	1.1614	5.2467	3.1896	-3.9301	4.8927	2.8325	-4.7834	-2.8309
H	6.9177	0.5631	4.8135	3.5194	-5.6042	4.3767	4.4107	-5.1128	-2.0626
H	6.1570	1.8550	3.8463	4.3581	-4.2453	3.5812	2.9044	-5.7410	-1.3256
H	6.8587	-1.0162	-2.3046	2.8554	-5.8009	-2.8853	2.7256	-3.0483	4.9814
C	6.1545	-2.8312	0.0428	0.7099	-6.2767	-0.7553	4.5293	-1.7929	2.9705
C	6.0878	-3.7185	-1.2048	0.0254	-6.6023	-2.0890	4.5069	-0.8500	4.1773
C	4.6387	-3.9101	-1.6435	-0.8451	-5.4324	-2.5377	3.6673	0.3865	3.8728
C	3.7646	-4.2942	-0.4526	-1.7129	-4.9246	-1.3881	4.1086	0.9882	2.5443
C	3.9409	-3.3188	0.7061	-0.8614	-4.6139	-0.1612	4.1142	-0.0520	1.4279
C	3.0964	-3.6523	1.9266	-1.6174	-4.0755	1.0427	4.6083	0.5222	0.1145
C	7.3819	-3.9151	-3.2031	0.6886	-7.6976	-4.1028	4.4402	-1.1839	6.5436
C	4.2516	-5.5230	2.8157	-2.7539	-5.9526	1.9275	6.9496	0.5112	-0.2174
O	6.8774	-3.0682	-2.1795	1.0693	-6.8938	-2.9948	3.9956	-1.6011	5.2596
O	4.5729	-4.9150	-2.6301	-1.6724	-5.8290	-3.6097	3.8226	1.3195	4.9175

O	2.4371	-4.2980	-0.9463	-2.3724	-3.7930	-1.9259	3.1979	2.0382	2.2541
O	5.3277	-3.3310	1.0647	-0.2255	-5.8477	0.2021	4.9770	-1.1240	1.8180
O	3.0511	-5.0229	2.2547	-2.8591	-4.6999	1.2775	5.8256	1.2197	0.2734
H	7.1726	-2.8146	0.4463	1.1894	-7.1716	-0.3441	5.2313	-2.6152	3.1433
H	6.5157	-4.7018	-0.9587	-0.6155	-7.4849	-1.9449	5.5386	-0.5333	4.3885
H	4.2739	-2.9456	-2.0321	-0.1742	-4.6111	-2.8379	2.6165	0.0702	3.7753
H	4.0477	-5.3027	-0.1195	-2.4474	-5.6976	-1.1208	5.1253	1.3860	2.6572
H	3.6604	-2.3075	0.3801	-0.0926	-3.8728	-0.4210	3.0937	-0.4323	1.2763
H	2.0648	-3.3596	1.7127	-1.8285	-3.0158	0.8790	3.8525	1.2283	-0.2495
H	3.4682	-3.0584	2.7763	-0.9629	-4.1611	1.9233	4.7118	-0.2888	-0.6208
H	8.0499	-3.3044	-3.8114	1.6020	-7.9198	-4.6555	4.0350	-1.8986	7.2607
H	7.9497	-4.7466	-2.7685	0.2378	-8.6368	-3.7596	5.5353	-1.2034	6.5953
H	6.5740	-4.3152	-3.8197	-0.0209	-7.1738	-4.7473	4.0843	-0.1779	6.7748
H	4.0334	-6.5205	3.2002	-3.7668	-6.2653	2.1869	7.8323	1.1122	0.0099
H	5.0545	-5.5855	2.0734	-2.2906	-6.7081	1.2840	7.0439	-0.4698	0.2629
H	4.5926	-4.8837	3.6405	-2.1582	-5.8632	2.8466	6.8777	0.3685	-1.3038
H	3.6461	-5.2125	-2.6831	-2.3511	-5.1373	-3.7134	3.5950	2.2003	4.5678
C	1.5614	-5.2864	-0.4647	-3.6856	-3.5403	-1.5109	3.7179	3.2200	1.6927
C	0.9648	-6.0051	-1.6810	-4.4882	-3.1141	-2.7520	3.3870	4.3703	2.6504
C	0.0286	-5.0745	-2.4385	-4.1030	-1.7056	-3.2015	1.8800	4.5720	2.6992
C	-0.9609	-4.4244	-1.4740	-4.0791	-0.7637	-1.9981	1.3175	4.7068	1.2865
C	-0.2203	-3.7285	-0.3388	-3.1195	-1.3250	-0.9592	1.7495	3.5465	0.3960
C	-1.1281	-3.0859	0.6999	-2.8373	-0.4594	0.2650	1.3574	3.7599	-1.0565
C	1.8374	-7.5966	-3.2344	-5.2834	-4.2681	-4.6811	4.2856	5.0856	4.7471
C	-0.8561	-0.7746	0.4084	-0.8183	0.5764	-0.3973	0.3566	1.7220	-1.7315
O	2.0636	-6.4239	-2.4651	-4.2378	-4.0959	-3.7355	3.9211	4.0002	3.9051
O	-0.6560	-5.7970	-3.4368	-5.0176	-1.2444	-4.1718	1.5704	5.7199	3.4563
O	-1.7166	-3.4794	-2.2182	-3.6728	0.5143	-2.4573	-0.0972	4.7165	1.4054
O	0.5646	-4.7142	0.3372	-3.7067	-2.5514	-0.5167	3.1734	3.4517	0.4211
O	-0.5555	-1.8933	1.2163	-1.4465	-0.3231	0.4932	1.4753	2.5831	-1.8246
H	2.0961	-5.9910	0.1769	-4.1248	-4.4336	-1.0578	4.7982	3.1304	1.5447
H	0.3947	-6.8765	-1.3263	-5.5547	-3.1082	-2.4790	3.8711	5.2876	2.2846
H	0.6375	-4.2699	-2.8800	-3.0818	-1.7469	-3.6128	1.4369	3.6701	3.1488
H	-1.6220	-5.1929	-1.0477	-5.0813	-0.7026	-1.5492	1.6728	5.6497	0.8446
H	0.4415	-2.9599	-0.7622	-2.1545	-1.5430	-1.4365	1.3082	2.6165	0.7796
H	-1.2672	-3.7699	1.5433	-3.2453	-0.9515	1.1486	2.0459	4.4954	-1.4883
H	-2.1160	-2.8769	0.2640	-3.3269	0.5203	0.1718	0.3393	4.1675	-1.1106
H	2.7779	-7.8267	-3.7364	-4.9907	-5.0996	-5.3232	4.7225	4.6487	5.6459
H	1.5629	-8.4357	-2.5837	-6.2238	-4.5191	-4.1749	5.0338	5.7174	4.2539
H	1.0482	-7.4368	-3.9722	-5.4270	-3.3660	-5.2801	3.4142	5.6896	5.0082
H	-0.3066	0.0859	0.7969	0.2528	0.3641	-0.3849	0.5889	0.8524	-2.3509
H	-0.5694	-0.9377	-0.6399	-1.1917	0.4663	-1.4246	0.1720	1.3965	-0.6989
H	-1.9328	-0.5530	0.4475	-0.9769	1.6148	-0.0721	-0.5524	2.2119	-2.1108
H	-1.1572	-5.1540	-3.9722	-4.6983	-0.3785	-4.4854	0.6005	5.7527	3.5536
H	-4.5940	-2.0059	0.4808	-4.2239	2.7490	1.0649	-1.6308	5.3166	-2.5121
C	-6.1378	-1.9087	2.7749	-2.6606	-2.7737	4.0231	5.8252	-2.2477	-2.4546
C	-1.3869	0.0416	3.7409	-5.4006	1.3599	5.7833	4.6369	2.7251	-2.5099
C	-6.5606	0.2487	4.4709	-0.7743	-0.7332	4.1172	8.1213	-1.0301	-3.4467
C	-7.1980	-1.0346	2.5386	-1.3456	-3.0530	4.3981	7.0828	-2.6668	-2.0192
C	-2.7931	1.4056	5.6755	-3.0390	2.6369	5.1359	7.0038	2.8622	-3.9230
C	-0.9378	1.2918	4.1522	-5.0072	2.4813	6.5071	5.4477	3.8481	-2.3805
C	-7.4082	0.0511	3.3808	-0.3978	-2.0367	4.4451	8.2339	-2.0592	-2.5097
C	-1.6528	1.9839	5.1272	-3.8207	3.1298	6.1765	6.6371	3.9226	-3.0985
C	-0.7933	-5.1086	4.1975	-7.8721	-0.1170	1.2432	0.6364	0.3940	-5.1898
C	-4.2684	-3.8854	3.5787	-5.0595	-2.2305	2.5303	3.3872	-1.7608	-3.8487
C	-1.7308	-2.9035	4.2752	-6.2241	0.3187	2.9090	2.8201	0.9579	-4.3765

C	-3.1845	-4.7825	4.1481	-6.0491	-1.6300	1.5434	2.2495	-1.3357	-4.7617
C	-4.5300	-0.4045	5.8490	-2.5089	0.9539	3.3442	6.7011	0.5438	-4.8531
C	-5.2658	-1.6952	3.8465	-3.0387	-1.4696	3.6877	5.7112	-1.2131	-3.3877
C	-2.5252	-0.5576	4.2931	-4.6310	0.8661	4.7258	4.9868	1.6618	-3.3482
C	-5.5005	-0.6176	4.7186	-2.0757	-0.4385	3.7245	6.8747	-0.6081	-3.8981
C	-3.2362	0.1381	5.2824	-3.4281	1.5125	4.4046	6.1960	1.7306	-4.0602
C	-2.8357	-1.9604	3.7717	-5.2358	-0.2755	3.9239	3.9673	0.5352	-3.4518
N	-1.8859	-4.2549	3.7646	-6.9679	-0.7174	2.2102	1.7482	-0.0297	-4.3574
N	-4.1495	-2.5347	4.1249	-4.3214	-1.1548	3.1843	4.4650	-0.7761	-3.9068
H	-5.9931	-2.7538	2.1118	-3.3895	-3.5765	3.9736	4.9275	-2.7148	-2.0618
H	-0.8483	-0.5072	2.9737	-6.3300	0.8553	6.0376	3.7089	2.6654	-1.9488
H	-6.7245	1.0859	5.1445	-0.0300	0.0597	4.1322	9.0143	-0.5510	-3.8398
H	-7.8443	-1.2043	1.6835	-1.0647	-4.0726	4.6467	7.1547	-3.4674	-1.2886
H	-3.3624	1.9441	6.4286	-2.1034	3.1263	4.8769	7.9440	2.9009	-4.4668
H	-0.0351	1.7160	3.7206	-5.6235	2.8430	7.3236	5.1502	4.6489	-1.7103
H	-8.2282	0.7376	3.1977	0.6333	-2.2526	4.7058	9.2131	-2.3826	-2.1722
H	-1.3260	2.9663	5.4545	-3.5001	4.0061	6.7304	7.2847	4.7890	-3.0078
H	-0.9392	-6.1165	3.7997	-8.4437	-0.8991	0.7361	-0.1793	-0.3320	-5.1126
H	0.1536	-4.7207	3.8089	-8.5717	0.5518	1.7520	0.2721	1.3648	-4.8404
H	-0.7181	-5.1764	5.2968	-7.3290	0.4676	0.4765	0.9162	0.4908	-6.2535
H	-5.2566	-4.2747	3.8370	-4.3538	-2.8673	1.9866	3.7835	-2.7255	-4.1796
H	-4.1705	-3.8902	2.4777	-5.6049	-2.8534	3.2607	3.0009	-1.8891	-2.8241
H	-0.7641	-2.5177	3.9369	-6.9329	0.9683	3.4330	2.4173	1.9099	-4.0169
H	-1.7423	-2.8830	5.3834	-5.6432	0.9488	2.2022	3.2248	1.1033	-5.3976
H	-3.2927	-4.8409	5.2475	-5.4842	-1.1052	0.7498	2.6050	-1.3210	-5.8092
H	-3.2942	-5.7924	3.7404	-6.6245	-2.4296	1.0638	1.4356	-2.0665	-4.6863
H	-4.9388	0.3019	6.5757	-1.6469	1.6138	3.2264	7.6506	0.7937	-5.3329
H	-4.3497	-1.3592	6.3543	-3.0334	0.9113	2.3828	5.9784	0.2640	-5.6272
H	-2.7512	-1.9202	2.6670	-5.8264	-0.8904	4.6271	3.5151	0.4164	-2.4505

	**FΛ2**			**FV2**			**S**		
**atom**	**x**	**y**	**z**	**x**	**y**	**z**	**x**	**y**	**z**
C	-4.0651	-3.2745	-2.5314	-4.6914	-1.4771	2.5198	-4.1750	0.5147	1.1843
C	-4.4470	-2.5989	-3.8524	-4.3813	-2.3977	3.7010	-4.3159	-0.3203	2.4535
C	-4.1290	-1.1096	-3.8092	-3.1079	-3.1857	3.4254	-3.2534	-1.4057	2.4991
C	-4.6815	-0.4878	-2.5296	-3.1887	-3.8523	2.0550	-3.3091	-2.1997	1.1997
C	-4.2026	-1.2587	-1.3084	-3.5685	-2.8537	0.9698	-3.1958	-1.2961	-0.0253
C	-4.6862	-0.7169	0.0252	-3.7039	-3.4693	-0.4155	-3.3615	-2.0577	-1.3350
C	-4.4038	-3.3034	-6.1286	-4.6401	-2.1918	6.0605	-5.0644	0.2082	4.6473
C	-6.9488	-1.4434	-0.0262	-5.7501	-4.6332	-0.1859	-5.6900	-2.5579	-1.2426
O	-3.7411	-3.2785	-4.8709	-4.2765	-1.5628	4.8379	-4.2359	0.5669	3.5501
O	-4.6840	-0.4669	-4.9339	-2.9123	-4.1619	4.4227	-3.4910	-2.2522	3.5988
O	-4.1942	0.8399	-2.5308	-1.8830	-4.3553	1.8203	-2.2398	-3.1264	1.2510
O	-4.6413	-2.6153	-1.4334	-4.7881	-2.1920	1.3152	-4.2213	-0.2999	0.0389
O	-6.0489	-0.3508	0.0604	-4.3582	-4.7176	-0.4332	-4.3646	-3.0495	-1.3020
H	-4.4590	-4.2960	-2.5154	-5.6638	-0.9976	2.6707	-5.0371	1.1816	1.1197
H	-5.5316	-2.7232	-3.9899	-5.2216	-3.0987	3.8158	-5.3131	-0.7845	2.4209
H	-3.0328	-0.9982	-3.7881	-2.2700	-2.4706	3.3996	-2.2657	-0.9224	2.5721
H	-5.7799	-0.4920	-2.5695	-3.9151	-4.6755	2.0974	-4.2679	-2.7344	1.1648
H	-3.1030	-1.2310	-1.2971	-2.7654	-2.1137	0.9316	-2.2104	-0.8049	-0.0235
H	-4.1240	0.1885	0.2734	-2.7016	-3.6418	-0.8181	-2.4319	-2.5858	-1.5653
H	-3.7728	-3.8824	-6.8035	-4.5476	-1.4362	6.8410	-4.9824	1.0111	5.3812
H	-5.3814	-3.7924	-6.0401	-5.6779	-2.5441	6.0201	-6.1082	0.1197	4.3206
H	-4.5378	-2.2918	-6.5182	-3.9788	-3.0341	6.2755	-4.7375	-0.7374	5.0863
H	-7.9298	-1.0741	0.2767	-6.1745	-5.6146	-0.4026	-6.3542	-3.3741	-1.5346
H	-7.0052	-1.8441	-1.0436	-5.9654	-4.3645	0.8543	-5.9623	-2.2288	-0.2305
H	-6.6418	-2.2575	0.6445	-6.2169	-3.8830	-0.8387	-5.8356	-1.7089	-1.9257

H	-4.6721	0.4908	-4.7526	-2.2328	-4.7780	4.0922	-2.9676	-3.0637	3.4643
C	-5.0236	1.8612	-2.0443	-1.7450	-5.5895	1.1688	-2.4914	-4.4498	0.8539
C	-4.9727	2.9934	-3.0764	-0.7957	-6.4419	2.0184	-1.9113	-5.3644	1.9367
C	-3.5779	3.6061	-3.1024	0.6078	-5.8521	1.9797	-0.3934	-5.2530	1.9532
C	-3.1071	3.9413	-1.6878	1.0432	-5.5852	0.5393	0.1721	-5.4345	0.5458
C	-3.2358	2.7317	-0.7697	0.0066	-4.7407	-0.1933	-0.5134	-4.5026	-0.4503
C	-2.8518	3.0184	0.6624	0.3133	-4.5117	-1.6676	-0.0941	-4.7605	-1.8859
C	-5.9260	3.3033	-5.2429	-1.0420	-7.6156	4.0826	-2.6338	-5.9886	4.1262
C	-1.9453	2.0189	2.5611	0.6873	-2.2116	-1.9876	0.3361	-2.6235	-2.7719
O	-5.3305	2.4094	-4.3132	-1.3405	-6.4525	3.3231	-2.4938	-4.9578	3.1586
O	-3.5792	4.7666	-3.9017	1.5124	-6.7309	2.6076	0.1409	-6.2224	2.8228
O	-1.7438	4.3274	-1.7931	2.2727	-4.8775	0.6127	1.5585	-5.1386	0.6387
O	-4.5964	2.2999	-0.7826	-1.2496	-5.4170	-0.1328	-1.9208	-4.7251	-0.3955
O	-2.4541	1.8063	1.2618	-0.2267	-3.2821	-2.1198	-0.5272	-3.7392	-2.7600
H	-6.0465	1.4979	-1.9105	-2.7172	-6.0776	1.0584	-3.5659	-4.6176	0.7389
H	-5.7034	3.7642	-2.7905	-0.7728	-7.4609	1.6051	-2.1950	-6.4015	1.7054
H	-2.8893	2.8475	-3.5058	0.5748	-4.8804	2.4970	-0.1356	-4.2344	2.2842
H	-3.7115	4.7680	-1.2874	1.1780	-6.5407	0.0119	0.0230	-6.4786	0.2329
H	-2.5840	1.9293	-1.1409	-0.0683	-3.7658	0.3110	-0.2894	-3.4633	-0.1706
H	-3.7050	3.4614	1.1979	-0.1563	-5.3136	-2.2446	-0.5664	-5.6903	-2.2191
H	-2.0260	3.7440	0.6804	1.3968	-4.5514	-1.8410	0.9959	-4.8867	-1.9377
H	-6.2044	2.7087	-6.1138	-1.5646	-7.5100	5.0341	-3.1411	-5.5451	4.9838
H	-6.8281	3.7580	-4.8156	-1.4074	-8.5137	3.5702	-3.2468	-6.8069	3.7298
H	-5.2278	4.0910	-5.5347	0.0327	-7.7080	4.2541	-1.6592	-6.3781	4.4281
H	-1.5766	1.0601	2.9339	0.1392	-1.2813	-2.1546	-0.0457	-1.9244	-3.5183
H	-1.1135	2.7382	2.5323	1.1252	-2.1737	-0.9802	0.3765	-2.1142	-1.7986
H	-2.7185	2.3998	3.2382	1.4987	-2.2918	-2.7239	1.3576	-2.9216	-3.0493
H	-2.6598	5.0892	-3.9477	2.3817	-6.2896	2.6316	1.1129	-6.1627	2.7685
C	-1.4265	5.6636	-1.5214	3.4319	-5.5320	0.1766	2.4826	-6.1094	0.2329
C	-0.3776	6.1105	-2.5441	4.5641	-5.1723	1.1426	3.6029	-6.1461	1.2769
C	0.9321	5.3698	-2.3160	4.8722	-3.6840	1.0588	4.3877	-4.8424	1.2543
C	1.3392	5.5140	-0.8553	5.0810	-3.2733	-0.3928	4.8258	-4.5437	-0.1734
C	0.2241	5.0698	0.0859	3.9468	-3.7526	-1.2933	3.6446	-4.5569	-1.1400
C	0.5804	5.2746	1.5532	4.1854	-3.4482	-2.7657	4.0685	-4.3257	-2.5852
C	-0.5049	6.7876	-4.8278	5.1942	-5.9607	3.3036	3.7860	-7.1343	3.4413
C	0.3959	7.6352	1.7442	5.7858	-5.1187	-3.3134	5.0916	-6.4036	-3.1085
O	-0.9186	5.8641	-3.8277	4.1411	-5.5570	2.4364	2.9946	-6.3816	2.5307
O	1.9277	5.9050	-3.1558	6.0258	-3.3909	1.8145	5.5100	-4.9570	2.0973
O	2.4964	4.7136	-0.6871	5.1301	-1.8553	-0.3728	5.4490	-3.2725	-0.1362
O	-0.9597	5.8207	-0.2046	3.7577	-5.1610	-1.1395	2.9931	-5.8289	-1.0472
O	1.2335	6.4960	1.8207	5.4887	-3.7364	-3.2210	5.2482	-5.0046	-2.9551
H	-2.3162	6.2964	-1.6039	3.2841	-6.6167	0.1627	2.0056	-7.0923	0.1605
H	-0.2048	7.1879	-2.4050	5.4567	-5.7429	0.8469	4.2804	-6.9740	1.0214
H	0.7639	4.3014	-2.5261	3.9964	-3.1373	1.4423	3.7187	-4.0335	1.5885
H	1.5785	6.5688	-0.6622	6.0391	-3.6840	-0.7403	5.5506	-5.3096	-0.4819
H	0.0287	3.9985	-0.0779	3.0336	-3.2290	-0.9830	2.9354	-3.7634	-0.8570
H	1.2803	4.4924	1.8618	4.0445	-2.3734	-2.9161	4.2814	-3.2612	-2.7248
H	-0.3424	5.1880	2.1486	3.4365	-3.9942	-3.3603	3.2331	-4.6147	-3.2428
H	-1.0135	6.4996	-5.7484	4.7292	-6.2521	4.2458	3.1878	-7.2616	4.3441
H	-0.7998	7.8082	-4.5563	5.7306	-6.8201	2.8843	4.0218	-8.1198	3.0228
H	0.5770	6.7453	-4.9691	5.8945	-5.1394	3.4702	4.7124	-6.6068	3.6780
H	0.9501	8.4684	2.1796	6.7116	-5.2073	-3.8837	5.9974	-6.7750	-3.5904
H	0.1266	7.8780	0.7105	5.9200	-5.5746	-2.3268	4.9574	-6.9097	-2.1463
H	-0.5296	7.4790	2.3134	4.9842	-5.6557	-3.8363	4.2246	-6.6324	-3.7419
H	2.7918	5.6185	-2.8069	6.3807	-2.5447	1.4862	6.1330	-4.2468	1.8565
C	3.5491	5.2300	0.0875	5.9719	-1.2185	-1.3024	6.6225	-3.0932	-0.8893

C	4.8341	5.0925	-0.7355	7.0144	-0.4157	-0.5153	7.6843	-2.5068	0.0468
C	5.2167	3.6260	-0.8898	6.3601	0.7717	0.1749	7.3157	-1.0841	0.4453
C	5.2001	2.9325	0.4709	5.4955	1.5525	-0.8130	6.9659	-0.2628	-0.7942
C	3.8606	3.1493	1.1732	4.4838	0.6306	-1.4869	5.8956	-0.9643	-1.6274
C	3.8367	2.5522	2.5667	3.6191	1.3211	-2.5371	5.5888	-0.2322	-2.9189
C	5.7223	6.2533	-2.6281	8.9319	-1.0425	0.7661	9.0144	-3.4079	1.8183
C	1.6122	1.7752	2.7375	1.4489	1.3687	-1.6681	3.2506	-0.4532	-3.1328
O	4.5781	5.7212	-1.9749	7.5876	-1.3254	0.4035	7.7587	-3.3814	1.1543
O	6.4961	3.5387	-1.4697	7.3549	1.6043	0.7258	8.3957	-0.4968	1.1312
O	5.4494	1.5568	0.2180	4.8298	2.5653	-0.0711	6.5200	1.0022	-0.3243
O	3.6559	4.5541	1.3103	5.2186	-0.3999	-2.1553	6.3920	-2.2529	-1.9857
O	2.5876	2.6867	3.2098	2.3195	0.7624	-2.6062	4.5224	-0.8138	-3.6399
H	3.3606	6.2775	0.3376	6.4587	-1.9581	-1.9444	6.9580	-4.0468	-1.3051
H	5.6437	5.6150	-0.2049	7.7786	-0.0473	-1.2156	8.6460	-2.4876	-0.4870
H	4.4558	3.1407	-1.5223	5.6928	0.3795	0.9587	6.4162	-1.1317	1.0800
H	6.0029	3.3550	1.0934	6.1331	2.0033	-1.5878	7.8711	-0.1424	-1.4078
H	3.0596	2.7138	0.5593	3.8342	0.1841	-0.7211	4.9832	-1.0711	-1.0245
H	4.5691	3.0810	3.1876	4.0937	1.1816	-3.5136	6.4728	-0.2811	-3.5651
H	4.1250	1.4960	2.5016	3.5594	2.3974	-2.3328	5.3745	0.8197	-2.6955
H	5.3599	6.7758	-3.5142	9.2522	-1.8485	1.4274	8.9459	-4.1827	2.5830
H	6.2389	6.9676	-1.9758	9.5746	-1.0300	-0.1224	9.8149	-3.6668	1.1149
H	6.4154	5.4606	-2.9174	9.0071	-0.0826	1.2815	9.2365	-2.4436	2.2801
H	0.8030	1.7641	3.4704	0.4900	0.8494	-1.6992	2.5086	-0.8044	-3.8532
H	1.2007	2.0785	1.7631	1.8574	1.3141	-0.6465	3.0447	-0.9249	-2.1613
H	2.0307	0.7639	2.6529	1.2862	2.4240	-1.9273	3.1713	0.6362	-3.0296
H	6.7819	2.6076	-1.4219	6.9062	2.2935	1.2503	8.1657	0.4351	1.3040
C	6.4620	0.9135	0.9440	5.2933	3.8767	-0.2481	7.1917	2.1466	-0.7740
C	7.2999	0.0964	-0.0440	5.2703	4.5887	1.1076	7.3940	3.0792	0.4238
C	6.4690	-1.0434	-0.6114	3.8357	4.7599	1.5778	6.0573	3.6256	0.8989
C	5.8214	-1.8426	0.5172	2.9717	5.3849	0.4842	5.2857	4.2250	-0.2741
C	5.0492	-0.9292	1.4686	3.1244	4.6354	-0.8398	5.1686	3.2225	-1.4212
C	4.4974	-1.6563	2.6812	2.4019	5.2878	-2.0056	4.5099	3.8048	-2.6580
C	9.0076	0.6754	-1.6084	6.6988	4.5528	3.0174	8.9479	3.0865	2.2351
C	4.0712	-0.1855	4.5085	3.4187	4.5644	-4.0345	5.0919	2.5870	-4.6157
O	7.7385	0.9868	-1.0488	6.0352	3.8060	2.0040	8.0487	2.3342	1.4303
O	7.2893	-1.8785	-1.3918	3.7944	5.5637	2.7341	6.2796	4.5988	1.8899
O	4.9559	-2.7700	-0.1212	1.6579	5.2747	1.0083	4.0062	4.5667	0.2391
O	5.9348	0.0853	1.9493	4.5099	4.5693	-1.1846	6.4810	2.7967	-1.7970
O	3.5561	-0.8563	3.3709	2.3310	4.4410	-3.1355	4.1507	2.7930	-3.5767
H	7.0943	1.6461	1.4558	6.3138	3.8739	-0.6441	8.1622	1.8787	-1.2041
H	8.1610	-0.3189	0.4993	5.7315	5.5781	0.9756	8.0276	3.9164	0.0973
H	5.6589	-0.6024	-1.2137	3.4303	3.7552	1.7778	5.4662	2.7825	1.2908
H	6.6072	-2.3737	1.0745	3.2506	6.4426	0.3643	5.8107	5.1259	-0.6245
H	4.2127	-0.4664	0.9251	2.7282	3.6221	-0.6972	4.5788	2.3582	-1.0818
H	5.3269	-1.9484	3.3398	2.9005	6.2363	-2.2559	5.1868	4.5379	-3.1193
H	3.9917	-2.5659	2.3465	1.3724	5.4989	-1.7049	3.5980	4.3270	-2.3558
H	9.2272	1.4557	-2.3378	7.2683	3.8370	3.6107	9.3939	2.3869	2.9428
H	9.7817	0.6799	-0.8320	7.3863	5.2814	2.5718	9.7390	3.5291	1.6184
H	8.9876	-0.3002	-2.0988	5.9759	5.0719	3.6501	8.4183	3.8757	2.7729
H	3.3271	0.5626	4.7977	3.1926	3.9282	-4.8925	4.7842	1.6815	-5.1440
H	4.2281	-0.8862	5.3380	3.5237	5.6021	-4.3769	5.0947	3.4339	-5.3136
H	5.0142	0.3216	4.2791	4.3616	4.2427	-3.5813	6.1022	2.4441	-4.2189
H	6.7519	-2.6435	-1.6692	2.8605	5.7983	2.8856	5.4191	5.0048	2.1043
C	5.0841	-4.1318	0.1812	0.7155	6.2764	0.7558	3.5360	5.8755	0.0662
C	4.9887	-4.9116	-1.1351	0.0311	6.6023	2.0894	2.9222	6.3276	1.3957
C	3.5850	-4.8023	-1.7150	-0.8404	5.4329	2.5378	1.6682	5.5206	1.7008

C	2.5585	-5.1526	-0.6447	-1.7085	4.9261	1.3880	0.7407	5.5426	0.4927
C	2.7607	-4.3183	0.6169	-0.8572	4.6150	0.1611	1.4587	5.0974	-0.7774
C	1.7800	-4.6605	1.7295	-1.6136	4.0774	-1.0429	0.5706	5.1651	-2.0125
C	6.4952	-5.2980	-2.9472	0.6950	7.6973	4.1030	3.8457	7.0893	3.4618
C	2.6058	-6.7628	2.4573	-2.7487	5.9558	-1.9270	0.4564	7.5087	-2.3763
O	5.9747	-4.3791	-1.9953	1.0751	6.8926	2.9955	3.9202	6.1666	2.3827
O	3.4533	-5.6781	-2.8113	-1.6674	5.8299	3.6099	1.0162	6.0683	2.8232
O	1.2963	-4.9193	-1.2437	-2.3689	3.7949	1.9254	-0.3301	4.6736	0.8158
O	4.0946	-4.5471	1.0884	-0.2202	5.8483	-0.2019	2.5949	5.9474	-0.9752
O	1.5345	-6.0416	1.8770	-2.8548	4.7029	-1.2775	-0.2436	6.3157	-2.0740
H	6.0457	-4.3323	0.6649	1.1958	7.1710	0.3448	4.3540	6.5465	-0.2150
H	5.2025	-5.9691	-0.9199	-0.6091	7.4854	1.9454	2.6484	7.3888	1.3013
H	3.4217	-3.7560	-2.0190	-0.1702	4.6111	2.8378	1.9699	4.4771	1.8846
H	2.6642	-6.2168	-0.3919	-2.4424	5.6998	1.1209	0.3676	6.5670	0.3560
H	2.6399	-3.2528	0.3717	-0.0890	3.8732	0.4208	1.7989	4.0581	-0.6561
H	0.8165	-4.2029	1.4929	-1.8256	3.0179	-0.8794	-0.1169	4.3148	-1.9980
H	2.1571	-4.2288	2.6699	-0.9591	4.1627	-1.9235	1.2103	5.0914	-2.9056
H	7.2788	-4.7715	-3.4929	1.6084	7.9178	4.6565	4.6949	6.8779	4.1124
H	6.9301	-6.1689	-2.4426	0.2463	8.6372	3.7594	3.9217	8.1190	3.0929
H	5.7160	-5.6289	-3.6370	-0.0159	7.1749	4.7471	2.9115	6.9681	4.0144
H	2.2268	-7.7565	2.7016	-3.7613	6.2694	-2.1862	-0.2923	8.2704	-2.6002
H	3.4565	-6.8559	1.7735	-2.2848	6.7106	-1.2832	1.0772	7.8440	-1.5383
H	2.9546	-6.2728	3.3759	-2.1531	5.8662	-2.8462	1.1048	7.3704	-3.2513
H	2.5000	-5.7806	-2.9870	-2.3467	5.1389	3.7133	0.1075	5.7155	2.8355
C	0.2576	-5.8198	-0.9607	-3.6824	3.5433	1.5102	-1.6305	5.0602	0.4538
C	-0.3901	-6.2128	-2.2962	-4.4854	3.1180	2.7513	-2.5112	4.9100	1.6969
C	-1.1617	-5.0372	-2.8823	-4.1013	1.7093	3.2012	-2.6505	3.4401	2.0694
C	-2.0736	-4.4198	-1.8233	-4.0782	0.7671	1.9980	-3.0345	2.6086	0.8471
C	-1.2581	-4.0423	-0.5916	-3.1180	1.3274	0.9589	-2.0990	2.8761	-0.3282
C	-2.0470	-3.4145	0.5465	-2.8368	0.4612	-0.2651	-2.5513	2.1854	-1.6015
C	0.2877	-7.5784	-4.1339	-5.2799	4.2729	4.6802	-2.7719	6.1771	3.7064
C	-1.0512	-1.2876	0.8066	-0.8188	-0.5760	0.3977	-0.5483	1.3599	-2.5301
O	0.6650	-6.6473	-3.1293	-4.2344	4.0998	3.7346	-1.8905	5.6695	2.7142
O	-1.9254	-5.4638	-3.9872	-5.0162	1.2491	4.1716	-3.6292	3.3073	3.0746
O	-2.6681	-3.2842	-2.4348	-3.6728	-0.5111	2.4575	-2.9806	1.2450	1.2439
O	-0.6760	-5.2451	-0.0872	-3.7042	2.5543	0.5162	-2.1131	4.2754	-0.6023
O	-1.2212	-2.5862	1.3456	-1.4461	0.3234	-0.4933	-1.6065	2.2899	-2.6433
H	0.6460	-6.7032	-0.4474	-4.1206	4.4369	1.0570	-1.6384	6.0919	0.0924
H	-1.0925	-7.0388	-2.1088	-5.5519	3.1128	2.4782	-3.5077	5.3171	1.4700
H	-0.4306	-4.2680	-3.1792	-3.0800	1.7500	3.6124	-1.6687	3.0857	2.4204
H	-2.8459	-5.1468	-1.5339	-5.0804	0.7067	1.5491	-4.0597	2.8792	0.5496
H	-0.4613	-3.3495	-0.8951	-2.1529	1.5447	1.4361	-1.0828	2.5583	-0.0585
H	-2.4343	-4.2068	1.1935	-3.2443	0.9535	-1.1488	-3.4653	2.6809	-1.9489
H	-2.9004	-2.8525	0.1562	-3.3274	-0.5180	-0.1716	-2.7853	1.1329	-1.3842
H	1.2052	-7.8790	-4.6411	-4.9870	5.1048	5.3217	-2.1691	6.7964	4.3715
H	-0.1796	-8.4622	-3.6827	-6.2203	4.5237	4.1740	-3.5513	6.7966	3.2465
H	-0.4054	-7.1286	-4.8482	-5.4236	3.3713	5.2799	-3.2396	5.3668	4.2699
H	-0.2893	-0.7813	1.4041	0.2525	-0.3646	0.3854	0.0780	1.4725	-3.4165
H	-0.7127	-1.3184	-0.2374	-1.1922	-0.4652	1.4249	0.0692	1.5374	-1.6399
H	-1.9816	-0.7044	0.8606	-0.9781	-1.6145	0.0730	-0.9345	0.3321	-2.4880
H	-2.4200	-4.6902	-4.3146	-4.6975	0.3830	4.4855	-3.7377	2.3562	3.2680
H	-4.4757	-1.4853	0.7873	-4.2268	-2.7468	-1.0636	-3.5661	-1.3246	-2.1320
C	-5.3278	1.3332	3.1631	-2.6579	2.7743	-4.0232	-9.0641	-2.6318	-0.5635
C	-0.8366	-1.2058	4.2399	-5.4032	-1.3552	-5.7840	-6.0494	1.1899	-2.3051
C	-4.3416	2.6996	5.3815	-0.7743	0.7313	-4.1185	-10.2826	-1.8175	-2.9254
C	-5.4598	2.7199	3.2496	-1.3427	3.0519	-4.3988	-9.6011	-3.5794	-1.4357

C	-1.0060	0.3954	6.4704	-3.0433	-2.6355	-5.1369	-8.1644	1.5397	-4.0440
C	0.3321	-0.8001	4.8754	-5.0114	-2.4771	-6.5081	-5.8052	1.6572	-3.5925
C	-4.9722	3.4070	4.3571	-0.3963	2.0343	-4.4465	-10.2075	-3.1774	-2.6203
C	0.2463	0.0117	6.0037	-3.8257	-3.1271	-6.1776	-6.8735	1.8453	-4.4654
C	-4.3380	-4.9260	3.3373	-7.8726	0.1237	-1.2435	-7.0782	2.5562	2.7256
C	-5.6929	-1.4562	3.4855	-5.0573	2.2338	-2.5301	-8.5507	-0.6346	1.4096
C	-3.3177	-2.8746	4.0198	-6.2252	-0.3137	-2.9093	-7.4053	1.7643	0.4792
C	-5.6575	-2.9578	3.6831	-6.0477	1.6344	-1.5434	-8.4371	0.6080	2.2799
C	-3.5090	0.5093	6.3729	-2.5109	-0.9536	-3.3450	-9.8091	0.6154	-2.3681
C	-4.7098	0.6230	4.1976	-3.0375	1.4706	-3.6879	-9.1314	-1.2693	-0.8699
C	-2.1059	-0.8170	4.6931	-4.6329	-0.8627	-4.7264	-7.3470	0.9074	-1.8631
C	-4.2097	1.3157	5.3131	-2.0759	0.4383	-3.7251	-9.7641	-0.8602	-2.0605
C	-2.1882	-0.0084	5.8387	-3.4308	-1.5108	-4.4054	-8.4182	1.0703	-2.7525
C	-3.2865	-1.3468	3.8741	-5.2362	0.2794	-3.9242	-7.4718	0.5091	-0.4015
N	-4.3768	-3.4763	3.2255	-6.9676	0.7232	-2.2103	-7.2747	1.3892	1.8803
N	-4.6112	-0.7998	4.2206	-4.3205	1.1573	-3.1843	-8.6663	-0.2555	0.0052
H	-5.7128	0.8173	2.2876	-3.3856	3.5781	-3.9731	-8.5882	-2.9599	0.3548
H	-0.7712	-1.8408	3.3613	-6.3320	-0.8494	-6.0381	-5.2202	1.0137	-1.6230
H	-3.9557	3.2256	6.2507	-0.0309	-0.0625	-4.1342	-10.7647	-1.4915	-3.8433
H	-5.9474	3.2591	2.4428	-1.0606	4.0711	-4.6474	-9.5382	-4.6330	-1.1829
H	-1.0797	1.0327	7.3476	-2.1083	-3.1262	-4.8782	-8.9981	1.6549	-4.7313
H	1.2981	-1.1040	4.4789	-5.6282	-2.8377	-7.3246	-4.7872	1.8603	-3.9124
H	-5.0793	4.4847	4.4253	0.6349	2.2488	-4.7082	-10.6255	-3.9119	-3.3005
H	1.1439	0.3464	6.5147	-3.5062	-4.0038	-6.7317	-6.7031	2.2077	-5.4739
H	-5.1185	-5.3639	2.7093	-8.4432	0.9064	-0.7362	-7.0330	2.2432	3.7726
H	-3.3679	-5.3001	2.9978	-8.5731	-0.5440	-1.7524	-6.1257	3.0369	2.4777
H	-4.4932	-5.2697	4.3752	-7.3303	-0.4617	-0.4769	-7.8908	3.2955	2.6213
H	-6.6403	-1.0515	3.8540	-4.3508	2.8697	-1.9863	-9.4414	-1.2013	1.6926
H	-5.6433	-1.2516	2.4075	-5.6019	2.8575	-3.2604	-7.6651	-1.2713	1.5896
H	-2.3591	-3.2749	3.6780	-6.9349	-0.9623	-3.4336	-6.5368	2.3657	0.1865
H	-3.4325	-3.1185	5.0951	-5.6451	-0.9448	-2.2028	-8.3094	2.3767	0.2966
H	-5.8274	-3.2013	4.7493	-5.4834	1.1086	-0.7499	-9.3676	1.1989	2.1921
H	-6.4619	-3.4178	3.0993	-6.6220	2.4346	-1.0636	-8.3236	0.3086	3.3272
H	-3.3265	1.1229	7.2587	-1.6498	-1.6147	-3.2275	-10.5051	0.8142	-3.1866
H	-4.1487	-0.3318	6.6605	-3.0351	-0.9105	-2.3834	-10.1535	1.1547	-1.4785
H	-3.0694	-1.1308	2.8098	-5.8260	0.8954	-4.6271	-6.5754	-0.0876	-0.1617

References

(1) *HyperChem(TM) Professional*, version 8.0.10. 1115 NW 4th Street, Gainesville, Florida 32601, USA: Hypercube, Inc; 2011.

(2) Stewart, J. J. P. *MOPAC2016*, Stewart Computational Chemistry, 2016, http://OpenMOPAC.net (accessed October 15, 2019).

(3) Klamt, A.; Schüümann, G. COSMO: a New Approach to Dielectric Screening in Solvents with Explicit Expressions for the Screening Energy and Its Gradient. *J. Chem. Soc., Perkin Trans. 2* **1993**, 799–805.

(4) Allouche, R. *Gabedit 2.5.1*, Gabedit - A Graphical User Interface for Computational Chemistry Softwares. *J. Comput. Chem.* **2011**, *32*, 174-182.

(5) Frisch, M. J.; Trucks, G. W.; Schlegel, H. B.; Scuseria, G. E.; Robb, M. A.; Cheeseman, J. R.; Scalmani, G.; Barone, V.; Mennucci, B.; Petersson, G. A., et al. *Gaussian 09*, Revision D.01; Gaussian, Inc.: Wallingford, CT, 2013.

(6) Becke, A. D. Density-Functional Thermochemistry. III. The Role of Exact Exchange. *J. Chem. Phys.* **1993**, *98*, 5648-5652.

(7) Grimme, S. Semiempirical GGA-Type Density Functional Constructed with a Long-Range Dispersion Correction. *J. Comput. Chem.* **2006**, *27*, 1787-1799.

(8) Tomasi, J.; Mennucci, B.; Cammi, R. Quantum Mechanical Continuum Solvation Models. *Chem. Rev.* **2005**, *105*, 2999-3093.

(9) Tantillo, D. J. Chemical Shift Repository, http://cheschirenmr.info/Instructions.htm (accessed November 21, 2015).

(10) Ignaczak, A.; Porwanski, S.; Szyszka, M. Deeper Insight into the Properties of the Newly Synthesized Macrocycles as Drug Receptors – Some Preliminary Quantum Chemical Studies. *New J. Chem.* **2017**, *41*, 521-529.

(11) Yamamoto, Y.; Onda, M.; Takahashi, Y.; Inoue, Y.; Chûjô, R. Two-Dimensional N.M.R. Spectra of *O*-Methylated Cyclomalto-Oligosaccharides: Assignment of ^{1}H and ^{13}C Resonances and Conformational Analysis. *Carbohydr. Res.* **1987**, *170*, 229-234.

(12) Correia, I.; Bezzenine, N.; Ronzani, N.; Platzer, N.; Beloeil, J.-C.; Doan, B.-T. Study of Inclusion Complexes of Acridine with β- and (2,6-di-*O*-Methyl)-β-cyclodextrin by Use of Solubility Diagrams and NMR Spectroscopy. *J. Phys. Org. Chem.* **2002**, *15*, 647-459.

(13) Belica, S.; Jeziorska, D.; Urbaniak, P.; Buko, V. U.; Zavodnik, I. B.; Pałecz, B. Calorimetric and Spectroscopic Characterization of Complexes Between β-Cyclodextrin or Heptakis(2,6-di-*O*-methyl)-β-cyclodextrin and Sertraline Hydrochloride in Aqueous Solution. *J. Chem. Thermodynamics* **2014**, *70*, 160–167.

(14) Szejtli, J.; Lipták, A.; Jodál, I.; Fügedi, P.; Nánási, P.; Neszmélyi, A. Synthesis and ^{13}C-NMR Spectroscopy of Methylated Beta-cyclodextrins. *Starch-Stärke* **1980**, *32*, 165-169.
